# Collaborative and integrated working between general practice and community pharmacies: A realist review of what works, for whom, and in which contexts

**DOI:** 10.1177/13558196241290923

**Published:** 2024-10-23

**Authors:** Emily Owen-Boukra, Ziyue Cai, Claire Duddy, Nina Fudge, Julia Hamer-Hunt, Fran Husson, Kamal R Mahtani, Margaret Ogden, Deborah Swinglehurst, Malcolm Turner, Cate Whittlesea, Geoff Wong, Sophie Park

**Affiliations:** 1 NIHR SPCR Research Fellow, Department of Primary Care and Population Health, University College London, London, UK; 2 NIHR SPCR Intern, Department of Primary Care and Population Health, 4919 University College London, London, UK; 3 Pre-Doctoral Fellow, Nuffield Department of Primary Care Health Sciences, University of Oxford, Oxford, UK; 4 THIS Institute Research Fellow and Lecturer, Wolfson Institute of Population Health, 4617 Queen Mary University of London, London, UK; 5 PPI Co-applicant, Department of Primary Care and Population Health, 4919 University College London, London, UK; 6 GP and Professor of Evidence Based Healthcare, Nuffield Department of Primary Care Health Sciences, 6396 University of Oxford, Oxford, UK; 7 GP and Professor of Primary Care, Wolfson Institute of Population Health, 4617 Queen Mary University of London, London, UK; 8 Professor of Pharmacy Practice and Director, UCL School of Pharmacy, 4919 University College London, London, UK; 9 GP and Associate Professor of Primary Care, Nuffield Department of Primary Care Health Sciences, 6396 University of Oxford, Oxford, UK; 10 GP and Professor of Primary Care and Clinical Education, Nuffield Department of Primary Care Health Sciences, 6396 University of Oxford, Oxford, UK; 11 Honorary Professor of Primary Care and Medical Education, Department of Primary Care and Population Health, University College London, London, UK

**Keywords:** community pharmacies, general practice, realist review

## Abstract

**Objectives:**

Collaborative and integrated (C + I) working between general practice and community pharmacies has the potential to increase accessibility to services, improve service efficiency and quality of care, and reduce health care expenditures. Many existing studies report challenges and complexities inherent in establishing effective C + I ways of working. The aim of our review is to understand how, when and why working arrangements between General Practitioners (GP) and Community Pharmacists (CP) can provide the conditions necessary for effective communication, decision-making, and C + I working.

**Methods:**

We conducted a realist review to explore the key contextual factors and mechanisms through which GP-CP C + I working may be achieved. MEDLINE, Embase, CINAHL, PsycINFO, HMIC, Web of Science, IBSS, ASSIA, Sociological Abstracts, Sociology Database and the King’s Fund Library Database were searched for articles and grey literature published between January 2000 and April 2022.

**Results:**

A total of 136 documents were included in the final synthesis. Our findings highlight the importance of mutually beneficial remuneration models to support effective integration of services; supportive organisational cultures and values; flexible and agile IT systems/technologies; adequate physical infrastructure and space design to support multidisciplinary teamworking; the importance of establishing patient’s trust in collaborative processes between GP-CP; and the need to acknowledge, support and utilise effective triadic relationships.

**Conclusions:**

Our research generates new insights regarding how, why and in which contexts C + I working can be achieved between GPs and CPs. The findings of our review can be used to inform future policy, research and clinical practice guidelines for designing and delivering C + I care.

## Introduction

The organisation of international and United Kingdom (UK) primary care is rapidly changing. Implementation of the National Health Service (NHS) Long-Term Plan^1^ and additional Workforce Plan^2^ formalises expectations for collaborative and integrated (C + I) working across systems, professional boundaries and institutional organisations.

Many health care organisations, including general practice and community pharmacies, have been encouraged to transition *away* from organisational autonomy and competition, and *towards* more C + I ways of working. General Practitioner (GP) and Community Pharmacist (CP) C + I working has been reported (UK and internationally) to achieve greater resource efficiency, increase the continuity of service delivery, and improve the quality and safety of patient care.^3–5^ Many recognise that most health care objectives cannot be achieved by a single health care professional or an organisation working in isolation.^6^ With increasingly dynamic and under-resourced health care environments, single approach and/or single-solution interventions are no longer sufficient. Instead, the provision of safe, effective and equitable patient care requires innovative solutions and the synergistic integration of knowledge, skills and expertise from diverse health care professionals and service users.^3,6,7^

Despite increased interest in C + I working, inconsistencies remain in the definitions and terminology used. Some definitions indicate that collaboration involves common goals, values and objectives, mutual trust, communication, and shared decision-making, along with opportunities to identify, explore and reconcile differences.^7^ Integration is often used to mean a combined set of organisational methods, processes and structures (e.g. common management policies and incentives, defined referral and governance processes) seeking to achieve high quality and efficient care.^8^ Collaboration and integration are not synonymous, but effective collaboration between health care professionals is commonly referred to as a prerequisite for improving the integration of care systems.

Existing evidence has shown that C + I working between GPs-CPs can lead to effective and equitable solutions while increasing the efficiency of services and reducing health inequalities.^9^ In recent years, there have been several initiatives aimed at improving GP-CP collaboration and integration. Examples include the provision of influenza vaccinations, medication use reviews, minor aliment schemes, smoking cessation interventions, and joint diabetes monitoring.^10,11^ Despite such advances, relatively little is known about how and under what circumstances C + I working arrangements are best achieved. The reported barriers to collaboration and integration include limited financial remuneration models, time constraints, lack of readiness to change among professionals and users, geographical separation, cultural diversity, and turbulent and competitive contexts.^12,13^ Better understanding is needed of the causal processes that support GP-CP C + I working: what works, for whom, when and why. The aim of our review is to understand how, when and why working arrangements between GPs-CPs can provide the conditions necessary for effective communication, decision-making and C + I working.

## Methods

A realist review is a theory-driven interpretive approach to synthesising grey literature, qualitative, quantitative and mixed-methods evidence.^14^ The approach aims to identify causal pathways through which complex social interventions (e.g. C + I initiatives) work. To do this, a realist review examines the dynamic interplay between contextual factors, underlying mechanisms and outcomes of interest.^15^ The approach aims to move beyond a focus on ‘does it work?’ towards exploring ‘how something may work, for whom, under which circumstances, and why’.^14^ A realist review is particularly suitable for this research because C + I initiatives are inherently complex, with many factors affecting their success. For instance, collaboration and integration may work in some contexts, but not in others, because the responses of GPs, CPs, and patients may vary, depending on factors such as the culture of an organisation and the professional identities and ideological values of health care professionals. Our review aims to explore the wider human, policy, regulatory, and professional elements that can influence working relationships and processes to support or inhibit collaboration and integration. Our methods are outlined below (see our protocol for further detail).^16^ The review was carried out in accordance with the Realist and Meta-narrative Evidence Synthesis: Evolving Standards (RAMESES) quality and reporting standards^17,18^ and has been registered on PROSPERO (CRD42022314280). We followed Pawson’s five iterative stages (finding existing theories, searching for evidence, selecting articles, extracting data, and synthesising evidence and drawing conclusions).^14^

**Figure 1.**
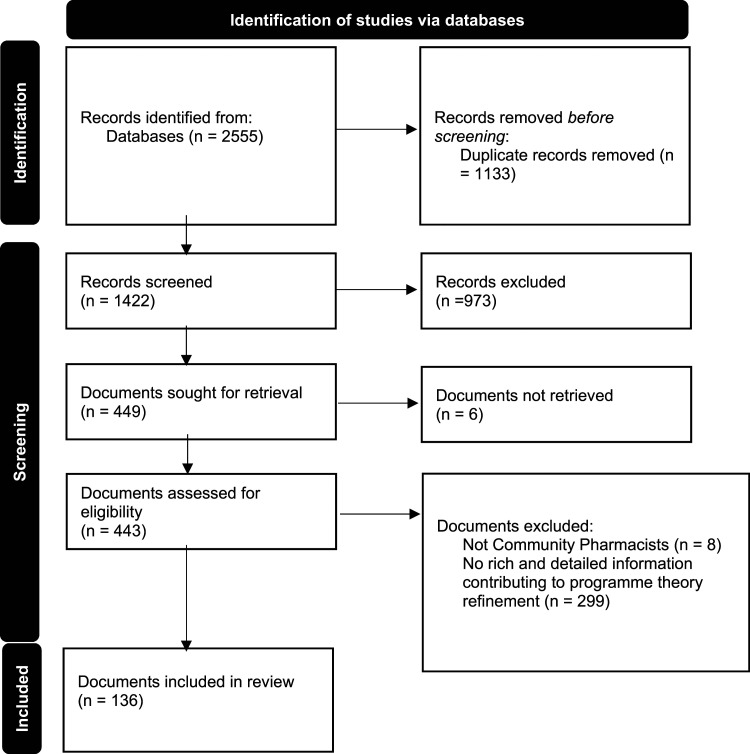
PRISMA summary of searching and selection processes.

To develop our initial programme theory, we consulted with key content experts in our stakeholder group (representatives from different community pharmacies and general practice organisation types) and informally scoped existing literature identified via citation tracking and snowballing. Our formal searches were then carried out in nine databases (by CD): MEDLINE (Ovid), Embase (Ovid), CINAHL (EbscoHost), PsycINFO (Ovid), HMIC (Ovid), International Bibliography of the Social Sciences (Proquest), Sociology Collection (Proquest, comprising ASSIA, Sociological Abstracts, Sociology Database), Web of Science (SCIE, SSCI, ESCI, CPCI) and the King’s Fund Library database (see S1 in the online supplement for more details). We searched for articles and grey literature published between January 2000 and April 2022. A PRISMA summary of the included studies is shown in Figure 1.

The formal searches (conducted in April 2022) resulted in 2555 potentially relevant documents. Our inclusion and exclusion criteria can be found in S2 in the online supplement. In line with RAMESES guidelines, documents were assessed based on their relevance (whether they contained data which contributed to programme theory (PT) refinement) and rigour (whether the methods used to generate the relevant data were credible and trustworthy – for example, depending on the type and source of document, appropriate quality standards were used in addition to multidisciplinary team discussions).^14,17^

Through prolonged engagement with the literature, we modified and refined our PT (see S3 in the online supplement) by interrogating data sources and forming context-mechanism-outcome configurations (CMOCs). The characteristics of included documents were extracted (by ZC and EO-B) into a Microsoft Excel spreadsheet, including bibliographic data and information about the study methodology, focus of the paper, intervention, solutions proposed, descriptive information regarding GPs-CPs, collaboration, integration, the patient perspective and an overall summary of findings. Data interpreted by the authors (EO-B, SP, CD, NF, GW) as relevant to contexts, mechanisms, or outcomes were coded, and a realist logic of analysis was used to synthesise these data. A document was formulated listing CMOCs with narrative summaries and direct quotes relating to each configuration (EO-B, NF, CD, ZC). Emerging analytical findings and CMOCs were then critically discussed with the research team (EO-B, SP, CD, NF, GW, CW, DS, ZC, KRM), including patient and public involvement (PPI) co-applicants (JH-H, FH, MO, MT). This helped to modify, expand and refine the CMOCs where appropriate (CMOCs and supporting evidence can be found in S4 in the online supplement).

### Patient, public and stakeholder involvement

From project inception, we purposively invited a diverse group of professionals, including those from remote/urban, policy/practitioner, and large multiple/independent, small/medium organisations, to gain a wide range of perspectives on C + I working. The reflective PPI/stakeholder dialogue informed the selection of texts, interpretation of data, consolidation of findings, and production of recommendations to maximise relevance and utility for policy, practice and patient care. For example, PPI/stakeholders informed ways of thinking about patient care in relation to access, help-seeking behaviour, therapeutic relationships, continuity, and the role of the patient as both ‘broker’ and ‘user’, as they play a potential mediating and managing role, between GP and CP.

## Results

We included 136 documents (see Figure 1) published between 2000-2021: 124 published research articles, four conference materials (abstracts) and eight others (e.g. policy reports and guidance articles) (see S5 in the online supplement for included document characteristics).

Our refined PT comprises 26 CMOCs with six overarching headings: (1) historical norms, (2) infrastructure, (3) knowledge sharing, (4) interprofessional relationships, (5) beyond the dyad - supporting patient involvement and engagement, and (6) unanticipated consequences. Collectively, these findings generate new insights regarding the contexts and mechanisms through which GP-CP C + I working may be achieved. Illustrative data excerpts from included documents with narrative summaries are provided below (see S4 online supplement for all supporting evidence and extracts). Where appropriate, substantive or formal theories about C + I working phenomenon are incorporated to increase explanatory power and provide cumulative explanations.^19^

### Historical norms

Historically, GPs and CPs have operated in professional silos, with infrequent formal communication and collaboration. GP-CP collaboration and integration is not suitable for all working. However, where introduced, challenges must be addressed to overcome historical norms, patterns, and traditional ways of working. The reviewed literature identified barriers in relation to the perceived scope of CP practice.^20,21^ For example, CPs were reluctant to make clinical decisions where they felt less knowledgeable, due to a lack of confidence or perception that this was beyond their scope of practice:

We’re not doctors, and whilst we can interpret things, I think a lot of that should be left to doctors … Basically, managing people’s medicine, I think is far more important than being able to prescribe. I think, do that, and do it well, and prescribing on the more basic things (CP).^20^

We identified both self (CP) and ‘other’ (GP) perceptions of CP professional responsibilities. For instance, some GPs expressed concerns that CPs may not have the necessary experience or training required to diagnose, especially complex and undifferentiated illness.^22^ This included the importance of CPs recognising ‘red flags’ or ‘unknowns’ while remaining within their areas of competence:

[A] red eye 99 times out of 100 will be conjunctivitis, but it might be acute glaucoma, and if you miss that somebody loses the sight in their eye … So, I think you need your most senior people dealing with these difficult things (GP).^22^

There was a concern among CPs that making decisions historically made by other health care professionals, could result in jurisdictional conflict:^20,23^

You’re stepping into a more clinical report, and we have to … try and straddle that without stepping on too many toes. The service is still very new, and there are some GPs that are very welcoming of the service, and there are some that appear to be feeling threatened (CP).^20^

In some contexts, there was a fear that both GPs and CPs could be motivated by performance management approaches and financial incentives, rather than patient care. For instance, there was an acknowledgement among GPs that the ‘tick box’ approach of the UK Qualities and Outcomes Framework and Care Quality Commission inspections had the potential to create tensions between financial goals and patient care.^22^ Similarly, some perceived a ‘shopkeeper’ image of CPs, operating in a transactional mode. Consequently, some GPs were reluctant to collaborate because of the concern that CPs were motivated by profit, which could prevent them from acting in the best interests of the patient:^20,22,24,25^

Typically, if you start entering targets and it becomes a financial incentive and then when you hear about [large pharmacy chain] performing medication use reviews on each other ... I get a bit more sceptical about the use, whether it’s actually for patient focus and quality (GP).^22^

The fear of financial competition was also identified. In contexts in which GPs perceived CP services as a threat to their income, clinical autonomy, and/or expertise, they were less likely to engage and cooperate with CPs, because they felt threatened:^20,26–28^

It bothers me that they [CPs] want to take something away from the GPs. Not only asthma, but they will also take hypertension, they will take cholesterol ...We have studied for nine years. Pharmacist [studies] take five years, and now they are completely invading our domain (GP).^26^

I set up that meeting at the practice, just to explain what an LPS [Local Pharmaceutical Service] was and what we were trying to do and what we were trying to achieve and how the pharmacist was there to help and support the practice in managing that group of patients and they [GPs] said, ‘We engage terribly well with our patients, we do not need any help and we would appreciate it if you didn’t interfere with us’ (Pharmaceutical Advisor).^28^

Without effective collaboration and integration, GPs and CPs were often unaware of one another’s clinical priorities, demands and treatment plans. This could lead to conflicting messages or management plans being proposed to patients, causing ambiguity and frustration:

Sometimes the pharmacist will say something that contradicts what I say to the patient or give medication information that scares the patient from taking what I’ve prescribed (GP).^29^

### Infrastructure

Service remuneration models were a significant identified barrier to GP-CP C + I working. For many GPs and CPs, financial remuneration was considered essential for C + I sustainability, given the time, energy, and resources needed to develop and maintain trusting collaborative relationships.^11–13,27,30^ In contexts where GPs-CPs were not contracted or remunerated to provide C + I care, this typically led to competition and a reluctance to work together on collaborative tasks, perceiving collaboration as not worthwhile.^13,26–28,31^ Remunerating CP-GP joint working can support effective integration of services.^25,30^ For instance, some GPs described how adequate remuneration could enable the development of an interprofessional adherence support strategy.^29,32,33^ Both GPs and CPs described the importance of remuneration systems in supporting collaboration:

I think remuneration being what it is, it’s not a good viable model. It’s ‘one size fits all’… But there’s an increasingly complex cohort, if you really want that person-centred care, then there has to be a change in the remuneration structure … We really need to have the capacity to provide a more in-depth service for those who warrant it, and they should be remunerated accordingly … That’s why you end up having people [pharmacists] feeling disillusioned because they end up putting more in than the remuneration is providing for (CP).^33^

Organisational leadership and management support provided crucial mediation and facilitation for GP-CP collaboration.^34^ When organisations established a culture that valued and promoted C + I working, GPs and CPs were more likely to actively choose to work with one another on collaborative tasks:

[Management] really support us doing these things … these new services. [The manager] helps us find help to get started and points us in the right direction with how to track and record (CP).^34^

Signalling is one theory identified in the literature to explain how management can signal the importance of C + I working by fostering the values, beliefs, and behaviours needed to create a culture of trust and partnership.^35^ In contrast, when an organisation establishes a culture that does not value or prioritise C + I working, collaborative relationships are unlikely to be sustained:

They designated a new manager, and, at the beginning, we explained to her everything we had been doing and everything looked fine to her ‘very good, very good, very good,’ but we had neither meetings, nor health controls … everything diminished (CP).^13^

Where GP-CP C + I working was needed, the literature pointed to the importance of moving *away* from fixed and rigid systems and *towards* more flexible and agile approaches to accommodate complexity, innovation, and change.^31,36^ In contexts where IT systems and technologies used for collaboration were inflexible and could not easily be adapted to fit professional practice and patients’ unique needs, this could lead to the avoidance and abandonment of technological systems.^37–39^ Flexible and agile systems were considered crucial to enable GPs and CPs to electronically communicate information in a timely and responsive manner (e.g. changes in patients’ conditions). This enhanced agility could facilitate the interdisciplinary integration of resources and knowledge, and ensure that CPs-GPs expertise can be fully utilised.^30^ For instance, flexible systems could adapt to different professional lenses in the ways information was prioritised or recorded (and related clinical priorities, routines, safety nets), as well as adaptation to particular patient needs.

Establishing formal and informal arenas for communication, networking and interpersonal dialogue was identified in the literature as key to support CP-GP C + I working.^12^ Effective communication was considered critical to fostering mutual trust and understanding and ensuring the delivery of safe and efficient patient care.^37,40^ However, several contextual factors could be inimical to communication and discourage C + I working. For instance, in contexts where GPs and CPs had multiple demands on their time,^11,29,41^ they were more reluctant to take on additional tasks due to a lack of capacity.

### Knowledge sharing

Trusting professional relationships were considered essential to achieving C + I working, and could be established through repeated, frequent, respectful interactions and behaviours.^11,27,38^ The presence or absence of a close personal and working relationship impacted how comfortable GPs felt collaborating and sharing information: ‘It’s a question of manners and of existing relationships … Trust is a premise, isn’t it?’ (GP).^40^

GPs who described a close and established working relationship with their CP were more likely to appreciate CPs’ complementary expertise and specialist knowledge, and to have confidence in their clinical abilities:

Having worked with the pharmacists for long enough now … My confidence in their ability has grown. At first, you know, I wanted them [CPs] to explain exactly all the details of the case and what the alternative treatments were and what the alternative diagnoses were, but now we’ve worked with each other for a bit longer, I think I have more confidence in their clinical ability (GP).^28^

I soon got to know them [GPs], and once I had suggested a few drug changes, not just the anginal drugs but others as well, they started to ask my advice, as did the other practice staff (CP).^42^

When GPs and CPs did not have an established trusting working relationship, they were likely to limit sharing information and/or electronic records with each other, because of safety concerns:^28,38^

I wouldn’t be happy for it [repeat dispensing] unless I had a close relationship with the pharmacist, and I trusted them to tell me if things are going wrong or if patients aren’t picking up prescriptions … I think if the chemist didn’t know the patients, didn’t know the GP, it could be a very dangerous system indeed (GP).^38^

In contexts where GPs and CPs shared relevant information and electronic records with one another, this could avoid duplication of work/effort, eliminate gaps and facilitate shared decision-making, because both GPs and CPs could develop a common understanding of issues and patient care needs.^31,40^ For example, some GPs described the importance of receiving information from CPs about potential medication interactions and patient adherence.^40,43^ There was recognition among GPs that CPs may be able to discover important information about patients given their more frequent contact:

We have had chats about 15 or 20 patients in whom we weren’t aware of side effects, or poor compliance, or we weren’t aware of them not having had their cholesterol checked ... Some minor points, but when they add up in those sorts of numbers, they become significant … In fact, he [CP] picked up a couple of people who unfortunately had impotence with their drug therapy and we didn’t know - he by the way gave them very good advice. We changed one person’s medication, and we referred one for special counselling and treatment (GP).^42^

A potential barrier to information sharing was the lack of integrated communication strategies.^13,20,39,40,43^ In contexts where CPs did not have an easy to use/reliable means of communicating with GPs or accessing elements of patient records, this could constrain the advice and management CPs could provide to patients, due to a lack of relevant information:

I don’t see how you could do it without clinical information … It just gently reminds you that you are doing the best to the best of your ability, but you are kind of working with your hands behind your back (CP).^44^

### Interprofessional relationships

Some factors could prevent opportunities for informal GP-CP reflection and dialogue. These include geographical distance, long opening hours, CP requirements to be on the pharmacies’ premises all or most of the time, and being an online-only pharmacy. In such contexts, this could undermine patient care, making it harder to establish trust and develop the relationships needed to support C + I working:^13,40^

Sometimes, it gets as late as half seven, and we close. So, you can’t really go [to meetings with GPs] after work, ‘go before work because we open at 8.45, and we don’t close for lunch (CP).^45^

If there is a new GP in my neighbourhood, I make an appointment and then I introduce myself so that the general practice knows who we are when we are calling. I think this custom is getting lost—I am always surprised when a GP answers, ‘I appreciate your visit, so far none of your colleagues stopped by’ (CP).^40^

In contrast, when GPs and CPs had opportunities to engage in critical reflection and dialogue, this could enable them to communicate information effectively and address clinical issues collaboratively:

There’s [sic] a few other doctors that we do chat on a regular basis to the point where they don’t want us to call them doctors or by their surname. We have got each other’s mobile phones [numbers]. It’s to the point where they can call me on a Sunday night and can ask me for something or do a favour for them. That’s the level it’s gone to (CP).^32^

I got the satisfaction of running my recommendations past the doctor, not just sending him the report, and getting a thank you back … I actually learned a lot from that doctor because he was able to say, ‘that’s not practical’ or ‘that’s a good idea’ (CP).^33^

Role clarity is key to successful C + I models, minimising confusion and ambiguity. One approach involved participation in interprofessional activities (e.g. education, joint service initiatives, case conferencing, quality circles and face-to-face meetings), enabling safe and interactive learning with, from, and about each other:^29,37,42,46^

Personally, I think I have much more of a feel for the type of work the GP does, and what sorts of pressures they have to deal with and how to overcome them (CP).^42^

In line with adult learning theory and contact hypothesis,^47^ such opportunities may engender cultural change, by allowing GPs and CPs to develop knowledge and clarity about their respective roles and capabilities, challenging misconceptions and reducing reciprocal prejudicial attitudes:

It breaks a series of stereotypes that exist from the doctor towards the pharmacist, that there is intrusiveness … and the opposite, from the pharmacist towards the doctors, that they are arrogant … all these things stop when two professionals with similar knowledge, or even a similar age, see each other, a lot of barriers are broken (CP).^13^

We share the same belief in what we’re here for … we’re both part of the total solution for patients … we’re meant to work together (CP).^29^

CP and GP learners may require more and earlier exposure to interprofessional education during their formative years. Professional identities and negative attitudes towards other disciplines can become entrenched over time and act as powerful barriers to effective collaboration.^40,46^ Interprofessional education can help learners internalise the benefits of C + I working and equip them with the interpersonal teamwork skills needed to collaborate in an increasingly complex and resource-constrained health system.^48^

Through opportunities to participate in multi-disciplinary training and joint service initiatives, GPs and CPs became better at collaborating together for the benefit of the patient because they learned how to work together by capitalising on their respective capabilities, knowledge and unique skills.^40,42^ Many GPs and CPs described how collaboration could stimulate mutual and transformative learning:

It’s been hard work, especially at the start, but it gave me an incredible buzz, playing a complementary role to the doctors and the practice nurses, and I would really like to do more of this type of work (CP).^42^

There is obviously a lot more work we can do together … I mean big areas like gastrointestinal disease, well that’s a massive area. I could see him [the pharmacist] interviewing these patients, getting their prescriptions sorted out, telling them what their drugs are for, perhaps even converting them to cheaper therapy (GP).^42^

### Beyond the dyad - supporting patient involvement and engagement

The reviewed literature highlighted the complex triadic relationships between GPs, CPs and patients, and the importance of establishing a strong therapeutic alliance. In contexts where patients found one-to-one interactions with certain health care professionals intimidating, they were more likely to seek health care from another whom they judged to be approachable, because they felt more comfortable to do so.^9,31,49^ Examples included patients valuing the friendly, approachable and non-judgemental manner of the CP: ‘You know your pharmacist, he is more like a friend.’^49^

It was also reported that patients may feel more comfortable opening up to the CP about financial difficulties before disclosing this information to their GP:

They’ll confess to me … ‘I don’t take as much as the doctor said because it’s so expensive.’ I said, ‘Did you tell the doctor?’ ‘No.’ (CP).^43^

For other patients, their preference was to consult the GP, who they viewed as the ‘controller’ of their medical care, with some believing that the CP’s advice needed affirmation by their familiar and trusted GP:

If the pharmacist suggests it to you, then you go to the doctor with it … but I would be reluctant to rely entirely on the pharmacist’s decision that this is different, that this is better, or.^49^

Patients can adapt their stories, or perform different narratives anticipating the needs, interests, or reactions of different audiences (CP and/or GP):

CP: She uses complementary therapies for a few days on and then a few days off … GP: Oh, it’s interesting, because she hasn’t mentioned that to me.^50^

CP: He said that he usually took two in a day [olanzapine 10mg tablets] approximately three times a week. Otherwise, he was only having one 10mg tablet a day … GP: He’d never tell me that!^50^

GPs and CPs are often interested in different aspects of a patient's story; however, there are many patients and stories. When working together, the accumulation of information and knowledge can be used to support patient care. Alongside the potential for patients to feel more comfortable and relaxed with certain health care professionals,^31^ we identified the importance of the geographical accessibility and availability of pharmacies, particularly in areas of high social deprivation.^5,25,31,51^ This sentiment was illustrated by a patient:

Community pharmacies are nearer to my house than the hospital. Pharmacies are widespread, accessible, without appointments and open longer hours.^25^

Similarly, a GP acknowledged the immediate access/convenience that CPs could provide:

Patients don’t need to make an appointment, come in, and wait all that time just for anticoagulation adjustments when the pharmacist can just do it based on the same INR [international normalised ratio] results.^51^

Sometimes patients prefer accessibility, availability, flexibility and/or informality to address a health care need and may be more likely to access CPs because of convenience. Additionally, for patients experiencing difficulties travelling to their GP (due to the cost of travel/limited public transport services), the accessibility of a local CP may provide increased access to health services and support.^5,52^ As reflected by one CP:

We were always going to be a support service to them [GPs], for those people that couldn’t get to them for whatever reason.^5^

Through interaction with patients, CPs could support the identification of unmet health care needs and address them collaboratively with the GP.^52^

However, in some contexts, the physical infrastructure and architecture of pharmacies could inhibit patient engagement, service utilisation, and prevent GP-CP C + I working.^26,31,34,49,53,54^ For instance, if CPs could not provide a suitable ‘safe’ confidential space for consultations, some patients chose not to receive services at the pharmacies, because they valued confidentiality and privacy. For example, when discussing the lack of private rooms for vaccine administration, one CP acknowledged: ‘There could be a privacy thing. It might make people a little uncomfortable … especially children.’^34^ Similarly, a patient expressed their discomfort:

Occasionally I have gone to the supermarket pharmacies ... they’ll start talking openly in a supermarket, over the counter to you about your condition … I don’t want other people listening to what my problems are. (patient).^49^

Adequate infrastructure and private areas may be essential for GPs to feel confident referring patients to pharmacies and patients to feel comfortable receiving services. Some patients perceived pharmacies private consultation rooms as associated with substance misuse services.^31,49^ As one patient succinctly stated: ‘To give drug addicts the privacy to take their methadone.’^49^ This perception could activate stigma and prevent patients from using them and/or seeking help.

Some data showed that in contexts where patients are loyal to their GP, they may be more reluctant to seek health care elsewhere because they are concerned that their GP may view this as a form of betrayal.^49^ Interestingly, however, when patients see that their GPs and CPs have an effective working relationship, they may be more willing, where appropriate, to access health care and receive services with either because they have confidence in GPs-CPs shared care:^34,40,43,49^

I'm going to let the doctor we've chosen, and trusted help, guide us in those decisions. If she says my children should get the vaccine and it doesn't matter if they get it at her office or the pharmacies … I trust her (patient).^34^

What my doctor says to me … If the pharmacist confirms it, then I’m very happy (patient).^43^

GPs and CPs can help patients navigate the available services effectively and equitably. In some instances, patients may not know where to attend, requiring advocacy from a trusted professional. In contexts where patients prefer to see health care professionals who they believe share their cultural, ethnic, religious and/or socioeconomic backgrounds, they may seek such service providers because of homophily.^31,34^ While many general practices provide interpreters, CPs could minimise direct language and cultural barriers to support more effective and equitable patient care:

I must say it’s [collaboration] been really excellent … My context is a largely immigrant population where English is a second language so the time and understanding required to explain the roles of the medications, their side effects, possible interactions …We can’t generate that time for each patient. (GP).^20^

As such, in contexts where CPs could devote time and energy to communicating with patients in a way they could understand, patients were supportive of CPs involvement, because they valued CPs taking time to do this:

Maybe doctors have no time, and that’s why I prefer that pharmacists work collaboratively with doctors so that they would clarify things for patients … it is my first time to talk with the pharmacist, and I’m very glad that they listen to the patient and explain everything simply and plainly (patient).^25^

It was evident that while some patient decision-making was informed by preferences, others were unaware of the breadth of pharmacy services available and, therefore, less likely to make use of these services.^25,31,49^ While increased advertising and national public education campaigns have been supported,^24^ patient use and access to CPs is complex and context dependent. For example, different areas (e.g. urban and rural), pharmacies (e.g. chain or independent) and general practices provide different professional services,^31^ reflecting local needs and service strengths. Patient needs differ, ranging from straightforward to unidentified or complex problems. It may also be difficult for patients to predict in advance the necessary support to identify/diagnose (often multiple or inter-related) problems before being able to consider suitable management options.

### Unanticipated consequences

When CP and GP health care tasks overlap and services are provided in both, patients may experience a lack of continuity of care, due to fragmentation:^3,20,27,34,55^

Many times, my adult patients can get vaccines elsewhere and I don't ever know that it happened … That's when you run into problems. So, when I start talking to them [patients] about it, they'll say they've already had that … That is why documentation is extremely important. It needs to be in the vaccine registry, or we need to be informed somehow (GP).^34^

If you spread things around too much, people tend to miss out or fall in the gaps (GP).^55^

Where services overlap, patients may pick and choose which provider to use. However, this can potentially impact continuity, in addition to creating ambiguity and lack of clarity among GPs and CPs regarding who has overall accountability for patient care:

Follow-up would be a concern of mine ... who is responsible for the next steps of the patients’ care? I’m talking about liability – what if it’s really bad and the pharmacist recommends, they go see a doctor, but they never go and then something bad happens to them, who is liable? Either party could be (GP).^51^

Current UK governance arrangements indicate that the GP has an overall and ongoing responsibility/duty of care for patients. While CP services can be more immediate, their current responsibilities can also be more transient. In contexts where ambiguity exists around accountability, the distribution of clinical services may be more challenging for GPs, CPs and patients to manage. For certain health conditions, a collaborative care model in which patients are first seen and diagnosed by a GP who provides an initial treatment plan may be most effective.^56^

## Discussion

C + I working between GPs and CPs has often been positioned as a transfer of pre-set tasks between two providers. However, this review makes visible the complex and nuanced ways in which the delivery of patient services is negotiated in practice. A conversation with a CP could focus on medication and available options. Whereas a GP-patient interaction might focus on the formulation of problems, range of management options (including medication) and overlaps with social determinants of health.

While role clarity is key, so too is recognition of overlaps and connections between CPs and GPs. Continuity, consistency and knowledge of individual patients/families are often valued by both - helping to identify disruptions to normal patterns which might, for example, indicate a patient is not taking their medication as presumed.^22^ Familiarity and trusting professional relationships between CPs-GPs (pre-existing or evolving) were highly valued as a means to navigate and smooth processes, where appropriate, to support patient management. Identified ways to enable this included frequent, respectful interactions and behaviours, close geographical proximity, regular face-to-face meetings, case conferencing, quality circles and interprofessional education during training to foster a culture of trust and collaboration between professional groups.^27,46^ Challenges in forming professional relationships included temporary staff, lack of integrated communication strategies and geographical separation (such as online pharmacy provision, where informal or ad hoc problem-solving conversations and regular planned meetings were not possible).^22,38^

Organisational leadership, structures and management support are not exclusive triggers to C + I working, but important foundations, minimising a sense of competition between individuals and groups, and maximising opportunities for planning how to deliver complementary and integrative care for patients. Supporting patients requires attention not only to continuity, but also to the complex ways in which clinicians identify or connect problems during clinical reasoning, or work opportunistically with patients at relevant moments to prevent disease or promote health. A system that relies on patients accessing health care from providers independently, without a central place of responsibility and duty of care to patients, is likely to widen health care inequalities for certain patient groups. Effective triadic relationships (patient-GP-CP) are needed to help avert or mitigate ambiguity regarding responsibility, enabling meaningful, ongoing dialogue, engagement and authentic partnerships.

Current technology has elements of integration to support transfer of prescriptions. Communication or clarification beyond that, or shared patient summary records, is less well-established in the UK. While technological solutions are appealing, it is important that any future innovation safely supports C + I patient care where needed, with consent, to maximise agility and flexibility to meet professional and patient’s unique needs. These should avoid compartmentalising patient problems to the extent that they reduce possibilities for patient care, or conflict with individual patient health care needs and professional needs/approaches. Professionals, for example, may record patient interactions differently to reflect their particular clinical priorities. Future IT systems would need to reflect these differences, rather than constraining or standardising approaches to work, and potentially undermining patient safety or elements of existing professional practice.

Technology and other educational innovations need to ensure opportunities for mutual development of learning systems. A spirit of curiosity and respect between individuals and organisations, alongside ongoing relationships and building of trust, between both professional groups and professionals with patients, has been shown in this review to be essential for effective communication, decision-making and C + I working.

### Implications for policy and practice

This review addresses critical knowledge gaps in our understanding of how, when and why patient-CP-GP working arrangements can provide the conditions necessary for effective communication, decision-making and C + I working to maximise equitable patient care. Collectively, our results provide detailed and practical information for policymakers, commissioners, health service managers and professionals in designing and implementing C + I care models that can work for all, rather than just some, groups in society.

Based on the findings of the review and regular PPI/stakeholder engagement workshops, we have developed a series of recommendations where C + I working is needed/appropriate (Table 1). These are tailored to the NHS, but have wider potential generalisability to other health care systems and settings.

**Table 1.** Recommendations for future collaborative and integrated care models.

**How to enable and maximise GP-CP collaboration and integration**
1. Collaboration and integration are maximised when organisations establish a culture that values and promotes collaborative and integrated ways of working (e.g. support may be demonstrated through creating and rewarding spaces for reflection and dialogue, encouraging exploration of differences, diversity of opinion, and organising multi-disciplinary training, joint service initiatives and quality circles)
2. Professional relationships (pre-existing or evolving) between GPs and CPs are essential for collaboration and integration. These relationships can be established through repeated, frequent, respectful interactions and behaviours
3. Established and consistent professional relationships/interactions are important for patient care and enable patient trust and participation in GP-CP collaborative processes
4. Effective triadic relationships are needed between GPs, CPs, and patients. This may comprise meaningful, ongoing dialogue, engagement, and authentic partnership (e.g. participatory and collaborative shared decision making and problem solving on health/social issues). When patients see GPs and CPs collaborating effectively together and in their best interests, they are more likely to engage as active partners in the process
5. Different patients (or patients with different problems) might at times prefer particular spaces or staff. Some patients, for example, prefer open access to a pharmacist, rather than a booked GP appointment. Some like a private room, whereas others associate private rooms with stigma (e.g. unprotected sex or illicit drug use). Adequate and flexible physical infrastructure and space design are therefore needed to nurture cross-disciplinary teamworking, enabling GPs to feel confident referring patients to the CP (or vice versa) and patients to feel comfortable and safe receiving services
6. As services change and GP-CP shared offerings of services evolve, regular and effective communication is required to support patient access and ongoing engagement with CP services. This includes any overlap or distinctions in provision, and/or knowledge to support patients attending with undifferentiated symptoms or problems
**Recommendations for the NHS**
7. IT systems and technologies need to support a movement *away* from rigid and fixed approaches and *towards* more flexible and agile systems, which can support professional practice and ensure that each patient’s unique needs are met
8. Adequate funding must be made available to build strong interpersonal communication and infrastructure. This needs to support regular GP-CP communication and dialogue (formal and informal). CPs should also have an easy to use and reliable means of communicating with GPs (e.g. five-minute discussions at the end of clinic, fast-track access via reception) or accessing elements of patient records

## Limitations

This study has two main limitations. First, our review is limited by the constraints of the current evidence base. Due to the ongoing uncertain and changing health care landscape, it is possible that empirical work lags behind practice changes. New information on GP-CP C + I working will continue to be published; however, this timely review will support future responses to new ways of working, following, for example, the NHS Long-Term Plan and integrated care systems.

Second, while many of our findings are related to the provision of equitable/effective patient care, few identified documents looked explicitly at the impact of CP-GP on equitable provision and access of care for patients. This is an area ripe for further research.

## Conclusion

Our research generates new insights regarding how and in which circumstances CP-GP collaboration and integration may work. Although GP-CP C + I working has often been positioned as transfer of pre-set tasks between the two providers, our findings demonstrate the dynamic and highly complex nature of patient-GP-CP interactions, relationships and delivery of services. Our review findings can be used to inform future policy, research and practice regarding how to best design and deliver C + I care when appropriate. Future research is needed to empirically test our findings and refined PT in other policy contexts and settings.

## Supplemental Material

**Supplemental Material -** Collaborative and integrated working between general practice and community pharmacies: A realist review of what works, for whom, and in which contexts Supplemental Material for Collaborative and integrated working between general practice and community pharmacies: A realist review of what works, for whom, and in which contexts by Emily Owen-Boukra, Ziyue Cai, Claire Duddy, Nina Fudge, Julia Hamer-Hunt, Fran Husson, Kamal R Mahtani, Margaret Ogden, Deborah Swinglehurst, Malcolm Turner, Cate Whittlesea, Geoff Wong and Sophie Park in Journal of Health Services Research & Policy.
