# Supplementary material for: Collaborative and integrated working between general practice and community pharmacies: A realist review of what works, for whom, and in which contexts
Source: J Health Serv Res Policy. 2024 Oct 23;30(2):136–48. doi: 10.1177/13558196241290923 (PMC11877979; doi:10.1177/13558196241290923)
Supplement: Supplemental Material - Collaborative and integrated working between general practice and community pharmacies: A realist review of what works, for whom, and in which contexts [file sj-pdf-1-hsr-10.1177_13558196241290923.pdf]

Journal of Health Services Research & Policy
Collaborative and integrated working between general practice and community pharmacies: A realist review of what works, for whom, and in which contexts
Owen-Boukra E, et al

Online Supplement

S1: Complete search strategy used in each database.

S2: Inclusion and exclusion criteria.

S3: Refined programme theory diagram.

S4: CMOCs and supporting evidence.

S5: Included document characteristics.

Journal of Health Services Research & Policy
Collaborative and integrated working between general practice and community pharmacies: A realist review of what works, for whom, and in which contexts
Owen-Boukra E, et al

S1: Complete search strategy used in each database.

Details of the search developed for MEDLINE:

**Data Source**	Database	MEDLINE
	Details	Medline (Ovid MEDLINE® Epub Ahead of Print, In-Process & Other Non-Indexed Citations, Ovid MEDLINE® Daily and Ovid MEDLINE®) 1946 to present
	Host	Ovid
	Date searched	18/04/2022
	Database Update	Daily update
**Limiters**	English only?	Yes
	Time period searched	2000 to current
	Publications types	No limit
	Other	
**Results**	Items found	609
	Internal duplicates (within one database)	0
	(between databases)	0
	New	609

In Ovid, save your search. Go to **Saved Searches** and click on the eye icon of your SR saved search to **View** your search. Copy and paste your search into **Notepad;** put tabs between the line number and text. Copy and paste into cell **B15**.

	**Paste text of search strategy below**	
**CompanionTest Version E MEDLINE**		
Medline (Ovid MEDLINE® Epub Ahead of Print, In-Process & Other Non-Indexed Citations, Ovid MEDLINE® Daily and Ovid MEDLINE®) 1946 to present		
		10064
1	((communit* or local) adj2 pharmac*).ti,ab,kw.	5409
2	Community Pharmacy Services/	11999
3	1 or 2	249834
4	(general practi* or family practi* or family physician* or primary health* or primary care).ti,ab,kw.	106846
5	exp *Primary Health Care/	50899
6	exp *General Practice/	22059
7	*general practitioners/ or *physicians, family/ or *physicians, primary care/	20715
8	*Ambulatory Care/	348227
9	or/4-8	158988
10	((integrat* or collab* or cooperat* or co-operat* or coordinat* or co-ordinat* or interprofessional* or inter-professional* or multidisciplin* or multi-	4322
11	((joint or joined-up or joined up or relations*) adj1 work*).ti,ab,kw.	14362
12	((organi?ation* or delivery or care) adj1 model*).ti,ab,kw.	267499
13	(infrastructure* or interface*).ti,ab,kw.	65859
14	*Attitude of Health Personnel/	18726
15	*Cooperative Behavior/	29750
16	exp *Interprofessional Relations/	46346
17	exp *Professional Role/	10524
18	exp *"Delivery of Health Care, Integrated"/	6488
19	*models, organizational/	583426
20	or/10-19	43231
21	9 and 20	15886
22	Primary Health Care/og [Organization & Administration]	54656
23	21 or 22	669
24	3 and 23	609
25	limit 24 to (english language and yr="2000 -Current")	

Details of the search developed for PsycINFO:

**Data Source**	Database	PsycINFO	
	Details	PsycINFO 1806 to present	
	Host	Ovid	
	Date searched	18/04/2022	
	Database Update	Weekly update	
**Limiters**	English only?	Yes	
	Time period searched	2000 to current	
	Publications types	No limit	
	Other		
**Results**	Items found	65	
	Internal duplicates (within one database)	0	
	External duplicates (between databases)	49	
	New	16	
	**Paste text of search strategy below**		
	PsycINFO 1806 to present		
	1	((communit* or local) adj2 pharmac*).ti,ab.	1218
	2	(general practi* or family practi* or family physician* or primary health* or primary care).ti,ab.	50490
	3	*primary health care/	16288
	4	*family medicine/	1039
	5	*general practitioners/ or family physicians/	5671
	6	or/2-5	53935
	7	((integrat* or collab* or cooperat* or co-operat* or coordinat* or co-ordinat* or interprofessional* or inter-professional* or multidiscipli	93067
	8	((joint or joined-up or joined up or relations*) adj1 work*).ti,ab.	5105
	9	((organi?ation* or delivery or care) adj1 model*).ti,ab.	5793
	10	(infrastructure* or interface*).ti,ab.	32850
	11	exp *health personnel attitudes/	21629
	12	*cooperation/ or *collaboration/ or *cooperative learning/ or *teamwork/	23429
	13	*professional role/ or *role expectations/ or *professional identity/ or *professionalism/ or *role perception/ or *role satisfaction/	11252
	14	exp *organizational structure/ or exp *organizational behavior/ or *organizational climate/ or *organizational development/ or *self-ma	63148
	15	or/7-14	237261
	16	1 and 6 and 15	72
	17	limit 16 to (english language and yr="2000 -Current")	65

Journal of Health Services Research & Policy
Collaborative and integrated working between general practice and community pharmacies: A realist review of what works, for whom, and in which contexts
Owen-Boukra E, et al

Details of the search developed for Embase:

**Data Source**	Database	Embase
	Details	Embase 1974 to present
	Host	Ovid
	Date searched	18/04/2022
	Database Update	Daily update
**Limiters**	English only?	Yes
	Time period searched	2000 to current
	Publications types	No limit
	Other	
**Results**	Items found	725
	Internal duplicates (within one database)	11
	External duplicates (between databases)	336
	New	378

In Ovid, save your search. Go to **Saved Searches** and click on the eye icon of your SR saved search to **View** your search. Copy and paste your search into **Notepad;** put tabs between the line number and text. Copy and paste into cell **B15**.

Paste text of search strategy below

Companion FINAL Embase

Embase 1974 to present

1	((communit* or local) adj2 pharmac*).ti,ab,kw.	19044
2	pharmacy (shop)/	7783
3	1 or 2	24320
4	(general practi* or family practi* or family physician* or primary health* or primary care).ti,ab,kw.	320535
5	exp *primary health care/	66969
6	*general practice/	38520
7	*general practitioner/	26190
8	*ambulatory care/	13392
9	or/4-8	362434
10	((integrat* or collab* or cooperat* or co-operat* or coordinat* or co-ordinat* or interprofessional* or inter-professional* or multidisciplin* or multi-discipli	210824
11	((joint or joined-up or joined up or relations*) adj1 work*).ti,ab,kw.	5802
12	((organi?ation* or delivery or care) adj1 model*).ti,ab,kw.	19594
13	(infrastructure* or interface*).ti,ab,kw.	287322
14	exp *health personnel attitude/	89746
15	exp *cooperation/	18502
16	exp *organizational structure/	1472
17	*multidisciplinary team/ or *collaborative care team/	2390
18	or/10-17	612435
19	3 and 9 and 18	778
20	limit 19 to (english language and yr="2000 -Current")	725

Details of the search developed for CINAHL:

**Data Source**	Database	CINAHL	
	Details		
	Vendor	Ebsco	
	Date searched	18/04/2022	
	Database Update	Unknown	
**Limiters**	English only?	Yes	
	Time period searched	2000 to current	
	Publications types	No limit	
	Other		
**Results**	Items found	421	
	Internal duplicates (within one database)	11	
	External duplicates (between databases)	279	
	New	131	
In EBSCOhost, click on Search History/Alerts. Copy the lines from the search that represent your full search and paste them into Notepad. Put tabs between the line number and text. Delete extraneous text, such as the text from the Actions column. You will need to include Limiters which appear to the right of the search string. Copy and paste the edited version into cell B15.	**Paste text of search strategy below**		
	**Companion FINAL CINAHL**		
	S1	TI (((communit* or local) N2 pharmac*)) OR AB (((communit* or local) N2 pharmac*))	5664
	S2	(MH "Pharmacy, Retail")	7758
	S3	S1 OR S2	10,757
	S4	TI ("general practi*" OR "family practi*" OR "family physician*" OR "primary health*" OR "primary care")) OR AB ("general prac	124,957
	S5	(MM "Primary Health Care")	43055
	S6	(MM "Family Practice")	17037
	S7	(MM "Physicians, Family")	12503
	S8	(MM "Ambulatory Care")	7066
	S9	S4 OR S5 OR S6 OR S7 OR S8	151957
	S10	TI ((integrat* OR collab* OR cooperat* OR co-operat* OR coordinat* OR co-ordinat* OR interprofessional* OR inter-professiona	98652
	S11	TI ((joint OR joined-up OR "joined up" OR relations*) N1 work*)) OR AB ((joint OR joined-up OR "joined up" OR relations*) N1	5602
	S12	TI ((organi?ation* OR delivery OR care) N1 model*)) OR AB ((organi?ation* OR delivery OR care) N1 model*))	19037
	S13	TI ((infrastructure* OR interface*)) OR AB ((infrastructure* OR interface*))	33778
	S14	(MM "Attitude of Health Personnel+")	65362
	S15	(MM "Cooperative Behavior")	3453
	S16	(MM "Interprofessional Relations")	11055
	S17	(MM "Professional Role+")	51391
	S18	(MM "Health Care Delivery, Integrated")	8793
	S19	(MM "Organizational Structure") OR (MM "Organizational Culture")	9932
	S20	S10 OR S11 OR S12 OR S13 OR S14 OR S15 OR S16 OR S17 OR S18 OR S19	276,394
	S21	S3 AND S9 AND S20; Limiters - Published Date: 20000101-; English Language	421
	S22		

Details of the search developed for HMIC:

**Data Source**	Database	HMIC	
	Details		
	Vendor	Ovid	
	Date searched	19/04/2022	
	Database Update	Unknown	
**Limiters**	English only?	Yes	
	Time period searched	2000 to current	
	Publications types	No limit	
	Other		
**Results**	Items found	162	
	Internal duplicates (within one database)	15	
	External duplicates (between databases)	42	
	New	105	
	**Paste text of search strategy below**		
	HMIC Health Management Information Consortium <1979 to March 2022>		
	1	((communit* or local) adj2 pharmac*).ti,ab.	1,376
	2	community pharmacy/ or community pharmacies/ or community pharmacists/	1,233
	3	1 or 2	1,822
	4	(general practi* or family practi* or family physician* or primary health* or primary care).ti,ab.	31,971
	5	exp primary care/ or primary care teams/ or primary care trusts/	26,978
	6	general practice/ or general practice management/ or general practices/ or general practitioners/	17,912
	7	ambulatory care/ or ambulatory care services/	288
	8	or/4-7	45,681
	9	((integrat* or collab* or cooperat* or co-operat* or coordinat* or co-ordinat* or interprofessional* or inter-professional* or	11499
	10	((joint or joined-up or joined up or relations*) adj1 work*).ti,ab.	1,570
	11	((organi?ation* or delivery or care) adj1 model*).ti,ab.	918
	12	(infrastructure* or interface*).ti,ab.	2480
	13	exp attitudes/	22249
	14	exp interprofessional relations/	2550
	15	professional role/ or professional responsibility/	3422
	16	integrated care/ or collaborative care/	2902
	17	exp organisational structure/ or organisational infrastructure/	1523
	18	or/9-17	43145
	19	3 and 8 and 18	193
	20	limit 19 to (yr="2000 -Current" and english)	162

Details of the search developed for WoS:

**Data Source**	Database	Web of Science Core Collection	
	Details	SCIE, SSCI, ESCI, CPCI; 1900 to present	
	Vendor	Clarivate	
	Date searched	19/04/2022	
	Database Update	Daily	
**Limiters**	English only?	Yes	
	Time period searched	2000 to current	
	Publications types	No limit	
	Other		
**Results**	Items found	431	
	Internal duplicates (within one database)	1	
	External duplicates (between databases)	316	
	New	114	
	**Paste text of search strategy below**		
	**Not saved**		
	1	((communit* or local) NEAR/2 pharmac*) (Topic)	12714
	2	("general practi*" or "family practi*" or "family physician*" or "primary health*" or "primary care") (Topic)	256,610
	3	(integrat* or collab* or cooperat* or co-operat* or coordinat* or co-ordinat* or interprofessional* or inter-professional* or multidisciplin* or multi-	479,533
	4	((joint or joined-up or "joined up" or relations*) NEAR/1 work*) (Topic)	21,438
	5	((organi?ation* or delivery or care) NEAR/1 model*) (Topic)	36,019
	6	(infrastructure* or interface*) (Topic)	1,297,298
	7	#3 or #4 or #5 or #6	1,798,602
	8	#1 and #2 and #7	463
	9	#7 AND #2 AND #1 and 2022 or 2021 or 2020 or 2019 or 2018 or 2017 or 2016 or 2015 or 2014 or 2013 or 2012 or 2011 or 2010 or 2009	457
	10	#7 AND #2 AND #1 and 2022 or 2021 or 2020 or 2019 or 2018 or 2017 or 2016 or 2015 or 2014 or 2013 or 2012 or 2011 or 2010 or 2009	431

Journal of Health Services Research & Policy
Collaborative and integrated working between general practice and community pharmacies: A realist review of what works, for whom, and in which contexts
Owen-Boukra E, et al

Details of the search developed for IBSS:

**Data Source**	Database	IBSS	
	Details	International Bibliography of Social Sciences	
	Vendor	Proquest	
	Date searched	01/12/21	
	Database Update	Unknown	
**Limiters**	English only?	Yes	
	Time period searched	2000 to current	
	Publications types	No limit	
	Other		
**Results**	Items found	4	
	Internal duplicates (within one database)	0	
	External duplicates (between databases)	2	
	New	2	
	**Paste text of search strategy below**		
	**Not saved**		
	S1	noft((communit* OR local) N/2 pharmac*)	294
	S2	noft("general practi*" or "family practi*" or "family physician*" or "primary health*" or "primary care")	10,038
	S3	noft((integrat* or collab* or cooperat* or co-operat* or coordinat* or co-ordinat* or interprofessional* or inter-professional* or multidisciplin* or m	42,247
	S4	noft((joint or joined-up or "joined up" or relations*) N/1 work*)	7,350
	S5	noft((organi?ation* or delivery or care) N/1 model*)	4,231
	S6	noft(infrastructure* or interface*)	58,203
	S7	S3 OR S4 OR S5 OR S6	109,374
	S8	S1 AND S2 AND S7	4
	S9	Applied filters	4

Details of the search developed for Sociology Collection:

**Data Source**	Database	Sociology Collection	
	Details	Applied Social Sciences Index & Abstracts (ASSIA), Sociological Abstracts and Sociology Database	
	Vendor	Proquest	
	Date searched	19/04/2022	
	Database Update	Unknown	
**Limiters**	English only?	Yes	
	Time period searched	2000 to current	
	Publications types	No limit	
	Other		
**Results**	Items found	38	
	Internal duplicates (within one database)	7	
	External duplicates (between databases)	27	
	New	4	
	**Paste text of search strategy below**		
	**Not saved**		
	S1	noft((communit* OR local) N/2 pharmac*)	899
	S2	noft("general practi*" or "family practi*" or "family physician*" or "primary health*" or "primary care")	50,193
	S3	noft((integrat* or collab* or cooperat* or co-operat* or coordinat* or co-ordinat* or interprofessional* or inter-professional* or multidisciplin* o	67,741
	S4	noft((joint or joined-up or "joined up" or relations*) N/1 work*)	20,934
	S5	noft((organi?ation* or delivery or care) N/1 model*)	10,050
	S6	noft(infrastructure* or interface*)	26,952
	S7	S3 OR S4 OR S5 OR S6	120,384
	S8	S1 AND S2 AND S7	43
	S9	Applied filters	38

Details of the search developed for King's Fund Library:

**Data Source**	Database	King's Fund Library Database	
	Details	https://koha.kingsfund.org.uk/; 1863 to present	
	Vendor	King's Fund	
	Date searched	19/04/2022	
	Database Update	Unknown	
**Limiters**	English only?	Yes	
	Time period searched	2000 to current	
	Publications types	No limit	
	Other		
**Results**	Items found	100	
	Internal duplicates (within one database)	2	
	External duplicates (between databases)	35	
	New	63	
	**Paste text of search strategy below**		
	**Not saved**		
	1	Keyword: community pharmacy AND Keyword: general practice	103
		Limited to 2000 -	100

DB Results Table:

Data Source				Limits				Duplicates Results			
**Database**	**Vendor**	**Date searched**	**Database update**	**English only?**	**Time Period Searched**	**Publication types**	**Other Limits**	**Items found**	**Inner dups**	**Ext dups**	**New**
MEDLINE	Ovid	18/04/2022	Daily update	Yes	2000 to current	No limit	-	609	0	0	609
Embase	Ovid	18/04/2022	Daily update	Yes	2000 to current	No limit	-	725	11	336	378
PsycINFO	Ovid	18/04/2022	Weekly update	Yes	2000 to current	No limit	-	65	0	49	16
CINAHL	Ebsco	18/04/2022	Unknown	Yes	2000 to current	No limit	-	421	11	279	131
HMIC	Ovid	19/04/2022	Unknown	Yes	2000 to current	No limit	-	162	15	42	105
Web of Science Core Collection	Clarivate	19/04/2022	Unknown	Yes	2000 to current	No limit	-	431	1	316	114
IBSS	Proquest	19/04/2022	Unknown	Yes	2000 to current	No limit	-	4	0	2	2
Sociology Collection	Proquest	19/04/2022	Unknown	Yes	2000 to current	No limit	-	38	7	27	4
King's Fund Library Database	King's Fund / Koha	19/04/2022	Unknown	Yes	2000 to current	No limit	-	100	2	35	63
							**Totals**	**2555**	**47**	**1086**	**1422**

Total duplicates removed **1133**

S2: Inclusion and exclusion criteria.

Date	2000 onwards (to capture literature prior to the first collaborative and integrated initiative called 'MUR' in 2003)
Setting	General practice, community pharmacy
Country	Our initial focus was on the UK, but we did draw on data from other healthcare systems relevant to the UK
Focus	A focus on relevant elements of the working relationship between GPs and CPs (to include terms such as collaborative and integrated working, but not exclusively)
Exclusion	We excluded papers focused entirely on clinical pharmacists working within general practice settings (our aim was to explore collaborative and integrated working across organisations and sites), and documents that did not provide any rich or detailed information contributing to programme theory refinement

S3: Refined programme theory diagram.

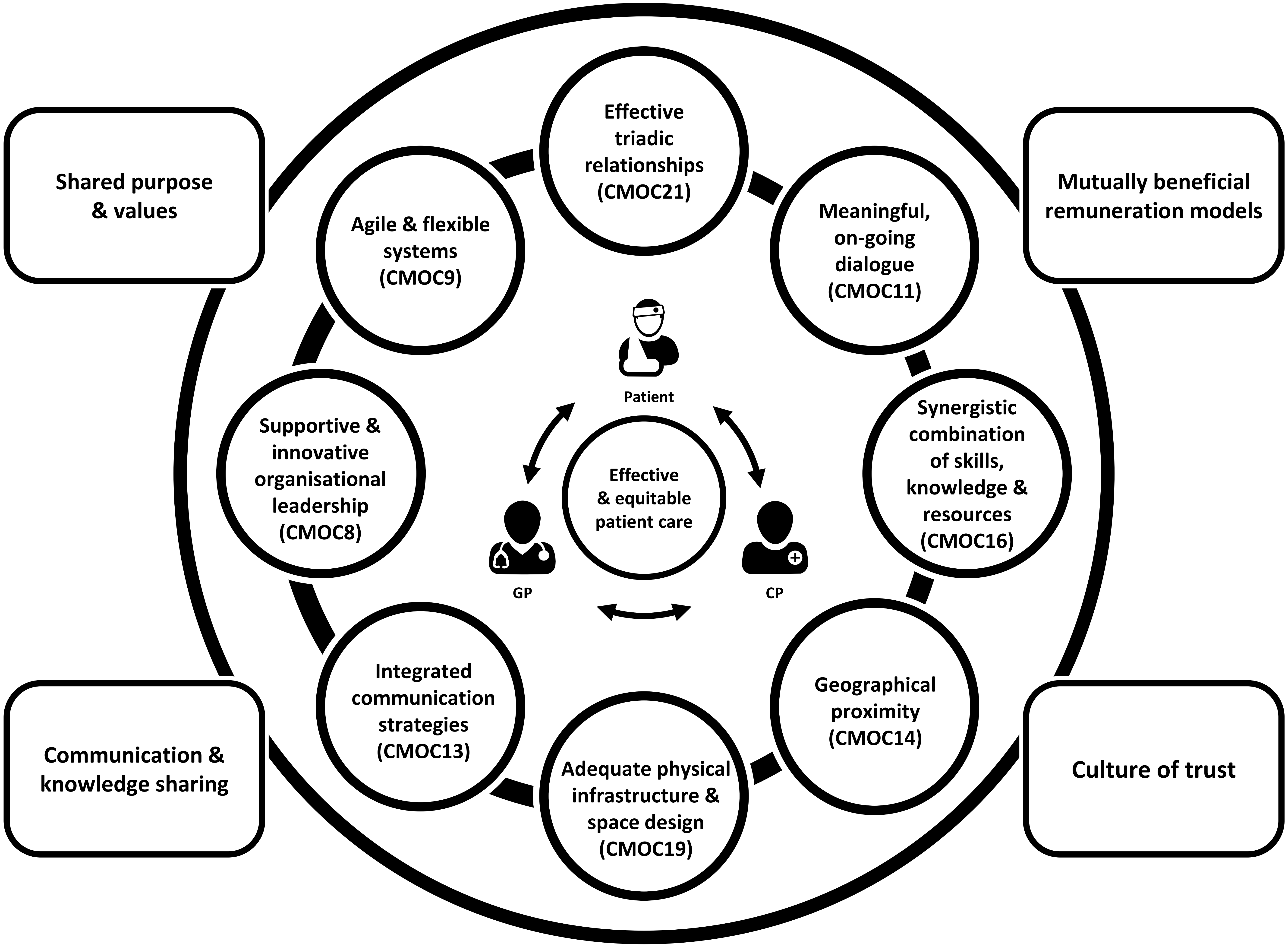

S4: CMOCs and supporting evidence.

CP = Community Pharmacists, GP = General Practitioners

Historical norms

1. Scope of practice

When CPs are asked to make clinical decisions that they are less familiar with or knowledgeable about (C), they tend to feel reluctant to make such decisions (O) because they perceive it is beyond their scope of practice and lack the confidence to do so (M).

“The perception of inadequacy by community pharmacists appears to be a major barrier, with less than 50% feeling adequate dealing with general practitioners on clinical medicine-related issues, having sufficient confidence in their clinical knowledge and feeling sufficiently trained.” – Bryant et al., 2009.

“I probably need to up-skill to be able to be more confident and say, ‘okay this is the research that I am talking about’, not just my gut feeling. [P9]” – Bryant et al., 2010.

“I don’t have the confidence.” [P10] – Bryant et al., 2010.

“Pharmacists were open to expanding their role to include services such as medicines management, monitoring, screening, advisory and prescribing roles, but expressed concern on how this would be implemented and identified a lack of confidence in their clinical knowledge and training. Only about half considered they have adequate skills to undertake clinical roles. GPs demonstrated less agreement for all roles. While accepting of the traditional dispensing role, they were less supportive of other roles, although there was a gradual shift towards agreement concerning pharmacists’ involvement in medicines management.” – Bryant et al., 2017.

“Investigation of the culture of the pharmacy profession has revealed characteristics including lack of confidence, aversion to change, and apparent unwillingness to leave the comfort of the dispensary. This may be impeding the profession’s advancement; attitudes held by pharmacists may be sabotaging the development of the profession. - Bryant et al., 2017.”

“Pharmacist-perceived barriers to expansion of their roles include a lack of orientation to take on new roles, and perceptions that some roles are not legitimate for pharmacists. Other pharmacists perceive they lack knowledge and clinical problem-solving skills, so feel uncomfortable with the accountability that is integral to these services.” – Bryant et al., 2017.

“Only a few were interested in providing vaccines and while there were some interviewees who were keen to do or who were already engaged in continuing education to become pharmacist prescribers, they did not see this as a core part of their professional roles: We’re not doctors and whilst we can interpret things, I think a lot of that should be left to doctors. … basically, managing people’s

medicine, I think is far more important than being able to prescribe. I think, do that, and do it well and prescribing on the more basic things. (Pharmacist).” – Bidwell & Thompson, 2015.

“Some members of both professional groups raised concerns about where the limits of their professional jurisdiction lay. GPs tended to be supportive of pharmacist involvement in the management of patients who were taking multiple medications. There was much more limited support for other non-medicines related services, including among pharmacists themselves.” – Bidwell & Thompson, 2015.

“Many pharmacists were reluctant to explore areas outside their traditional scope of practice and invest considerable financial and personnel resources for an uncertain return.” – Bidwell & Thompson, 2015.

“A further reason why pharmacists may not be used to having conversations with patients around their AR is that they may perceive that their patients may not need to speak to them. A recent study found that patients feel confident in self-managing their AR and they do not see the need to speak to the pharmacist [17], propagating the pharmacists reduced confidence in communication.” - Cvetkovski et al., 2020.

“Although, at one site offering out-of-hours care, there was evidence that the pharmacists involved were taking longer to complete work than those for whom they were substituting. This was attributed to a lack of confidence and the situation was anticipated to improve with time: …you can feel that the majority don’t have that confidence yet . . . they don’t turn over work as quick as say a GP, but [the GPs have] been doing this for ten years and they’re used to how out of hours works… I’m sure another one or two years down the line they’ll just swing along with the rest of the nurse practitioners and doctors. (Site A, LPS Provider).” – Bradley et al., 2008.

“In addition to the lack of interprofessional skills, individual pharmacists have often had a lack of knowledge in particular topics and lack of individual confidence, which affects the variety of service provision to the patients. A shortcoming in the graduate's competence themselves could also be one of the causes of the lack of pharmacists’ competency. Other obstacles from the individual pharmacists relate to the poor motivation from the pharmacist to be more involved in patient care, to adopt new roles and to participate in Continuing Professional Development (CPD) program.” - Hermansyah et al., 2020.

“While, at a political level, it may seem straightforward and even common-sense to argue that a range of practitioners should be working collaboratively and that they should be working at the top of their respective scopes of practice, how this is operationalised is less clear-cut. Not the least of the complexity surrounding this issue is the respective groups’ differential positioning within the healthcare arena, both in terms of power and employment structure.” – Bidwell & Thompson, 2015.

2. Practice boundaries

When CPs are asked to make decisions that they perceive are usually undertaken by other health care professionals (C), they may be reluctant to do so (O) because of concerns about conflict (M).

"They believe that pharmacists see themselves as dispensers of the general practitioner's prescriptions and wish to avoid conflict with general practitioners over clinical decisions. They feel unworthy to challenge the status quo, despite a perception that they are knowledgeable about drug therapy." – Bryant et al., 2010.

"Pharmacist-perceived barriers to expansion of their roles include a lack of orientation to take on new roles, and perceptions that some roles are not legitimate for pharmacists. Other pharmacists perceive they lack knowledge and clinical problem-solving skills, so feel uncomfortable with the accountability that is integral to these services." – Bryant et al., 2017.

"GPs' perceptions of pharmacists may determine their willingness to collaborate. Historically, the 'shop keeping' role of community pharmacists has resulted in their being perceived as tainted health professionals, who might act for commercial gain and not in the patients' best interests." – Bryant et al., 2017.

"A significant challenge reported by pharmacists was the absence of collaboration and inconsistency in the messages delivered by doctors working within a shared pharmacy and medical centre premises. Pharmacists found it difficult to challenge the appropriateness of doctors' recommendations when they felt it was inappropriate. For example, they reported that many people with AR (Allergic Rhinitis) came from the doctor with a doctor's note or a prescription for an OAH. Upon review of the patient, pharmacists determined that this was not necessarily an optimal treatment for the patient, however they felt it was not appropriate to challenge the general practitioner's recommendations." – Cvetkovski et al., 2020.

"We can't change the perspective if the doctors are writing script for [OAH], if I say, no you just take intranasal corticosteroid, that's not good." PH#8 - Cvetkovski et al., 2020.

"Sources of tension include pharmacy's 'shopkeeper' image, traditional medical hierarchies, and potential encroachment on professional boundaries." – Bradley et al., 2018.

"Historically their relationship has been described as 'uneasy' (Cowen 1992), due to an apparent overlap in both service and skill, resulting in conflict and competition between the two professions (Turner 1999). Power also comes into play, with the pharmacist often perceived as subordinate to the doctor in the hierarchy of medical care (Cooper et al. 2009). This subordination is said to have arisen due to educational differences, with medicine deemed an academic discipline and pharmacy considered a craft (Helmstaedter and Staiger 2002) and is continuously reinforced through the enduring image of the community pharmacist as 'the shopkeeper' (Hughes and McCann 2003). From a profession trait theory approach, this shopkeeper image, inherently at odds with altruism, along with a failure by pharmacists to gain control over the social object of their practice – the drug – led to pharmacy being an incomplete profession (Denzin and Metlin 1968)." – Bradley et al., 2018.

“Pharmacists were mindful of professional boundaries with GPs in recommending changes to older patients’ existing prescriptions (‘Social/professional role and identity’), and some doubted their ability to have recommended medication changes implemented by GPs (‘Beliefs about capabilities’).” – Cadogan et al., 2015.

“Despite wishing to pursue more patient-focussed roles, many pharmacists did not want to encroach on the general practitioner’s territory, generally seeing their role as reducing the general practitioner’s workload (delegatory roles) and helping with adherence problems and patients’ management of medicines rather than more clinical roles.” – Bryant et al., 2010.

“They described conflicting views about the management of long-term medical conditions by pharmacists. Basically, as perceived by the pharmacists, there remained an entrenched division between general practitioners as prescribers and pharmacists as dispensers.” – Bryant et al., 2010.

“One pharmacist about informing general practitioners: ‘‘previous experience with doctors made me decide not to inform them. They do not want pharmacist’s pharmaceutical care projects. They believe that we are trying to take their place. If necessary, I take their contact details because then I can call them about a specific problem of a certain patient.” – Adhien et al., 2013.

Other obstacles from the individual pharmacists relate to the poor motivation from the pharmacist to be more involved in-patient care, to adopt new roles and to participate in Continuing Professional Development (CPD) program.” - Hermansyah et al., 2020.

“As revealed by the focus groups discussions, some GPs found it inappropriate that pharmacists can initiate an NMS without a physician’s approval and suggested that the NMS should be prescribed and that feedback from the pharmacist should be added to the patient’s file (if applicable).” – Fraeyman et al., 2016.

“You’re stepping into a more clinical report, and we have to … try and straddle that without stepping on too many toes. The service is still very new and there are some GPs that are very welcoming of the service and there are some that appear to be feeling threatened by it or haven’t perhaps have got as good a working relationship with the pharmacies and so they, for whatever reason, are not as open to receiving feedback. (Pharmacist).” – Bidwell & Thompson, 2015.

“While, at a political level, it may seem straightforward and even common-sense to argue that a range of practitioners should be working collaboratively and that they should be working at the top of their respective scopes of practice, how this is operationalised is less clear-cut. Not the least of the complexity surrounding this issue is the respective groups’ differential positioning within the healthcare arena, both in terms of power and employment structure.” – Bidwell & Thompson, 2015.

“The very need to argue that all professionals should be working at the top of their scopes of practice implies that some degree of role change is needed. Any discussion of role change raises questions about potential conflict between different groups of professionals. While Adams is at pains to point out that it should not be assumed that interprofessional conflict will occur, she does argue that

jurisdictional conflict is more likely when "occupational groups are less evenly matched in terms of power, status, and organisation". – Bidwell & Thompson, 2015.

"Pharmacists reported a more complicated relationship with GPs. While most of them had built up a good rapport with the GPs whose prescriptions they most often dispensed, they treated the relationship cautiously and seldom appeared to feel on an equal professional footing." – Bidwell & Thompson, 2015.

"Only a few were interested in providing vaccines and while there were some interviewees who were keen to do or who were already engaged in continuing education to become pharmacist prescribers, they did not see this as a core part of their professional roles: We're not doctors and whilst we can interpret things, I think a lot of that should be left to doctors. … basically, managing people's medicine, I think is far more important than being able to prescribe. I think, do that, and do it well and prescribing on the more basic things. (Pharmacist)." – Bidwell & Thompson, 2015.

"Some members of both professional groups raised concerns about where the limits of their professional jurisdiction lay. GPs tended to be supportive of pharmacist involvement in the management of patients who were taking multiple medications. There was much more limited support for other non-medicines related services, including among pharmacists themselves." – Bidwell & Thompson, 2015.

"Many pharmacists were reluctant to explore areas outside their traditional scope of practice and invest considerable financial and personnel resources for an uncertain return." – Bidwell & Thompson, 2015.

"The encounter itself can be viewed as a constant source of potential conflict with the majority of queries instigated by pharmacists and relating to omissions on prescriptions, dosage queries, or potential medication interactions, all of which hold connotations of fault or error on the part of the GP. Understandably, a primary concern for CPs was to try to ensure that these encounters went smoothly and without conflict. CPs conceptualised some aspects of their interaction with GPs as a type of 'game' and spoke of the need to adopt a particular 'approach', to avoid damaging their relationship or potential relationship." – Bradley et al., 2018.

"Overlapping responsibilities generated a negative opinion of the other professional. GPs and CPs believed that sometimes the other professional was performing tasks that should not be done by him/her. This concern was generated by the fact that the roles of professionals were not well defined." – Rubio-Valera et al., 2012.

"We have the experience of seeing a productive cough, a dry cough, some mucosity. I think we can recommend a medication… a doctor will tell you: "but well, we are doctors, and it is us who have to (prescribe)" and, well, he/she is right, on the one hand they're right, but where is the line where we end and the doctor begins, you know? [CP7: CP WO BCN]." – Rubio-Valera et al., 2012.

"Second, culture and behavioural issues in primary care around the role and identity of pharmacists, significantly slowing the mobilisation of the profession into healthcare models." – Brewster et al., 2020.

"GPs' lack of knowledge regarding pharmacists' professional training, responsibilities and continuing professional development obligations, and their professional skills and strengths, has resulted in contributions made by pharmacists to patient-focused services being undervalued. GPs have also expressed concerns about pharmacists taking on GP roles such as screening, monitoring, and prescribing." – Bryant et al., 2017.

"In part, the issue is that pharmacists' roles appear ill-defined and on the periphery of primary healthcare teams, so pharmacists have been viewed as subordinates to GPs who consider themselves the decision-makers and ultimately responsible for patient outcomes." – Bryant et al., 2017.

"Some doctors assume that I only call for business, or for something not available, not for the patient. So, when I make a recommendation, some doctors feel I want to take his job I want to make overlay of his rule… physician feels threatened so when I talk with them, to ease the conversation, I would say: I know you know more than me." (CP) – El-Awaisi et al., 2018.

"Moreover, participants reported that some physicians were threatened by the increasing therapeutic role of the pharmacist and preferred the traditional way of practice." – El-Awaisi et al., 2018.

3. Culture of mistrust

When GPs or CPs perceive that the other is mainly motivated by financial incentives/prosperity (C), they are more reluctant to collaborate (O) because they do not believe that this motive is in the best interest of the patient (M).

"Trust was reported to be an essential for GP–pharmacist interactions (i.e. trust that the pharmacist will act in the best interest of the patient was as important as trust in the pharmacist's clinical ability). However, several GPs questioned the role of the pharmacist as a shopkeeper and believed not all pharmacists acted in the best interest of the patient: 'Some pharmacists don't do things for the best interest of the patient. They are just a business out to make profit.' (GP09) In some cases, this perception of pharmacists as shopkeepers resulted in a lack of respect for them as healthcare professionals and a lack of trust in their clinical ability: '[there needs to be] respect for the fact that we can consider drug therapy a bit more than [what GPs] think' (P08)" - Van et al., 2011.

"One pharmacist was sceptical as to whether GPs wanted to work with him at all. 'There are doctors who still have the attitude that they're the doctor and no one can possibly question them… that attitude makes it impossible to collaborate' (P04)" - Van et al., 2011.

"GPs' perceptions of pharmacists may determine their willingness to collaborate. Historically, the 'shop keeping' role of community pharmacists has resulted in their being perceived as tainted health professionals, who might act for commercial gain and not in the patients' best interests." – Bryant et al., 2017.

"Consumers perceived that most pharmacists operated in a transactional mode, and it was apparent that existing practices within pharmacy did not have consumers' trust." - Almansour et al., 2020.

"Participants appeared to not fully trust pharmacists, particularly community pharmacists. Most participants indicated only usually seeing community pharmacists in a transactional role, given that most pharmacies operate in a business model. Further, participants in general did not feel that their community pharmacists had high level clinical skills." – Almansour et al., 2020.

''I don't trust community pharmacists, to be honest. Hospital pharmacists work under the supervision of the Ministry of Health while community pharmacists are unsupervised, and they usually look for financial profits." – Almansour et al., 2020.

"As revealed by the focus groups discussions, some GPs found it inappropriate that pharmacists can initiate an NMS without a physician's approval and suggested that the NMS should be prescribed and that feedback from the pharmacist should be added to the patient's file (if applicable)." – Fraeyman et al., 2016.

"Dispensing doctors and retail pharmacists have always been arch enemies. So, while relationships (between our practice and the pharmacist) were never particularly hostile, I wouldn't say they were particularly good either. Actually, being able to talk to one another and use their skills is a relative innovation for a dispensing practice… We are certainly getting much more from the pharmacists since we went on the scheme than we had before. (GP 01)." – Elvey et al., 2008.

"As revealed by the interviews, pharmacists considered the remuneration for NMS as an important step in the evolution of pharmaceutical care and as a motivation for pharmacists to perform NMS. In contrast, the focus groups with the GPs indicated that they found it unnecessary or even inappropriate to give a fee to pharmacists to provide this kind of service, in proportion to existing services in general practice." – Fraeyman et al., 2016.

"But pharmacists receive 40 euros for two consultations. We receive 30 euros for a pile of administrative work. That is not in proportion! I think what we do for the Global Medical patient Record (GMR) is much more extensive than what the pharmacist does for this NMS." (Focus group with GPs)." – Fraeyman et al., 2016.

"Interprofessionally, in this regard, issues arise around, for example, the perception that pharmacists are 'shopkeepers' with conflicts between their health care and business roles. This was identified by general practitioners in the UK as being a key reason they did not support the extension of prescribing rights and other extended services to pharmacists." – Bidwell & Thompson, 2015.

"Fears about conflicts of interest, particularly as this related to dispensing self-written prescriptions, was raised by some of those concerned about the extension of prescribing to some pharmacists in the New Zealand context." – Bidwell & Thompson, 2015.

"GPs remained opposed to pharmacists recommending herbal medicines, suggesting pharmacists would rather make a sale than adhere to evidence-based practice for particular remedies. The tainted 'shopkeeper' role has been well documented. Although pharmacists remained ambivalent over time, results concurred with the requirement for scientific evidence and better training when recommending herbal medicines." – Bryant et al., 2017.

"That the paradigm shift from a product-focussed profession of dispensing to a more patient-focussed one with 'shared' responsibility for optimal drug therapy outcomes required the development of an ethical covenant between the pharmacist and patient. This was considered important, particularly in some organisational settings, e.g. chain drug stores, because there was an increased opportunity for ethical problems to arise where there could be an inherent conflict between professional values and organisational demands. For example, where the primary reimbursement is from dispensing prescriptions and selling retail products, a conflict may arise when more time is required for patient-focussed activities such as counselling." – Bryant et al., 2010.

"One study found that GPs particularly favoured schemes relating to repeat dispensing as they thought that pharmacists could carry some of the burden of repeat prescribing. However, the same study also described 'ambivalent' GP attitudes to pharmacists' extended roles, with a pervasive concern that pharmacists must remain within their 'limitations'. Similarly, Zermansky contended that community pharmacists were well placed to contribute to the repeat-prescription management process, but expressed some reservations about their clinical skills and competitive commercial relationship, while a focus-group study found that GPs supported the extension of community pharmacists' roles but the prevailing view of the pharmacist as a 'shopkeeper' remained a barrier to interprofessional working." – Elvey et al., 2008.

"GPs thought that if prescribing were undertaken outside the GPs' surgery, and without close communication, this could fragment patient care, reduce the frequency of GPs seeing their patients, and decrease their opportunities to talk with their patients about other health concerns. GPs were also worried that those services could conflict with pharmacists' business interests." – Hatah et al., 2013.

"Some studies reported public suspicion over the pharmacy commercial affiliations and financial incentives." – Brewster et al., 2020.

''I think the obstacles will be primarily psychological. I mean people might feel the hospital is more suitable and a safer place for providing these services. However, over time, people's attitude would change, especially if they see it done professionally without a focus on the business side, as community pharmacy are mostly profits oriented." – Almansour et al., 2020.

"Some [patients] were surprised at how much the pharmacist knew about the medicines they were taking. However, when considering whether the pharmacist should make recommendations to the GP for their treatment they expressed more anxiety, citing concerns regarding drug companies' influence on pharmacists, and their need to make profit and so preferred that the GP should make the final judgments on any treatment recommendations." - Twigg et al., 2013.

"Internal barriers to communication ''I guess some GPs consider CPs just to pull drawers the whole day (FG2, CP1).''" - Weissenborn etal., 2017.

"Conflicting opinions were expressed regarding the provision of MURs. Some believed they benefited patient care, others saw it as a reason for increased GP workload and paperwork, with little patient benefit. The ability of some pharmacists to undertake this form of review and their motivation was also questioned, for example, to reach company targets." - Deslandes & Frazer, 2011.

"[CPs] they perceived that the retail environment of community pharmacies reinforced negative perceptions of patients towards their suitability as providers of extended healthcare services." – Hindi et al., 2018.

"Hughes and McCann identified barriers to interprofessional collaboration in Northern Ireland. They identified a key barrier as the 'shopkeeper' image of pharmacists, arguing that the conflict between providing health services and running a business limited the willingness of GPs to collaborate with pharmacists." - Rathbone et al., 2016.

"Typically, if you start entering targets and it becomes a financial incentive and then when you hear about [large pharmacy chain] doing NMRs [sic MURs, medication use reviews] on each other ... I get a bit more sceptical about the use, whether it's actually for patient focus and quality or is this just another measure of working out how much money do people get (GP)." - Watson et al., 2020.

"Most focus groups discussed community pharmacists' links with commercial enterprise. This was seen to be at odds with the NHS healthcare provider role. Participants questioned whether pharmacists prioritised profit margins over patient care. This is exemplified by a discussion of blood pressure monitoring services. Pharmacists were seen to be selling blood-pressure monitoring equipment at a profit whilst the GP surgery offered blood-pressure testing services free of charge." – Gidman & Cowley, 2013.

"I'm listening to what you're saying here about the blood-pressure tests, etc. Surely when a chemist is doing blood-pressure checks… They're selling equipment or trying to sell equipment.' (R17)." – Gidman & Cowley, 2013.

"Comparisons of this sort raised doubts about pharmacists' integrity when providing advice to buy products. In addition to this, older participants specifically voiced concerns about other issues which undermined pharmacists' credibility. By way of example some older participants were suspicious of pharmacists' motives for supplying unbranded prescription medication. There was conjecture that unbranded medication was inferior and supplied to maximise pharmacy profits." – Gidman & Cowley, 2013.

"The business image of community pharmacy was viewed as conflicting with intentions pharmacists may have of delivering patient-centred care and as impairing the credibility of pharmacists. Pharmacists here are really . . . under-used [for their] professional knowledge, underpaid, and business oriented. (FH-I#1)." – Hasan et al., 2015.

"This also applied to requests from community pharmacists to substitute branded medicines prescribed by physicians; some participants perceived that these requests were influenced by business interests. "I should decide the medication, and a pharmacist can discuss with me regarding clinical [use], if I'm right or wrong, but not to guide me which [product] to write – then I'll enter into market [competition]. (HT-I#1)" – Hasan et al., 2015.

"Others criticised the 'must sell' approach some pharmacists take in dealing with requests for advice about health or management of simple ailments. Physicians were particularly concerned about over-the-counter sale of antibiotics:

What happens usually, I'm telling you real practice, the pharmacist doesn't only give . . . suppose Panadol, he gives antibiotics, he gives other medications. So, this should be stopped . . . first of all, most bacteria are resistant to antibiotics because of this practice [of prescribing antibiotics]. And many, many drugs which are not controlled, they give just like that, just for money… I can't trust such practice. (AM-F#5)" - Hasan et al., 2015.

"And many times, you [the patient] don't need anything and while we are motivated to say that you don't need anything, the pharmacist, if he is making money by selling something, his motivation is going to be different, and I see that as not necessarily to the patients' best advantage.' [GP2]." – Hughes & McCann, 2003.

'As GPs we want to make a profit as well and we are healthcare providers, but I think it is all the commercialism around the chemists' shops.' [GP20]." – Hughes & McCann, 2003.

"All of the interviewed physicians perceived that the CPs' role was more heavily weighted towards that of a retailer/shopkeeper than a healthcare professional." – Rieck & Pettigrew, 2013.

"Well, they make a lot [more] money out of retailing than they do out of prescribed medication. So, they're actually businessmen. So murky waters, if you're a pharmacist, that's what you do." Physician 3 M MSES (28 years of experience). – Rieck & Pettigrew, 2013.

"It is what kills the ability for pharmacists to say that they're health professionals, because they are making a profit based on everything sold. So many of them are running a business, and that's very evident from what's on their shelves. And from the fact that they're equally prepared to push a hangover cure as they are to talk about something that's got a lot more evidence behind it." Physician 6 F MSES (22 years of experience) – Rieck & Pettigrew, 2013.

4. **Siloed working**

When GPs and CPs are unconvinced of the benefits of collaborative and integrated working (C), they may continue working in silos (O) because this is what they are used to (M).

“Community pharmacists typically work in a silo within the walls of pharmacy. The problem arises when there is a need for interprofessional collaboration which requires communication and teamwork skills.” - Hermansyah et al., 2020.

“GP-pharmacist collaboration as consisting of three stages: isolation, communication and collaboration.” - Van et al 2013 & Van et al., 2012.

It is fair to say that the pharmacy education system in Indonesia has significantly changed over a decade. However, the challenge as aforementioned is to prepare graduates that can collaborate and communicate with other professionals and the patients. It is indeed an explicit call from the Association of Indonesian Pharmacy Higher Education (APTFI) for the integration of curriculum content and practice experience i.e. period of workplace learning in form of interprofessional education. There is evidence that interprofessional education can contextualize learning, develop skills, knowledge, and values to become professional pharmacist. Whilst there is a small portion of pharmacy schools strive to pursue such integration, the majority might need to be stimulated.” - Hermansyah et al., 2020.

“Participants also referred to what they saw as the isolated position of the pharmacist within primary care, as they did not see them as fully integrated with the rest of the primary care team. In characterizing what the pharmacist could provide they highlighted it as lacking in joined up care and as duplicating services they received from the medical practice. This aligns with other research that has highlighted the isolated working practices and environment of the community pharmacy and is something that, professionally, community pharmacists themselves can do little to alter.” - Twigg et al., 2013.

“People want to know that people are in the loop, people want to know that their care is not just provided one bit over here, one bit over there, one bit over there, all disconnected. In fact, the biggest problem we have in our system is that there are so many silos of care.” – PHN1” – Hossain et al., 2018.

5. Scepticism

When GPs and CPs are sceptical about the benefits of collaborative and integrated working (C), they may be less likely to engage and cooperate with one another (O) because they cannot see the value (M).

"Apparent threats were the GPs' perceptions of a related, and not renumerated, increase in their workloads, and the perception of limited benefit to patients." - Hatah et al., 2012.

"Both GPs and pharmacists had expectations of their counterpart. Whether a practitioner was able to meet the expectations of their counterpart affected how well the pair interacted. At the beginning of a professional relationship, expectations revolved mainly around GPs being expected to write prescriptions correctly or pharmacists being able to dispense medications correctly. As basic expectations were met or exceeded, rapport, trust and confidence in the practitioner's abilities improved and future expectations increased. For example, after years of working together with several GPs in a nearby medical centre, a pharmacist reveals the GPs expected her to play a larger role in patient care and provide extra services." - Van et al., 2011.

"Some GPs thought that pharmacists' contributions to medication reviews might be a threat to patient care, as the advice provided could conflict with that given by the GP (e.g., about a clinically irrelevant drug interaction) and hence might confuse patients and harm GP–patient relationships." – Hatah et al., 2013.

"I would not want to refer patients to the pharmacist for review only to find that the patient comes back and tells me that I shouldn't have done this, I shouldn't have done that. So, it's a relationship that could be undermined by the pharmacist if they're not careful what they're saying. (GP#3, G3)." – Hatah et al., 2013.

"Whether the GP saw a role for the pharmacist in medication safety and effectiveness was the variable found to be the strongest predictor in our model." – Van et al., 2013.

"Extension and/or expansion of pharmacist roles were met with caution by GPs, although there was greater acceptance of medicines management. Pharmacists had mixed views about role expansion. Most were keen on role extension, particularly in relation to medicines management." – Bidwell & Thompson, 2015.

"GPs were considerably more divided about the increasing role of pharmacists in a range of areas, and for some this also included reviewing medicines through MMS encounters. Some were unreservedly enthusiastic, finding it saved time for them and benefited their patient." – Bidwell & Thompson, 2015.

"Others were neutral though unconvinced they were either necessary or valuable, and a few GPs were directly negative, seeing the involvement of the pharmacist in managing their patients as an intrusion into the GP's core role." – Bidwell & Thompson, 2015.

"A few GPs were noticeably unenthusiastic about the need for or the benefit of closer relationships." – Bidwell & Thompson, 2015.

"All participants, even those most positive about collaboration, shared the range of concerns about the uncertain impact of the developments in pharmacy on workload, reimbursement and professional boundaries." – Bidwell & Thompson, 2015.

"The current evaluation study suggests that physicians and patients were generally not aware of the intervention and that pharmacists lack collaboration with physicians. However, the acceptance of the intervention was also a topic of debate in the focus groups. GPs did not see the added value of the NMS intervention, and some found it inappropriate that pharmacists receive a fee for this kind of services." – Fraeyman et al., 2016.

"Roberts et al, have shown that collaboration with physicians was the most important factor for successful implementation of interventions in the community pharmacy. Future NMS could benefit from formalizing the role of the physician in the service." - Fraeyman et al., 2016.

"Pharmacists were also thought to lack the appropriate skills to prescribe or review patients' medications clinically. Some patients' unfavourable experiences with pharmacists' advice on over-the-counter medications had raised GPs' doubts about pharmacists' clinical skills." – Hatah et al., 2013.

"I think it depends on what the condition is and how well the pharmacist has trained as a diagnostician… I'm not convinced that pharmacists or nurses have that background. (GP#6, G2) [Speaking about pharmacist prescribing]." – Hatah et al., 2013.

"Conflicting opinions were expressed regarding the provision of MURs. Some believed they benefited patient care, others saw it as a reason for increased GP workload and paperwork, with little patient benefit. The ability of some pharmacists to undertake this form of review and their motivation was also questioned, for example, to reach company targets." - Deslandes & Frazer, 2011.

"The only way I think everything could improve would be to have some way of getting the GPs back into the loop and stressing to them how important it is that patients know about any new medications they've been put on… For any service to work you need to have everybody engaged with the actual service and it's just this problem that the lack of responses from GPs. (Pharmacist, Lucas & Blenkinsopp, 2015)." – Hindi et al., 2018.

6. **Psychological readiness to change**

When GPs perceive CP services as a threat to their income, clinical autonomy, and/or expertise (C) they are less likely to engage and cooperate with CPs (O) because they feel threatened (M).

"GPs during the focus groups indicated that they did not perceive the Asthma Control Test (ACT) as an appropriate tool to be used by pharmacists, as this could imply that the latter is performing a clinical act. Some GPs experienced this kind of service as a threat to their expertise." - Fraeyman et al., 2016.

"It bothers me that they want to take something away from the GPs. Not only asthma, but they will also take hypertension, they will take cholesterol… that is my remark. We have studied for nine years. Pharmacist [studies] take five years, and now they are completely invading our domain." (Focus groups with GPs)." - Fraeyman et al., 2016.

"Some GPs thought that pharmacists' contributions to medication reviews might be a threat to patient care, as the advice provided could conflict with that given by the GP (e.g., about a clinically irrelevant drug interaction) and hence might confuse patients and harm GP–patient relationships." – Hatah et al., 2013.

"I would not want to refer patients to the pharmacist for review only to find that the patient comes back and tells me that I shouldn't have done this, I shouldn't have done that. So, it's a relationship that could be undermined by the pharmacist if they're not careful what they're saying. (GP#3, G3)." – Hatah et al., 2013.

"I set up that meeting at the practice, just to explain what an LPS [local pharmaceutical service] was and what we were trying to do and what we were trying to achieve and how the pharmacist was there to help and support the practice in managing that group of patients and they said, 'We engage terribly well with our patients, we do not need any help and we would appreciate it if you didn't interfere with us'. (Site E, PCT Pharmaceutical Advisor)." – Bradley et al., 2008.

"One pharmacist about informing general practitioners: ''previous experience with doctors made me decide not to inform them. They do not want pharmacist's pharmaceutical care projects. They believe that we are trying to take their place. If necessary, I take their contact details because then I can call them about a specific problem of a certain patient." – Adhien et al., 2013.

"The GPs considered the major threats to their own work were time constraints and increased workloads (carrying out recommendations from pharmacists). Even though GPs thought pharmacist prescribing could potentially compete with GPs' own work, they thought that it would not be a problem currently as there are shortages of doctors in NZ." – Hatah et al., 2013.

"…a few GPs were directly negative, seeing the involvement of the pharmacist in managing their patients as an intrusion into the GP's core role. Pharmacists, although they welcomed the opportunity to take an increasing part in medicines management were also keenly aware of GP sensitivities." – Bidwell & Thompson, 2015.

"You're stepping into a more clinical report, and we have to … try and straddle that without stepping on too many toes. The service is still very new and there are some GPs that are very welcoming of the service and there are some that appear to be feeling threatened by it or haven't perhaps have got as good a working relationship with the pharmacies and so they, for whatever reason, are not as open to receiving feedback. (Pharmacist)." – Bidwell & Thompson, 2015.

"Attempts to encourage one professional group to expand or extend their practice may be perceived as a threat by those adjacent." – Bidwell & Thompson, 2015.

"It is important to recognise that any change that has implications for more than one profession has the potential to be construed as a threat." – Bidwell & Thompson, 2015.

"The GPs considered the major threats to their own work were time constraints and increased workloads (carrying out recommendations from pharmacists). Even though GPs thought pharmacist prescribing could potentially compete with GPs' own work, they thought that it would not be a problem currently as there are shortages of doctors in NZ." – Hatah et al., 2013.

"I don't know if there's anywhere in NZ where there are too many GPs, it might be seen as a turf war when there was not enough business… But I think New Zealand is under-provided with GPs and so, in general I think it [pharmacists' medication-related services] would be welcomed. (GP#10, G1)." – Hatah et al., 2013.

"Pharmacists' attempts to re-professionalise have been reported as threatening to GPs' status, autonomy and control. When working collaboratively, role clarification for both professions is paramount." – Bryant et al., 2017.

" Internal barriers to communication: 'I've experienced GPs and their employees to react very sensitive when they are made aware of errors that occurred on their part. Therefore I think it's better to paraphrase the problem while directly talking to each other (IDI, GP2).'' - Weissenborn et al., 2017.

"GPs had reservations surrounding the loss of their professional autonomy if pharmacists were to significantly extend their role. Dr 9 'depends if you want to do us [GPs] out of business or not' - Deslandes & Frazer, 2011.

"This is a small-scale exploratory study which examined the views of GPs on the community pharmacist's role. Previous research has highlighted that doctors may fear for their autonomy if the pharmacy profession continues to expand. Support was expressed, however, with the caveat for clear boundaries of responsibilities within the health care team and increased awareness of the pharmacist's role." - Deslandes & Foster, 2011.

This perception was influenced by GPs' negative attitudes towards pharmacists, GPs being suspicious of pharmacists' financial motives (Urban et al., 2008) and competition for services." – Hindi et al., 2018.

"Once they saw a lot of patients choosing to come here [to the pharmacy] straight away they got really, really defensive and made all kinds of sort of threats to try and stop me from doing it." – Evans et al., 2016.

Pharmacists also believed that any extension of their role would be seen as an encroachment on GP activity:

"We have not got pharmacy prescribing yet, … but that would be seen by some as an invasion of their territory and the thin edge of the wedge, and some would see it as brilliant. A lot of GPs, with some justice, they feel that we are crossing on to their territory and taking away their territory.' [P8]

"'GPs are very reluctant to relinquish any sort of control to us. The pharmacist is an outsider and to a certain extent is a threat to the GP.' [P30.]" – Hughes & McCann, 2013.

"It is my responsibility. I do not expect anyone else to take part of the blame if something goes wrong. And in that respect, I must say that I feel that I should be the one in charge. So, if the pharmacist advises the patient very differently than what I have decided, I can get a little insulted." (GP 2, group 3) – Rakvaag et al., 2020.

Infrastructure

7. **Limited central infrastructure and commissioning support**

When GPs and CPs are not contracted or remunerated to collaborate and provide integrated care (C), they may be more reluctant to work together on collaborative tasks (O) because of the perception that collaboration is not worthwhile (M).

"This study also sheds light on the important issue of contracting and incentivizing practitioners to provide integrated care. GPs were not contracted nor incentivized to co-operate with the LPS [local pharmaceutical services] pilots." – Bradley et al., 2008.

"A barrier influencing the manager's interest was the perception that the NHS did not incentivize collaboration." – Rubio-Valera et al., 2012.

"Lastly, complex system designs including pharmacy contracts and commissioning routes are poorly understood, further disadvantaging the involvement of community pharmacy in the negotiation of

evolving care models and more integrated working. These barriers are not unique to England and have been described in other countries." – Brewster et al., 2020.

"The main results from the study show that the intervention was not sufficiently embedded in the existing health care organization, meaning there was a lack of communication from the service commissioner toward patients, pharmacists and physicians alike." - Fraeyman et al., 2016.

"As revealed by the interviews, pharmacists considered the remuneration for new medicines service as an important step in the evolution of pharmaceutical care and as a motivation for pharmacists to perform new medicines service" – Fraeyman et al., 2016.

"This resulted in a lack of engagement from the participants (pharmacists and patients) and other health care professionals (GPs and lung specialists), causing a low uptake of the service and a large resistance to its implementation." - Fraeyman et al., 2016.

"Initiating collaboration involved extra work for these busy professionals who received no additional remuneration for it." – Rubio-Valera et al., 2012.

"GPs and CPs stated that their attitude would be even worse if the NHS forced them to collaborate without releasing them from other duties or offered economic incentives." – Rubio-Valera et al., 2012.

"However, integration of community pharmacy into primary health care faces challenges, including the lack of realistic integration in PHNs, and in service and remuneration models from government." – Dineen-Griffin et al., 2020.

"A significant barrier to pharmacists achieving high-impact contribution as integrated members of the health team is the current state of remuneration for services." – Dineen-Griffin et al., 2020.

"The right incentives must be provided for all health practitioners, like GPs, nurses and pharmacists to integrate services effectively, with clear responsibilities contributing to overall health outcomes." – Dineen-Griffin et al., 2020.

"The Pharmaceutical Society recommends funding that reflects quality and complexity of pharmacist care. This may be achieved through establishing practice incentive payments linked to quality measures, revising remuneration structures to account for complexity, or moving to a time-based fee structure. All aspects of the pharmacist's role need to be resourced appropriately." – Dineen-Griffin et al., 2020.

"GPs were also concerned that they could be given extra work (e.g., reviewing pharmacists recommendations) without remuneration." – Hatah et al., 2013.

"What worries me is that it will be time-consuming and not remunerated anyway. (GP#7, G2)."- Hatah et al., 2013.

"Poor co-operation with GPs and lack of time as well as financial compensation were interfering factors." - Adhien et al., 2013.

"Poor co-operation between pharmacists and GPs and lack of re-imbursement are obstructions." - Adhein et al., 2013.

"The pharmacy teams experienced problems in the implementation of the intervention. They specifically complained about a lack of time and shortage of staff. In addition, they reported that under these conditions the lack of financial compensation impeded the implementation of the intervention more widely." - Adhien et al., 2013.

"Apparent threats were the GPs' perceptions of a related, and not renumerated, increase in their workloads, and the perception of limited benefit to patients." - Hatah et al., 2012.

"Many respondents also believed that while remuneration for GPs and pharmacists participating in HMR [home medicines review] was only modest it allowed for a sustainable service. However, several called for remuneration for case conferencing after HMRs to encourage further collaboration: 'Ideally, if it's possible, we should maybe sit down and talk about the patients. Maybe have some sort of conference. If conferencing with the local chemist was sponsored that would be good." – Van et al., 2011.

"Financial incentives and motivational rewards to pharmacists providing these services were also thought to be essential for sustainability." - Almansour et al., 2020.

''We can encourage them [Pharmacists] by focusing on offering them three types of incentives: monetary, non-monetary, and social incentives. Non-monetary incentives should be often linked to the pharmacist's ability to serve the community. By social incentives I mean making people identify the valuable and significant role of pharmacists in the society." – Almansour et al., 2020.

"In July 2012 a new funding model, The Pharmacy Services Agreement, was signed between community pharmacists and District Health Boards, changing the existing model which had operated for more than 60 years. Consistent with the above shift in philosophy, the new model was designed to encourage patient-centeredness and integration between prescribers and pharmacists, to incentivise pharmacists to better use their medicines management skills and to attempt to limit pharmacy dispensing costs." – Bidwell & Thompson, 2015.

"All participants, even those most positive about collaboration, shared the range of concerns about the uncertain impact of the developments in pharmacy on workload, reimbursement and professional boundaries." – Bidwell & Thompson, 2015.

"Current barriers to this model identified were: medico-legal issues, budgetary issues, limited access to patient data, lack of privacy to talk to patients, inadequate time and remuneration, no patient registration, patients describing vague symptoms and patient confidence in pharmacists." – Aradottir & Kinnear, 2008.

"The absence of community pharmacy accreditation and poor remuneration system for pharmacy services are also among the factors hindering the sustainability of pharmacy service delivery." – Hermansyah et al., 2020.

"This review highlights the need to incentivise joint working between community pharmacists and GPs to achieve better integration within the patient's primary care pathway." – Hindi et al., 2018.

"Interestingly, remuneration was raised as a potential strategy. GP participants suggested that the availability of remuneration would allow a dedicated interprofessional adherence support strategy to be developed and implemented in the community setting." - Rathbone et al., 2016.

"This may involve changing the perception of the pharmacist by patients but also, and possibly more importantly, changing the way pharmacists work and are paid by the NHS. Changes to the national pharmacy contract may be appropriate to ensure pharmacists have the time and space to not only engage effectively with patients but also members of the wider primary healthcare team." - Twigg et al., 2013.

"Key barriers to the achievement of initiating, conducting high quality medication reviews and follow-up discussions were perceived to include inadequate financial and process supports for collaborative practice." - Disalvo et al., 2019.

"Almost all interviewees perceived the RMMR programme to offer inadequate remuneration to enable the collaborative practice required." - Disalvo et al., 2019.

"I think remuneration being what it is, it's not a good viable model. It's 'one size fits all'…But there's an increasingly complex cohort, if you really want that person-centred care, then there has to be a change in the remuneration structure…We really need to have the capacity to provide a more in-depth service for those who warrant it, and they should be remunerated accordingly… that's why you end up having people [pharmacists] feeling disillusioned because they end up putting more in than the remuneration is providing for. (P12)." - Disalvo et al., 2019.

"A lack of remuneration for case conferencing, especially, was seen as stymieing interdisciplinary communication required for high quality RMMR-related collaborative practice." - Disalvo et al., 2019.

"If there was some ability where we could be remunerated for case conferences, and pharmacists aren't obviously at this stage. GPs are, but if we could tap into that sort of funding…where we could all sit down together and discuss patients, or residents of aged care facilities, and determine a management plan that way I think. (P09)." - Disalvo et al., 2019.

"CP12F: Paid work is transformed into goodwill. (…) That is the core problem. CS14M: I believe this, too. (…) The pharmacist, just like the physician, invests his time and work (in medication optimization) without getting any compensation for it. (…) Of course, there is a problem at that point." – Gerlach et al., 2020.

"CP12F: We just needed the possibility to provide consulting hours in which patients can come and ask questions about things that we have noticed. But we would also need some kind of monetary compensation for this consultation. And principally this could also reduce the doctor's burden." – Gerlach et al., 2020.

"CP12F: We have an obligation to give counsel and we must check interactions. We have already received this mandate and we are penalized if we don't comply with it. But unfortunately, it is an additional expense and really takes time, but we are not being refunded for this. That's the great problem." – Gerlach et al., 2020.

"Participants described the need to remunerate community pharmacists for time spent on clinical non-dispensing services (e.g., communicating with other members of the health care team), which were viewed as important for integration of care." – Lake et al., 2020.
"If there was a compensation model. It would be a much easier sell. Because right now it's done out of the goodness of your heart because it's really difficult to bill for it. (Pharmacist, P04)." – Lake et al., 2020.

"I mean we always say remuneration […] that would really help pharmacists spend that extra time on the phone or researching something for a physician." (clinician) – Lake et al., 2020.

8. Organisational leadership and management support

When an organisation establishes a culture that values and promotes collaborative and integrated ways of working (C), GPs-CPs are likely to actively choose to work with one another on collaborative tasks (O), because of signalling (M).

"To collaborate it was necessary that the PCHC manager was interested in promoting collaboration." – Rubio-Valera et al., 2012.

"The interest of the PCHC manager was influenced by his/her own perception of usefulness and by the initial cost in terms of infrastructure and human resources required to trigger the collaboration." – Rubio-Valera et al., 2012.

"Process factors mentioned by pharmacy staff included use of opinion leaders, especially management within the company. Administration in the pharmacy was typically described as "top down" decision making with support provided by management to implement decisions. For example,

one pharmacy staff member explained, "…[management] really support us doing these things… these new services. [Manager] helps us find help to get started and points us in the right direction with how to track and record…" Overall, pharmacy staff felt they were given the resources and support from management needed to successfully provide HPV vaccines and participate in the VFC program." – Teeter et al., 2021.

"If the new management team was not in favour of collaboration, practitioners would no longer have time or support to conduct meetings, collaborative work, etc." – Rubio-Valera et al., 2012.

"One of the most relevant factors in promoting or initiating the GP–CP relationship is the perception that these professionals have regarding the usefulness of the collaboration." – Jove et al., 2014.

"Many respondents believed that for PPS [professional pharmacy services] to work both practitioners must 'want to work together' (P02) and share the belief that they were working towards the same goal thus justifying the need for teamwork. 'I think it's easier working with some doctors because we share the same belief in what we're here for . . . we're both part of the total solution for patients . . . we're meant to work together.' (P16)" - Van et al., 2011.

"In recognition of the need for physicians and pharmacists to work together, arrangements for physician–pharmacist collaboration have been made legal and/or financed in countries such as Germany, Switzerland, and the US. For example, under 'collaborative practice agreements', US physicians may legally delegate some patient-management responsibilities to pharmacists, and pharmacists have the autonomy to modify patient medication regimens." - Van et al 2013.

"A positive perception of usefulness was necessary in order to start the collaboration." – Rubio-Valera et al., 2012.

"Locality groups, where GPs and pharmacists meet to discuss services including repeat dispensing, can be helpful. Efforts to highlight benefits to practices, such as the potential to score points under the Medicines Management collaborative indicators in the Quality and Outcomes Framework (QOF), the system through which payments are allocated to general practice, may also be worthwhile." – Elvey et al., 2008.

"Having worked with the pharmacists for long enough now, best part of this pilot scheme, my confidence in their ability has grown. At first, you know, I wanted Integrating pharmacists into the primary health care team them to explain exactly all the details of the case and what the alternative treatments were and what the alternative diagnoses were, but now we've worked with each other for a bit longer, I think I have more confidence in their clinical ability. (Site A, GP)." – Bradley et al., 2008.

"For the relationship to be maintained over time, both professionals and the PCHC manager had to recognize the benefit of this collaboration. There were a number of factors that could help to fulfil the goals. According to GPs and CPs, it seemed essential to have a coordinator(s) or reference person(s), responsible for leading the collaboration and linking the two professional groups." – Rubio-Valera et al., 2012.

"Many of the GPs articulated a desire to continue working with the pharmacists and suggest- ed other areas of disease management in which they could become involved. Most of these areas comprised one or more aspects of cost reduction. One said: "There is no doubt he could become a member of the team here. I mean there is obviously a lot more work we can do together… I mean big areas like gastrointestinal disease, well that's a massive area. I could see him [the pharmacist] interviewing these patients, getting their prescriptions sorted out, telling them what their drugs are for, perhaps even converting them to cheaper therapy." (GP 7)" - Woolley & Cantrill 2000.

9. Inflexible systems

When IT systems and technologies used for collaboration are inflexible and cannot easily be adapted to fit professional practice and patients' unique needs (C) this may lead to the avoidance and abandonment of technological systems (O) because users perceive them to be unhelpful (M).

"The administrative procedures for the organization of local projects seem to have hindered the implementation of the Medical-Pharmaceutical Concertation (MPC) project. These procedures were unanimously described as too extensive and too complex by project leaders, which meant they had to ask for help of professional associations. Leaving these procedures untouched might result in a decrease of service users perceived behavioural control and eventually in them not organizing local projects anymore." – Damiaens et al., 2021.

"Pharmacists and GPs both felt the opportunity for the systems to deliver transformation in prescription-related communication practices between prescribers and pharmacists was yet to be fully realised. Although some tools were built in, participants described pitfalls such as not knowing where messages ended up or when they had been read, and a limited number of interchanges." – Campbell et al., 2021.

"They particularly disliked their role in policing the regulations around prescriptions whereby they were required to check up on minor errors caused by the way the GP prescribing software was set up (for example, if the GP inadvertently selected 90 tubes instead of 90 tablets from the drop-down menu). Pharmacists were very conscious that they risked annoying GPs by contacting them over small details and yet they were not legally able to make the change themselves." – Bidwell & Thompson, 2015.

"Three key thematic barriers were identified as contributing to the low uptake of the clinical services delivered by pharmacists. First, poor integration with other parts of the NHS, largely as a result of the limited capability of available digital platforms." – Brewster et al., 2020.

"The biggest problem, though, concerns the way it doesn't pick up the compliance rate properly. If you issue a repeat dispensing item and they come back six months later the computer assumes they haven't had any medication for six months, so every time you then have to repeat the item it says,

'this patient has not been taking the medication, are you sure you want to issue it?' for each item. (GP 12)." - Elvey et al., 2008.

"Interviewees felt that the initiation process and software issues had constrained uptake of repeat dispensing and that negative perceptions still prevailed, which had discouraged some GPs from engaging in the scheme." - Elvey et al., 2008.

"Limitations of existing computer support systems and information resources [e.g. British National Formulary (BNF), a standard reference text] were noted, particularly in terms of their clinical relevance and application in the context of older patients receiving polypharmacy." - Cadogan et al., 2015.

"There was also minor concern with how administration at some of the clinics would react. For example, one physician explained, "I think it's a brilliant idea. As long as the systems are updated and there is a way for us to know what vaccines have been given… the issue would be more with administration than anything." – Teeter et al., 2021.

"However, this study underlined that—in contrast to standardization—an approach allowing individualization is needed." – Weissenborn et al., 2017.

"When you know what is needed and you know what the doctor is going to say, well it's hard to say to the patient 'I'll have to discuss this statin with your doctor, or this change with your doctor'. I mean if we had a proper agreed sort of scheme, we could move patients through, something whereby we could make the changes and then maybe just notify the doctors later." (Pharmacist 3)." – Woolley & Cantrill, 2000.

"Attention should be given to educating physicians about the breadth of services that pharmacists can provide, building pharmacist–physician relationships to enhance patient care, and developing systems to promote pharmacist–patient communication." - Tarn et al., 2012.

"Systems encouraging better physician–pharmacist communication could facilitate patient medication management." - Tarn et al., 2012.

10. Capacity

When GP and CP staff have multiple demands on their time (C), they are more reluctant to undertake additional tasks (O) because they do not have the capacity to do so (M).

"The pharmacy teams experienced problems in the implementation of the intervention. They specifically complained about a lack of time and shortage of staff." – Adhien et al., 2013.

"GP practices and community pharmacies are under intense pressure, mainly due to government funding cuts and increased workloads. It can be hard to find time for a lunch break, let alone to set up a meeting with your local surgery." – Jankovic, 2017.

"Community pharmacists typically work in a silo within the walls of pharmacy. The problem arises when there is a need for interprofessional collaboration which requires communication and teamwork skills." - Hermansyah et al., 2020.

"Rural health practitioners experience difficulties related to professional isolation and lack of opportunities for professional development." – Bidwell et al., 2017.

"During the interviews there were a number of comments made regarding clinical skills, personal confidence and the desire for peer support, with an indication that a number of the pharmacists felt they were working in isolation." – Bryant et al., 2010.

"Despite a mutual interest in optimising the benefits of medication for patients, the general practitioner (GP) and community pharmacist (CP) often work in isolation from one another, both physically and figuratively." – Bradley et al., 2018.

"Despite encountering the same patients and sharing a common interest in optimising the benefits of medication for these patients, it is still the norm for the two professions to work in isolation from one another both physically and figuratively." – Bradley et al., 2018.

"In a study of New Zealand community pharmacists, general practitioners, general surgeons, and general physicians, community pharmacists were rated the most stressed and dissatisfied, overwhelmed by paperwork, and more likely to be contemplating giving up their job because of work stress. With this level of dissatisfaction and psychological stress, it is not surprising that community pharmacists are not particularly amenable to, or capable of, change." – Bryant et al., 2009.

"However, for a number of HCPs, their levels of attention ('Memory, attention and decision processes') and commitment ('Motivation and goals') to carrying out these procedures as part of routine practice were lacking, particularly in light of existing time and workload barriers ('Environmental context and resources')." - Cadogan et al., 2015.

"The main barriers that hindered both HCP groups in ensuring that older patients received appropriate polypharmacy related to the current work environment ('Environmental context and resources'). For example, in addition to time and workload barriers, a lack of appropriate resources (e.g. staff) was also noted by both groups." – Cadogan et al., 2015.

"Interprofessional interactions occurred via telephone while a small number reported email and face-to-face exchanges. As illustrated in the following data extract: '[We communicate] usually via telephone. Face-to-face interactions are not practical due to our workload, phoning is the quickest way to get information. We do email but not for quick information… [I] only check it once a day or once every two days.' (GP08)" - Van et al, 2011.

"Both GP and pharmacist respondents felt regular communication that focused on clinical issues engaged both parties in professional dialogue and helped build rapport and trust. In turn rapport and trust lead to open, honest, and bilateral communication between GPs and pharmacists helping them work together more effectively: 'Constant communication is the thing that probably helps the most. The doctors that we talk to the most are the ones that give the most feedback and are more likely to respond to requests either by accepting requests or telling us why they don't want to. I feel that is just because we already have some dialogue happening.' (P04)" - Van et al., 2011.

"The extent of accessibility of both GPs and pharmacists were highlighted as barriers to GPs and pharmacists working together. Nearly all respondents cited a lack of time as the main reason for not working together with their GP or pharmacist colleagues. Indeed, several pharmacists noted that GPs were not always easily accessible. In contrast, GPs, and pharmacists whose counterparts were easily accessible found it easier to collaborate and develop positive interprofessional relationships: 'You tend to make better relationships with the ones you interact more with and the [more physically] closer you are to them the easier it is to see and interact with them.' (GP06)"- Van et al., 2011.

"The GPs considered the major threats to their own work were time constraints and increased workloads (carrying out recommendations from pharmacists)." – Hatah et al., 2013.

"Many pharmacists commented that advising doctors about medicines should be a core part of their role, but their ability to do this was limited by time constraints: There's a lot more scope for pharmacists to be involved in helping GPs manage their patients, in particular certain types of patients as well, in terms of reviewing medications that they're on. Things that GPs just don't get time to do. (Pharmacist)." – Bidwell & Thompson, 2015.

"All participants, even those most positive about collaboration, shared the range of concerns about the uncertain impact of the developments in pharmacy on workload, reimbursement and professional boundaries." – Bidwell & Thompson, 2015.

"As we already have a lot of work, it couldn't work, and it was cancelled. Because we all have enough work… I don't think there was any other reason, there was no misunderstanding or anything else. It was just this, the pressure they were under and ours too. [GP4: GP WBCN]." – Rubio-Valera et al., 2012.

"You're always short of time. It's always a problem." (GP-7) – Dew et al., 2018.

"It may also bring consequence to overcrowding in puskesmas due to an increased number of daily visits by patients. The excessive workload for all health professionals is inevitable with a pharmacist is arguably worst affected by this situation. Only less than half of puskesmas (4,986 puskesmas) are equipped with pharmacist reflecting that there are still major imbalances. and gaps in the availability and distribution of pharmacist across puskesmas. The high workload experienced by primary care pharmacists has been evident, often due to understaffing, and the need to deal with many administrative and technical tasks." – Hermansyah et al., 2020.

"Pharmacists frequently mentioned having insufficient time to balance dispensing duties alongside extended services. They acknowledged the need for community pharmacies to employ sufficient

pharmacy staff to be able to manage and distribute responsibilities to provide a range of extended services. At times, pharmacists were reluctant to approach patients/provide extended services due to high workload which was mainly linked to dispensing duties." – Hindi et al., 2018.

"There's just not enough pharmacist hours out on to do everything that pharmacists need to do… because we are busy you kind of don't drive it as much as you could (Pharmacist, Evans et al., 2016)." – Hindi et al., 2018.

"We are trying to get together to build up a collaborative relationship. But to date, 8 months have passed and there was no possibility to come together. It's difficult (FG3, CP3).'' – Weissenborn et al., 2017.

"National legislation regarding pharmacy supervision, coupled with long opening hours, made it difficult to establish face to face contact with other healthcare professionals. As a result, interaction with GPs had been reactive: "...sometimes it gets as late as half seven and we close. So you can't really go [to meetings with GPs] after work, can't go before work because we open at 8.45 and we don't close for lunch..."10M -- Sokhi et al. 2020.

"All groups noted that pharmacists were under a great deal of time pressure. As one patient noted: Patient: '. . . [pharmacists are] under even more pressure because the huge line up [of patients] there . . . the phones are jangling, ringing all the time, they're even under more pressure' (FG5:5)." - Tarn et al., 2012.

"Several physicians also recognized the problem, and one commented: Physician: '. . . I think a lot of people just feel like there is a big line behind them and they don't want to hold up the line [to receive medication counselling], several people just say no, that's okay.' (FG1:1)." - Tarn et al., 2012.

Knowledge sharing

11. Digital integration

When GPs and CPs share information and electronic records with each other for patient care (C), this may avoid duplication of work/effort, eliminate gaps, and facilitate shared decision-making and collaboration (O) because they can develop a common understanding of issues and patient care needs (M).

"To avoid duplication of work and to strengthen communication, shared care plans were recommended with read/write access to care records. The importance of good safeguarding measures was stressed." – Brewster et al., 2020.

"This finding reiterates the importance of accurate, up-to-date immunization registries and the need for development of a shared electronic medical record that provides access across practice sites and types." – Teeter et al., 2021.

"The International Pharmaceutical Federation envisages a future of common patient databases and shared care protocols across care settings, developed collaboratively and based on best evidence. Digital integration is important for this and for pharmacy services to become better embedded into healthcare models but is still limited in most countries." – Brewster et al., 2020.

"Standardized working methods as well as standardized communication channels facilitated collaboration." – Rubio-Valera et al., 2012.

"One of the things that we had is that we phoned the health centre to have direct access to the doctor's surgery. We had the switchboard number… each doctor had a switchboard number and we dialled it. I mean we went in directly. This meant that any problem we had could be solved straight away. [CP6: CP W BCN]." – Rubio-Valera et al., 2012.

"Having a good relationship with the local GP …I think to make referrals easier, to feel like you're part of the team, dealing with it, would obviously help. (Pharmacist, Horsfield et al., 2011)." – Hindi et al., 2018.

"I've worked in practices where there's very often been a pharmacist like you who you get to know personally… The problem is when requests are coming for prescriptions to pay back tablets that have been lent out from a chemist that you're not really that familiar with, and we start to wonder about what the patient's up to. (GP, Morecroft et al., 2015)." – Hindi et al., 2018.
co
"Some GPs stated they would like to receive more information from CPs about their patients' adherence because CPs see the patients more often and could much easier identify whether the patients handle their medication incorrectly (e.g. FG2, GP2: ''One of my patients just bought the medication that I prescribed him to do me a favour. But he did not take it. Seriously!'')." – Weissenborn et al., 2017.

"We have had chats about 15 or 20 patients in whom we weren't aware of side effects, or poor compliance, or we weren't aware of them not having had their cholesterol checked etc. Some minor points, but when they add up in those sorts of numbers, they become significant. The patients have told me that they really appreciated someone actually giving them sound advice about what the tablets do, how to solve problems with minor side effects, whether there were any alternative medicines they could try - in fact he [CP] picked up a couple of people who unfortunately had impotence with their drug therapy and we didn't know - he by the way gave them very good advice. We changed one person's medication, and we referred one for special counselling and treatment." – Woolley & Cantrill, 2000.

12. Pre-existing relationships

When GPs and CPs do not have an established trusting working relationship (e.g., when either party could be locums) (C), they are more likely to limit sharing information and/or electronic records with each other (O) because of confidentiality or safety concerns (M).

"GP concerns about confidentiality and information sharing with pharmacists was mentioned as a barrier to gaining access: …that practice in the beginning wouldn't let [the pharmacist] access any medical records because they felt that it was a confidentiality issue… pharmacists are bound by the same confidentiality clauses as GPs and nurses or anyone else and I don't think that's understood in primary care at all… (Site E, PCT Pharmaceutical adviser)." – Bradley et al., 2008.

"The extent of accessibility of both GPs and pharmacists were highlighted as barriers to GPs and pharmacists working together. Nearly all respondents cited a lack of time as the main reason for not working together with their GP or pharmacist colleagues. Indeed, several pharmacists noted that GPs were not always easily accessible. In contrast, GPs, and pharmacists whose counterparts were easily accessible found it easier to collaborate and develop positive interprofessional relationships: 'You tend to make better relationships with the ones you interact more with and the [more physically] closer you are to them the easier it is to see and interact with them.' (GP06)" - Van et al., 2011.

"The nature and extent of interactions between GP–pharmacist pairs were often seen to be unique to that particular pair. A single GP may have very little contact with one pharmacist but a lot of contact with another and vice versa. It was reported by both GPs and pharmacists that having regular contact within a GP–pharmacist pair was more likely due to the pharmacist taking the initiative to make the first contact, establish a pattern of communication suitable to both parties and maintain the relationship on an ongoing basis. An established pattern of communication conducted on a regular basis facilitated not only routine interactions relating to dispensing but also collaborative interactions, such as the sharing of information and discussions relating to patient care." - Van et al., 2011.

"Despite the possible threats, GPs thought they would be more accepting of the services if they knew the pharmacist well, or if they already had a good working relationship with them. GPs felt that it was essential for them to trust the pharmacist in order to accept their new services." – Hatah et al., 2013.

"Obviously sometimes we don't make a careful decision… So, I think that's a more difficult area, because I think that's almost where you actually need a pharmacy facilitator on site, or at least in close relationship. I'd probably accept it from her [local pharmacist] because I speak to her frequently and know her. I think it's to do with the relationship. (GP#1, G3)." – Hatah et al., 2013.

"Another possible threat to patient care voiced was that pharmacists were not trained in diagnosis and treatment. It was thought unsafe for pharmacists to give recommendations without appropriate skills and training, based on assumptions, and with little clinical information. The majority of GPs would agree to share clinical notes with pharmacists providing that patients gave consent. However, many GPs preferred the information to stay within the GP's surgery." – Hatah et al., 2013.

"I wouldn't be happy for it unless I had a close relationship with the pharmacist, and I trusted them to tell me if things are going wrong or if patients aren't picking up prescriptions… I think if the chemist didn't know the patients, didn't know the GP, it could be a very dangerous system indeed. (GP 11)." – Elvey et al., 2008.

"If they had more locums and things, I wouldn't trust the system so well because they wouldn't necessarily know the patients. That's another thing, the chemists have a relationship with the patient as well I believe. (GP 12)" – Elvey et al., 2008.

"GPs' reluctance to engage where they do not have an established working relationship with the local community pharmacist." – Elvey et al., 2008.

"This echoes previous findings that GPs' views on whether they would like a patient to seek advice from a pharmacist were influenced by whether or not they knew the pharmacist personally." – Elvey et al., 2008.

"Some of the pharmacists reported to have no good relationship with the GPs of their patients. This was considered an obstacle in conducting the intervention. Since good co-operation between pharmacists and GPs is of great importance for the optimal drug treatment of patients." - Adhien et al., 2013.

"GPs have also been reluctant to use services led by pharmacists they did not trust, respect or have confidence in, with regard to competence and quality of cognitive services." - Bryant et al., 2017.

"As with this study, others have also noted collaboration tended to be person-dependent and could fade away if a key pharmacist or GP left their position." – Bidwell & Thompson, 2015.

"A good working relationship with the local pharmacist(s) was sometimes considered a prerequisite to establishing repeat dispensing. Some GPs felt strongly that they would only operate repeat dispensing with pharmacists whom they knew and perceived it to be potentially unsafe where they did not know the pharmacist. Particularly serious doubts were expressed about patients collecting repeat prescriptions from pharmacies frequently staffed by locums, as it was feared the lack of continuity could be unsafe for patients." – Elvey et al., 2008.

"Both pharmacists and GPs believed that collaboration could be improved by enhancing their relationships." – Hindi et al., 2018.

"A substantial internal barrier mentioned by both professions in three out of four FGs was that GPs and CPs do not know each other in person. Hence, they might be unaware of their respective education, their fields of competence, their working conditions and/or statutory duties." – Weissenborn et al., 2017.

"It's a question of manners and of existing relationships… Trust is a premise, isn't it (GP)." – Weissenborn et al., 2017.

"In contrast both professions stated that knowing each other eases collaboration (FG1, GP1: ''I learned a lot about their profession, and I adopted a positive attitude towards them. I never had problems in cooperating with pharmacies.''). GPs mentioned they appreciate when CPs introduce themselves and both professions stated that long-term relationships are developed over years." – Weissenborn et al., 2017.

"All but one CP and one GP agreed that communication might be improved if they knew each other and that they would be willing to attend face-to-face meetings." – Weissenborn et al., 2017.

"Some respondents indicated they had good relations with their local doctors or pharmacists that were mediated by regular contact and were willing to collaborate with each other to improve patients' adherence to medications." - Rathbone et al., 2016.

"You do not know who you are dealing with, and you cannot build up a relationship with them [employee pharmacists] because they are forever changing.' [GP20]." – Hughes & McCann, 2013.

13. Integrated communication strategies

When CPs do not have an easy to use and reliable means of communicating with GPs and accessing elements of patient records (C), this may constrain the advice and management CPs can provide to patients (O) because they lack relevant information (M).

"[The medication review] is impossible to do without [access to records] . . . I mean, you can do some sort of review without it, but you've only got half the information . . . it's only when you come to look at their records that it's obvious why the patient's got these two things, that look bizarre to start with but actually when you take the chance to read through the notes it makes sense. So, you can't do it without it. (Site D, LPS Pharmacist)." – Bradley et al., 2008.

Many of the respondents agreed that there was a need for more communication between GPs and pharmacists. 'There just needs to be more communication between each of the caregivers. So, everyone knows what's going on with the patient.' (GP12) Generally, GPs who reported a high frequency of contact with pharmacists also tended to find input from the pharmacist more beneficial to their practice." - Van et al., 2007.

"Community pharmacy should also be enabled to 'write' in patient records so that any medication or advice given can be recorded to maintain continuity of care and ensure advice and treatment given in other healthcare settings (GP, hospital) can take general health, underlying conditions and medicines use into account." – Smith et al., 2016.

"Concerns appeared to have been further exacerbated by the apparent lack of a coherent and integrated communication strategy to promote the various changes to both professions. This latter point was commented on by almost all participants." – Bidwell & Thompson, 2015.

"This concern of GPs about fragmentation of care was even more pronounced in relation to other expansions of pharmacy into services such as INR testing, and providing influenza vaccines, although for some GPs, anything that increased influenza immunisation coverage was seen positively. There was a strong feeling that such functions were better to be kept within the medical centre, which had traditionally always provided these services. Even those who were the most positive about it would prefer the pharmacist to be within their team: The pharmacist should be as part of the team in this centre and not just randomly doing it because they don't have all the information potentially of what's going on with them [the patient]. (GP)." – Bidwell & Thompson, 2015.

"Current barriers to this model identified were: medico-legal issues, budgetary issues, limited access to patient data, lack of privacy to talk to patients, inadequate time and remuneration, no patient registration, patients describing vague symptoms and patient confidence in pharmacists." – Aradottir & Kinnear, 2008.

"The main barriers that hindered both HCP groups in ensuring that older patients received appropriate polypharmacy related to the current work environment ('Environmental context and resources'). For example, in addition to time and workload barriers, a lack of appropriate resources (e.g. staff) was also noted by both groups. Pharmacists raised other challenges that they encountered in practice (e.g. lack of direct communication network with GPs, lack of access to patients' clinical data, lack of availability of non-pharmacological treatment options)." – Cadogan et al., 2015.

"Pharmacists felt the advice they could give patients was limited by their lack of access to patient medical records, and that they could only advise depending on a patient's account of their TSH results and medications. Pharmacists felt that access to TSH results would save time and inaccuracies in advice they give:" – Dew et al., 2018.

"And that's something that, you know, it would really help us if we had access to things like TSH results and things. Just as a second check, I think. (P-2)." – Dew et al., 2018.

"The International Pharmaceutical Federation envisages a future of common patient databases and shared care protocols across care settings, developed collaboratively and based on best evidence. Digital integration is important for this and for pharmacy services to become better embedded into healthcare models but is still limited in most countries." – Brewster et al., 2020.

"Perceived pharmacists' weaknesses included a lack of knowledge about each patient's medical history and clinical conditions." – Hatah et al., 2013.

"It was important to share a clinical chart so that both professionals could have access to complete patient information. Standardized working methods as well as standardized communication channels facilitated collaboration." – Rubio-Valera et al., 2012.

"We feel we are out on a limb, and the only, first, contact we have is when we get a prescription through for a controlled drug (CD), and then we are left wondering what it's for. It's like doing a jigsaw and only having half the parts, and trying to make the picture, you know? (Pharmacist, Savage et al., 2013)." – Hindi et al., 2018.

"Pharmacists expressed frustration about not having enough information about a prescribed medication and about a patient's clinical situation to effectively counsel patients." - Tarn et al., 2012.

"I don't see how you could do it without clinical information… it just gently reminds you that you are doing the best to the best of your ability, but you are kind of working with your hands behind your back (CP) (Cardwell et al., 2018)."

"Improved communication and information sharing between community pharmacists and general practice could facilitate community pharmacist-role expansion." – Gidman & Cowley, 2013.

"Pharmacists do not have access to patients´ clinical background or medical records. They therefore often help patients based on little and incomplete information, often limited to what the patient tells them. This lack of information makes their job difficult." – Rakvaag et al., 2020.

Interprofessional relationships

14. Learning systems

Informal opportunities for reflection and dialogue between GP and CP (C) can support patient care (O) because it is easier to establish trust, communicate information effectively, and address clinical issues collaboratively (M).

"There's [sic] a few other doctors that we do chat on a regular basis to the point where they don't want us to call them doctors or by their surname. We have got each other's mobile phones [numbers]. It's to the point where they can call me on a Sunday night and can ask me for something or do a favour for them. That's the level it's gone to (CP)." – Rathbone et al., 2016.

"There was a lot of phone communication, but travelling both ways, him querying scripts and us asking for advice. I would often pop into the shop and have a chat with him. I suppose I would see him, talk to him, two or three times a week (GP)." – Bradley et al., 2012.

"I got the satisfaction of running my recommendations past the doctor not just sending him the report and getting a thank you back… I actually learnt a lot from that doctor because he was able to say, 'that's not practical' or 'that's a good idea'… (CP)." – Disalvo et al., 2019.

"GP–pharmacist group meetings may be hard to arrange in rural pharmacy depending on where it is organised due to restriction on travel/distance (CP-222)." – Saha et al., 2021.

"Legislative and geographical factors had the potential to increase the effort required to coordinate collaboration, which in turn also affected the professionals' attitudes." – Rubio-Valera et al., 2012.

"Professionals felt that working together was easier in small areas or when pharmacists in the area worked together and coordinated the delivery of services between them." – Rubio-Valera et al., 2012.

"What happens is that I have the advantage that I am in a basic unit where I have a single reference pharmacy. So, of course, there is only one pharmacy with which I have regular contact… which is a very big advantage. [GP17: General practitioner with experience from Mallorca (GP W MLL)]." – Rubio-Valera et al., 2012.

"Location and proximity may be associated with a higher frequency of interaction. One study in Wales found that the frequency of interaction with GPs was significantly higher (a two-fold increase) for pharmacies co-located with general practice (which made up 17% of their sample). This increase could not however be attributed to a greater level of face-to-face contact due to co-location, as the method of contact was not captured in this study (Jenkins et al. 2016)." – Bradley et al., 2018.

"GPs working in closer proximity to their pharmacist counterparts were also found to have higher levels of collaboration than isolated practitioners. This may be because being geographically closer to one another provides more opportunity to develop rapport and positive relationships as a result of increased interaction." – Van et al., 2013.

"GPs report more distrust of pharmacists practicing in pharmacy chains, and many GPs preferred an interprofessional model that involves pharmacists located within their practices and working directly with GPs." – Bryant et al., 2017.

"Both GP and pharmacist respondents felt regular communication that focused on clinical issues engaged both parties in professional dialogue and helped build rapport and trust. In turn rapport and trust lead to open, honest and bilateral communication between GPs and pharmacists helping them work together more effectively: 'Constant communication is the thing that probably helps the most. The doctors that we talk to the most are the ones that give the most feedback and are more likely to respond to requests either by accepting requests or telling us why they don't want to. I feel that is just because we already have some dialogue happening.' (P04)" - Van et al., 2011.

"The extent of accessibility of both GPs and pharmacists were highlighted as barriers to GPs and pharmacists working together. Nearly all respondents cited a lack of time as the main reason for not working together with their GP or pharmacist colleagues. Indeed, several pharmacists noted that GPs were not always easily accessible. In contrast, GPs, and pharmacists whose counterparts were easily accessible found it easier to collaborate and develop positive interprofessional relationships: 'You tend to make better relationships with the ones you interact more with and the [more physically] closer you are to them the easier it is to see and interact with them.' (GP06)"- Van et al., 2011.

"When proximity to GP's office' was modelled as a predictor of collaboration using Sample 1 data, those pharmacists working in closer proximity to their GP counterparts were found to have higher levels of collaboration than isolated practitioners. This may be because being geographically closer to one another provides more opportunity to develop rapport and positive relationships as a result of increased interaction. However, when 'proximity to GP's office' was modelled and tested on the validation sample (Sample 2), the impact of this variable was not replicated. As this variable has been identified as important in previous studies [15,27], and significantly impacted on collaboration when modelled using Sample 1 data, it should not be disregarded but may be worth investigating in future research." - Van et al., 2012.

“Telephone communication seemed to be the most convenient mode of collaboration. ‘I think the best way is by phoning us that’s the quickest... but also fax would be alright if there’s non-urgent things.’ (GP11). Many of the respondents agreed that there was a need for more communication between GPs and pharmacists. ‘There just needs to be more communication between each of the caregivers. So everyone knows what’s going on with the patient.’ (GP12) Generally, GPs who reported a high frequency of contact with pharmacists also tended to find input from the pharmacist more beneficial to their practice.” - Van et al., 2007.

“Where there was a long-standing and more personal relationship between a particular pharmacist and GP practice it appeared that an efficient arrangement had been worked out to minimise the burden on both, as both GPs and pharmacists dealt with many different pharmacies and practices. However, this was not possible in all cases. Moreover, any change in staff, either in the pharmacy or the practice, could mean that relationships had to be developed anew.” – Bidwell & Thompson, 2015.

“The greatest acceptance and support for collaboration in Britain has been in situations where a pharmacist has been fully integrated into the healthcare team and available for consultation by the GPs or assistance to patients as required. Although the employment models for GPs and pharmacists in New Zealand are different from those in the UK, some participants in this study also believed that this type of arrangement was the ideal solution. There are emerging examples of co-location and pharmacists being integrated into existing practice teams on a part-time basis.” – Bidwell & Thompson, 2015.

“GPs and CPs do not know each other in person. Hence, they might be unaware of their respective education, their fields of competence, their working conditions and/or statutory duties. In contrast both professions stated that knowing each other eases collaboration (FG1, GP1:‘‘I learned a lot about their profession and I adopted a positive attitude towards them. I never had problems in cooperating with pharmacies.’’). GPs mentioned they appreciate when CPs introduce themselves and both professions stated that long-term relationships are developed over years. Several participants reported sympathy, trust, and self-motivation as a premise for good communication. Both professions reported that GPs sometimes feel criticized when receiving information or support from CP.” - Weissenborn et al., 2017.

‘‘Internal barriers to communication: “After 15 years I met the pharmacist who owned the pharmacy 500 m away from my general practice for the first time [...]. We talked and it indeed was very positive (FG2, GP1)!’’ ‘‘I agree that we know each other too little [...]. Because if we knew each other, then maybe we would be able to take some things better (FG3, CP1).’’ ‘‘If there is a new GP in my neighbourhood, I make an appointment and then I introduce myself so that the general practice knows who we are when we are calling. I think this custom is getting lost—I am always surprised when a GP answers, ‘I appreciate your visit, so far none of your colleagues stopped by’ (IDI, CP8).’’” - Weissenborn et al., 2017.

“External factors were, among others, increasing bureaucratic demands, a lack of time, long distances between GPs and CPs, and poor availability (e.g. because of patients requiring counselling, varying opening hours, and GPs making home visits).” – Weissenborn et al., 2017.

‘‘If there is a new GP in my neighbourhood, I make an appointment and then I introduce myself so that the general practice knows who we are when we are calling. I think this custom is getting lost—I am always surprised when a GP answers, ‘I appreciate your visit, so far none of your colleagues stopped by’ (IDI, CP8).’’ – Weissenborn et al., 2017.

“National legislation regarding pharmacy supervision, coupled with long opening hours, made it difficult to establish face to face contact with other healthcare professionals. As a result, interaction with GPs had been reactive: “...sometimes it gets as late as half seven and we close. So you can't really go [to meetings with GPs] after work, can't go before work because we open at 8.45 and we don't close for lunch...”10M - Sokhi et al. 2020.

“There’s [sic] a few other doctors that we do chat on a regular basis to the point where they don’t want us to call them doctors or by their surname. We have got each other’s mobile phones [numbers]. It’s to the point where they can call me on a Sunday night and can ask me for something or do a favour for them. That’s the level it’s gone to (CP).” - Rathbone et al., 2016.

15. Role clarity

When GPs and CPs participate in interprofessional activities (e.g., education, joint service initiatives, quality circles, case conferencing, and face-to-face meetings) that enable them to safely and interactively learn with, from, and about each other (C), they develop knowledge and clarity about their respective roles and capabilities (O), because their misconceptions are challenged (M).

“The main advantage when collaboration is established is that it breaks a series of stereotypes that exist from the doctor towards the pharmacist, that there is intrusiveness, this or that… and the opposite, from the pharmacist towards the doctors, that they are arrogant, that they do this or that, all these things stop when two professionals with similar knowledge, or even a similar age, see each other, a lot of barriers are broken. [CP16: Community pharmacists with experience from Mallorca (CP W MLL)].” – Rubio-Valera et al., 2012.

“However, the view about the other profession changed when practitioners knew each other. Stigmatized views and conflicts were resolved.” – Rubio-Valera et al., 2012.

“They noted that perspectives from all disciplines were now incorporated in decisions about caring for individual patients and that good teamwork had resulted in a more consistent approach to patient care. Group discussions in a relaxed atmosphere meant that collective decisions were carried through into practice change, further reinforcing relationships and teamwork.” – Bidwell et al., 2017.

“One of the things that the pharmacies have really appreciated is having these meetings (monthly meetings in the PCHC with GPs and CPs from the area) and so the thing is, since we started this, they have improved their relationship with each other (. . .) each year, they have a lunch, a ‘‘gathering’’, them, all of them, the pharmacy managers, but in reality they have been doing it since they started to meet each other here with us. (GP with collaboration experience).” – Jove et al., 2014.

"This finding corroborates results of previous studies that have found that role recognition shapes interactions and successful teams recognize the professional contributions of its members. Other studies emphasize the importance of precisely defining pharmacist responsibilities as GPs are typically conservative in their view of the pharmacist's role." - Van et al., 2013.

"The item that made up contact with pharmacists during formative years asked respondents whether during their residency, they rarely, occasionally, or frequently had contact with pharmacists regarding drug therapy. Those GPs who had frequent contact with pharmacists during their residency were more likely to have higher levels of collaboration with their pharmacist counterpart in their current practice. This may be due to two reasons. Firstly, it may indicate that physicians who are more likely to have or seek contact with pharmacists during their residency are also more likely to collaborate with pharmacists later on. Secondly, it may be because an effective time to expose physicians to pharmacists and raise awareness of the potential contributions a pharmacist can make to medication management is during a physician's formative years." - Van et al., 2013.

"Physician contact during training' was found to be a predictor of collaboration… Those pharmacists who had frequent contact with GPs and/or medical officers during their pre-registration training were more likely to have higher levels of collaboration with their GP counterpart in their current practice… Exposure to collaboration during the final year of pharmacist training equips pharmacists with the skills and confidence for future collaboration." - Van et al., 2012.

"Many respondents agreed that the presence of a set protocol with well-defined roles for the GP and pharmacist provided a good framework for teamwork. HMRs [home medicine reviews] often brought GPs and pharmacists together and provided a platform for collaboration: 'It's improved relationships . . . [it answers to an extent] what is the pharmacist expected to do, what is the GPs role' (GP05)" - Van et al., 2011.

Interprofessional education events were also seen as helpful in bringing together GPs and pharmacists. It afforded both professions an opportunity for practitioners to meet face-to-face (something quite rare in practice) and to explore each other's perspectives on medication and patient management. This often facilitated the development of shared understandings: 'Combined education certainly helps [foster collaboration] . . . it's a good environment, good atmosphere and everyone gradually realises that each group has their own specialty' (P04)." - Van et al., 2011.

Practitioners who were exposed to interprofessional learning modules, for example, during their education and training were more likely to have a positive impression and be more willing team players:' I think the older doctors tend to have less interaction with pharmacists . . . the younger doctors, the newer generation, are taught to interact with other HCPs and so there's more teamwork involved.' (P01) Similarly, several respondents believed that being exposed to interprofessional teams in hospitals or nursing homes not only allowed them to appreciate the benefits of teamwork but also equipped them with skills to work effectively with their GP counterpart in community practice: 'When I was working in a hospital it was a team environment . . .[so] you get used to working as a team' (P07)" - Van et al., 2011.

"This study has several important implications for practice and highlights possible strategies for improving GP-pharmacist interprofessional collaboration. Whether the GP saw a role for the

pharmacist in medication safety and effectiveness was the variable found to be the strongest predictor in our model. Collaboration will improve as GPs become more aware and accepting of the pharmacist's role in medication management. One such strategy to foster this may be to create opportunities for GPs to interact more with pharmacists on a professional level to gain a better understanding of the pharmacist's potential contribution to patient care, for example via co-location or interprofessional education. Creating more opportunities for GPs to interact with pharmacists in their formative years may be an effective way to promote acceptance and appreciation for the pharmacist's role. Professional pharmacy services such as HMR are also helpful as they provide a formal platform for GP-pharmacist interaction and clearly highlight the contribution and responsibilities of each partner" - Van et al., 2013.

"Also, the GPs and CPs believed that collaboration facilitated the definition of the professional roles which could prevent conflicts between the GPs and the CPs." – Jove et al., 2014.

"They noted that perspectives from all disciplines were now incorporated in decisions about caring for individual patients and that good teamwork had resulted in a more consistent approach to patient care. Group discussions in a relaxed atmosphere meant that collective decisions were carried through into practice change, further reinforcing relationships and teamwork." – Bidwell et al., 2017.

"All respondents agreed that the multidisciplinary group had improved their access to professional development and their incentive to attend. Moreover, it provided a forum for staff to meet together even if their working hours did not coincide:

We don't have travel time or costs of going elsewhere. As a group, we are able to have a more relaxed discussion afterwards. It forms a good opportunity to get together outside of work hours, reinforcing the team approach of our practice. This is particularly of benefit as we all work part time and don't necessarily see each other (Participant 2)." – Bidwell et al., 2017.

"GPs and CPs felt that meetings needed to be held regularly so that professionals could discuss shared objectives." – Rubio-Valera et al., 2012. (Meetings held at lunch times). "While this is not an ideal arrangement, the option of combining lunch with meetings is the most practical solution in a country where the midday meal is a social occasion and many business premises, including pharmacies, close between 2-5 pm." – Rubio-Valera et al., 2012.

"All the pharmacists were comfortable with the prospect of extending their role into more direct patient management. They saw the management of angina as no more or less threatening than that of any other chronic stable condition managed largely by drug therapy. The predominant view was that they were ready for this type of "hands on" challenge, which they saw as a natural and necessary extension to their evolving role. This clear vision was shared by pharmacists with little experience of working in the community pharmacy setting and also those with many years of experience working in the community pharmacy setting: "This might seem pretty revolutionary, but no I think it's about time we got into the surgeries and got involved. After all we are the drugs experts, we have years of experience there and added to that we have acquired expertise in the area of improving lifestyles, smoking cessation and that sort of thing." (Pharmacist 1)" - Woolley & Cantrill 2000

"The same pharmacist spoke of his improved understanding of the day-to-day problems faced by GPs when providing this type of preventive care: "Personally I think I have much more of a feel for the type of work the GP does - and what sorts of pressures they have to deal with and how to overcome them." (Pharmacist 2)" - Woolley & Cantrill 2000.

"Most of the pharmacists described a closer, more constructive, and professionally satisfying relationship with the GPs, which they attributed to participating in the study. This extended beyond the realm of angina management. Some of the comments made in this respect clearly reveal the emergence of a pharmaceutical care role: "I didn't know the GPs before I started the study, but I soon got to know them and once I had suggested a few drug changes, not just the anginal drugs but others as well, they started to ask my advice, as did the other practice staff." (Pharmacist 3) Another pharmacist said: "I have come across things in the notes occasional hospital letters that had not been acted on - sometimes drugs not put on to the repeat prescription, substitutions not made, drugs not crossed off. I think that both my practices have benefited, and I know that here the doctors would like me to stay on and do some prescription reviews." (Pharmacist 2)" - Woolley & Cantrill 2000.

"Some GPs made "uneasy" comparisons between themselves and the pharmacists, with respect to the management of their patients with stable angina. Two GPs suggested that because the pharmacists had performed so well, they became acutely aware of their own shortcomings. One said: "I have to gird my loins better, pull my socks up now, because well it's a bit discomforting when you realise that there are problems out there and you are not aware of them - when you think you are doing a good job - as best you can given the constraints of time and appointments. But he gave you that extra look at things so that things were changed for the better and for the patient's benefit." (GP 1)

Another GP identified a need for change in his approach to practice: "I realise now, and this is partly because of the pharmacist, that it would be better to see the patients for 10 minutes or so three or four times a year instead of for five minutes six times a year. Tn that 10 minutes I could do a lot more. With these sorts of patients well it's not just a question of a quick blood pressure and a quick blood test, but you have to look at the other risk factors as well and we haven't been doing that. That's not just me that's general practice overall. I thought we were doing a fairly keen job, but I realise it's time to change." (GP 3)" - Woolley & Cantrill 2000.

"However, participants expressed general support for the extension of the pharmacists' role, as long as each profession's responsibilities were clearly defined." - Deslandes & Foster, 2011.

"This is a small-scale exploratory study which examined the views of GPs on the community pharmacist's role. Previous research has highlighted that doctors may fear for their autonomy if the pharmacy profession continues to expand. Support was expressed, however, with the caveat for clear boundaries of responsibilities within the health care team and increased awareness of the pharmacist's role." - Deslandes & Foster, 2011.

"First of all, GPs and CPs should be supported to get to know to each other. This may be achieved by either interprofessional quality circles (an interdisciplinary group that meets to discuss work related problems or to train each other) or personal face-to-face meetings. As early as at university collaborative teaching sessions could be initiated." – Weissenborn et al., 2017.

"Several professionals have already given attention to interprofessional education and some research projects regarding collaborative teaching sessions have been conducted. Interprofessional education might improve team working skills [37] and foster positive attitudes, respect, and working relationships between the healthcare providers [36–41], e.g., by knowing and valuing their mutual skills and responsibilities." – Weissenborn et al., 2017.

"Knowing each other is a premise for sympathy and trust and one GP stated that he received a better understanding of CPs' obligations after having talked to him. Therefore, the concept should encourage GPs and CPs to deal with each other and to make a first move towards the respective profession. Moreover, information about both professions' working conditions and statutory duties should be provided to establish better mutual understanding." – Weissenborn et al., 2017.

"Although several pharmacists already had good working relationships with GPs, CPAMS strengthened many of these existing relationships." – Egunsola et al., 2022.

"GPs and patients also developed a better understanding of the value that pharmacists provide to patients." – Egunsola et al., 2022.

"Joint training at undergraduate and postgraduate level of the two professions was suggested by pharmacists as a way to overcome barriers and increase awareness of professional skills and strengths:

"We need to work together; we have to start working as teams. We really do have to break down those barriers, we really should start our training together. The first year of pharmacy and medicine should be the same, the same as medicine and dentistry. So that you know those people, have the same training and same background knowledge.' [P9]." – Hughes & McCann, 2013.

16. Synergistic combination of skills, knowledge, and resources

When CPs and GPs have opportunities to participate in multi-disciplinary training and joint service initiatives (C), they are better at collaborating together for the benefit of the patient (O), because they have learned how to work together by capitalising on their respective capabilities, knowledge, and unique skills.

"I don't think pharmacology is a strong point with GPs. I don't think it's well taught … in med schools. So having feedback about any potential [interactions]… will be really fortunate. (GP#10, G1) [Speaking about Clinical Medication Review]." – Hatah et al., 2013.

"GPs thought that pharmacists' strengths were their knowledge of pharmacology and drug interactions, and a better knowledge about aspects, such as dosage forms and the appearance of medications. GPs also thought pharmacists had a better knowledge of herbal and alternative preparations than GPs. Another strength perceived was that pharmacists would have a longer

consultation time if funded by the health system to talk about medication use with patients." - Hatah et al., 2013.

"I don't think it's possible for us to have some of the skill set that the pharmacists do. Pharmacists have specialist knowledge in some aspects of medication, or you might say all aspects… so if somehow combining the two so that we work together and utilise everybody's skill set… (GP#11, G1)." - Hatah et al., 2013.

"We find we're working quite well because we find that each professional has their expertise and their area that they're not familiar with, but by working together we can sort of iron out all the sort of disadvantage. (Site F, LPS Pharmacist)." – Bradley et al., 2008.

"They [pharmacists] are an invaluable back-up because GPs do make prescribing errors and to know it's a blanket that… if you write something stupid there's somebody else checking it… that they make sure that no harm comes to the patient. (GP#10, G1) [Speaking about Clinical Medication Review." – Hatah et al., 2013.

"Sharing responsibilities and working together have a positive impact on the quality of drug therapy, avoid adverse drug-related incidents, and are economically efficient as they reduce the number of patient visits to doctors." – Waszyk-Nowaczyk et al., 2021.

"For patients, effective collaboration between the healthcare team can lead to better coordination with healthcare professionals and more effective treatment." - Waszyk-Nowaczyk et al., 2021.

"Both professions showed good agreement on pharmacists' roles related to managing side effects, improving adherence, assisting in dosage adjustment, providing advice regarding drug interactions, and providing drug information to physicians." – Albassam et al., 2020.

"They (pharmacists) deal better with banal pathologies (mild and/or self-limiting diseases), for instance, lice in children, in treatments with dietary supplements, vitamin supplements, etc. that sometimes we don't know about whether it's because of training because the information doesn't reach us, and they know more and many times they explain things to us. (GP with collaboration experience)." – Jove et al., 2014.

"Many respondents agreed that the presence of a set protocol with well-defined roles for the GP and pharmacist provided a good framework for teamwork. HMRs often brought GPs and pharmacists together and provided a platform for collaboration: 'It's improved relationships . . . [it answers to an extent] what is the pharmacist expected to do, what is the GPs role' (GP05)" - Van et al., 2011.

"GP records state what was prescribed, provided it was entered electronically, while pharmacist records state what was actually collected. Therefore, linking the community pharmacy and GP databases enables a more accurate and complete picture of drug utilization to be obtained. The prescriber will have a more accurate picture of patient's medicine-taking behaviour available to them when authorizing new prescriptions and considering increasing dosages in light of apparent therapeutic failure, which may actually be non-adherence. The pharmacist will additionally be able to confirm whether prescriptions have been authorized and at what dose and use this information to support patients who present without their medicines requiring additional supplies in an emergency." - Wright & Twigg, 2016.

"Pharmacy staff also felt they could leverage their existing relationships with local clinics with one pharmacist stating, "I think it's an excellent idea. I have no problem reaching out to those physicians. I know those clinics and talk to the nurses and doctors… And most of them, they've quit giving all those shots. They're kind of referring them all out… that's what I've seen." – Teeter et al., 2021.

"To improve AR management within the community pharmacy setting, the pharmacists recommended collaborative AR training with GPs to ensure a consistent message is delivered to AR sufferers that is consistent with ARIA guidelines, incorporating these values into an integrated care approach." - Cvetkovski et al., 2020.

"The pharmacists stated that if their neighbouring GP colleagues were provided with the same training at the same time, it would ensure a consistent message was delivered to their common patient community and would re-enforce the recommended AR management pathways." - Cvetkovski et al., 2020.

"Interprofessional education events were also seen as helpful in bringing together GPs and pharmacists. It afforded both professions an opportunity for practitioners to meet face-to-face (something quite rare in practice) and to explore each other's perspectives on medication and patient management. This often facilitated the development of shared understandings: 'Combined education certainly helps [foster collaboration] . . . it's a good environment, good atmosphere and everyone gradually realises that each group has their own specialty' (P04)" - Van et al., 2011.

"Some said they appreciated being contacted by pharmacists about potential interactions or safety issues and relied on pharmacists to be a back-stop for them in identifying inadvertent errors that came about through the drop-down menus in their prescribing software for example. Pharmacists were also highly valued in formulating medicines for patients who could not or would not take tablets and providing up-to-date advice on new medicines." – Bidwell & Thompson, 2015.

"… pharmacology has just got so complicated that a good pharmacist is worth his weight in gold in peer support (GP)." – Bidwell & Thompson, 2015.

"When looking ahead, most GPs had some positive comments about how relations between the two professions might change and develop. They recognised the expertise that pharmacists had and most welcomed the current approach that focused on a greater role for pharmacists in providing advice, particularly on medicines, to patients. Many were highly supportive of pharmacists' efforts to encourage compliance and see that patients used their medicines properly. Some would have liked to have a pharmacist working within their practice." – Bidwell & Thompson, 2015.

"Some GPs expressed surprise at the pharmacists' level of knowledge and skills, suggesting a lack of recognition for the professional capabilities of pharmacists. GPs at three sites reported that a major benefit of working alongside their LPS pharmacist was utilizing the pharmacists' specialist knowledge. There was also evidence that through LPS, GP opinions about the role of pharmacists was changing." – Bradley et al., 2008.

"I've never made so much use of a pharmacist and found the pharmacist so helpful in providing care. (Site F, GP)." – Bradley et al., 2008.

"Well, we do [conduct medication reviews] but not in quite the formalised intelligent way that [the pharmacist is] doing it. (Site D, GP)." – Bradley et al., 2008.

"GPs considered their own strengths to be their skills and training in diagnosis and treatment, and their knowledge of clinical conditions. They thought they knew their patients' clinical conditions well and understood the usual processes of care. Another perceived strength was GPs' ability to practise according to guidelines." – Hatah et al., 2013.

"Part of my training is to use all that I have… the talking, the examination, the history to formulate what's going on. I'm not convinced that pharmacists or nurses have that background. (GP#6, G2)." – Hatah et al., 2013.

"GPs mentioned some opportunities for pharmacist contributions. GPs thought that pharmacists' greater knowledge of pharmacology and dosage forms, adverse drug effects and drug interactions would be an asset in helping ensure patient safety. They thought that a clinical medication review by pharmacists might reduce prescribing errors and provide reassurance of correct prescribing. Pharmacists' up-to-date knowledge of pharmacology was also thought an advantage as they could help to optimise patients' medication and be a source of medications information for GPs." – Hatah et al., 2013.

"It will be quite good to have a community pharmacist that visits GP practices and gives some input into some of the complicated drug regimen patients. Not necessarily just from the local pharmacy, but maybe someone that is more clinically orientated and has experience in that area... you know [see the] polypharmacy type of patients. (GP#3, G2) [Speaking about Clinical Medication Review]." – Hatah et al., 2013.

"Well, my recollection [for MUR] is that there were one or two things that the pharmacist pointed out that could be changed for the patient's benefit. (GP#3, G3) [Speaking about MUR]." – Hatah et al., 2013.

"Many GPs thought pharmacists might have a limited role in prescribing. Prescribing under agreed protocols or guidelines (for a limited number of diseases and conditions), with appropriate training and close monitoring was more acceptable to GPs. In addition, pharmacist prescribing for patients with warfarin was perceived as potentially beneficial by some as it could reduce GPs' and nurses' workloads. However, GPs would prefer this to happen within the GP's practice, as it could improve communication and prevent information being 'lost in transfer'. It would also allow GPs to have more control over decisions on patients' medications." – Hatah et al., 2013.

"The most obvious example is the relationship with the Pharmacy. The input they provide in conjunction with the knowledge we have of patients have allowed excellent patient centred decisions to be made (Participant 6)." – Bidwell et al., 2017.

"Thus, effective communication and collaboration between general practitioners (GPs) and community pharmacists (CPs) could potentially increase medicines optimisation and enhance patient safety." – Bradley et al., 2018.

"Measures also need to be put in place to facilitate improved communication and collaboration with other healthcare professionals and services, so that pharmacists can offer a synergistic role, becoming

more fully integrated and equipped to facilitate a more responsive and flexible healthcare system." – Brewster et al., 2020.

"There is consistency across studies showing that community pharmacy interventions are capable of offering an additional strategy and skill set in the delivery of diabetes care which often leads to improved healthcare outcomes for those enrolled." – Brewster et al., 2020.

"Considerable cost savings can be achieved globally, across a breadth of settings by increased delivery of additional services in community pharmacy." – Brewster et al., 2020.

"The main values added included freeing up GP time and easier access for patients." – Brewster et al., 2020.

"Greater collaborative working between GPs and local community pharmacists could achieve better understanding of pharmacy services in general and more realistic expectations in terms of their delivery, however, incentives are needed to achieve this goal." Watson et al., 2020.

"The pharmacist's role was perceived to be important to patient care and to the GP's role. Benefits included ensuring patient safety whilst dispensing medication, prescribing advice and referral to the GP." - Deslandes & Frazer, 2011.

"Dr 6 'community pharmacists in my own opinion are an essential part of the primary health care team.' - Deslandes & Frazer, 2011.

"Government reports, which advocate multidisciplinary team working in order to make full use of the skills and competencies of the various health care professionals." – Woolley & Cantrill, 2000.

The same pharmacist spoke of his improved understanding of the day-to-day problems faced by GPs when providing this type of preventive are: "Personally I think I have much more of a feel for the type of work the GP does - and what sorts of pressures they have to deal with and how to overcome them" (Pharmacist 2)." – Woolley & Cantrill, 2000.

All the pharmacists expressed a desire to continue with their "new role." One pharmacist said: "It's been hard work, especially at the start, but it gave me an incredible buzz, playing a complementary role to the doctors and the practice nurses and I would really like to do more of this type of work." (Pharmacist 5)." – Woolley & Cantrill, 2000.

"For the group of patients, he [the pharmacist] saw, I feel it has been quite a significant improvement in their care, not only from a pharmacy viewpoint which you could expect, but also spending time with them [the patients] discussing their problems and how to overcome them through helping themselves. He has been able to point them in the right direction - to discuss everything with them that we really needed to tell them about their condition." (GP 2). - Woolley & Cantrill, 2000.

"At the end of the clinics we would sit down with the notes and there were quite a few, I would probably say maybe 50 per cent of the cases, where we have done something, a pharmacological change, or maybe another referral or some investigation or test. It picked up a few things that we were doing wrong as doctors, inappropriate prescribing - wrong drugs where better alternatives could have

been used, or the wrong strength, the wrong dose, the wrong mechanism of action and he even picked on one or two that were not on aspirin.” (GP 1). – Woolley & Cantrill, 2000.

“Although the GPs indicated a high regard for the pharmacists’ input as the “experts on drugs” they were also aware of their ability to relate to the patients as individuals experiencing difficulties in coming to terms with the need to accept and modify risky lifestyles. One GP said:

“He had a very easy down to earth approach - not like us, too little time and too harsh – no he started slowly and just kept on encouraging them. I think he even got a few to give up smoking.” (GP3) – Woolley & Cantrill, 2000.

“Many of the GPs articulated a desire to continue working with the pharmacists and suggested other areas of disease management in which they could become involved. Most of these areas comprised one or more aspects of cost reduction. One said:

“There is no doubt he could become a member of the team here. I mean there is obviously a lot more work we can do together… I mean big areas like gastrointestinal disease, well that’s a massive area. I could see him [the pharmacist] interviewing these patients, getting their prescriptions sorted out, telling them what their drugs are for, perhaps even converting them to cheaper therapy.” (GP 7). - Woolley & Cantrill, 2000.

“The qualitative findings illustrate with remarkable clarity that the participant GPs and pharmacists were able to work together to deliver a specified programme of secondary prevention for patients with stable IHD.” - Woolley & Cantrill, 2000.

“Both professional groups viewed the experience as a positive one, which met or exceeded their expectations and one which was reported to have influenced their subsequent approach towards, and style of management of, patients with IHD.” - Woolley & Cantrill, 2000.

“One GP told he had a crucial experience during his first year of working (FG1, GP2: ‘‘After the first six weeks, the owner went on vacation. Because I didn’t have a clue, I often called the pharmacist and asked him what to do.’’). One CP mentioned he received a prescription with an eye drops preparation which was ten times overdosed (FG3, CP1: ‘‘I called the GP to ask her if she did it on purpose. She said <No!>. Three days later she called again and thanked me.’’). Another CP reported there was a patient who needed to be treated with fenofibrate which was not available for several months (FG4, CP1: ‘‘We suggested producing capsules with fenofibrate in our pharmacy. The GP reacted in a very positive way and agreed.’’). This highlights the importance of GPs and CPs knowing each other’s competences and tasks, and that their collaboration can jointly contribute to patient care.” – Weissenborn et al., 2017.

“Collaborating in the case conferences provided the pharmacists and physicians with an opportunity to demonstrate to each other their unique skills and practice competence.” - Bell et al., 2007.

“The building of trust and interdependence encouraged health professionals to engage in further opportunities for communication and a stronger understanding of the value of other health professional roles for medication management.” - Disalvo et al., 2019.

"Pharmacists highlighted the potential offered by collaboration for pooling knowledge to gain the best understanding of each resident, in order to provide the most appropriate person-centred care capable of delivering positive outcomes." - Disalvo et al., 2019.

"Pharmacists saw their role and others' as value adding to one another, with pharmacists' knowledge of deprescribing, auditing and monitoring of medications, pain management and weighing risk against benefits of different drugs contributing to the team." - Disalvo et al., 2019.

"CPAMS [CP-Led Anticoagulation Management Service] is the most rewarding project I have been involved in as a pharmacist thus far in my career. I truly enjoyed the collaboration, the patient experiences, and the [opportunity] to practice at a higher level. I always see the benefits to our patients and the cost savings to our health care system. It's a win for all (CP)." – Egunsola et al., 2022).

"For several pharmacists participating in the Nova Scotia and New Zealand CPAMS programs, their comfort and confidence in managing anticoagulation increased substantially over the course of the program. This increase in clinical confidence among New Zealand pharmacists led to the implementation of additional clinical services, such as medicines use review and smoking cessation support." – Egunsola et al., 2022).

"Pharmacists also noted that patients' trust and confidence in them increased over time, and they were more likely to share relevant health information with them. As a result, the pharmacists were able to identify other health needs and address them." – Egunsola et al., 2022.

"Collaborative working arrangements between community pharmacy and primary care could define cohesive care packages, maximising the efficient use of resources, avoiding duplication, and improving patient care. Australian home-based medicine reviews provides a good example of cooperation between GPs and pharmacists.[48] Potentially, systems of this type allow pharmacists and GPs to work together to improve prescribing practices." – Gidman & Cowley, 2013.

Beyond the dyad: supporting patient involvement and engagement

17. Therapeutic relationship

When patients find one-to-one interactions with certain health care professionals intimidating (C), they may seek health advice and care from one whom they judge to be approachable (O), because they feel more comfortable to do so (M).

"A few pharmacists indicated that patients sometimes told them about difficulties with medication cost or adherence before disclosing the problems to their physicians: Pharmacist: 'They'll confess to me . . . "I don't take as much as the doctor said because it's so expensive." I said, "Did you tell the doctor?" "No."' (FG8:2)." – Tarn et al., 2012.

Facilitators included pharmacists' professionalism, ease of access and convenience, not needing an appointment, and feeling more comfortable and relaxed than with a physician." – Brewster et al., 2020.

"Users perceive the pharmacy as a trustworthy, local healthcare resource that is occasionally visited before or even instead of the general practitioner (GP)." – Jove et al., 2014.

"Pharmacists are one of the most trusted professions worldwide alongside firefighters, nurses, teachers and doctors." – Brewster et al., 2020.

"The public's perception of their physician also had an influence on their confidence with pharmacists. Good relations with the physician reduced the need to consult the pharmacist and vice versa. Some believed that a pharmacist's advice needed confirmation by a physician." – Brewster et al., 2020.

"There were mixed responses from pharmacists and MCAs (medicines counter assistants) around pharmacy-based weight management services from positive views of providing the service in community pharmacy to those more reticent who would always favour patients visiting their physician." – Hindi et al., 2018.

"The public's attitudes towards pharmacy services was the second main theme. It depended on the perceived impact of pharmacy services, whether the individual had made use of them before and any barriers and/or facilitators to using pharmacy services."– Brewster et al., 2020.

"The barriers to patients asking pharmacists questions appear to fall into two categories: patient- and pharmacist-related. The patient-related barriers include fear or embarrassment, lacking initiative, having no need for any information and time constraints." - Twigg et al., 2013.

"The pharmacist-related barriers include being seen as less approachable and not being seen as such a credible or trustworthy information source." - Twigg et al., 2013.

"Whilst some patients report an educational benefit from the services a pharmacist provides others prefer to see their own doctor about matters relating to their health and treatment." – Twigg et al., 2013.

"Participants tended to view their GP as the 'controller' of their medical care and that he/she was the person who took overall responsibility for treatment and care for their condition. This appeared to impact on whether they felt comfortable approaching other professionals, and most therefore suggested that they would need the doctor to validate the role of the pharmacist in their care before they could commit to any advice the pharmacist had given them. Participants stated they would be unwilling to let the pharmacist change their prescription medicines unless the doctor had agreed that this was the correct thing to do and had assessed the situation themselves." – Twigg et al., 2013.

"If the pharmacist suggests it to you then you go to the doctor with it and that suggestion, but I would be reluctant to rely entirely on the pharmacists decision that this is different, that this is better or. I

wouldn't take advice from a pharmacist without, especially over the change of medication, without seeing the doctor but then I have got a very good doctor as well." (M4, FG2)" – Twigg et al., 2013.

"I think that the patients will appreciate talking to a professional for a half an hour - compared to the normal five minutes they get with the GP and judging by the problems I see in the shop [community pharmacy] I think, that yes, I will be able to help them, to improve the way they manage their angina." (Pharmacist 2)" - Wolley & Cantrill 2000.

"The majority of the pharmacists expressed some initial anxieties concerning both attendance at, and patient response to, CPRACs: "I thought there might be a bit of 'Oh we don't want to see you, we would rather see the doctor' or 'Oh well you can't do the same things that the doctor does, so it's a waste of time' - that sort of thing, but it's turned out to be the exact opposite. I've yet to come across someone who has said 'Oh I don't want to know about that from a pharmacist' or 'Well I haven't got anything out of coming." (Pharmacist 5)" - Wolley & Cantrill 2000.

"One unforeseen benefit associated with the intensive patient reviews was that they were said to provide many patients with the time and opportunity to express concerns and to discuss issues relating not merely to their angina, but also to other conditions, thus providing a "holistic" approach. The pharmacist interviews suggest that they were able to demonstrate their confidence in dealing with a range of issues. This resulted in improvements in patient management, which were translated into improved patient wellbeing and, as a consequence, heightened motivation towards cardiovascular risk factor modification. One pharmacist when describing his positive experiences from the study said: "One overweight patient, who came in this morning and has managed to lose a half a stone, was very grateful because when he attended for his first review he had obvious signs of heart failure, so I sent him to the doctor straight away. He said, 'it's only since you came here that the doctor has taken an interest in me.' He's feeling a lot better now he's less breathless and his angina is much improved." (Pharmacist 2)" - Wolley & Cantrill 2000.

Although the GPs indicated a high regard for the pharmacists' input as the "experts on drugs" they were also aware of their ability to relate to the patients as individuals experiencing difficulties in coming to terms with the need to accept and modify risky lifestyles. One GP said: "He had a very easy down to earth approach - not like us, too little time and too harsh - no he started slowly and just kept on encouraging them. I think he even got a few to give up smoking." (GP3)" - Wolley & Cantrill 2000.

"Some people feel that they will offend me if they take vitamins… Personally I'm not offended but they might not tell me so the pharmacist telling me that is fantastic (GP)." - Van et al., 2007.

"Pharmacists indicated that patients approached them prior to contacting their physicians. While the pharmacists said that they advised these patients to inform their physicians, they rarely mentioned notifying physicians themselves about patient problems or concerns." - Tarn et al., 2012.

"As shown in the following example, one patient admitted to confiding in her pharmacist, rather than her physician, about not wanting to complete her antibiotics." - Tarn et al., 2012.

"Patient: 'And it will also say on the bottle, complete the prescription. And I said to the pharmacist, oh, you know, I don't want to take it all. God, I feel better. I'm taking six of the antibiotics and I feel great. No, take the complete prescription because if you don't, you could have a relapse. So, I took the whole thing.' (FG3:10) In this case, the pharmacist's counselling contributed to appropriate patient medication use." - Tarn et al., 2012.

"In fact, pharmacists may play an integral role in patient adherence, since patients may use them as sounding boards when they are reluctant to disclose medication difficulties to their physicians." - Tarn et al., 2012.

"Well, I find our pharmacist very very good I mean he's far better than my doctor, we could talk to him can't we and he'll help you, he know most things and, he's just brilliant you know. We can talk to him about things we couldn't with the doctor or the practice nurse and he just. help quite a bit with the different things, if he can help you, he will, he is brilliant." (F6, FG3)." - Twigg et al., 2013.

"Over time, patients had developed certain habits with regard to their interactions with HCP. These were driven by personal preferences for convenience and familiarity, particularly favouring methods that had previously work for them in fulfilling their perceived asthma needs, e.g. selecting a GP who was known to prescribe requested asthma medications without 'interrogation' regarding use or a pharmacy located close by and open when medications were needed." – Cheong et al., 2013.

"Some perceived that the GP surgery was an intimidating, unwelcoming environment with restricted opening hours." – Gidman & Cowley, 2013.

18. Accessibility and anonymity

When patients want accessibility, availability, flexibility, and/or informality (C), they are more likely to access CPs (O) because of convenience (M).

"As providers of primary care, community pharmacies are accessible and convenient, offering long opening hours and nonappointment-based services." – Hindi et al., 2018.

'"... community pharmacy is nearer to my house than the hospital. Pharmacies are widespread, accessible, without appointments and open longer hours." (Pt19, F, 35yrs, D, FH CVD)." - Almansour et al., 2020.

"Pharmacies have longer working hours than many other healthcare facilities and are accessible without registration, therefore, offer a degree of anonymity, alongside a flexible, informal environment." – Brewster et al., 2020.

"GPs perceived a pharmacy-based setting with the presence of patients' medications would enhance the counselling process. Another perceived strength would be pharmacists' ability to obtain in-depth, medication-focused information from patients. GPs thought patients might have barriers to sharing their medication issues with GPs because patients might not feel confident to tell their doctor that they were unhappy with the treatment prescribed." – Hatah et al., 2013.

"With their increased availability, accessibility and informal set-up, pharmacists have the potential to be there for individuals when they are most at need. With appropriate training, they are well positioned to notice 'red flags' such as acute foot problems, frequent hypoglycaemia, diabetes distress and to refer to appropriate services if needed, while offering an alternative means of identifying and reaching out to people who are struggling." – Brewster et al., 2020.

"According to the pharmacists, patients appreciated that the pharmacists took time for them, answered their questions and supported them in addressing their medication problems. This was confirmed by the patients themselves. A previous study also showed that, according to patients, pharmacists should play a more prominent role in their medication management." – Adhien et al., 2013.

"There was general agreement that management in the pharmacy is more convenient for patients: ''It is a lot easier for them to come in to see us than it is to make an appointment with a doctor, take time off work.'' (CP2) - Aradottir & Kinnear, 2008.

"This view of pharmacists as vaccination providers and the pharmacy as an appropriate vaccination location was expressed by another parent who had previously received an influenza vaccine in the pharmacy. They stated, "It was easy, fast, I didn't need to have an appointment. If I could do it at the pharmacy without an appointment, I would do that every time. I'm not gonna lie, if it's something like Tylenol [acetaminophen], I'll buy it at Wal-Mart because it's cheaper, but for everything else, I know my pharmacist and it's convenient and I just, I like them." – Teeter et al., 2021.

"Convenience, accessibility, and trustworthiness have resulted in local pharmacies often being the triage centre for healthcare advice, a valid (mandated) role acknowledged in this study by GPs." – Bryant et al., 2017.

"The frequent contact the public has with pharmacists is unique and this contact is greatest in those with long-term conditions, notably diabetes." – Brewster et al., 2020.

"With their increased availability, accessibility and informal set-up, pharmacists have the potential to be there for individuals when they are most at need. With appropriate training, they are well positioned to notice 'red flags' such as acute foot problems, frequent hypoglycaemia, diabetes distress and to refer to appropriate services if needed, while offering an alternative means of identifying and reaching out to people who are struggling." – Brewster et al., 2020.

"Due to their accessibility and flexibility, community pharmacies are well suited to support and reach out to those with diabetes, particularly those who may be most at need." – Brewster et al., 2020.

"You can't always get in to see your doctor, that quickly but I mean you know you can just whip in there and they will at least give you a good bit of advice if you know nothing else." (F6, FG2)." - Twigg et al., 2013.

"Participants also highlighted the visibly multiple calls on the pharmacist's time and how this might affect their clear availability to answer any questions they may have. This lack of a formal individual appointment time also impacted on whether they were likely to approach the pharmacist for advice." - Twigg et al., 2013.

"It seems very intrusive to take him away from his job to do something that I feel is not part ofwhat his job is, it, I always view the pharmacist as someone who gives you your medicine, mixes it up for you if necessary and dispense it to you as the doctor has prescribed it, and you take it as prescribed, and I would view the doctors surgery is the place to go for advice and not the pharmacist." (M1, FG1)." - Twigg et al., 2013.

"Over time, patients had developed certain habits with regard to their interactions with HCP. These were driven by personal preferences for convenience and familiarity, particularly favouring methods that had previously work for them in fulfilling their perceived asthma needs, e.g. selecting a GP who was known to prescribe requested asthma medications without 'interrogation' regarding use or a pharmacy located close by and open when medications were needed." – Cheong et al., 2013.

"There is evidence that vaccinations are given safely by pharmacists and that access to pharmacies is greatest in areas of social deprivation, in which influenza vaccination rates are low." – Evans et al., 2016.

"It could be argued that community pharmacies are well suited to providing extended services because they are located in local communities." – Gidman & Cowley, 2013.

"Pharmacies provide convenient access points for those who cannot, or do not choose to, make use of other health services. Therefore, community pharmacy could potentially increase access to services and tackle health inequalities by targeting 'hard to reach' groups." – Gidman & Cowley, 2013.

"The pharmacist is bombarded day in and day out with people coming in and out. It's in your face. There's no appointments, you are directly accessible.' [P24]." – Hughes & McCann, 2003.

19. Physical infrastructure and space design

When CP cannot provide a suitable 'safe' confidential space for consultations on what patients judge to be sensitive matters (C), patients may not welcome the services provided at the pharmacy (O), because they value confidentiality and privacy (M).

"The physical layout of the pharmacy also presented a challenge to some pharmacists. Some pharmacies had no comfortable space in between shelves or a private counselling area, and pharmacists felt this limited their ability to deliver effective counselling to their AR patients." – Cvetkovski et al., 2020.

"In terms of the immediate organizational context of the NMS intervention, the physical space that is required in the pharmacy for an intervention to takes place remains an important barrier for pharmacists to engage in NMS. An NMS requires a designated space where the pharmacist can guarantee privacy for the patient. Although the presence of a private space in the Belgian pharmacy is mandatory since 2009, lack of such a space remains a barrier." – Fraeyman et al., 2016.

"GPs also felt that a pharmacy might not be an appropriate setting for discussing confidential issues with patients." – Hatah et al., 2013.

"Barriers included lack of privacy." – Brewster et al., 2020.

"A significant proportion of the public did not know about the private consultation rooms available in many pharmacies, or if they did, had associated them with being used for substance misuse services." – Brewster et al., 2020.

"Pharmacies reported multiple barriers and facilitators in the inner setting. Of note, structural characteristics, such as lack of a private room for vaccine administration, were reported. One pharmacist explained, "There could be a privacy thing. It might make people a little uncomfortable… especially children." – Teeter et al., 2021.

"Privacy in a community pharmacy is also cited as a potential barrier to conducting detailed consultations and therefore to eliciting appropriate advice and information." - Ambler, 2003.

"Some pharmacists expressed concern about new opportunities for providing extra services that required time away training, extra costs, and logistical implications about space within the pharmacy." – Bidwell & Thompson, 2015.

"Current barriers to this model identified were: medico-legal issues, budgetary issues, limited access to patient data, lack of privacy to talk to patients, inadequate time and remuneration, no patient registration, patients describing vague symptoms and patient confidence in pharmacists." – Aradottir & Kinnear, 2008.

"Many patients in this study saw the pharmacist as having the knowledge and time to discuss their medicines and condition but as being impeded from doing so by the community pharmacy environment." - Twigg et al., 2013.

"Many pharmacists also voiced concerns over the suitability or availability of pharmacy consultation rooms." – Hindi et al., 2018.

"Since 2005, community pharmacy has had to become more patient-focused, however, it is clear seven years after the commencement of the new pharmacy contract, that our participants still had concerns about the pharmacy being somewhere they would be willing to discuss private medical problems. These concerns relate to space, time, privacy and relationships with the pharmacist." - Twigg et al., 2013.

"The starkest contrasts were in their diverse views on supermarket pharmacies and 'local' pharmacies. Many participants thought that supermarkets lacked the privacy to enable them to fully discuss their medical problems with the pharmacist. Others thought that the supermarket pharmacy was ideal for their convenience. However, all pharmacies (supermarket and 'local') came under critical scrutiny as being less likely to allow discussions to be conducted with sufficient privacy." - Twigg et al., 2013.

"Occasionally I have gone to the supermarket pharmacy. they'll start talking openly in a supermarket,

over the counter to you about your condition. Now, I don't want to do that, I don't want other people listening to what my problems are. It's that lack of privacy that I don't like, discussing over the counter that I don't like that I think that's awful." (M4, FG4) - Twigg et al., 2013.

"However, other participants saw any crowded place, including the medical practice pharmacy as being at least as restrictive in terms of privacy." - Twigg et al., 2013.

"I mean if I went to the one at the medical practice it's not a big pharmacy, and quite often it's packed with people so you're still going to be standing there at the counter and it's all people listening so I can't see there's any difference, not for me." (F2, FG4).

"For others, any loud conversation conducted in earshot of anyone else would be public: "It's quite a public place. If the pharmacist speaks to you, you're in earshot of everybody else." (M1, FG1)." - Twigg et al., 2013.

"These quotes illustrate the public nature of the pharmacy environment and the discomfort caused with discussing in medical problems in this setting." - Twigg et al., 2013.

"Compared to the medical practice, with private consultation rooms as standard, it is not surprising that participants identified their absence in the pharmacy environment as a flaw for conducting confidential conversations." - Twigg et al., 2013.

"This lack of a suitable, formal and uninterruptible consultation space also appeared to impact on whether participants were likely to request a conversation with the pharmacist." - Twigg et al., 2013.

"Participants still have many reservations about the pharmacy as a suitable place for obtaining comprehensive, confidential healthcare advice that they could act on with confidence. It is perhaps surprising that seven years after the new pharmacy contract in England was implemented; pharmacies have not adapted sufficiently to create an appropriate environment in which patients feel comfortable asking personal medical questions." - Twigg et al., 2013.

"In terms of the pharmacy environment, most pharmacies cannot offer the privacy that this group of patients was seen to prefer to enable them to adequately discuss their medical condition. Even the consultation room, with which most pharmacies are now equipped, did not escape criticism. The 'little room' as it was described is often used as stock storage space and was often small and cramped. Some patients reported wondering why they had been taken into the room, fearing they had done something wrong or identifying it as somewhere stigmatized groups such as drug addicts might be treated." - Twigg et al., 2013.

"Pharmacists may assume that patients are aware of the consultation room and what it entails, and that the patient is automatically comfortable and happy with these consultations as they will view them as similar to those they will have encountered in their medical practice. However, in previous research patients have identified the opposite citing a small room and a lack of knowledge about why they were asked into the room in the first instance, suggesting that pharmacists are not explaining to patients the purpose of the encounter and setting." - Twigg et al., 2013.

"These findings underline the further work the profession may be need to do to educate patients about what the community pharmacy consultation room is like and how it can be used and remove their own perception of how patients view them." - Twigg et al., 2013.

"Any assumption by pharmacists that patients view the consultation room as simply a small medical practice rather than a completely different environment may need to be challenged." - Twigg et al., 2013.

"Privacy for acute consultations was considered essential. Most GPs expected a 'good' pharmacy to have at least one dedicated room, or at least a separate private area, and to take a "proactive approach" to offering it to patients/customers." - Watson et al., 2020.

"...things that are confidential and things that pertain to people's health, other than just giving people simple things for simple problems should always - should never be done in a public place, where a lot of other people should hear it. So it never should be done without privacy." (GP) - Watson et al., 2020.

"The structure does not guarantee a private conversation between the pharmacists and the patient (CP) Cruz et al., 2022.

"Importantly, the pharmacy setting was not seen to offer the privacy required for confidential consultations and health screening. In recent years, community pharmacies have installed consultation rooms in an attempt to provide an element of privacy. However, participants were reluctant to use these due to the consultation room's association with the provision of methadone substance services for problem drug users." – Gidman et al., 2012.

"Some commented that the community pharmacy environment was bright and welcoming; however, many respondents expressed concerns about the lack of privacy. Interestingly, some were unwilling to use consultation rooms because they associated them with opiate-substitution service provision." – Gidman & Cowley, 2013.

"One FG thought that the consultation room was used only for drug misuse clients: "The only people I have seen go in there [a consulting room] are the people on methadone. But other than that, they [community pharmacists] just take them [patients] to the side of the counter and talk to them. We definitely haven't got a room." – Saramunee et al., 2014.

20. Perceived betrayal

When patients are loyal to their GP (C), they may be reluctant to seek health care elsewhere (O), because they are concerned that their GP would view this as a form of betrayal (M).

"Participants again highlighted their trust in the physician to provide most of their information and also raised the fear that by speaking to the pharmacist they might be in some way going against their doctor." - Twigg et al., 2013.

"Participants tended to view their GP as the 'controller' of their medical care and that he/she was the person who took overall responsibility for treatment and care for their condition. This appeared to impact on whether they felt comfortable approaching other professionals, and most therefore suggested that they would need the doctor to validate the role of the pharmacist in their care before they could commit to any advice the pharmacist had given them. Participants stated they would be unwilling to let the pharmacist change their prescription medicines unless the doctor had agreed that this was the correct thing to do and had assessed the situation themselves. "If the pharmacist suggests it to you then you go to the doctor with it and that suggestion, but I would be reluctant to rely entirely on the pharmacists decision that this is different, that this is better or. I wouldn't take advice from a pharmacist without, especially over the change of medication, without seeing the doctor but then I have got a very good doctor as well." - Twigg et al., 2013.

"The participants in this study viewed the doctor as the 'controller' of their medication in a strictly observed health hierarchy so that they did not want to go against any treatment recommendations made by their doctor by obtaining advice from the pharmacist." - Twigg et al., 2013.

"They saw approaching the pharmacist as violating the natural line of treatment. Urban et al. have previously identified that pharmacists believe it is difficult to recruit patients for an MUR as they may be concerned about advice provided that conflicts with their GP's." - Twigg et al., 2013.

"I've known him [GP] my whole life, he's the guy I've always been to (patient) – Cheong et al., 2013.

21. Trust

When patients can see that their GPs and CPs have an effective working relationship (C), they are more willing to access health care and receive services with either (O) because they have confidence in GPs and CPs shared care (M).

"I'm going to let the doctor we've chosen, and trusted help, guide us in those decisions. If she says my children should get the vaccine and it doesn't matter if they get it at her office or the pharmacy or wherever, I trust her." - Teeter et al., 2021.

"Our patients really trust us, but they also really trust their GPs… I think it would be really beneficial if they had that recommendation from the GP to get it at the pharmacy." - Teeter et al., 2021.

"Specific patient groups have highlighted where they view the role of the pharmacist, and this largely depends on their personal experience. Patients also have an idea about how far this role extends to the greater management of their condition with most indicating that they still need the physician to be involved in their care if they are to trust what the pharmacist is doing for them. This is important information if the government's vision for pharmacy is to be realised in patients with chronic conditions." - Twigg et al., 2013.

"Some GPs mentioned that GPs and CPs both had to foster their mutual relationship to increase their patients' trust in healthcare." - Weissenborn et al., 2017.

"Some of the patients start off by coming to see what's on offer, but once they realise how thorough the sessions are, and how much time they get, and that we can help to improve things for them, they start to work with you and not against you." (Pharmacist 1)." – Woolley & Cantrill, 2000.

"Participants in both the patient and pharmacist focus groups indicated that patients often relied on pharmacists to reinforce information discussed with their physicians. Many patients said it was reassuring when the pharmacist and physician said the same things about prescribed medications." – Tarn et al., 2012.

"One patient stated: "Patient: '… what my doctor says to me… if the pharmacist confirms it, then I'm very happy.' (FG5:10). Pharmacists also mentioned the importance of concordant discussions, as exemplified by the following participant: Pharmacist: 'When we reinforce it, I find a lot of patients, not necessarily elderly patients, . . . find that that's comforting. "That's what the doctor told me." Just that two people are confirming the same expectations or something. It's comforting for them and maybe for me too.' (FG8:6)." – Tarn et al., 2012.

"Participants also suggested pharmacists' services would be more credible if offered by pharmacists working in collaboration with a physician to provide CVD risk screening/management services." - Almansour et al., 2020.

"Patients also have an idea about how far this role extends to the greater management of their condition with most indicating that they still need the physician to be involved in their care if they are to trust what the pharmacist is doing for them." - Twigg et al., 2013.

"In relation to pharmacy service provision, whilst some participants had already experienced being offered and had participated willingly in the medicine use review (MUR), an adherence focussed pharmacy service, some said they would want a doctor to refer them to the pharmacist if, as a patient, they were to perceive any benefit to it and if they were to engage with it fully. Repeatedly in the focus groups, participants refer to 'joined up care' and at present their perception of the pharmacist is of their offering isolated, and sometimes duplicating, interventions." - Twigg et al., 2013.

"If the doctor referred me, I'd be happy but if the pharmacist just took it on himself I wouldn't be happy. but I would think if the doctor said go and see the pharmacist to discuss it that would be good." (F1, FG3) - Twigg et al., 2013.

"Participants with more experience of interacting with pharmacists are more willing for them to become involved in wider aspects of their care, with the proviso that it could be seen to be 'joined up' with the work of the rest of the primary care team and that they understood why the pharmacist was becoming involved and what they could add." - Twigg et al., 2013.

"Participants identified that, for their view to change, pharmacists needed to become more integrated into the primary care team with better communication between the different professionals. Such communication would include being referred by the doctor to the pharmacist, so validating the pharmacist's input whilst not deviating from what they saw as the natural line of treatment as well as developing procedures so that the pharmacist can communicate information back to the doctor, if necessary, without the patient having to visit the medical practice." - Twigg et al., 2013.

"While these patients acknowledge that the pharmacist will have a wealth of expertise relevant to administration, supply, side effects and interactions of medicines, because they see pharmacists as isolated from the rest of the primary care team, they are less comfortable with taking up community pharmacist interventions which they see as possibly disrupting treatment by the participant's established care team." - Twigg et al., 2013.

"Our evidence suggests that the public might be more likely to choose community pharmacy services if community pharmacists worked with general practice to better co-ordinate service provision." – Gidman & Cowley, 2013.

22. Overcoming language and cultural barriers

When patients prefer to see health care professionals who they believe share their cultural, ethnic, religious, and/or socioeconomic backgrounds (C), they may seek such service providers (O) because of homophily (M).

"The pharmacy workforce represents people from different cultural, religious, and socio-economic backgrounds. This wealth of diversity potentially minimizes language and cultural barriers, which may otherwise limit an individual's access to healthcare or ability to optimally self-manage their health condition(s)." – Brewster et al., 2020.

''Some patients may be illiterate and can't read written instructions, so they need to do verbal counselling and make sure patients understand how to take their medications and why they are taking them." (Pt10, F, 52yrs, HCL, HTN)." - Almansour et al., 2020.

"For individuals living in low income and/or rural areas with few provider options, these characteristics may make pharmacies especially attractive." - Teeter et al., 2021.

GPs were considerably more divided about the increasing role of pharmacists in a range of areas, and for some this also included reviewing medicines through MMS encounters. Some were unreservedly enthusiastic, finding it saved time for them and benefited their patient:

"I must say it's been really excellent… My context is a largely immigrant population where English is a second language so the time and understanding required to explain the roles of the medications, their side effects, possible interactions, what to watch out for, what their roles are – we can't generate that time for each patient. Forty-five minutes with three monthly follow up, so brilliant, brilliant idea. (GP)." – Bidwell & Thompson, 2015.

"Maybe doctors have no time and that's why I prefer that pharmacists work collaboratively with doctors so that they would clarify things for patients in case doctors don't have enough time. You know it is my first time to talk with the pharmacist and I'm very glad that they listen to the patient and explain everything simply and plainly." (patient) – Almansour et al., 2020.

"Another strength perceived was that pharmacists would have a longer consultation time if funded by the health system to talk about medication use with patients." - Hatah et al., 2013.

"GPs said pharmacists would have an advantage over GPs with regard to consultation time, as in-depth medication reviews are difficult and time-consuming. Furthermore, GPs thought pharmacists could notify the GP about a patient's medication-related problems that the GP might be unaware of. They thought such services could also increase patients' understanding and adherence to medications." – Hatah et al., 2013.

"Well, my recollection [for MUR] is that there were one or two things that the pharmacist pointed out that could be changed for the patient's benefit. (GP#3, G3) [Speaking about MUR]." – Hatah et al., 2013.

"According to the pharmacists, patients appreciated that the pharmacists took time for them, answered their questions and supported them in addressing their medication problems. This was confirmed by the patients themselves. A previous study also showed that, according to patients, pharmacists should play a more prominent role in their medication management." – Adhien et al., 2013.

"They thought that improved communication with healthcare professionals and using simple language would facilitate their understanding of medicine information and encourage them to ask questions about their therapy." – Pasquier & Aslani, 2008.

"With their increased availability, accessibility and informal set-up, pharmacists have the potential to be there for individuals when they are most at need. With appropriate training, they are well positioned to notice 'red flags' such as acute foot problems, frequent hypoglycaemia, diabetes distress and to refer to appropriate services if needed, while offering an alternative means of identifying and reaching out to people who are struggling." – Brewster et al., 2020.

"While the current evidence supports increased integration of pharmacists into the care pathway for those with diabetes, there have been no studies published specifically looking at the role of pharmacists in supporting those with diabetes who are struggling to engage with the services currently available to them or who are 'hardly reached'. Although not exhaustive, this may include people with diabetes who have not been attending their clinical appointments, those not taking their medications, or those with multiple hospital attendances or admissions relating to their diabetes. These individuals are arguably most vulnerable to the complications and health burden associated with diabetes, but potentially also have the most to gain from an alternative supplementary intervention or healthcare service. Although there are likely to be varying reasons and self-determinants underpinning the aforementioned behaviours, pharmacists are in a privileged position to help explore these and offer support to these individuals. Future work will need to build public recognition of pharmacists, while also improving communication between them and other healthcare professionals in order to deliver continuity and best care." – Brewster et al., 2020.

"A survey of patients participating in a randomised controlled trial of a medication management service reported both positive and negative comments regarding the involvement of the pharmacist in their care. They liked how the pharmacists appeared to listen to them for longer than they expected the GP would do and were happy that another healthcare professional was interested in their care, which they found a good source of reassurance." - Twigg et al., 2013.

23. Time and community embeddedness

When CPs devote time and energy to communicating with patients in a way that they can understand (C), patients are supportive of CPs involvement (O), because they value CPs taking time to do this in a way that busy GPs are unable to (M).

"I must say it's been really excellent… My context is a largely immigrant population where English is a second language so the time and understanding required to explain the roles of the medications, their side effects, possible interactions, what to watch out for, what their roles are – we can't generate that time for each patient. Forty-five minutes with three monthly follow up, so brilliant, brilliant idea. (GP)." – Bidwell & Thompson, 2015.

"Maybe doctors have no time and that's why I prefer that pharmacists work collaboratively with doctors so that they would clarify things for patients in case doctors don't have enough time. You know it is my first time to talk with the pharmacist and I'm very glad that they listen to the patient and explain everything simply and plainly." (patient) – Almansour et al., 2020.

"Another strength perceived was that pharmacists would have a longer consultation time if funded by the health system to talk about medication use with patients." - Hatah et al., 2013.

"GPs said pharmacists would have an advantage over GPs with regard to consultation time, as in-depth medication reviews are difficult and time-consuming. Furthermore, GPs thought pharmacists could notify the GP about a patient's medication-related problems that the GP might be unaware of.

They thought such services could also increase patients' understanding and adherence to medications." – Hatah et al., 2013.

"According to the pharmacists, patients appreciated that the pharmacists took time for them, answered their questions and supported them in addressing their medication problems. This was confirmed by the patients themselves. A previous study also showed that, according to patients, pharmacists should play a more prominent role in their medication management." – Adhien et al., 2013.

"They thought that improved communication with healthcare professionals and using simple language would facilitate their understanding of medicine information and encourage them to ask questions about their therapy." – Pasquier & Aslani, 2008.

"With their increased availability, accessibility and informal set-up, pharmacists have the potential to be there for individuals when they are most at need. With appropriate training, they are well positioned to notice 'red flags' such as acute foot problems, frequent hypoglycaemia, diabetes distress and to refer to appropriate services if needed, while offering an alternative means of identifying and reaching out to people who are struggling." – Brewster et al., 2020.

"A survey of patients participating in a randomised controlled trial of a medication management service reported both positive and negative comments regarding the involvement of the pharmacist in their care. They liked how the pharmacists appeared to listen to them for longer than they expected the GP would do and were happy that another healthcare professional was interested in their care, which they found a good source of reassurance." - Twigg et al., 2013.

"They spend time with you, and you can spend more time in the pharmacy than you can the doctor.' (R22)." – Gidman & Cowley, 2013.

"We have more time…you can talk about education, review or whatever is required to be done…we can do inhaler technique…" [PH-016]." – Qazi et al., 2021.

"They also translate complicated medical terminology and the text on the medicine label into a language that the patient can understand." – Rakvaag et al., 2020.

"They thereby support and continue the GP´s work by consolidating the GP´s instructions towards the patient. In sum, the pharmacists feel that they and the GPs complement and complete each other." – Rakvaag et al., 2020.

24. Limited publicity and awareness of CP services

When patients do not know about the range of services that CPs offer (C), they will not make use of these services (O) because of a lack of awareness (M).

“Public cognisance was used to describe the opinions and views of the public. These were influenced by four factors: awareness of the pharmacy services available, underlying perceptions, whether the public regarded physicians to have a supremacy and promotional strategies encountered.” – Brewster et al., 2020.

“There was a general unfamiliarity of the local pharmacy services available, which qualitative work attributed to limited publicity of services.” – Brewster et al., 2020.

“Perceptions of pharmacists were highest for activities linked to their traditional roles relating to medicines such as medicine reviews and advice, but lower for other services.” – Brewster et al., 2020.

“Publicity was generally lacking, but few studies commented on how this could be improved. Word of mouth was deemed to be most effective in a questionnaire but focus group discussions did not reveal a preferable approach.” – Brewster et al., 2020.

“Later focus group work by Hindi et al in 2019 explored the experiences and expectations of patients, pharmacists, and GPs on the integration of community pharmacy into the primary care pathway for people with long-term conditions. Increased public awareness nationally was deemed important, but difficult when different areas provide different services.” – Brewster et al., 2020.

“High quality experience and word of mouth were deemed the most effective ways of publicising services.” – Brewster et al., 2020.

“In indicating a receptiveness to pharmacy-based CVD services, many participants also emphasized the need to promote and advertise these services via different means such as social media, posters in malls and streets, recommendations from doctors, public health experts and other healthcare professionals.” - Almansour et al., 2020.

“A significant proportion of the public did not know about the private consultation rooms available in many pharmacies, or if they did, had associated them with being used for substance misuse services.” – Brewster et al., 2020.

“The main barrier preventing people from visiting the pharmacy first for advice or medication for self-treatable conditions seems to be a lack of awareness of the skills and expertise of the pharmacist.” – Smith et al., 2016.

“More needs to be done to educate people about pharmacy and raise awareness of the expertise of pharmacists. The Government, through NHS England and Public Health England, should partner with the pharmacy sector in a national public education campaign.” – Smith et al., 2016.

"Evidence suggests that the public regard community pharmacy services as beneficial, but the clinical skills and capabilities of pharmacists are under-recognised both by patients and physicians." – Brewster et al., 2020.

"An increased public awareness of the skill set, and role pharmacists have to play is key to building public trust. It is hoped that with increasing recognition in government policy this will begin to be achieved." – Brewster et al., 2020.

"Pharmacists' heavy involvement in the dispensing process, commercial intent, and patients' lack of awareness of pharmacists' ability to offer this service constrain the effectiveness of the service." - Bryant et al., 2017.

"Current barriers to this model identified were: medico-legal issues, budgetary issues, limited access to patient data, lack of privacy to talk to patients, inadequate time and remuneration, no patient registration, patients describing vague symptoms and patient confidence in pharmacists." – Aradottir & Kinnear, 2008.

"Raising public awareness of pharmacist's role in Saudi Arabia is crucial and key to improving patient-centred care services in pharmacies. Lack of public awareness of pharmacist's role and underestimating pharmacist potential to provide extended professional services in community pharmacy in Saudi Arabia was evident within our study and other previous studies." – Almansour et al., 2020.

"Also, there is a mismatch between actual pharmacists' competence and skills and public perception about these aspects, which seems to be an issue across the broader geographic region, for example in Kuwait and the UAE (Hasan et al., 2013, Awad et al., 2017). In fact, this mismatch is apparent even in developed countries, despite the advancement of community pharmacy practice in providing various professional services to consumers beyond medication supply." - Almansour et al., 2020.

"For example, in Wales, United Kingdom, low awareness of available community pharmacy-based services was apparent among participants in an exploratory study that aimed to explore public views of community pharmacy-based services (Kember et al., 2018). Findings suggested the necessity of promoting community pharmacy roles and services to the public; possibly through campaigns run by the government." - Almansour et al., 2020.

"The main government vision for community pharmacy in the UK is for pharmacists to assume greater responsibility for the management of medicines for patients with chronic conditions. To achieve this transformation, public perception of the role of the community pharmacist also may need to be changed, as many people still view the main role of the pharmacist as one of medicines supply." - Twigg et al., 2013.

"Whilst some patients report an educational benefit from the services a pharmacist provides others prefer to see their own doctor about matters relating to their health and treatment. This seems to suggest that patients who have had different experiences of the pharmacist may consequently view them in different ways." - Twigg et al., 2013.

"Findings from Tinelli et al. also confirm this preference, shared by over three quarters of participants in their study still preferring to discuss their medicines with their physician, even after a pharmacist intervention." - Twigg et al., 2013.

"Where patients were not involved in a particular service, the pharmacist has been found to be used primarily by patients to gain information specifically about drug interactions and side effects, with fewer patients wanting to discuss their condition, adherence and impact on their lifestyle." - Twigg et al., 2013.

"The pharmacist-related barriers include being seen as less approachable and not being seen as such a credible or trustworthy information source." - Twigg et al., 2013.

"Participants again highlighted their trust in the physician to provide most of their information and also raised the fear that by speaking to the pharmacist they might be in some way going against their doctor." - Twigg et al., 2013.

"Current barriers to this model identified were: medico-legal issues, budgetary issues, limited access to patient data, lack of privacy to talk to patients, inadequate time and remuneration, no patient registration, patients describing vague symptoms and patient confidence in pharmacists." – Aradottir & Kinnear, 2008.

"The public doesn't fully understand what the pharmacy has to offer. They see us as a supplier of medicines only. (Pharmacist, Saramunee et al., 2014)." – Hindi et al., 2018.

"However, their discussions also made clear that participants were not simply 'anti-pharmacist'; it was more that they did not have a clear understanding of where pharmacist expertise could be useful to them in managing their condition." - Twigg et al., 2013.

"Viewing all of these features of both the pharmacy and pharmacist, together, highlights many changes the community pharmacist and the wider healthcare team will need to make if patients with diabetes, or others with chronic conditions, are going to confidently engage effectively with new pharmacy services and see the pharmacist as a credible healthcare professional. This may involve changing the perception of the pharmacist by patients but also, and possibly more importantly, changing the way pharmacists work and are paid by the NHS." - Twigg et al., 2013.

"Participants were on occasions unaware of the range of services on offer in community pharmacy. A minority were confused about how to access services like minor-ailment schemes and repeat dispensing." – Gidman & Cowley, 2013.

"It was commented on that CPs and GPs should do more to promote services and inform the public about what is available, with leaflets and signs being the most commonly suggested method. It was also felt that 'Government' had a responsibility to promote the role, particularly around public health/ health promotion services." – Kember et al., 2018.

"Well, you could have – like I said before – Public Information films on TV Most doctors surgeries

have um – TV – the TVs- So they could) put it in there sort of thing (PM3).” – Kember et al., 2018.

“Also, maybe you could get GPs to make people more aware of them– because obviously people are obviously always going to see the GP. The GP could always suggest to them that you could actually go to a pharmacist – which would be a lot quicker and a lot more convenient for you– so- (SM3).” – Kember et al., 2018.

“I don’t think it [pharmacy service] is advertised enough. If it was more advertised people would use the pharmacy a lot more.” – Saramunee et al., 2014.

Unanticipated consequences

25. Fragmentation

When CP and GP health care tasks overlap and services are provided in both (C), patients may experience a lack of continuity of care (O), because of fragmentation (M).

“The top four perceived barriers to collaborative practice were lack of time (84.1%), lack of financial compensation (76.3%), lack of face-to-face communication (68.9%), and the possible fragmentation of patient care by the involvement of multiple healthcare professionals (68.9%).” – Albassam et al., 2020.

“When clinic staff were asked about the 3 potential collaboration models, overall sentiments were positive. All clinic staff interviewed expressed interested in collaborating with a pharmacy to provide more HPV vaccines but there were some concerns about documentation and accuracy of records.” – Teeter et al., 2021.

“For example, one physician explained, “Many times my adult patients can get vaccines elsewhere and I don't ever know that it happened. And we're within driving distance of [bordering states] so we see patients from 3 different states. And when it happens state to state, that's when you run into problems. So, when I start talking to them about it, they'll say they've already had that…that is why documentation is extremely important. It needs to be in the vaccine registry, or we need to be informed somehow. We used to get a ton of faxes but that has kind of stopped.” Teeter et al., 2021.

“One of the primary factors causing unease among GPs appeared to be that the MMS service could be initiated by the hospital discharge team, the pharmacist or the patient themselves without the GP’s involvement. The GP may then be unaware that the review was happening until they received the results. This made some concerned that, not only had they been uninvolved, but rather than encouraging integrated care, it was more likely to risk disrupting continuity of care.” – Bidwell & Thompson, 2015.

"Most of the GP participants were also aware that pharmacist prescribing may become a reality. All of them had reservations. Most were opposed; a few believed it may have some place in very limited circumstances. Fragmentation of care and the likelihood of deteriorating conditions being missed were the prime concerns cited: I can't be completely opposed because it's going to happen. But I want to make sure that we don't have people with significant illnesses missed and that doctors are allowed to keep a close eye on their elderly patients and not be seen as – oh, you don't really need to see us because the pharmacist can do it. And it's cheaper sort of thing. (GP)." – Bidwell & Thompson, 2015.

"Sometimes the pharmacist will say something that contradicts what I say to the patient or give medication information that scares the patient from taking what I've prescribed' (GP09)." – Van et al., 2011.

"GPs thought that if prescribing were undertaken outside the GPs' surgery, and without close communication, this could fragment patient care, reduce the frequency of GPs seeing their patients, and decrease their opportunities to talk with their patients about other health concerns. GPs were also worried that those services could conflict with pharmacists' business interests." – Hatah et al., 2013.

"One respondent felt that if too many people were involved patients might miss out on being immunised altogether. 'If you spread things around too much people tend to miss out or fall in the gaps.' (GP5)" - Van et al., 2007.

"OK, it will be nice to do it (collaborate), but still there should be a discussion between the doctor and the pharmacist, because if everyone is working alone, the patient will be in trouble. (FJ-I #2)." – Hasan et al., 2018.

26. Pick and choose: offering options to patients

When patients are given the option of accessing CP and GP services that overlap (C), they may pick and choose which provider to use (O) because they have the option to do so (M).

"GPs thought that if prescribing were undertaken outside the GPs' surgery, and without close communication, this could fragment patient care, reduce the frequency of GPs seeing their patients, and decrease their opportunities to talk with their patients about other health concerns. GPs were also worried that those services could conflict with pharmacists' business interests." – Hatah et al., 2013.

"One physician stated, "If the vaccines were just administered at the pharmacy that would decrease the number of opportunities for the parents to bring their children in for wellness visits. That is what I think they think about when they bring their children in, the shots, they're not thinking about the other advantages of the physical signs, searching for signs of depression, diabetes… I don't really like the [pharmacy-based model] very much." – Teeter et al., 2021.

"Follow-up would be a concern of mine... who is responsible for the next steps of the patients' care? I'm talking about liability – what if it's really bad and the pharmacist recommends, they go see a doctor, but they never go and then something bad happens to them, who is liable? Either party could be." – Participant 11 – Gordan et al., 2018.

"One of the primary factors causing unease among GPs appeared to be that the MMS service could be initiated by the hospital discharge team, the pharmacist or the patient themselves without the GP's involvement. The GP may then be unaware that the review was happening until they received the results. This made some concerned that, not only had they been uninvolved, but rather than encouraging integrated care, it was more likely to risk disrupting continuity of care." – Bidwell & Thompson, 2015.

"This concern of GPs about fragmentation of care was even more pronounced in relation to other expansions of pharmacy into services such as INR testing, and providing influenza vaccines, although for some GPs, anything that increased influenza immunisation coverage was seen positively. There was a strong feeling that such functions were better to be kept within the medical centre, which had traditionally always provided these services. Even those who were the most positive about it would prefer the pharmacist to be within their team: The pharmacist should be as part of the team in this centre and not just randomly doing it because they don't have all the information potentially of what's going on with them [the patient]. (GP)." – Bidwell & Thompson, 2015.

"Most of the GP participants were also aware that pharmacist prescribing may become a reality. All of them had reservations. Most were opposed; a few believed it may have some place in very limited circumstances. Fragmentation of care and the likelihood of deteriorating conditions being missed were the prime concerns cited: I can't be completely opposed because it's going to happen. But I want to make sure that we don't have people with significant illnesses missed and that doctors are allowed to keep a close eye on their elderly patients and not be seen as – oh, you don't really need to see us because the pharmacist can do it. And it's cheaper sort of thing. (GP)." – Bidwell & Thompson, 2015.

"The majority of parents still emphasized the importance of having their child's physician involved in the process… I'm going to let the doctor we've chosen, and trusted help, guide us in those decisions. If she says my children should get the vaccine and it doesn't matter if they get it at her office or the pharmacy or wherever, I trust her." – Teeter et al., 2021.

"When asked about the 3 potential collaboration models, parents expressed interest in the shared-responsibility or pharmacy-based model but explained the importance of including the physician. For example, a parent stated, "If I've already gone through and figured out the risk and feel like I don't have any more questions and the doctor says it's fine, then I think I'd feel comfortable going to a pharmacy." Another parent expressed similar feelings saying, "I'm not opposed to that at all. As long as Dr. [redacted] knows about it, if I received a call from the pharmacy or from Dr. [redacted]'s office that said, hey, you know, your child needs this vaccine and you're not going to get in here for a few weeks, but, you know, you can go ahead and get it at [pharmacy name], nuh-uh, I wouldn't have any problem with that at all." – Teeter et al., 2021.

“Physicians indicated a desire to be involved in at least one component of the child's HPV vaccination process; either through the initial clinic visit and recommendation of the HPV vaccine or by providing the first dose of the vaccine and vaccine education. Parents also stressed the importance of keeping their child's physician involved. As a result, the shared responsibility model was the most highly favoured of the 3 proposed models among primacy care clinic staff and parents.” – Teeter et al., 2021.

“Another physician explained, “I think the most effective would be the shared-responsibility… I think it's still important to have everyone involved with it. I think that one would be more effective, especially with the 2-part vaccination. It's a nice option that patients would welcome. People don't like going to the doctor so if you can avoid that and go to a pharmacy in the community which many times happens to be in a store they're already going to, you're more likely to capture that repeat.” – Teeter et al., 2021

“Utilizing self-care can enhance patients’ confidence by improving their skill set and empowering them to treat certain conditions on their own instead of always relying on an HCP. For certain conditions, a collaborative care model where the individual is first diagnosed by a physician who provides an initial treatment plan is most appropriate [3]. After the initial diagnosis, the pharmacist is responsible for educating the consumer on identifying signs and symptoms of recurrence and ways to effectively self-treat these recurrences, as well as identifying circumstances when it is necessary to consult a physician again, for instance if symptom severity increases.” – Bell et al., 2016.

S5: Included document charactersitics.

Paper N	Author	Year	Title	Journal	Country	Study methodology	Type of document	Type of condition	Topic or focus of paper (e.g., Particular disease/unselected p	Patient group/ Ethnicity/age/gender/	Research objectives and focus	Model/intervention/initiative/theories/theoretical framework	Additional useful information
1	Cvetkovski et al.	2020	Qualitative Exploration of Pharmacists' Feedback Following the Implementation of an "Allergic Rhinitis Clinical Management Pathway (AR-CMaP)" in Australian Community Pharmacies.	Pharmacy.	Australia.	Qualitative study - 15 telephone interviews (15 pharmacists from six pharmacies).	Primary research.	Allergic Rhinitis.	AR management.	Patients with AR.	This study aimed to explore the experiences and perceptions of community pharmacists with regards to the implementation of AR management guidelines in real-life everyday practice.	The "Promoting Action on Research Implementation in Health Services (PARIHS)" framework, designed to ensure the optimal implementation of evidence-based guidelines in healthcare settings.	The PARIHS framework identifies three key elements—1) evidence, 2) context, and 3) facilitation—that are essential for the successful implementation of guidelines in practice. With regards to AR and the implementation of guidelines, the AR Clinical Management Pathway (AR-CMaP) is a guideline pathway for community pharmacists based on this implementation framework. It was specifically developed and implemented to address the physical environment, leadership, culture, and evaluation barriers associated with the implementation of guidelines in practice, as described in PARIHS.
2	Fraeyman et al.	2016	Evaluating the implementation fidelity of New Medicines Service for asthma patients in community pharmacies in Belgium.	Research in Social and Administrative Pharmacy.	Belgium.	Qualitative evaluation - telephone interviews with pharmacists, interviews with patients, and focus groups among GPs and lung specalists.	Primary research.	Asthma.	In October 2013, a New Medicines Service (NMS) was introduced in community pharmacies in Belgium to support asthma patients who are novice users of inhaler devices with corticosteroids. The present study evaluated the degree to which the NMS program is delivered as intended, drawing on the concept of implementation fidelity.	Asthma patients who are novice users of inhaled corticosteroids, meaning first use in the past 12 months.	In October 2013, a new community pharmacy asthma service was introduced in Belgium. The service is a form of NMS, which was recently introduced in the UK, with the aim to improve adherence to newly prescribed medication. This study aimed to evaluate degree to which the NMS program is delivered as intended, drawing on the concept of implementation fidelity.	The evaluation of implementation fidelity of the NMS program drew on the conceptual model proposed by Carroll et al. This model describes the main dimensions of implementation fidelity and highlights potential factors that moderate the implementation of a program, thereby affecting its effects.	
3	Hatah et al.	2013	General practitioners' views of pharmacists' current and potential contributions to medication review and prescribing in New Zealand.	Journal of Primary Health Care.	New Zealand.	Qualitative study - semi-structured interviews.	Primary research.	N/A	Internationally, non-medical practitioners are increasingly involved in tasks traditionally undertaken by GPs, such as medication review and prescribing. This study aims to evaluate GPs' perceptions of pharmacists' contributions to those services.	N/A	This study aims to explore GPs' perceptions of pharmacists' current and potential contributions to services in NZ.	N/A	Semi-structured interviews were carried out in two localities with GPs whose patients had and had not undergone a pharmacist-led adherence support Medication Use Review (MUR). GPs were asked their opinions of pharmacists' provision of MUR, clinical medication review and prescribing
4	Van et al.	2011	General practitioner–pharmacist interactions in professional pharmacy services.	Journal of Interprofessional Care.	Australia.	Qualitative study - Individual semi-structured face-to-face and telephone interviews were conducted with a purposive sample of 15 GPs and 15 pharmacists in rural and metropolitan areas of New South Wales, Australia	Primary research.	N/A	Evaluating the extent of interaction and collaboration between GPs and CPs in the context of professional pharmacy services (PPS). This study aims to evaluate 1) the nature and extent of interations 2) factors influencing interactions	N/A	The aim of this study was to investigate (1) the nature and extent of interactions between GPs and CPs and; (2) the factors that influence these interactions in the context of PPS	N/A	Paring; Although the study was carried out in Australia, it's mentioned that Home Medicines Review (HMR) is comparable to MUR in the UK. The nature and extent of interactions between GP–pharmacist pairs were often seen to be unique to that particular pair
5	Maxwell et al.	2014	Using a conflict conceptual framework to describe challenges to coordinated patient care from the physicians' and pharmacists' perspective.	Research in Social and Administrative Pharmacy.	United States.	Qualitative evaluation - two rounds of face-to-face interviews with physicians and community pharmacists. The first round involved one-on-one interviews with pharmacists and physicians. The second round brought pharmacist–physician dyads together in an open-ended interview exploring issues raised in the first round.	Primary research.	N/A	Using constructs from the conflict conceptual framework, the investigation focused on identifying and describing challenges faced by physicians and those faced by pharmacists not currently in a patient-centered medical home in their efforts to provide coordinated quality patient care.	N/A	Using constructs from the conflict conceptual framework, the investiga- tion focused on identifying and describing challenges faced by physicians and those faced by pharmacists not currently in a patient-centered medical home in their efforts to provide coordinated quality patient care	This study is based on the conflict theoretical framework provided through Wilmot and Hocker's (2011) definition of conflict, which is expressed as an expressed struggle between interdependent parties with perceived incompatible goals, scarce resources, and interference from others in achieving goals. The framwork is used to examine the interpersonal relationship of physicians and pharmacists, and between the professional roles and within the environment where each practices locate.	
6	Teeter et al.	2021	Perceptions of HPV vaccination and pharmacist-physician collaboration models to improve HPV vaccination rates.	Exploratory Research in Clinical and Social Pharmacy.	United States.	Qualitative evaluation - developmental formative evaluation using semi-structured interviews with pharmacy staff, primary care clinic staff, and parents of adolescent children living in the southern US.	Primary research.	HPV Vaccination	Human Papillomavirus (HPV) is the most common sexually transmitted disease in the United States (US), with 12 cancer causing strains. .HPV vaccination rates in the southern US fall below the national average. Study is thus conducted to 1) identify barriers and facilitators to providing the HPV vaccine and Vaccines for Children (VFC) program participation in pharmacies and clinics, and 2) assess pharmacy staff, clinic staff, and parent perceptions of 3 collaboration models to improve HPV vaccination.	Parents of adolescents age 11–17.	1) Identify barriers and facilitators to providing the HPV vaccine and Vaccines for Children (VFC) program participation in pharmacies and clinics, and 2) Assess pharmacy staff, clinic staff, and parent perceptions of 3 collaboration models to improve HPV vaccination.	1. "shared responsibility model" - an agreement between physician/clinic and pharmacist/pharmacy can be established so that the first dose of HPV vaccine is given in the clinic while the second dose is provided in the pharmacy,may be beneficial for pharmacy/clinic partnerships in which both are enrolled in the VFC program. 2. "pharmacy-based model" in which a pharmacist and physician agree that all adolescent patients will receive a strong recommendation in the physician's clinic and all doses of HPV vac- cine will be administered in the pharmacy. This collaboration model may be beneficial for pharmacy/clinic pairs in which only the pharmacist is en- rolled in the VFC program. 3. "insourced model" in which a pharmacist and physician agree on specific days/times that the pharmacist provide immunizations in the physician's clinic. This may also be a collaboration model that is beneficial for pharmacy/clinic pairs in which the pharmacist is the only VFC enrolled provider.	Community pharmacies are highly accessible when compared to clinical vaccination sites due to their extended business hours, no copays for visits, and no requirement to schedule an appointment. For individuals living in low income and/or rural areas with few provider options, these characteristics may make pharmacies especially attractive. Pharmacies reported multiple barriers and facilitators in the inner setting. Of note, structural characteristics, such as lack of a private room for vaccine administration, were reported. Pharmacists were confident in their ability to provide the vaccine and counsel patients. "[The pharmacy] is one place I think it should be administered, but it's not happening yet…conversations mostly happen at physicians' offices… but we should be having those conversations in the pharmacy, too." - quote from a pharmacist.
7	Urban et al.	2009	Perceptions of medicines use reviews — the views of community pharmacists within a West Yorkshire primary care trust.	the Pharmaceutical Journal.	UK.	Qualitative study - semi-structured interviews.	Primary research.	N/A	CPs' experiences of conducting medicine use reviews (MURs), including how this affects their relationship with GPs.	N/A	To explore community pharmacists' experiences of conducting medicine use reviews (MURs), including how this affects their relationship with GPs and the extent to which training and accreditation prepared them for this work.	N/A	Of those who were not providing an MUR service, one claimed that the available remuneration would not compensate for the effort required to provide a service of the required quality and another complained of a poor relationship with the local GP so did not think that setting up an MUR service would be worthwhile. Pharmacists reported that conducting MURs had increased patients' confidence in the pharmacist and raised the profile of the pharmacist as an accessible health care professional. However, MURs may have a negative impact on other patients waiting for their prescriptions to be dispensed while the pharmacist was busy conducting MURs. This serves as reminder that it is important to maintain the continuity and efficiency of the essential dispensing service.
8	Bidwell & Thompson.	2015	GPs, community pharmacists and shifting professional boundaries.	The New Zealand medical journal.	New Zealand.	Qualitative study - interviews with 16 GPs and 17 pharmacists were conducted in the Canterbury region.	Primary research.	N/A	In the context of expectations regarding role evolution, including increased interprofessional working, this study aimed to gain insight into how GPs and pharmacists understood the professional role of the pharmacist and its expansion, extension and calls for increased collaboration.	N/A	Gain insight into how GPs and pharmacists understood the professional role of the pharmacist and its expansion, extension and calls for increased collaboration.	N/A	The complexity surrounding this issue is the respective groups' differential positioning within the healthcare arena, both in terms of power and employment structure. Fears about conflicts of interest, particularly as this related to dispensing self-written prescriptions, was raised by some of those concerned about the extension of prescribing to some pharmacists.
9	Hindi et al.	2019	Solidarity or dissonance? A systematic review of pharmacist and GP views on community pharmacy services in the UK.	Health and Social Care in the Community.	UK.	Systematic review - UK studies investigating pharmacists' and/or GPs' views of community pharmacy services or roles from 2005 to 2017.	Secondary research.	N/A	There has been a strong policy emphasis over the past decade on optimising patient- centred care and reducing general practitioners' (GPs') workload by extending community pharmacy services and collaboration between pharmacists and GPs. This paper aims to review current evidence of pharmacists' and GPs' views of extended community pharmacy services and pharmacists' roles in the United Kingdom (UK).	N/A	By undertaking a systematic review looking at UK studies investigating pharmacists' and/or GPs' views of community pharmacy services or roles from 2005 to 2017, this study reviews the current evidence of pharmacists' and GPs' views of extended community pharmacy services and pharmacists' roles in the United Kingdom.	The community pharmacy contractial framework - In England, the revised contractual framework consists of three types of ser- vice: "essential", "advanced" and "locally commissioned". "Essential services" cover traditional services provided by all community phar- macies (i.e. dispensing medications/appliances, repeat dispensing and signposting). "Advanced services" focus on medication reviews conducted by pharmacists in the form of one-to-one consultations. "Locally commissioned services" include a wide range of medication and public health services such as minor ailments management, life- style advice, substance misuse and sexual health service	
10	Wilcock et al.	2007	General practitioners' perceptions of medicines use reviews by pharmacists.	the Pharmaceutical Journal.	UK.	Qualitative study - self-administered questionnaire.	Primary research.	N/A	To explore GPs' perceptions of community pharmacist-conducted medicines use reviews.	N/A	Explore GPs' perceptions of community pharmacist-conducted medicines use reviews.	N/A	GPs reported the following would constitute useful MUR: Having a single sheet of paper with brief relevant action points; The importance of having a closer working relationship with the practice; Receiving information on patients who have compliance problems or where there are issues with adverse effects or interactions; The pharmacist ensuring that patients understand more about their medicines; Targeting of MURs to particular groups of patients (eg, those on polypharmacy or complex regimens, those who do not attend surgery for follow up, and conducting domiciliary MURs)<br>GPs reported the following would constitute MUR regarded as a waste of time: Pages of information and having to hunt for (unhelpful) advice; Asking the GP to check BPs etc when these reviews have already been done; MURs conducted on patients who have been on the same treatment for years and the GP knows they are compliant; When the practice has recently conducted a medication review; One that highlights known problems that the GP is working on or has solved to their best ability; Discussing adverse effects that are inevitable and which the GP will have balanced against the clinical need for the drug
11	Bidwell et al.	2017	A model of multidisciplinary professional development for health professionals in rural Canterbury, New Zealand.	Journal of Primary Health Care.	New Zealand.	Qualitative study - anonymous questionnaire.	Primary research.	N/A	An initiative by the Pegasus Clinical Quality and education team aimed to provide more satisfactory and accessible professional development for these rural areas, in order to fill in the gap that rurally located health practitioners have largely been unable to participate because of the travel time and distances involved.	N/A	The initiative reported in this paper aimed to fill this gap by developing an accessible and high-quality multidisciplinary model of professional development for general practitioners, nurse practitioners, practice nurses and community pharmacists in rural areas of North Canterbury, New Zealand.	N/A	
12	Bollen et al.	2018	Factors influencing interprofessional collaboration between community pharmacists and general practitioners—A systematic review.	Health and Social Care in the Community.	Australia (n = 12), Canada (n = 10), Cuba (n = 1), England (n = 3), Germany (n = 3), Iran (n = 1), Ireland (n=2),Malaysia(n=1),Scotland(n =1),Spain(n=1),US(n=1), and Wales (n = 1). Three studies were conducted in lower/upper-middle income economies (Cuba, Malaysia and Iran).	Systematic review - 37 publicaitons.	Secondary research.	N/A	Effective interprofessional collaboration is critical for sustaining high quality care in the context of the increasing burden on primary healthcare services. Despite this, there is limited understanding of the factors contributing to effective collaboration between general practitioners and community pharmacists. The aim of this systematic review was to identify the factors that impact on interprofessional collaboration between general practitioners (GPs) and community pharmacists (CPs).	N/A	The aim of this systematic review was to identify the factors that impact on interprofessional collaboration between general practitioners (GPs) and community pharmacists (CPs).	A conceptual model describing the working relationship between pharmacists and physicians in a community setting was developed in one study by McDonough and Doucette (McDonough & Doucette, 2001), who synthesised the model from interpersonal and business relation- ships, and collaborative care. Important concepts in this model are the participant (e.g., knowledge, attitudes, beliefs, experience), context (e.g., proximity and practice features) and exchange char- acteristics (e.g., communication, power, and expectations), that influence the ability to reach certain stages of collaboration. This study was conducted in 2001 but given the substantial and rapid increase in pressure on primary care subsequently, contemporary understanding is now required. Bardet, Vo, Bedouch, and Allenet (2015) reviewed specific models related to collaboration between physicians and CPs in primary care. This review showed that the key concepts influencing collaboration were: trust, interdependence, perceptions, and expectations about the other professional, skills, interest for collaborative practice, role definition, and communica- tion (Bardet et al., 2015). Reported barriers for collaboration were lack of time and remuneration, and facilitators included proximity (positively influencing collaboration due to increased accessibility) and encouraging regular contacts (Bardet et al., 2015). However, this review only included studies that made use of models and not the wider evidence base; therefore a complete overview of all research regarding factors impacting collaboration between GPs and CPs was not provided and is necessary. Given that effective collaboration between primary care professions can contain costs and improves patient outcomes and quality of life of patients, a synthesis of the evidence base is critical in order to gain insight into factors influencing collaboration and to improve collaborative working.	Whilst literature to date demonstrates a growing number of interventions that aim to improve collaboration in healthcare, there is still limited evidence regarding the effectiveness. Identified approaches that aimed to enhance collaboration included educational activities (e.g., seminars, workshops, and simulations), practice interventions (e.g., checklists, team briefings, and patient safety rounds), and a few organisation-based practices (e.g., policies, guidelines, redesign of workspaces) . While outcomes showed that the interventions improve the attitudes, knowledge, and skills of the professionals, evidence on change in individual behaviours, organisational practices and patient benefit still lacks. Furthermore, studies that were conducted in community settings are limited, with most studies conducted in mental health facilities or hospitals.
13	Bradley et al.	2008	The challenge of integrating community pharmacists into the primary health care team: A case study of local pharmaceutical services (LPS) pilots and interprofessional collaboration.	Journal of Interprofessional Care.	UK.	Mixed-method evaluation.	Primary research.	N/A	The factors which improve and impede the integration of community pharmacy with other organizations, examine the LPS pharmacists' perceived level of integration into the PHCT and investigate their relationships with local GPs. contributes to the existing body of literature on interprofessional relationships, but also examines the impact GP- pharmacist relationships can have on the success or failure of enhanced pharmacy services.	N/A	Investigate interprofessional collaboration between general practitioners (GPs) and pharmacists involved in the delivery of enhanced pharmacy services under the local pharmaceutical services (LPS) contract in England.	N/A	The reasons why some GPs chose not to be involved in LPS are unknown. Evidence from one pilot does suggest that GPs were offended by the introduction of a pharmacist led medication review service, feeling that it implied shortcomings in their existing level of care. This could suggest a certain level of professional insecurity.
14	Bryant et al.	2009	General practitioners' and pharmacists' perceptions of the role of community pharmacists in delivering clinical services.	Research in Social and Administrative Pharmacy.	New Zealand.	Mixed-method evaluation.	Primary research.	N/A	Investigate whether community pharmacists' and general practitioner's perceptions of the role of community pharmacists may be a barrier to pharmacists increasing their role in medication management. Other potential barriers were also explored that could provide a framework for future research.	N/A	The aims of this study were to examine whether the perception of the roles of community pharmacists may be a barrier to greater involve- ment in medicines management and to explore other potential barriers that may provide a frame- work for further study.	N/A	The results from this survey do not differ greatly from those of other surveys on general practitioner's perceptions of the role of commu- nity pharmacists, particularly the traditional roles of supply and distribution,counseling,and monitoring for compliance.The survey found only marginal support from general practi- tioners for counseling about the benefits of medicines, a role not specified in other surveys. This may be considered to be encroaching on the general practitioner's territory. There was less agreement with respect to monitoring therapy.
15	Bryant et al.	2010	Community pharmacist perceptions of clinical medication reviews.	Journal of Primary Health Care.	New Zealand.	Qualitative study.	Primary research.	N/A	Community pharmacists are being encouraged to increase their participation in patient-focussed services such as medication reviews. implementation of these services into routine practice have been very limited because of perceived physical or functional barriers. This study explores possible attitudinal factors that prevent increased participation of community pharmacists in medication reviews undertaken in collaboration with GPs.	N/A	This study explores possible attitudinal factors that prevent increased participation of community pharmacists in medication reviews undertaken in collaboration with GPs.	N/A	In response to the broad question about the good aspects of the reviews, the pharmacists found particular satisfaction in building a better rapport with customers, and making a difference or help- ing people. Although the themes of mandate, legitimacy, effectiveness and adequacy emerged, a final ques- tion on whether the pharmacists believe there is a future for clinical medication reviews provided a summary of their overall view. All the pharma- cists considered that these reviews should be part of the future for pharmacy. A number of pharmacists commented that the standard of the reviews needed to be maintained and the service needed to be done properly if there was to be a future for clinical medication reviews.
16	Dahne et al.	2019	General practitioner–pharmacist collaboration in Germany: an explanatory model.	International Journal of Clinical Pharmacy.	Germany.	Quantitative evaluation.	Primary research.	N/A	To provide evidence for the factor structure of the previously validated Frequency of Inter-professional Collaboration Instrument and the Attitudes Toward Collaboration Instrument in measuring attitudes toward and frequency of collaboration from the general practitioner's perspective in the context of primary care in Germany; to develop an explanatory model which illus- trates factors influencing collaboration.	N/A	The objectives of this study were to provide evidence for the factor structure of two previously validated instruments of physician-pharmacist collaboration in measuring atti- tudes toward and frequency of collaboration as perceived from the general practitioner's perspective and to develop an explanatory model which illustrates factors influencing general practitioner-pharmacist collaboration.	The instrument measuring frequency (Frequency of Inter-professional Col- laboration Instrument (FICI-GP)) was developed and validated using an Australian practitioner cohort, it has eleven Likert-type items addressed collaboration (communication, information sharing, shared decision making). The scale measuring attitudes (Attitudes Toward Col- laboration Instrument (ATCI-GP)) was developed and validated in an Australian sample of 376 physicians. It comprised 15 items on a five-point Likert-scale ('strongly disagree', 'disagree', 'neither', 'agree', 'strongly agree') which reflected three determinants of collaboration (inter- actional, practitioner, environmental)	Mecklenburg-Western Pomerania is prototypical of rural areas in Germany for the purposes of exploring practitioner-pharmacist interactions in the context of an ageing multi-morbid population using polypharmacy. This cross- sectional study provided a snapshot of the current attitudes and behaviours of general practitioners toward collaboration with pharmacists. Whilst respondents expressed generally positive attitudes to the value of collaboration, the current level of collaboration was very limited.
17	Elvey et al.	2008	General practitioner engagement: the key to repeat dispensing?	International Journal of Pharmacy Practice.	UK.	Qualitative evaluation.	Primary research.	N/A	Repeat dispending by community pharmacists.	N/A	Identify and explore general practitioners' (GPs') experiences of, and views about, repeat dispensing by community pharmacists.	N/A	Recent evidence about the provision of services through the new contract for community pharmacy in England provides evidence that locality groups, where GPs and pharmacists meet to discuss services including repeat dispensing, can be helpful.Efforts to highlight benefits to practices, such as the potential to score points under the Medicines Manage- ment collaborative indicators in the Quality and Outcomes Framework (QOF), the system through which payments are allocated to general practice, may also be worthwhile
18	Smith et al.	2016	Self Care Nation – Self care attitudes and behaviours in the UK.	N/A	UK.	N/A	Primary research.	N/A	Self care attitudes and behaviours in the UK.	N/A	To explore self care attitudes and behaviours in the UK and conclude on how to empower people to self care	N/A	
19	Waszyk-Nowaczyk et al.	2021	Cooperation Between Pharmacists and Physicians – Whether It Was Before and is It Still Ongoing During the Pandemic?	Journal of Multidisciplinary Healthcare.	China, Qatar, USA, Canada, UK, Pakistan, Saudi Arabia, Itady, Israel.	Systematic review.	Secondary research.	N/A	View the literature on the cooperation of physicians and pharmacists in the provision of medical services for patients.	N/A	To view the literature on the cooperation of physicians and pharmacists in the provision of medical services for patients. Analyzing the challenges and approaches can lead to better and improved health care.	N/A	Furthermore, tough pandemic times, more than ever, have emphasized the importance of cooperation between physicians and pharmacists, regarding providing medical services for patients.
20	Watson et al.	2020	"What counts can't always be measured": a qualitative exploration of general practitioners' conceptualisation of quality for community pharmacy services.	BMC Family Practice.	UK.	Qualitative study - semi-structured interviews.	Primary research.	N/A	Exploration of general practitioners' conceptualisation of quality for community pharmacy services.	N/A	explore GPs': - Conceptualisation of quality for community pharmacy services, including the management of acute (low acuity) conditions and defining indispensable aspects of the patient experience ('always events'); - Opinions regarding whether and how to measure quality in the community pharmacy sector.	An emerging approach to exploring and defining healthcare quality, and one that has also been used in general practice, is the concept of 'always events'. The IHI has defined these as "aspects of the patient experience that are so important to patients, their care partners, and service users that health care providers must aim to perform them consistently for every individual, every time"	
21	Almansour et al.	2020	Health consumer engagement in developing novel preventive health community pharmacy roles in cardiovascular disease in Saudi Arabia: A qualitative analysis.	Saudi Pharmaceutical Journal.	Saudi Arabia	Qualitative study - semi-structured interviews.	Primary research.	Cardiovascular disease.	Health consumers' experiences of the current role of the pharmacists in Saudi Arabia and their perspectives around possible future pharmacy services with particular focus on CVD and related risks.	Face to face interviews were conducted with 25 individuals (11 from Najran and 14 from Riyadh) who had at least one modifiable CVD risk factor. Most participants were Saudi Arabian (88%) and the majority were women (56%). Nineteen (76%) participants were recruited via pharmacies and general practitioners' (primary care physicians') offices or primary healthcare centres, four (16%) were recruited from a community centre in Riyadh and two (8%) were recruited via personal contacts.	Health consumers' experiences of the current role of the pharmacists in Saudi Arabia and their perspectives around possible future pharmacy services with particular focus on CVD and related risks.	N/A	
22	Ambler	2003	General practitioners and community pharmacists: times they are a-changing.	British Journal of General Practice.	UK.	Editorial.	Secondary research.	N/A	Based on the current published literature to structure a meaningful dialogue and debate and to support general practitioners and pharmacists through a potentially difficult period of change to promote collaboration.	N/A	Based on the current published literature, to and address three critical questions, which may help us to structure a meaningful dialogue and debate and to support general practitioners and pharmacists through a potentially difficult period of change:1. Can a pharmacist currently undertake the main task of a general practitioner?<br>2. Can a community pharmacist work with a general practi- tioner to provide high-quality care?<br>3. Does the high-quality care provided by general practitioners and pharmacists meet the needs and expectations of patients?	N/A	We need to be wary of being too introspective and excluding patients from our deliberations. If we work with patients and involve them in designing new services we may be surprised about the extent and pace of the change that can be achieved18 — without damaging any of the parties' confidence or sense of self worth.
23	Anderson & Sharma	2020	Primary health care policy and vision for community pharmacy and pharmacists in England.	Pharmacy Practice.	UK.	Editorial.	Secondary research.	N/A	This paper describes the NHS and primary care and community pharmacy in England at the start of 2020, a time of great change.	N/A	This paper described the NHS 10 year plan, Primary care context, overview of community pharmacy in England. It gave an overview of medicines optimisation and safety by describing the New Medicines service, structured mdication reviews. It also covers prevention strategies including fluvaccination service and urgent care (i.e., Community Pharmacist Consulation Service.)	N/A	N/A

24	Aradottir & Kinnear	2008	Design of an algorithm to support community pharmacy dyspepsia management.	Pharmacy World & Science.	UK.	Qualitative study.	Primary research.	Dyspepsia.	Care pathway and referral documentation system to support dyspepsia management in the community pharmacy.	N/A	To design a care pathway and referral documentation system to support dyspepsia management in the community pharmacy according to national guidelines. To explore attitudes of community pharmacists and general practitioners (GPs) towards the proposed model of care.	N/A	The finding provides tools for pharmacist prescribers to use in a partnership approach to dyspepsia management in accordance with national guidelines. It should encourage evidence based management of dyspepsia ensuring that appropriate patients are referred for investigation and uncomplicated patients are managed safely and appropriately.	
25	Banh & Cave	2021	A De Novo Pharmacist-Family Physician Collaboration Model in a Family Medicine Clinic in Alberta, Canada	Pharmacy.	Canada.	Quantitative evaluation.	Primary research.	Chronic diseases.	Collaborative practice in health-care has proven to be an effective and efficient method for the management of chronic diseases. This study describes a de novo collaborative practice between a pharmacist and a family physician.	159 patients, with an average age of 62, 76 male, 83 female. Regarding past medicl history, 57 HTN, 32 Mental health, 22 DM, 22 Chronic pain, 17 Hypothyroidism, 14 Dyslipidemia.	The primary objective of the study is to describe the collaboration model between a pharmacist and family physician. The secondary objective is to describe the pharmacist workload.	A De Novo Pharmacist-Family Physician Collaboration Model in a Family Medicine Clinic: The pharmacist would independently see patients who required prescription refills, comprehensive medication reviews, or follow up visits for stable chronic conditions such as hypertension, diabetes, mental health, chronic pain, dyslipidemia and hypothyroidism. Each patient visit is scheduled n the family physician schedule in 20 min time slot except for physical exams or short procedures, which are allocated more time. At the start of the clinic, both the family physician and pharmacist review the patient list and decide which patient will be seen by the pharmacist independently as above. This will expedite the clinic flow as the pharma- cist would be able to discharge the patient after the visit without a consultation with the family physician. As for the patients who would be seen collaboratively, there is a brief discussion of the approach to provide care to the patient based on the reason for the visit. The pharmacist would see the patient and gather pertinent information and assess and develop a plan. The plan is discussed with the family physician. Both the family physician and the pharmacist would deliver the plan to the patient. If the patient required a follow up appointment, the pharmacist would be able to see that patient independently.		
26	Bardet et al.	2015	Physicians and community pharmacists collaboration in primary care: A review of specific models.	Research in Social and Administrative Pharmacy.	Multiple.	Systematic review.	Secondary research.	N/A	Identify theoretical models that have been developed to understand the physician-CP collaboration (PCPC) and to identify the associated determinants.	N/A	This review is aimed to identify theoretical models that have been developed to understand the physician-CP collaboration (PCPC) and to identify the associated determinants.	Four specific models of collaboration centered on PCPC were identified: (i) the Collaborative Working Relationship Model (CWR), (ii) the Conceptual model of GPCP collaboration, (iii) the CP Attitudes towards Collaboration with GPs Model (ATC-P), (iv) the GP Attitudes towards collaboration with CPs (ATC-GP). The CWR was first described in 2001 by McDonough et al, the GPCPC conceptual model in 2012 by Bradley et al, the rCWR by Dey et al in 2011, and the ATC-P and the ATC-GP respectively in 2012 and 2013 by Van et al. The CWR is a model based on the trust development between professionals. It describes the collaboration dynamics in five stages, from 'Professional awareness' to 'Commitment to the Collaborative Working Relationship'. An instrument, the "Physician- Pharmacist Collaboration Instrument" (PPCI) has been developed from the CWR to measure PCPC from the physician's perspective. It was then adapted for CPs. PPCI is not validated to explore PCPC from pharmacists' perspective. The GPCPC conceptual model was developed from the new pharmaceutical ser- vices experimented in the United Kingdom. The GPCPC conceptual model was established on the experience of PCPC from two qualitative studies conducted in the United Kingdom on local pharmaceutical services and repeat dispensing, with both GP's and CP's. It was conceptu- alized from interviews conducted with HCPs involved in a collaborative practice, according to the Grounded Theory. Modeling was based on the theoretical contributions of the Contingency Theory of Laurewce and Lorsch, the conceptual framework of integration in public health of Ax- elsson and Axelsson and used the terminology defined by Hudson et al and Armitage. The GPCPC conceptual model describes the collabo- ration dynamics in three stages: isolation, commu- nication and collaboration. The ATC-P and the ATC-GP are two empirical models developed on the experience of PCPC after the implementation of HMR and dia- betes medication	CPs and physicians do not consider collaborative activities as a prior- ity over the dispensing tasks. Lack of time and remuneration, which are often cited as barriers for the implementation of collaborative CPS, may reflect a lack of motivation.	
27	Barron et al.	2016	Report of the inquiry into primary and community care: how can service improvements be achieved and where does pharmacy fit?	All-Party Pharmacy Group.	UK.	Qualitative study.	Primary research.	N/A	Understand the greater role that pharmacy could, and should, be playing in the context of the Five Year Forward View and its focus on prevention.	N/A	In October 2015 the All-Party Pharmacy Group (APPG) launched an inquiry into primary and community care. The inquiry sought to understand the greater role that pharmacy could, and should, be playing in the context of the Five Year Forward View and its focus on prevention.	N/A		
28	Bawab et al.	2020	Implementation and Effectiveness of an Interprofessional Support Program for Patients with Type 2 Diabetes in Swiss Primary Care: A Study Protocol.	Pharmacy.	Switzerland.	Mixed-method evaluation.	Primary research.	Type 2 Diabetes.	Illustrates the use of implementation science to support the development, dissemination and integration in primary care of effective and sustainable collaborative pharmacy services for chronic care management.	Patients with type 2 diabetes taking at least one oral antidiabetic medication in the French-speaking part of Switzerland.	The study's objectives are:<br>(1) To evaluate the appropriateness of the implementation strategies for Siscare-DT2,<br>(2) To describe the implementation process of Siscare-DT2 in the French-speaking part of Switzerland, and<br>(3) To evaluate the effectiveness of Siscare-DT2 for patients with type 2 diabetes.	In this study, the Framework for Implementation of Services in Pharmacies (FISpH) was used, which was adapted for the community pharmacy setting from the Generic Implementation Framework (GIF). The core concepts are the process of implementation (divided into stages), the innovation to be implemented, and the multi-level context (divided into domains: individuals, organization, local setting and system) in which the implementation is to occur, which is influenced by factors, strategies, and evaluations	The scientific evaluation observes the process in stages, which will provide insights on both the effectiveness and the identified barriers and facilitators for its implementation in primary care. In particular, the results will add new knowledge on the recommendations regarding the need to adapt the framework conditions (e.g., strategies and cost) to broaden the application of these collaborative models.	
29	Ekincioglu & Demirkan	2018	Opinions of Community Pharmacists About Collaboration with General Practitioners: A Descriptive Pilot Study After New Legislation in Turkey.	Turkish Journal of Pharmaceutical Sciences.	Turkey.	Quantitative evaluation.	Primary research.	N/A	Explore community pharmacists' opinions on collaborative care along with new primary care regulations.	N/A	The aim of this study was to identify community pharmacists' opinions on a shared care process and their willingness to be involved in patient monitoring in order to improve quality in primary health care settings.	N/A	About the new legislation: In Turkey, the National Health Service in primary care has been revised and general practitioners' (GPs) roles have been expanded since 2010. The new legislation on the primary health care process was introduced in 2013 and revised in 2015. By January 2013, electronic prescribing also began to be implemented in Turkey. Previously, GPs were responsible for monitoring chronic diseases as well as providing preventive health services along with the nurses in primary care settings. Before the new legislation, GPs practicing in primary care settings were only allocated in a small and a limited number of community clinics in a city where they only have close contact with nurses for the patient care process. Patients were free to attend any community clinics for their monitoring and prescriptions according to their preferences. After the legislation, the patients are obliged to attend a certain healthcare center and monitored by an assigned GP according to their home addresses, and were not allowed to change their doctor within the first 3 months. Patients are guided to visit GPs first before attending secondary or tertiary care settings where health expenses could not be covered by the [illegible]	
30	Bell et al.	2007	Mental health case conferences in primary care: Content and treatment decision making.	Research in Social and Administrative Pharmacy.	Australia.	Qualitative study.	Primary research.	Mental illness.	Explore the process of decision making in mental health case conferences involving community pharmacists and primary care physicians.	N/A	The objective of this study was to explore the content and treat- ment decision-making processes in mental health case conferences involving primary care physicians and community pharmacists.	The Model of Shared Decision Making was originally proposed to describe acute care decision making that occurs in consultations between physicians and patients.33,34 Proponents of the Model have acknowledged that decision making about chronic illnesses in primary care may involve teams of health professionals. More recently, the Model has been used to describe the communication between staff and clients in multidisciplinary community mental health teams. The Model states that for a shared decision to take place, 3 criteria must be met: (1) information exchange, (2) deliberation, and (3) decision making. Information exchange refers to the type and amount of infor- mation that is exchanged, and whether this exchange is 1 or 2 way. The deliberation stage refers to expressing and discussing treatment options. The final stage refers to deciding which treatment to implement. For a shared decision to take place, both parties must take steps to build consensus (through the deliberation process) and have an investment in the ultimate decision.	In October 2001, the Australian Government introduced a fully funded medication management review service (known as "Home Medicines Re- view" or HMR) undertaken collaboratively by community pharmacists and primary care physicians. This service represented the first opportunity for pharmacists and physicians to collaborate on professional issues in a structured and remunerable manner in the community setting. The HMR service is potentially well suited to identifying and resolving medication- related problems among people with a mental illness. The majority of HMRs are undertaken using a system of paper-based referrals and reporting. Conducting a case conference following the HMR may provide an important opportunity for direct face-to-face interprofessional discussion between the referring physician and reviewing pharmacist. A case conference is a multidisciplinary meeting of 2 or more health pro- fessionals to plan care for a specific person with chronic and complex care needs. Although psychiatrists and physicians are remunerated for their partici- pation in case conferences, the uptake of this service in primary mental health care has been low. Reasons suggested for the low uptake of case conferences include the lack of remuneration for allied health professionals and the difficulty in arranging mutually [illegible]	
31	Bell et al.	2016	Self-Care in the Twenty First Century: A Vital Role for the Pharmacist.	Advances in Therapy.	Multiple.	Review article.	Secondary research.	N/A	Self-care and the role of the pharmacis in assisting self-care.	N/A	Using the management of frequent heartburn with an OTC proton-pump inhibitor as a model, describe global healthcare trends affecting self-care with a specific focus on the role of the pharmacist in facilitating over-the-counter (OTC) medication management. The importance of the collaboration between healthcare providers (HCPs), including specialists, primary care providers, and pharmacists, is also discussed.	N/A	Self-care is characterized by healthcare consumers who take proactive roles in identifying and managing their health conditions to optimize their overall physical health and psychological and spiritual well-being	
32	Bereznicki et al.	2011	Perceived feasibility of a community pharmacy-based asthma intervention: a qualitative follow-up study.	Journal of Clinical Pharmacy and Therapeutics.	Australia.	Qualitative evaluation.	Primary research.	Asthma.	Pharmacy-based asthma intervention	N/A	Explore the views of general practitioners (GPs), community pharmacists and patients towards a pharmacy-based intervention that saw patients with poorly managed asthma supplied with educational material and referred to their GP for an asthma manage- ment review.	N/A		
33	Berry et al.	2011	Asthma Friendly Pharmacies: A Model to Improve Communication and Collaboration among Pharmacists, Patients, and Healthcare Providers.	Journal of Urban Health.	United States.	Qualitative evaluation.	Primary research.	Asthma.	The Asthma Friendly Pharmacy (AFP) model which aim to improve Communication and Collaboration among Pharmacists, Patients, and Healthcare Providers	Asthma patients.	This article describes the feasibility of the model in an urban community pharmacy setting and documents the interventions and communication activities promoted through the AFP model.	The Asthma Friendly Pharmacy (AFP) model. The goal of the AFP model was to use, at each step of the dispensing process, both pharmacists and technicians to effectively and efficiently deliver routine, brief (i.e., 3–5 minute) interventions to all patients (adults and children) with inhaled-medication prescriptions.		
34	Beyene et al.	2020	Pharmacists views on participating in New Zealand's community pharmacy anticoagulation management service: a mixed methods study.	International Journal of Clinical Pharmacy.	New Zealand.	Mixed-method study.	Primary research.	Anticoagulation therapy.	The Community Pharmacy Anticoagula- tion Management Service (CPAMS) has beneficial effects on anticoagulant management. However, limited research exists on pharmacists' views of CPAMS provision, particularly the perspectives of pharmacists who do not provide CPAMS.	Patients on anticoagulation therpay and require INR monitoring.	To explore the experience and attitudes of pharmacists who do and do not provide CPAMS, and to identify factors that may influence further uptake of CPAMS	The Community Pharmacy Anticoagulation Management Service (CPAMS), was first piloted in NZ at 15 community pharmacies between November 2010 and July 2011. In the CPAMS model, community pharmacists provide point-of-care INR testing (with the device Coaguchek XS Plus or Pro) and adjust war- farin doses as needed using a decision support system INR Online. Pharmacists provide the service in collaboration with the patient's family doctor, and the doctor takes overall responsibility for the patient's management and could intervene at any time. The results of the pilot study showed that CPAMS achieved greater anti- coagulation control than GP-led warfarin management.		
35	Bishop et al.	2021	Pharmacists' perceptions of the Canadian opioid regulatory exemptions on patient care and opioid stewardship.	Canadian Pharmacists Jounral.	Canada.	Qualitative evaluation.	Primary research.	N/A	Barriers and facilitators of providing opioid stewardship activities in pharmacy practice	N/A	This study explored the perceptions of canadian pharmacists about the barriers and facilitators of providing opioid stewardship activities in pharmacy practice, considering the subsection 56(1) class exemption under Health canada's controlled Drugs and substances Act (cDsA).	N/A		
36	Black et al.	2015	Collaboration with Community Pharmacies in the Patient Centered Medical Home.	Primary Care Reports.	United States.	Qualitative evaluation.	Secondary research.	N/A	PCMH model, leveraging CP services in the PCMH model, joint principles of PCMH and CP	N/A	Illustrate the principles of the PCMH model with examples of how PCPs working within the PCMH framework can reach out and leverage partnerships with community pharmacy (CP) service providers in pursuit of improved patient engagement and satisfaction as well as the better outcomes that provide the objective basis for measuring quality of care.	While emphasis has been placed on enhancing patient satisfaction in an effort to increase patient involvement and commitment to their own health, a great deal of focus has been placed on encouraging healthcare professionals to commit to cooperative efforts and greater accountability to improve the quality of the care they provide.The patient centered medical home (PCMH) is a model that leverages the skills and training of primary care physicians (PCP) to help coordinate these efforts toward healthcare reform. The PCMH model for primary care was first introduced by the American Academy of Pediatrics (AAP) in the 1960s. The PCMH model has since been widely promoted as a potential basis for the reform and improvement of healthcare delivery in the United States.<br>In the PCMH model, a PCP serves as the leader of a care team dedicated to identifying and meet- ing the healthcare needs of the patient. Enlisting and coordinating the services of consultants, specialists, and other healthcare service providers in a cooperative effort provides PCPs in a PCMH practice with the information needed to track and monitor the care pro- vided through other segments of<br>the healthcare system. Collecting, coordinating, and reconciling information from other facilities, such as hospitals or skilled nursing facilities, further helps the PCP maintain their involvement in patient care, even when outside of the primary care setting. This allows the PCP to facilitate safe and efficient transitions of care when patients move between different care settings. There are seven components to the Joint Principles of the PCMH - 1. A Personal Physician, 2. Physician-directed Medical Practice, 3. Whole Person Orientation, 4. Coordinated and/or Integrated Care, 5. Quality and Safety, 6. Enhanced Access, 7. Payment. The overall aim of these principles is to provide patients with a principal healthcare relationship that engages them in a [illegible]	The premise is that these combined efforts will increase efficiency and effectiveness of healthcare delivery in this country while reducing the per capita cost.	
37	Blenkinsopp & Bond.	2008	The Potential and Pitfalls of Medicine Management What Have We Learned So Far?	Disease Management & Health Outcomes	UK.	Qualitative evaluation.	Secondary research.	N/A	Community pharmacy medicines management (CPMM)	N/A	Can CPMM services improve health outcomes? Which barriers and challenges prevent medicines management achieving its full potential? What needs to be done in the future to achieve the full potential of CPMM?	CPMM was defined by the British National Prescribing Centre as:<br>"... a range of activities intended to improve the way that medi- cines are used, both by patients and by the NHS [National Health Service]. Medicines management services are processes based on patient need that are used to design, implement, deliver and monitor patient-focused care. They can include all aspects of the supply and use of medicines, from an individual medication re- view to a health promotion programme."	Community pharmacies are widely considered to be an under-utilized resource. Despite being educated to degree level and having a thorough understanding of pharmacology and therapeutics, those working in community pharmacy settings spend much of their time involved in the technical (dispensing) rather than the cognitive (advisory) aspects of medication supply.A final point to note when defining the role of medicines management is that the patient makes the ultimate decision about whether or not to take the treatment. The involvement of the patient in decision making is a central component of the decision of whether or not to prescribe a medication. Patients whose treatment goals have been identified and met are more likely to comply with medication regimens. However, despite an ever greater understanding of this fact and incorporation of greater patient involvement in prescribing decisions in the UK and else-where, there is an ever growing mountain of dispensed but unused medicines, and therefore wasted resource.	
38	Blondal et al.	2017	General practitioners' perceptions of the current status and pharmacists' contribution to primary care in Iceland.	International Journal of Clinical Pharmacy.	Iceland.	Qualitative study.	Primary research.	N/A	Icelandic general practitioners' views on the current status of primary care, their perceptions of pharmacists as a health care profession, and their attitudes towards future GP-pharmacist collaboration in primary care in Iceland.	N/A	To explore Icelandic general practitioners' views on the current status of primary care, their perceptions of pharmacists as a health care profession, and their attitudes towards future GP-pharmacist collaboration in primary care in Iceland.	N/A	The primary care system in Iceland is very complex. The service is on many levels: primary care, organ specialists, emergency department, and patient care are getting more and more heterogeneous. Further, as mentioned above, there is a shortage of GPs, they work under a strict business form (rules about ownership of primary care clinics where GPs are not allowed to freely establish a practice), and GPs do not have a gatekeeper role. Thus, GPs grapple with a number of critical challenges, while having pharmacists as members of their team is far from being the highest [illegible]	
39	Bradley et al.	2008	The challenge of integrating community pharmacists into the primary health care team: A case study of local pharmaceutical services (LPS) pilots and interprofessional collaboration.	Journal of Interprofessional Care.	UK.	Mixed-method evaluation.	Primary research.	N/A	Interprofessional collaboration between general practitioners (GPs) and pharmacists involved in the delivery of enhanced pharmacy services under the local pharmaceutical services (LPS) contract in England.	N/A	The aim of this paper is to investigate interprofessional collaboration between general practitioners (GPs) and pharmacists involved in the delivery of enhanced pharmacy services under the local pharmaceutical services (LPS) contract in England.	N/A	It proved difficult to access GPs for interview generally, especially those not involved in LPS. Consequently, the reasons why some GPs chose not to be involved in LPS are unknown. Evidence from one pilot does suggest that GPs were offended by the introduction of a pharmacist led medication review service, feeling that it implied shortcomings in their existing level of care. This could suggest a certain level of professional insecurity.	
40	Bradley et al.	2011	Social network analysis of contact made between community pharmacists and general practitioners. (conference abstract)	International Journal of Pharmacy Practice.	UK.	Qualitative study.	Primary research.	N/A	GP and community pharmacist contact at four geographically diverse areas using social network analysis.	N/A	Usingsocial network approach, this project aimed to explore the network of contact between GPs and community pharmacists at four case study areas.	Social network analysis, which focuses on the ties and connections between 'actors', has the potential to aid understanding of relations between health care organisations and teams. Using this approach, this project aimed to explore the network of contact between GPs and community pharmacists at four case study areas		
41	Bradley et al.	2012	Integration and differentiation: A conceptual model of general practitioner and community pharmacist collaboration.	Research in Social and Administrative Pharmacy.	UK.	Qualitative evaluation.	Primary research.	N/A	Conception model of GP and CP collaboration.	N/A	This article presents a new model of collaboration derived from interviews with general practitioners (GPs) and community pharmacists in England involved in service provision that required some form of collaboration.	Existing models of inter professional collaboration:<br>In Primary Care:<br>Armitage - 5- stage taxonomy of collaboration in primary care<br>1. Isolation: Members who never meet, talk or write to one another<br>2. Encounter: Members who encounter or correspond with others but do not interact meaningfully<br>3. Communication: Members whose encounters or correspondence include the transference of information<br>4. Collaboration between two agents: Members who act on the information sympathetically: participate in patterns of joint working: subscribe to the same general objective as others on a one-to-one basis in the same organisation<br>5. Collaboration throughout the organisation: Organisations in which the work of all members is fully integrated<br>Hudson et al - 4-point continuum of collaboration: isolation/encounter, communication, collaboration, and integration.<br><br>GP/CP specific model:<br>McDonough and Doucette's collaborative relationship model for pharmacists and physicians in the US: Demonstrate factors influencing the level of collaboration, including participant characteristics (level of education, training experience, and age), context characteristics (practice environment, type, and size), and exchange characteristics (trustworthiness, relationship initiation, and role specification). In which trustworthiness and role specification were reported to be the most significant influencing factors.	Fig3 in the paper shows the model at different levles (level 1-isolation, level 2 - communication, level 3- collaboration) in detail with examples.	

42	Bradley et al.	2012	What key components are required to improve pharmacist-GP collaboration?	the Pharmaceutical Journal.	UK.	Qualitative evaluation.	Primary research.	N/A	Conception model of GP and CP collaboration.	N/A	The article presents the main findings of a previous study (paper N41) and number of points to consider when focusing efforts on improving collaborative relationships between community pharmacists and GPs.	N/A		
43	Bradley et al.	2018	Negotiating inter-professional interaction: playing the general practitioner-pharmacist game.	Sociology of Health & Illness.	UK.	Qualitative evaluation.	Primary research.	N/A	It focused on the subtle and nuanced aspects of the GP-CP relationship, identifying a set of unwritten rules, that, community pharmacists in particular, may feel required to follow in practice.	N/A	This study investigates how GPs and community pharmacists perceive that they negotiate their interactions, how they attempt to establish, maintain and develop their relationships through these interactions, and how they play the 'GP-pharmacist game'.	N/A	In 1967, Stein conceptualised the interaction between doctors and nurses as 'the doctor-nurse game'. This 'game' involved the dominant doctor being covertly guided in clinical decisions by the subservient nurse.	
44	Brewster et al.	2020	The role of community pharmacists and their position in the delivery of diabetes care: an update for medical professionals.	Postgraduate Medical Journal.	UK.	Qualitative study.	Secondary research.	Diabetes.	Describe how integration of community pharmacy services into existing healthcare models may improve diabetes care.	Patients with diabetes (type 1 and 2).	The aim of this narrative review is to familiarise medical professionals with the expanding role of community pharmacists, and to explore the poten- tial for their improved integration in the delivery of diabetes care and its associated services.	N/A	While the current evidence supports increased integration of phar- macists into the care pathway for those with diabetes, there have been no studies published specifically looking at the role of pharma- cists in supporting those with diabetes who are struggling to engage with the services currently available to them or who are 'hardly reached'. These individuals are arguably most vulnerable to the complications and health burden associated with diabetes, but potentially also have the most to gain from an alternative supplementary intervention or healthcare service. Although there are likely to be varying reasons and self- determinants underpinning the aforementioned behaviours, pharmacists are in a privileged position to help explore these and offer support to these individuals.	
45	Bryant et al.	2017	Changing perspectives of the role of community pharmacists: 1998 – 2012.	Journal of Primary Health Care.	New Zealand.	Quantitative study.	Primary research.	N/A	Perceptions of community pharmacists and GPs of the expanding medicines management roles of community pharmacists in New Zealand.	N/A	To compare data from a 2012 study with two previous studies (1998, 2002) examining perceptions of community pharmacists and GPs of the expanding medicines management roles of community pharmacists.	N/A		
46	Buss et al.	2020	Exploring pharmacists' views on an in-pharmacy medication review program in Australia using a mixed-methods design	International Journal of Clinical Pharmacy.	Australia.	Mixed-method evaluation.	Primary research.	N/A	To explore MedsCheck from the com- munity pharmacists' perspective, focusing on the perceived effectiveness of the program, barriers to its optimal delivery, and the integration with other services.	N/A	This study was designed as an exploration of MedsCheck from the community pharmacists' perspective. The key research questions were: (1) what are pharmacists' perceptions of the effectiveness of MedsCheck; (2) what are the perceived barriers to the implementation/sustainability of MedsCheck; and (3) do pharmacists integrate MedsCheck with other pharmacy services?	N/A	Same barriers are being repeatedly described in international pharmacy practice research. Working relationship between pharmacists and GPs, the documentation, and too nonspecific definitions of the service were barriers in the delivery of the Medicines Use Review in the UK (Blenkinsopp A, Celino G, Bond C, Inch J. Medicines use reviews: the first year of a new community pharmacy service. Pharm J. 2007;278:218–23.). Several issues including: that the patients who are most in need were often not reached; difficulties in pharmacist-GP collaborations; focus on quantity instead of quality; and insufficient support, resources, skill sets, and confidence from pharmacists' side (Latif A. Community pharmacy medicines use review: current challenges. Integr Pharm Res Pract. 2017;7:83–92).	
47	Cadogan et al.	2015	Improving appropriate polypharmacy for older people in primary care: selecting components of an evidence-based intervention to target prescribing and dispensing.	Implementation Science.	UK.	Qualitative study - semi-structured interviews.	Primary research.	Polypharmacy.	Polypharmacy.	Patients receiving multiple mediines, especially elderly.	1) identify key theoretical domains that were perceived to influence the prescribing and dispensing of appropriate polypharmacy to older patients by general practitioners (GPs) and community pharmacists, and (2) map domains to associated behaviour change techniques (BCTs) to include as components of an intervention to improve appropriate polypharmacy in older people in primary care.	The underpinning theoretical model was the Theoretical Domains Framework (TDF). The TDF has been used to identify key theoretical domains that are perceived to influence HCPs' behaviours and provides a theoretically robust evidence base to inform intervention design. Key domains are then mapped to behaviour change techniques (BCTs) which can form the intervention's so-called 'active ingredients'. The identified intervention components (i.e. BCTs) were used to develop an evidence-based and well-theorised intervention (details of which will be reported in a future publication) ready for feasibility testing at a later stage of the project, before progressing to a larger scale evaluation in a randomised study.		
48	Campbell et al.	2021	Electronic transmission of prescriptions in primary care: transformation, timing and teamwork.	Journal of Primary Health Care.	New Zealand.	Qualitative study - semi-structured interviews.	Primary research.	N/A	Electronic transmission of prescriptions in primary care.	N/A	The purpose of this study was to undertake a preliminary exploration of the experiences of New Zealand general practitioners and community pharmacists when prescriptions are transmitted electronically directly from prescriber to pharmacy.	N/A	In the future, there may be opportunity to include patients as well with tools such as the shared electronic medication plans described in a recent Swiss study. (Bugnon B, Geissbuhler A, Bischoff T, et al. Improving primary care medication processes by using shared electronic medi- cation plans in Switzerland: lessons learned from a participa- tory action research study. JMIR Form Res. 2021;5(1): e22319. doi:10.2196/22319)	
49	Campion et al.	2007	Shared prescribing? A focus group study with community pharmacists.	Primary Health Care Research & Development.	UK.	Qualitative evaluation.	Primary research.	N/A	The RESPECT (Randomised Evaluation of Shared Prescribing for the Elderly in the Community, randomised over Time) trial.	N/A	This paper reports a qualitative study designed to explore attitudes of community pharmacists towards the process of 'pharmaceutical care' as tested in the trial.	N/A	The RESPECT (Randomised Evaluation of Shared Prescribing for the Elderly in the Com- munity, randomised over Time) trial (Wong et al., 2004) was designed to evaluate the impact of pharmaceutical care on the appropriateness of prescribing for patients aged 75 years or older. Community pharmacists across five Primary Care Trusts (PCTs) provided pharmaceutical care to over 700 patients for 12 months in a randomized time series sequence. All participating pharmacists received training in the provision of pharmaceu- tical care. This included two 3-hour meetings, and some 'pre-workshop tasks' or homework, covering conducting medication reviews and constructing care plans. In one of the two training evenings, general practitioners (GPs) and their local community pharmacists were 'paired up', with the aim of opening up communication and beginning the professional liaison, which is an important part of pharmaceutical care.	
50	Cardwell et al.	2018	Community pharmacists' views of using a screening tool to structure medicines use reviews for older people: findings from qualitative interviews.	International Journal of Clinical Pharmacy.	UK.	Qualitative evaluation.	Primary research.	N/A	Medicines use review (MUR) service.	N/A	To explore community pharmacists' views on the facilitators and barriers towards the utilisation of a screening tool as a guide to conducting structured MURs.	The Theoretical Domains Framework (TDF) (12 domain and 14 domain) was developed by a group of experts as a means to help identify the factors (barriers and facilitators) to achieving behaviour change, particularly in the context of clinical practice.	In a 2016 qualitative study by Rathbone et al. researchers identified a number of determinants of success- ful collaboration (between GPs and community pharmacists in Australia) to improve patients' adherence to prescribed medicines. The barriers identified were a lack of shared location, a difference in perspectives, poor communication due to gatekeepers (GPs' receptionists), irregular or volatile communication as well as a lack of mutual and professional respect. Conversely, the facilitators identified were access to a shared location, shared perspectives, face-to-face or electronic communication, regular communication as well as mutual and professional respect. (Rathbone AP, Mansoor SM, Krass I, Hamrosi K, Aslani P. Quali- tative study to conceptualise a model of interprofessional collaboration between pharmacists and general practitioners to support patients' adherence to medication. BMJ Open. 2016;6:e010488.)	
51	Cerbin-Koczorowska et al.	2019	As the twig is bent, so is the tree inclined: a survey of student attitudes toward interprofessional collaboration supported with the curricula analysis.	Journal of Interprofessional Care.	Poland.	Mixed-method study.	Primary research.	N/A	Pharmacy and medicine student attitudes toward interprofessional collaboration in Poland.	N/A	To assess the perception of physician-pharmacist collaboration among final-year medical and pharmacy students through questionnaires with both closed-ended and open-ended questions.	N/A		
52	Chaplin.	2014	Ten things GPs need to know about pharmacists.	Prescriber.	UK.	N/A	N/A	N/A	The author describes a new guide for GPs that aims to foster a better understanding of the role of community pharmacists.	N/A	The author describes a new guide for GPs that aims to foster a better understanding of the role of community pharmacists.	N/A		
53	Cheong et al.	2013	Primary health care teams and the patient perspective: A social network analysis.	Research in Social and Administrative Pharmacy.	Australia.	Mixed-method evaluation.	Primary research.	Asthma.	Multidisciplinary care (MDC), with a focus on astham patients.	English speaking patients, 18 years old or over, with a physician diagnosis of asthma reported by participants.	This research aims to gain a deeper understanding of patients' potential role in MDC: i) describe patients' health networks, ii) compare different care groups, iii) gain an understanding of the nature and extent of their interactions, and iv) identify the role of pharmacists within patient networks.	Social network theory focuses on relationships between individuals, groups, organizations, or complete societies. An underlying principle of the social network approach is that network analyses were investigated through the properties of relations (ties) between individuals (nodes) in a network. These connections may include the physical contact between individuals, the exchange of in- formation, or the presence of feelings towards individuals. By mapping these relationships and connections, social network analysis (SNA) describes the unique patterns, structures and compositions of networks, and how they influence individual's behavior. An egocentric social net- work focuses on an individual's network and its effects on that individual. It describes the net- work of a focal node (ego) and all nodes to whom ego has a connection to. Unlike traditional analytical approaches, egocentric SNA focuses on personal network data as opposed to whole or socio-centric data. In this study, an egocentric framework was applied whereby a sample of unre- lated patients with asthma (egos) was recruited and information about each health connection in their asthma management (personal asthma networks) was gathered, whether these are individuals (HCPs, family, friends) or other informa- tion sources (internet, books, brochures).	2 distinct groups of participants were recruited. One group (clinic group) consisted of participants who were recruited from an asthma clinic, known to consist of a range of HCPs, such as respiratory physicians (specialists), specialized respiratory nurses and pharmacists, as well as respiratory function scientists involved in respiratory function assessments. The second group (community group) consisted of participants who were re- cruited from a range of standard asthma health care access points. These included general practice clinics and community pharmacies in the Sydney metropolitan region, Australia; the Asthma Foun- dation – New South Wales, Australia; Sydney local newspapers; and the University of Sydney, Camperdown Campus, Sydney, Australia.	
54	Community Pharmacy Workforce Development	2021	A review of the community pharmacy workforce: 2021 and beyond	N/A	UK.	N/A	Report.	N/A	Pharmacy workforce: current situation and moving forward.	N/A	This paper recognises the ongoing ambition to better utilise the clinical skills of pharmacy colleagues and work to enable the future pharmacists to prescribe. It makes recommendations to enable the community pharmacy workforce to deliver the ambitions set out.	N/A		
55	Costa et al.	2015	Investigating general practitioner engagement with pharmacists in Home Medicines Review.	Journal of Interprofessional Care.	Australia.	Quantitative evaluation.	Primary research.	N/A	Home Medicines Review (HMR).	N/A	Assess GP attitudes and behaviours regarding the HMR and modelled factors associated with the frequency of GP engagement with HMRs.	This questionnaire, the Home Medicines Review Inventory (HMRI), consists of four behaviours items and six attitude items (Table I). The first four items assess frequency of behaviours related to the HMR performed in the last month, scored on a 4-point Likert-type scale (''Nil'', ''1–2'', ''3–4'' and ''5 or more''). The remaining items assess the GPs' attitude to the HMR, scored on a 5-point Likert-type scale (''Strongly disagree'', ''Disagree'', ''Neither agree nor disagree'', ''Agree'' and ''Strongly agree'').	HMR: In Australia, HMR was introduced in 2001 as a strategy to address the high prevalence of preventable adverse drug effects and medication errors contributing to morbidity and admission to hospitals. An HMR may be initiated for an at-risk patient by referral from a general practitioner (GP) to an accredited HMR pharmacist who then visits the patient in their home to conduct an interview during which the patients' medicines are discussed and inspected. After the review, the pharmacist prepares a report for the patient's GP with recommendations for improving medicine management. The pharmacist and GP discuss the findings in the report and the GP then develops a medicine management plan for the patient. The HMR is a free service to consumers but pharmacists and GPs are both reimbursed by the government.	
56	Cruz et al.	2022	Pharmaceutical services in primary health care: dissatisfaction among users, managers and pharmacists.	Brazilian Journal of Pharmaceutical Sciences	Brazil.	Mixed-method evaluation.	Primary research.	N/A	Performance indicators for pharmaceutical services (PS) in primary health care (PHC) in Brazil.	N/A	To assess the performance indicators for pharmaceutical services (PS) in primary health care (PHC), the level of satisfaction with pharmacy services among users and managers / pharmacists' impressions in relation to the findings were evaluated.	N/A	Pharmaceutical service (PS) provided by Brazil's Unified Health System (Sistema Único de Saúde - SUS) is designed to fulfill multiple functions that aim to facilitate access and rational use of medicines in order to promote, protect and recover individual and collective health.	
57	Curran et al.	2019	Communication between pharmacists and primary care physicians in the midst of a U.S. opioid crisis.	Research in Social and Administrative Pharmacy.	United States.	Qualitative study.	Primary research.	N/A	Communication between prescribers of opioids and community pharmacists in the US.	N/A	To present and explore key emergent themes from a qualitative study around the nature, frequency, and content of communication between primary care physicians (PCPs) and pharmacists focusing on opioids.	N/A	Studies of physician and community pharmacist relations/collaboration often find some element of a "hierarchy" between physicians and pharmacists, with physicians being somewhat weary of pharmacists questioning or attempting to change prescribing behavior and many pharmacists being uncomfortable with making recommendations. However, as noted in the current study and in Chui et al.,12 there seems to be more collaboration and "co-managing" of patients between pharmacists and physicians within the same healthcare systems as opposed to between independent physicians and community/retail pharmacists. (Chui MA, Stone JA, Odukoya OK, Maxwell L. Facilitating collaboration between pharmacists and physicians using an iterative interview process. J Am Pharm Assoc JAPhA. 2014;54:35–41.)	
58	Damiaens et al.	2021	General Practitioners and Community Pharmacists' Collaboration in Primary Care: Small Steps for a Major Change.	International Journal of Integrated Care.	Belgium.	Mixed-method evaluation.	Primary research.	N/A	Medical-Pharmaceutical Concertation (MPC) in Belguim.	N/A	To evaluate the implementation of MPC from the perspective of healthcare authorities and GPs/CPs.	Normalization Process Theory (NPT), another widely- known and frequently-used implementation framework, which states that the goal of an initiative should be clear to all parties in order to streamline operations at national, regional and local level and thus to ensure long- term implementation in daily practice.	MRC:<br>The rules for Medical-Pharmaceutical Concertation (MPC) were set by the publication of a Royal Decree, which aims to facilitate and encourage meetings concerning pharmacotherapy in which GPs and CPs participate in a constructive dialogue. As it is currently implemented, the initiative, which is also referred to as the MPC project, consists of two parts: local projects, and supporting programs. The local projects are meetings that are held between GPs and CPs on a geographical basis. Each meeting must be organized by at least one GP and one CP, henceforth referred to as the project leaders. The programs provide educational support for the local projects, such as materials and moderators. They are developed by the professional associations for GPs and CPs, universities, educational institutions and scientific organizations. Each program covers a topic related to the rational prescribing, rational dispensing and safe use of medication.<br><br>In several countries, more advanced formats of GP-CP collaboration are already in place. Practice pharmacists, for example, are active within general practices in Australia, USA and UK to perform tasks such as patient education and counselling, as well as medication reviews. Another example is that of the prescribing pharmacist in the UK. Pharmacist supplementary prescribing has been on the agenda since 2003, followed by pharmacist independent prescribing in 2006.	
59	Davis et al.	2020	Conversations about preventers in pharmacy.	Respirology.	Australia.	Qualitative study - semi-structured interviews.	Primary research.	Asthma.	Asthma medicine cost.	N/A	Obtain input from pharmacists regarding: cost-related adherence issues with asthma medicines; opportunities for conversations with patients; and the suitability of proposed pharmacy-based resources to address adherence barriers, including cost, for asthma medicines.	N/A		
60	Dineen-Griffin et al.	2019	Evaluation of a Collaborative Protocolized Approach by Community Pharmacists and General Medical Practitioners for an Australian Minor Ailments Scheme: Protocol for a Cluster Randomized Controlled Trial.	JMIR Research Protocols.	Australia.	Cluster randomized controlled trial.	Primary research.	Minor ailment.	Australian Minor Ailments Service (AMAS).	24 patients in intervention and controlled groups per pharmacy respectively (360 in total per group), aged 18 years or older, presenting to the pharmacy in person with a symptom-based or product-based request for one of the following ailments: reflux, cough, common cold, headache (tension or migraine), primary dysmenorrhea, or low back pain.	This trial aims to evaluate the clinical, economic, and humanistic impact of an Australian Minor Ailments Service (AMAS) compared with usual pharmacy care in a cluster randomized controlled trial (cRCT) in Western Sydney, Australia.	The Australian Minor Ailments Service (AMAS) is a practice model with key elements, such as agreed referral points, communication systems between pharmacists and general practitioners (GPs), and clinical treatment pathways, that is, HealthPathways. HealthPathways is a proprietary system of clinical pathways developed in New Zealand and adopted by clinicians throughout PHNs in Australia. The collaborative clinical pathways for each minor ailment (Table 1) are intended for use by community pharmacists delivering AMAS. Each ailment has the same structure and format to make the process of finding and using the information easy and practical. These pathways include types of questions, assessment, management approach recommending a particular course of action including self-care, and/or a nonprescription medicine for symptomatic relief, specific to each ailment. Included is a robust framework for referral, indicating red flag criteria to trigger escalation processes, and the time frame within which a patient is recommended to seek care from a particular health care provider (ie, the patient is recommended to see a GP within 24 hours). A red flag is a symptom that is recognized as likely to be of a more serious nature and requires immediate referral. The research and writing of these clinical pathways followed a literature review of contemporary international and national clinical guidelines in consultation with leading general medical professionals involved in PHN governance with comprehensive experience in HealthPathways development.	The literature internationally suggests that minor ailment services enhance the delivery of primary care, promote efficiencies, and reduce overall health care costs. Referrals (and importantly, red flag referrals) were a critical point that came up in the codesign process with GPs. GPs wanted to see patients quickly if there were any doubts and ensure patients are being referred in an appropriate and timely manner to the correct health provider.	
61	Dineen-Griffin et al.	2020	Primary health care policy and vision for community pharmacy and pharmacists in Australia.	Pharmacy Practice.	Australia.	N/A	Article.	N/A	Primary health care policy and vision for community pharmacy and pharmacists in Australia	N/A	To review primary health care policy, current pharmacy services and vision for community pharmacy and pharmacists in Australia.	N/A	In 2019, the Australian Government announced the development of a Primary Health Care 10-Year Plan which will guide resource allocation for primary health care in Australia. At the same time, the Government has committed to conclude negotiations on the 7th Community Pharmacy Agreement (7CPA) with a focus on allowing pharmacists to practice to full scope and pledges to strengthen the role of primary care by better supporting pharmacists as primary health care providers. The 7CPA and the Government's 10-year plan will largely shape the practice and viability of community pharmacy. It is essential that both provide a philosophical direction and prioritize integration, remuneration and resources which recognize the professional contribution and competencies of community pharmacy and community pharmacists, the financial implications of service roles and the retention of medicines-supply roles. Primary health networks – primary health care organisations<br>A policy response was the establishment of a network of primary health care organisations, known as PHNs. Thirty one PHNs were established across Australia,<br>This network was created to operate at a regional level with the authority and accountability to plan, integrate and coordinate primary health care at a local level.<br>PHNs work with primary care providers (primarily GPs), hospitals, and the broader community to:<br>* increase the efficiency and effectiveness of medical services for patients, particularly those at risk of poor health outcomes, and;<br>* improve coordination of care to ensure patients receive the right	

62	Disalvo et al.	2019	Pharmacists' perspectives on medication reviews for long term care residents with advanced dementia: a qualitative study.	International Journal of Clinical Pharmacy.	Australia.	Qualitative evaluation.	Primary research.	Advanced dementia.	Pharmacists' perspectives on medication reviews for long-term care residents with advanced dementia.	Patiets with advanced dementia.	To explore pharmacist perspectives of the Australian Government funded pharmacist-conducted residential medication management review and its role improving the quality and safety of prescribing in long-term care, in particular for those living with advanced dementia.	First initiated in 1997, the Residential Medication Management Review (RMMR) is an Australian Govern- ment funded service in which accredited pharmacists are remunerated to formally review permanent residents of government funded LTC facilities. The RMMR programme is a key component of the Australian Medi- cines Policy to achieve quality use of medicines (QUM) in LTC settings to prevent drug-related problems (DRPs), with a focus on general practitioner (GP) and pharmacist collaboration to reach these goals. The RMMR is intended to be a full clinical medication review including the following steps; (1) referral of the LTC resident by their GP, based on need or biennially, (2) accredited pharmacist gathers resident information from resident, family and/or carer, LTC staff, and/or case notes, (3) pharmacist provides medication review findings and recommendations in a written report for the GP within a reasonable time-frame of no more than 4 weeks from being initiated, and (4) post-RMMR discussion between pharmacist and GP. Retrospective audits of recommendations made by phar- macists following RMMRs have found that these identify DRPs and can reduce the median drug burden index by 12% from baseline if implemented by a GP. Their results demonstrate that medication review as a collaborative process—in this case GP cooperation—is essen- tial to reach positive medication management outcomes.<br>A meta-model of physician-community pharmacist collaboration (PCPC) was then adopted to refine themes. The PCPC model proposed by Bardet et al. concepualises trust and interdependence as core determinants of successful collaboration between physicians and commu- nity pharmacists. The forming of these core determinants depends on four processual determinants; perceptions, expectations, skills, and interest for collaborative practice. Finally, role definition and communication were conceptualised as tools to		
63	Dobson et al.	2006	Interprofessional health care teams: Attitudes and environmental factors associated with participation by community pharmacists.	Journal of Interprofessional Care.	Canada.	Quantitative study.	Primary research.	N/A	Attitudes and environmental factors associated with participation by community pharmacists in a primary health care team.	N/A	The purpose of this study was to determine whether community pharmacists are prepared to assume a more active role as members of the primary health care team. A secondary objective was to develop multi-items scales for future studies involving pharmacists, as well as other health professions.	N/A	In Canada, community pharmacists have traditionally practiced in privately-own businesses with remuneration provided by both private and public payers for a limited range of professional services. However, as health reforms push the Canadian health care system toward greater use of community-based models of care, community pharmacists will face increasing expectations to work with other health care disciplines in a more coordinated manner	
64	Pasquier & Aslani.	2008	Concordance-based adherence support service delivery: consumer perspectives	Pharmacy World & Science.	Australia.	Qualitative study.	Primary research.	N/A	Patient perspectives on concordance-based adherence support service delivery.	Three focus group discussions with consumers on chronic therapy (n = 22) and two focus groups with consumer representatives (n = 15) were conducted in 2002.	To explore consumers' attitudes towards, and expectations of, adherence support services in primary health care, specifically in community pharmacy; and to explore consumers' attitudes towards the concept of concordance	Concordance refers to an agreement between two people. In the context of health care, it means developing a partnership between the patient and health care provider (doctor or pharmacist), where you can explain your views about your illness and therapy, but also about your personal situation, and where you are encouraged to make a decision in relation to your health and therapy	This paper is focused on patient-GP/patient-CP relationship rather than CP-GP relationship.	
65	Duban et al.	2017	Identifying the interprofessional agreement between community pharmacist's and general practitioner's views on collaborative practice in Slovakia.	Ceska a Slovenska farmacie.	Slovakia.	Quantitative study.	Primary research.	N/A	Interprofessional agreement between community pharmacist's and general practitioner's views on collaborative practice in Slovakia.	N/A	The objective of this study is to identify the current level of GPs and CPs teamwork in Slovakia and to identify the key factors, where these professions agree.	N/A	CPs in Slovakia still perform technical roles such as dispensing and compounding. The GPs are responsible for traditional medical competencies, such as diagnosing patients and prescribing medication. The majority of interactions between CPs and GPs in Slovakia is based only on solving administrative issues.	
66	Edmunds & Calnan.	2001	The reprofessionalisation of community pharmacy? An exploration of attitudes to extended roles for community pharmacists amongst pharmacists and General Practioners in the United Kingdom.	Social Sience & Medicine.	UK.	Qualitative study.	Primary research.	N/A	Attitudes to extended roles for community pharmacists amongst pharmacists and General Practioners in the United Kingdom.	N/A	Analysis of community pharmacists' and GPs' perceptions of projects involving extending community pharmacy's role, this study seeks to consider the explanatory power of the neo-Weberian position on professionalism and reprofessionalisation. It looks at whether pharmacists are expanding their role for professional (job satisfaction), business, political (survi- val or expansionist) or altruistic reasons. It also considers whether doctors are encouraging these in- itiatives or resisting them and for what reasons (altruistic, self interested or political).	In 1997, the Department of Health sponsored a series of pilot projects aimed at testing some form of extended role for community pharmacists in the UK. Each of these projects had their own local project managers and evaluators. However,the role of this study was to provide an overall evaluation of all of the projects, looking specifically at questions of acceptability and feasibility. While they differed in some respects, all of the projects involved a departure from a remit that centred on dispensing to one that included more patient-oriented activities, such as reviewing patients for side effects, abilities to take medicines properly, and in two cases, moni- toring patients suffering from hypertension and heart disease.	It is possible that the new PCGs could play a part in this because evidence from other countries, such as The Netherlands, suggests that similar bodies have provided a basis for better communication between doctors and pharmacists. In The Netherlands, the government and the Dutch equivalent of the RPSGB promoted the establishment of consultation groups consisting of GPs and pharmacists who met at two- monthly intervals to discuss treatment and prescribing. (Mason, P. (1998). Pharmacy in the Netherlands: Five years on. The Pharmaceutical Journal, 261, 633–635.)	
67	Egunsola et al.	2022	A Qualitative Systematic Review of Facilitators of and Barriers to Community Pharmacists–Led Anticoagulation Management Service.	Annals of Pharmacotherapy.	Canada, UK, New Zealand, Australia.	Qualitative evaluation.	Secondary research.	Anticoagulation therapy.	Community Pharmacists–Led Anticoagulation Management Services (CPAMS).	Patients on anticoagulation therpay and require INR monitoring.	The purpose of this systematic review is to identify the facil- itators of and the barriers to the implementation of CPAMS across different jurisdictions.	Across jurisdictions, patients were eligible to enroll in a CPAMS program after referral from their physicians. These were generally medically stable patients without significant comorbid conditions. The community pharma- cists were empowered to prescribe warfarin, monitor INR with a point-of-care device, and adjust warfarin dosing. Whereas the Canadian and New Zealand programs were primarily driven by provincial and national health agencies, respectively, the Brighton and Hove program in the United Kingdom was facilitated by a pharmacy chain in collaboration with independent pharmacies. In all jurisdictions, physicians continued to provide clinical support for difficult and complicated cases through established communication channels.		
68	El-Awaisi et al.	2018	Perspectives of practising pharmacists towards interprofessional education and collaborative practice in Qatar.	International Journal of Clinical Pharmacy.	Qatar.	Mixed-method study.	Primary research.	N/A	Perspectives of practising pharmacists towards interprofessional education and collaborative practice in Qatar	N/A	To explore the awareness, views, attitudes and perceptions of practising pharmacists in Qatar towards interprofessional education and collaborative practice.	N/A		
69	El-Awaisi et al.	2018	A comprehensive systematic review of pharmacy perspectives on interprofessional education and collaborative practice.	Research in Social and Administrative Pharmacy.	United States (11), Australia (4), Canda (3), UK & Northern Ireland (2), Spain (2), Germany (1), New Zealand (1), Singapore (1), Qatar (1), Saudi Arabia (1).	Mixed-method study.	Secondary research.	N/A	Pharmacy perspectives on interprofessional education and collaborative practice.	N/A	The aim of this systematic review is to synthesise, summarise and evaluate the quality of the quantitative and qualitative literature related to the perspectives of pharmacy students, pharmacy faculty and practising pharmacists toward IPE and collaborative practice. The perspectives included their views, experiences and at- titudes with a special focus on their perceived benefits and challenges in relation to IPE and collaborative practice.	N/A		
70	Evans et al.	2016	National community pharmacy NHS influenza vaccination service in Wales: a primary care mixed methods study.	British Journal of General Practice.	UK.	Mixed-method evaluation.	Primary research.	N/A	NHS influenza vaccination service in Wales.	N/A	To explore and verify the factors that influence the relative performance of pharmacies providing NHS influenza vaccinations.	Pharmacy bodies in England have called for community pharmacies to take on aspects of GP workload as a potential solution to the workload pressures faced by GPs. Before 2015, Wales was the only part of the UK to have a national pharmacy influenza service.	In July 2015, NHS England announced its intention to commission a national NHS influenza vaccination service from community pharmacies. The move has been controversial and, although it has been welcomed by pharmacy representatives, it has been met with resistance from those representing GPs. The latter have expressed concern about the adverse financial impact that pharmacy provision could have on GP practices.	
71	Gallagher & Gallagher.	2012	Improving the working relationship between doctors and pharmacists: is inter-professional education<br>the answer?	Advances in Health Sciences Education.	Ireland.	Reflection.	Secondary research.	N/A	Improving working relationship between doctors and pharmacists in Ireland.	N/A	Insights into the types of factors that help or hinder the improvement of these working relationships and the importance of education and agreed working practices in defining the separate but inter-dependent professions of pharmacy and medicine are reviewed and discussed.	N/A		
72	Gerlach et al.	2020	Professional roles of general practitioners, community pharmacists and specialist providers in collaborative medication deprescribing - a qualitative study.	BMC Family Practice.	Germany.	Qualitative study.	Primary research.	N/A	Professional roles of general practitioners, community pharmacists and specialist providers in collaborative medication deprescribing.	N/A	Explore GPs', pharmacists' and specialist providers' perceptions of professional roles in deprescribing with a focus on inter-professional collaboration for medication optimization.	N/A	In Germany, work overload is persisting among primary-care practitioners. Still, numbers of practicing GPs are exceeded by those of community specialists (CSs), who independently work in outpatient secondary- care practices. GPs in Germany have limited gate- keeping functions and their interaction with CSs con- centrates on referrals which, however, rarely are compulsory. Hence, community-specialist services not only are highly accessible for patients, but also reflect great therapeutic autonomy such as for medication provision. Even community pharmacists' (CPs') services are easily accessible and free-of-charge. Yet, in Germany, only physicians may prescribe medications.	
73	Gidman et al.	2012	Understanding public trust in services provided by community pharmacists relative to those provided by general practitioners: a qualitative study.	BMJ Open.	UK.	Qualitative study.	Primary research.	N/A	Understanding public trust in services provided by community pharmacists relative to those provided by general practitioners.	A total of 26 people participated in one of five focus groups in the vicinity of Glasgow, Scotland. Participants were recruited through non-pharmacy or national health-related voluntary and charity organisations.	To apply sociological theories to understand public trust in extended services provided by community pharmacists relative to those provided by general practitioners (GPs).	Hall et al proposes that trust comprises five dimen- sions. These are fidelity, competence, honesty, confi- dentiality and global trust. Fidelity relates to putting the patients' interests above personal interests. This implies respect, care and avoiding conflicts of interest. Competence relates to avoiding errors and achieving optimal outcomes. Patients have difficulty in judging technical competence and assessments of this aspect relate strongly to practitioners' communication skills. Honesty entails telling the truth. Confidentiality involves protecting sensitive information. Global trust relates to the less easily categorised holistic aspects of trust, which cannot be easily described but are linked to all other aspects of trust. (Hall M, Dugan E, Zheung B, et al. Trust in physicians and medical institutions: what IS IT, can it be measured, and does it matter? The Millbank Quaterly 2001;79:613e39.)	In Scotland, general practitioners (GPs) and community pharmacies operate as independent contractors providing a range of services within the NHS. In some cases, GPs charge for certain services or products not funded by the NHS, for example, there may be charges for some travel vaccinations.<br>- Pharmacists in Scotland provide dispensing services, as well as a chronic medication service, minor ailment services, public health services, acute medication services, flu vaccination supply and some locally commissioned services (these include advice to residen- tial homes, methadone supply, needle exchange and domiciliary oxygen).<br>- The pharmacy services listed above are funded by the NHS, and there is no charge to the patient. Prescription charges were abolished in Scotland in 2011.<br>- Most people are not registered with a pharmacist, unless a specific service has been used, which requires registration, examples include the minor ailment service or chronic medication service.<br>- A number of non-NHS services are available at specific pharmacies at a charge, in Scotland, including weight management and seasonal flu vaccination.<br>- Community pharmacies in Scotland tend to be operated by commercial operations in retail settings. Most generate profit from the sale of medicines, medical equipment and other sundry items.	
74	Gidman & Cowley.	2013	A qualitative exploration of opinions on the community pharmacists' role amongst the general public in Scotland	International Journal of Pharmacy Practice	UK.	Qualitative study.	Primary research.	N/A	Opinions on the community pharmacists' role amongst the general public in Scotland.	Five focus groups were conducted with 26 members of the public resident in Scotland in March 2010	To understand members of the public's opinions and experiences of pharmacy services	N/A		
75	Gordan et al.	2018	Community pharmacist-led clinical services: physician's understanding, perceptions and readiness to collaborate in a Midwestern state in the United States.	International Journal of Pharmacy Practice.	United States.	Qualitative study.	Primary research.	N/A	Physician's understanding, perceptions and readiness to collaborate in Community pharmacist-led clinical services.	N/A	Identify general and family practice physicians' awareness of pharmacists' delivery of clinical services, uncover their perceived barriers to collaboration with community pharmacists, and collect their input on how to overcome such barriers in order to better understand how pharmacist-led clini- cal services can be integrated, improved and more widely utilized as a health- care delivery mechanism.	Many of the pharmacist-led clinical services, such as anticoagulation clinics or hypertension management programmes, are delivered in collaboration with physicians through collaborative practice agreements (CPAs), while some other services are delivered via pharmacist prescribing authority or statewide protocols such as naloxone, hormonal contraceptives, smoking cessation and immunizations.	New and innovative CPA services, such as point-of-care testing for group A strepto- coccus and influenza with respective per-protocol dispensing of anti-infective medications, are being implemented across the country. Collaboration between pharmacists and physicians is an essential step in expanding these pharmacist-led services and achieving the expected outcomes and will be instrumental in making the team-based approach to health care a standard of<br>practice. Although there have been important and successful initiatives to recognize pharmacists within the model of team-based care, some barriers remain to make collaboration a commonly accepted and valued practice.	
76	Hasan et al.	2015	Physicians' attitudes towards provision of primary care services in community pharmacy in the United Arab Emirates.	International Journal of Pharmacy Practice.	United Arab Emirates.	Qualitative study.	Primary research.	N/A	Physicians' attitudes towards provision of primary care services in community pharmacy.	N/A	This study aims to explore physicians' views of pharmacists' roles in providing primary care services through community pharmacies in the United Arab Emirates (UAE)	N/A	Pharmacists' primary care services including screening and monitoring of disease, health advice, referral, lifestyle and preventive care, supply of printed information, counselling on medications, patient record keeping, and pharmacist intervention in chronic disease management	
77	Hasan et al.	2018	Physicians' perspectives of pharmacist-physician collaboration in the United Arab Emirates: Findings from an exploratory study.	Journal of Interprofessional Care.	United Arab Emirates.	Qualitative study.	Primary research.	N/A	Physicians' perspectives of pharmacist-physician collaboration in the United Arab Emirates.	N/A	The purpose of this study is to investigate physicians' opinions on collaborative relationships with community pharmacists in the UAE.	N/A	Participants suggested that an in-house set-up or a system with a pharma- cist integrated into the practice could improve communica- tion between pharmacists and physicians and allow immediate resolution of patient drug therapy problems. However, Bradley et al. (2008) reported that an in-house effect could be achieved through regular face-to-face contact between healthcare professionals who are not co-located. It would seem that if collaboration as an interprofessional rela- tion between community pharmacists and physicians is diffi- cult to achieve in the UAE, interprofessional coordination may seem as an alternative. Coordination is seen as similar to collaboration as far as shared identity, shared accountability between individuals and clarity of roles, tasks, and goals. However, integration and interdependence are viewed as less important, team tasks are regarded as more predictable, less urgent and less complex than collaboration (Reeves et al., 2018). This suggests that it would still be feasible for pharma- cists in the UAE to maintain their independent community practice while engaging in coordinated relationships with physicians, a set-up that many pharmacists may prefer. (Reeves, S., Xyrichis, A., & Zwarenstein, M. (2018). Teamwork, collabora- tion, coordination, and networking: Why we need to distinguish between different types of interprofessional practice. Journal of Interprofessional Care, 32, 1–3.) Interestingly, a promising study assessing patient willingness to use services provided by pharmacists in the UAE had actually shown that participants were interested in accessing enhanced services by pharmacists such as receiving informa- tion about medications, help in self-	
78	Hatah et al.	2012	General practitioners' perceptions of pharmacists' new services in New Zealand.	International Journal of Clinical Pharmacy.	New Zealand.	Qualitative study.	Primary research.	N/A	General practitioners' perceptions of pharmacists' new servi	N/A	To examine GPs' perceptions of pharmacists' new services. Specific objectives of this study are to compare the perceptions of GPs with different backgrounds and to identify any perceived barriers to the new services provided or proposed by community pharmacists.	N/A	MUR was implemented in 2007 under local contracts with individual District Health Boards (DHBs). Community pharma- cists need to be accredited and they are reimbursed for providing the services.	
79	Hindi et al.	2019	Community pharmacy integration within the primary care pathway for people with long-term conditions: a focus group study of patients', pharmacists' and GPs' experiences and expectations.	BMC Family Practice.	UK.	Qualitative study.	Primary research.	Asthma, COPD, Type 2 diabates.	Patients', pharmacists' and GPs' experiences and expectations on integration of community pharmacy services within the primary care pathway for people with long-term conditions (LTCs).	Two focus groups were conducted with respiratory patients (n = 6, 5) and two with type 2 diabetes patients (both n = 5).	Use marketing theory to examine the views of patients, pharmacists and general practitioners (GPs) on how community pharmacies are currently used and to identify how community pharmacy services may be better integrated within the primary care pathway for people with long-term conditions (LTCs).	It has been argued that the use of marketing theories in public sectors could provide a better understanding of how these organisations could meet the expectations of their target population. Moreover, previous studies have demonstrated the applicability of marketing theories to shed light on factors which influence the demand and uptake of public sector services. Therefore, marketing theories may be applied to primary healthcare and, specifically, the community pharmacy context to provide valuable insights such as: identifying stakeholders' needs and preferences, understanding factors that influence service uptake, and exploring how services could meet quality standards. The "7Ps marketing mix" which was used in this study has been considered as one of the cornerstones of marketing theory. The 7Ps consists of seven components ("product", "price", "place", "promotion", "people", "process" and "physical evidence") that an organisation should account for to successfully market their product or service to target customers. The 7Ps are based on understanding what consumers want/need from a service whilst accounting for the influence of service design, service delivery and external communications on consumers' perceptions of services. Evidence has shown that the 7Ps can be applied to organisations providing public services		
80	Hindi et al.	2022	Using communities of practice as a lens for exploring experiential pharmacy learning in general practice: Are communities of practice the way forward in changing the training culture in pharmacy?	BMC Medical Education.	UK.	Quantitative evaluation.	Primary research.	N/A	Experiential pharmacy learning in general practice.	N/A	To apply educational theories to explore pharmacy stakeholders' perceptions of placements in general practice and contribute to the development of a model of experiential learning for pharmacy.	Lave and Wenger's 'communities of practice' (CoP), which focuses on the relationship between learning and the social situation in which it occurs, provides this paper's theoretical underpinning. CoP conceptualises that learners participate in 'communities of practice' and acquire knowledge and skills through newcomers moving from 'legitimate peripheral participation' toward 'full participation' in the sociocultural practice of a community. Novices begin by observing and performing basic tasks at the periphery. Through active participation and engagement they become more skilled, assume more responsibility and therefore move into a more central position within the community. Given that learning in CoP is viewed as a series of collective, relational and social processes rather than individual acquisition of knowledge considering pharmacy placements in general practice as CoPs could help to uncover the learning processes that take place within that sector.	This paper explored experiential learning in general practice, with learners and their GP based supervisors. Early exposure to general practice, role models, and scope of practice have been shown to contribute to healthcare students selecting careers in general practice, arly pharmacy placements in general practice may encourage pharmacists to consider roles in general practice from early in their careers.	
81	Hossain et al.	2017	Qualitative meta-synthesis of barriers and facilitators that influence the implementation of community pharmacy services: perspectives of patients, nurses and general medical practitioners.	BMJ Open.	Australia.	Qualitative meta-synthesis.	Secondary research.	N/A	Barriers and facilitators of implementation of community pharmacy service Australia.	N/A	To synthesise the literature on patients', general practitioners' (GPs) and nurses' perspectives of CPSs to identify barriers and facilitators to their implementation Australia.	N/A		
82	Hossain et al.	2018	A multilevel stakeholder approach for identifying the determinants of implementation of government-funded community pharmacy services at the primary care level.	Research in Social and Administrative Pharmacy.	Australia.	Qualitative study.	Primary research.	N/A	Determinants of T implementation of government-funded community pharmacy services at the primary care level.	N/A	To identify key determinants of practice that can influence the implementation of government-funded CPSs in a primary health network in Australia.	In Australia, Primary Health Networks (PHNs) are independent or- ganizations that aim to improve the effectiveness and efficiency of health services for patients by supporting and coordinating primary health care at a community level. Community pharmacy services (CPSs) are health facilities at the primary care level. CPSs can make a valuable contribution in improving patients' healthcare, particularly in the management of chronic disease. It has been sug- gested that insufficient knowledge regarding implementation, and lack of holistic implementation programs may be an influencing factor. A recent systematic review highlighted the importance of including the views of relevant ground-level stakeholders, such as patients, general practitioners (GPs) and nurses23 to complement and extend the phar- macist-centred literature on CPS implementation factors. All of these key stakeholders can strongly influence the implementation of CPSs at the primary care level. Therefore, they must be included alongside other stakeholders to navigate the complex healthcare system in which CPSs are to operate and thus facilitate their implementation.	The use of participatory approaches that involve multiple stake-holders to identify determinants of practice, and develop tailored in- terventions to address these determinants, have been widely used in health research.	

83	Hughes & McCann.	2003	Perceived interprofessional barriers between community pharmacists and general practitioners: a qualitative assessment.	British Journal of General Practice.	UK.	Qualitative study.	Primary research.	N/A	Perceived interprofessional barriers between community pharmacists and general practitioners: a qualitative assessment.	N/A	To explore barriers between the two professions in relation to closer interprofessional working and the extension of prescribing rights to pharmacists.	N/A		
84	Jankovic.	2017	The importance of improving GP and community pharmacy relations.	the Pharmaceutical Journal.	UK.	Review article.	Report.	N/A	GP and community pharmacy relationships.	N/A	To review current challenges in building collaborative relationship between CP and GP and how to overcome them.	N/A		
85	Jenkins et al.	2016	Too far away to work with each other: Does location impact on pharmacists' perceptions of interprofessional interactions?	Journal of Interprofessional Care.	UK.	Quantitative study.	Primary research.	N/A	location impact on pharmacists' perceptions of interprofessional interactions.	N/A	To determine whether these different primary healthcare 'spaces' impact on the frequency of interprofessional interactions	The notion of 'space' remains under-conceptualised and has most commonly been explored in the context of a patient's experience of healthcare. The theory would suggest, however, that when HCPs are divorced by location, there is a barrier to such interactions		
86	Jove et al.	2014	Perceptions of collaboration between general practitioners and community pharmacists: findings from a qualitative study based in Spain	Journal of Interprofessional Care.	Spain.	Qualitative study.	Primary research.	N/A	Perceptions of collaboration between general practitioners and community pharmacists.	N/A	The aim of this study was to assess the perceptions of GP–CP collaboration from these professionals' perspectives. One way to approach this issue is through qualitative research methodology which permits exploration, awareness and understanding of GPs and CPs' opinions and motivations.	In accordance with the theory of reasoned action (TRA), when a person forms beliefs about an action, in this case, initiating collaboration, the person automatically acquires an attitude towards it. This attitude influences the intention to carry out this action and the final behavior. Based on this theory, the importance of understanding GPs and CPs' perception of usefulness is clear as this will affect their attitude towards collaboration and, consequently, their intention to collaborate and to carry it out. GP and CP's belief that collaboration will have positive effects (e.g. as improved medi- cation adherence or use of cost-effective pharmaceuticals) is a strong predictor of attitude, intention and willingness to work in collaboration with each other.	The main difference between the two regions is the degree of commitment to primary care which is greater in Catalonia than in the Balearic Islands. As a consequence, the investment of resources in primary care with respect to total health expenditure is higher in Catalonia. Accordingly, the density of Primary Care Health Centres (PCHCs), the access to diagnostic procedures by GPs and the number of services integrated within primary care is higher in Catalonia than in the Balearic Islands while the ratio of patients per GP is lower. Another difference between the two regions relates to the stage of implementation of electronic prescribing. The main objective of electronic prescribing was to link the GP's prescriptions with the pharmacy. However, it can be used as a communication channel between the pharmacist and the GP and it has been the first formal electronic link between GPs and CPs in Spain. The Balearic Islands government initiated its implementation in 2006, long before its introduction in Catalonia. Thus electronic prescription had long been established in the Balearic Islands by the time we conducted the interviews, while in Catalonia it was being used exclusively in rural areas and small cities, excluding Barcelona where the interviews were conducted.	
87	Kelly et al.	2013	Pharmacist and physician views on collaborative practice: Findings from the community pharmaceutical care project.	Canadian Pharmacists Jounral.	Canada.	Quantitative study.	Primary research.	N/A	Pharmacist and physician views on collaborative practice.	N/A	The objective of this study was to capture the opinions of family physicians and community pharmacists in Newfoundland and Labrador (NL) regarding collaborative practice.	N/A	It has been suggested that the belief of physicians that collaboration will result in improved medication adherence may strongly influence their likelihood of collaborating with community pharmacists. Until recently, in NL as well as most other provinces in Canada, pharmacist services reimbursed by public payers were primarily those associated with dispensing prescriptions. Pharmacists' desire to apply their knowledge and skills to enhance patient care is hampered in an environment in which remuneration is tied to the dispensing of a product, rather than for services rendered. A recent analysis of remuneration models for pharmacy professional services indicates that the method of remuneration does appear to influence the provision of such services, noting that in countries where pharmacists are paid a flat fee to cover all services provided, there is a lack of incentive to provide more or higher- quality service.	
88	Kember et al.	2018	The public's perception of the role of community pharmacists in Wales.	International Journal of Pharmacy Practice.	UK.	Qualitative study.	Primary research.	N/A	The public's perception of the role of community pharmacists in Wales	Recruitment took place within a ten-mile radius of a large urban town in North East Wales, using quota sampling to identify four different social groups (i.e., sixth-form pupils from a local secondary school, a young adult group, an older adult group and a local community group) to repre- sent the general public. In total, there were 32 participants across five FGs; 14 were male and 18 females, ranging from 16 to 81 years of age. Apart from the school pupils and university students (n = 9), the majority of the sample were in the B, C1 and C2 socio-economic groups (B – intermediate managerial, administrative, professional; C1 – supervisory, clerical, junior managerial; C2 – skilled manual workers).	To investigate the general public's perceptions of the community pharmacist's (CP) role in Wales by exploring understanding, awareness of ser- vices provided and potential interventions for promoting the role of CPs.<br>1 To explore the public awareness of the role of the CP.<br>2 To establish what influences the public's awareness of the CP's roles and to identify which services provided by CPs the public are currently accessing.<br>3 To explore which services the public would use when made aware of their availability.<br>4 To canvas opinion on the potential interventions for raising public awareness of the role of CPs and the ser- vices offered by them.	N/A	The first pharmacy contractual framework was launched in 2005 in England and Wales (with similar services also available in Scotland). It consists of three different service levels: Essential, Advanced and Enhanced. This includes services such as disposal of unwanted medicines; promo- tion of healthy lifestyles; signposting (referral to other sources of professional or alternative providers for sup- port); medicines use review (MURs); discharge medication review (DMR) (Wales only); new medicine service (NMS) (England only); and vaccination services. The aim of the new contract was to make better use of the skills and expertise of CPs and their staff; to promote community pharmacies as an integral part of the National Health Ser- vice (NHS) organisation; support healthcare and tackle health inequalities; and support self-care.	
89	Lake et al.	2020	Understanding perceptions of involving community pharmacy within an integrated care model: a qualitative study.	BMC Health Services Research.	Canada.	Qualitative study.	Primary research.	N/A	Perceptions of involving community pharmacy within an integrated care model.	N/A	This study explored stakeholder perceptions and enablers of including community pharmacy within an integrated care model.	In 2012, an integrated care model called the Health Links Approach to Care was instituted in Ontario, Canada (most populous province). As per Goodwin (2016), the Health Links Approach to Care was an example of integration at an organizational level both horizontally and vertically. Specifically, the integration was designed to create care net- works (horizontal integration), as well as integration across different health care settings (vertical integration) [30] for a subgroup of people with complex health and so- cial needs. The target population within the Health Links Approach to Care was "high cost users", defined as Ontar- ians who have the highest health care expenditures ("top 5%") in publicly funded services during the previous fiscal year. The ra- tionale for targeting these patients within the Health Links Approach to Care was to improve quality of care and to maintain or reduce health care costs and spending.<br>The Health Links Approach to Care was implemented across Ontario, Canada. The Ministry of Health and Long-Term Care adopted a "low-rules" approach to im- plementation. Each site was predicated on organiza- tions working within their geographical area to design local solutions, network health care and social ser- vices, and provide care to persons with high health care costs. The only organizational parameters mandated by the Ministry of Health and Long-Term Care were the inclusion of an appropriate mix of health care providers, including at least 65% of the family physicians in the geographical area, and the ability to track and manage persons with high- cost.		
90	Lalonde et al.	2011	Physician-pharmacist collaborative care in dyslipidemia management: The perception of clinicians and patients	Research in Social and Administrative Pharmacy.	Canada.	Qualitative study.	Primary research.	Dyslipidemia.	The perception of clinicians and patients on physician-pharmacist collaborative care in dyslipidemia management.	66 patients were selected based on their coronary heart disease risk (moderate vs high), age (O vs % the median age of 60 years), and status at the end of pharmacist follow-up (target lipid levels reached vs above tar- get lipid levels).	In a qualitative study nested within the TEAM study, the perceptions of physicians, pharmacists, and patients regarding the PPCC model, interprofessional collaboration, and the clinicians' willingness to implement the model in their practice were explored.	Clusters of primary care physicians and community pharma- cists were recruited and randomized to the PPCC or the UC group. PPCC pharmacists participated in a 1-day workshop to prepare them to apply the intervention. Physicians were responsible for making the diagnosis and for prescribing lifestyle changes and statin treatment. Pharmacists then monitored lifestyle changes, tolerance, efficacy, and adherence to pharmacotherapy; they were responsible for requesting laboratory analyses and adjusting statin dosage accordingly. PPCC in- tervention ended when target lipid levels were reached for at least 3 months. The duration of the pharmacist follow-up varied from patient to patient and ranged from 5 to 12 months. After each visit, the pharmacist faxed the physician a re- port form summarizing the intervention. In the UC arm, the physicians adjusted lipid-lowering pharmacotherapy and pharmacists provided the usual counseling and dispensed medications. When compared with the UC, PPCC patients had more health professional visits and laboratory tests, were more likely to have their lipid-lowering treatment changed, and more likely to report lifestyle changes. However, after 1 year, this did not translate into clinically significant between- group differences in lipid control.		
91	Latif et al.	2013	Medicines use reviews: a potential resource or lost opportunity for general practice?	BMC Family Practice.	UK.	Qualitative study.	Primary research.	N/A	MURs	Patients receiving MURs.	This paper presents the patient's perspective of the MUR service and focuses on the importance of GP-pharmacist collaboration for patient care. Suggestions on how MURs may have value to GPs through the delivery of increased patient benefit are discussed.	N/A	Although pharmacy staff tended to invite patients with less complicated medicine regimes, there were a few instances during the MUR where the pharmacist identified particular concerns about the patient's health or medicine. In these cases, rather than inter- vene directly and on the patient's behalf, pharmacists preferred to place the onus on patients to return to their GP for resolution of these issues. In the follow- ing example the pharmacist identifies a potential de- terioration in the patients' asthma control. Whilst this event may not have been highlighted unless the pharmacist had initiated the MUR, the limited remit and lack of collaboration with the GP led to the pharmacist closing off discussion and failing to ensure that the issue was resolved	
92	Laubscher et al.	2009	Collaboration between family physicians and community pharmacists to enhance adherence to chronic medications Opinions of Saskatchewan family physicians.	Canadian Family Phycisian.	Canada.	Quantitative study.	Primary research.	N/A	Collaboration between family physicians and community pharmacists to enhance adherence to chronic medications.	N/A	To ascertain the opinions of family physicians about medication adherence in patients with chronic diseases and the role of community pharmacists in improving adherence to chronic medications, as well as their opinions on increased collaboration with pharmacists to enhance medication adherence.	N/A		
93	Lee et al.	2018	What role could community pharmacists in Malaysia play in diabetes self-management education and support? The views of individuals with type 2 diabetes.	International Journal of Pharmacy Practice.	Malaysia.	Qualitative study.	Primary research.	Type 2 Diabetes.	Role of community pharmacists in Malaysia in diabetes self-management education and support.	Fourteen participants with T2D were interviewed.	This study explored the experiences and views of individuals with type 2 diabetes mellitus (T2D) on their diabetes self-management and potential roles for community pharmacists in diabetes self-management education and support (DSME/S) in Malaysia.	In Malaysia, community pharmacies do not provide structured diabetes self-man- agement education and support (DSME/S).		
94	Loffler et al.	2017	Perceptions of interprofessional collaboration of general practitioners and community pharmacists - a qualitative study.	BMC Health Services Research.	Germany.	Qualitative study.	Primary research.	N/A	Perceptions of interprofessional collaboration of general practitioners and community pharmacists.	N/A	This qualitative study aims at investigating pharmacists' and general practitioners' views on barriers to interprofessional collaboration in the German health care system.	N/A		
95	Lucas & Blenkinsopp.	2015	Community pharmacists' experience and perceptions of the New Medicines Service (NMS)	International Journal of Pharmacy Practice.	UK.	Qualitative study.	Primary research.	N/A	Community pharmacists' experience and perceptions of the New Medicines Service (NMS).	N/A	The study aims to explore community pharmacists' experiences and per- ceptions of NMS within one area of the United Kingdom.	The introduction of a New Medicine Service (NMS) in England in October 2011 was designed to help improve medicines' adherence based on proof of concept and randomised controlled trials of pharmacist-conducted tele-phone consultations. The first of its kind, the state-funded service is provided by community pharmacists as part of their NHS Community Pharmacy Contractual Framework (CPCF). The NMS' primary objectives are to improve patient adherence and increase patient engagement with their condition. Other anticipated benefits included sup- plementation and reinforcement of information provided by the patient's general practitioner (GP) and practice staff to help patients make informed choices about their care. By implication, the service is intended to promote multidisci- plinary working with the patient's primary care medical practice.<br>The NMS consists of three stages: patient engagement, intervention and follow-up, and involves multiple contacts with the patient. Patients are recruited to the service by pre- scriber referral or opportunistically by the community phar- macy team. Initial advice is given to the patient about medicine usage and may include advice on healthy lifestyles. The second stage is an 'intervention' consultation, with a semi-structured interview to assess adherence, identify prob- lems and any need for information and support. The final stage involves a follow-up consultation. The patient can choose to have the intervention and follow-up by returning to the pharmacy or by telephone. The initial remuneration model for NMS included targets based on the pharmacy's volume of dispensing and national estimates of the percent- age of medicines newly prescribed, and a payment mecha- nism dependent on completion of all three stages for each patient. A revised and more flexible remuneration structure was introduced in May 2012, 8 months after the service began.		
96	Luetsch & Scuderi.	2020	Experiences of medical dominance in pharmacist-doctor interactions - An elephant in the room?	Research in Social and Administrative Pharmacy.	Australia.	Qualitative study.	Primary research.	N/A	Pharmacists' experiences of medical dominance in pharmacist-doctor interactions.	N/A	To investigate whether and how pharmacists may experience medical dominance in their practice.	Pharmacists in primary care mainly encountered general practi- tioners (GP), who seemed more senior or experienced in their practice than the pharmacists participating in this study. Most encounters and experiences of MD evolved around resolving issues with prescriptions presented by patients in community pharmacies.		
97	Marques et al.	2018	Pharmacist joint-working with general practices: evaluating the Sheffield Primary Care Pharmacy Programme. A mixed- methods study.	BJGP Open.	UK.	Mixed-method evaluation.	Primary research.	N/A	Evaluating the Sheffield Primary Care Pharmacy Programme.	N/A	To investigate (a) challenges of integration of CPs in general practices, and (b) the perceived impact on care delivery and community pharmacy practice.	One UK city, Sheffield, decided to deploy local CPs in all general practices; a programme known as the PCPP. The aim was to redistribute GPs' workload and enhance patient care over a 12-month period by providing support with (a) repeat prescriptions, (b) medicines reviews, (c) liaison with community pharmacies, (d) support to patients with long-term conditions on complex medicines regimens, and (e) support to care home patients. The PCPP was the first to integrate CPs in general practice at scale.	Benefits of CP–GP joint-working were particularly evidenced in cases where medicines-related tasks were seen as complex by GPs, difficult to complete in a timely way, or not prioritised because of competing workloads. CPs' medicines-focused skills and knowledge meant that these tasks were not viewed by them as complex, highlighting existing scope for role substitution between GPs and practice-based pharmacists, to enable better use of available GP time and more timely completion.	
98	Mercer et al.	2018	Physician and Pharmacist Medication Decision-Making in the Time of Electronic Health Records: Mixed-Methods Study.	JMIR Human Factors.	Canada.	Mixed-method study.	Primary research.	N/A	Integrated electronic health records (EHRs) in supporting interprofessional shared decision-making for medication therpay management.	N/A	The objective of our study was to examine how physicians and pharmacists understand and communicate patient-focused medication information with each other and how this knowledge can influence the design of EHRs.	Critical information is not shared, even when an electronic health record (EHR) is used, and the decision to prescribe or not prescribe, to take or not take a medication, is made with missing or distorted information. A medication-related decision involves, at minimum, a patient, a prescriber, and a pharmacist, and all parties are engaged in a process of shared decision-making (SDM). SDM is based on a model of communication where HCPs and patients both contribute to clinical decisions in unique ways. The HCPs share information about the benefits and risks of different treatment options; the patients describe their preferences and values as they relate to their treatment options. Interprofessional shared decision-making (IP-SDM) involves multiple HCPs and is emerging as a response to care increasingly being delivered by interprofessional teams to collaboratively work with a patient to decide on the best course of action.		
99	Mubarak et al.	2021	A Conceptual Framework of the Way Forward to a Community Pharmacist-General Practitioner Collaborative Medication Therapy Management Model for Chronic Diseases in Malaysian Primary Care: A Qualitative Study.	Risk Management and Healthcare Policy.	Malaysia.	Qualitative study.	Primary research.	N/A	Community Pharmacist-General Practitioner Collaborative Medication Therapy Management Model for Chronic Diseases in Malaysian Primary Care.	N/A	To conceptualize a stakeholder-driven framework to improve collaboration between CPs and GPs in Malaysian primary care to effectively manage medicines in chronic diseases.			
100	Myers & Roach.	2018	New Models of Care Supporting General Practice through Community Pharmacy.	N/A	UK.	Review article.	Report.	N/A	Integrated care model.	N/A	To review Jaunty Springs Integrated Care Model.	Jaunty Springs Integrated Care Model<br>Through IT integration and a formal Partnership Agreement between the Community Pharmacy and the General Practice, the Community Pharmacist was able to undertake appropriate consultations within the Community Pharmacy setting. Read/write access to the full GP Clinical Record enabled real time information sharing and allowed the community pharmacist to make full use of their training and knowledge as the NHS medicines expert.<br>Patients were proactively directed towards the pharmacy as a source of care, reducing demand on the practice, and improvements in the patient experience as they were able to be seen more quickly as the community pharmacist was more readily available.<br>The vast majority (67%) of pharmacist time was spent providing face to face direct consultation with patients, with the remaining 33% of time spent reviewing and updating patient care plans and pathways through the GP clinical system. The pharmacy team was also working to enhance the ongoing safety and efficiency of Community Pharmacy services. Our evidence suggests that the activity undertaken in the Community Pharmacy setting reduces pressure and redirects demand away from the GP surgery.	Community pharmacy support for General Practice through this integrated approach can:<br>• Create more capacity in primary care through a highly scalable model (9.45%).<br>• Reduce average waiting times to be seen in a primary care setting, reducing<br>pressure on the whole GP team. Average waiting times at the practice were<br>significantly lower than either the local or national average.<br>• Release GP time by utilising the expertise of the community pharmacist to deal<br>with medicines related issues, e.g. drug availability problems.<br>• Support patients in taking their medicines - enhancing adherence.<br>• Support quality and safety of prescribing within the practice.<br>• Enhance patient outcomes, continuity and seamlessness of care between GP<br>Practice and Community Pharmacy - leading to better coordinated management<br>of Long Term Conditions.<br>• Eliminate prescribing errors at source, and in real time by allowing the Community<br>Pharmacist to access the full GP record.<br>• Increase support for frail elderly patients in their own home, and those residing in<br>nursing homes on the Registered List.<br>• Support the best management and use of NHS resources through appropriate	
101	NHS Confederation.	2021	Working collaboratively in an ICS: Freeing up opportunities in community pharmacy.	N/A	UK.	Report.	Report.	N/A	Collaborative working between community pharmacy, primary care networks and federations within integrated care systems.	N/A	This report explores the opportunities for greater collaborative working between community pharmacy, primary care networks and federations within integrated care systems, and how barriers can be overcome.			

102	Parr et al.	2000	Shared learning – a collaborative education and training initiative for community pharmacists and general medical practitioners.	The Pharmaceutical Journal.	UK.	Quantitative evaluation.	Primary research.	N/A	Shared learning, collaborative education and training initiative for community pharmacists and general medical practitioners.	N/A	To plan, implement and evaluate a shared learning, collaborative education and training initiative for community pharmacists and general medical practitioners.	The World Health Organisation (WHO) has defined multiprofessional education as "the process by which a group of students (or workers) from health-related occupations with different educational backgrounds learn together during certain periods of their education, with interaction as an important goal, to collaborate in providing promotive, preventive, curative, rehabilitative and other health- related services".The Scottish Centre for Post Qualification Pharmaceutical Education (SCPPE) has a remit to provide education and training for hospital and community pharmacists in the NHS in Scotland. In early 1998, Greater Glasgow health board (GGHB) was approached with the proposal that the SCPPE be commissioned to undertake shared learning between local community pharmacists and general medical practitioners. A series of three direct learning courses, each of 2.5 hours' duration, was arranged for February and March, 1998, and invitations sent to community pharmacists and GPs in the GGHB area. The overall aim of the series of courses was to provide an opportunity for local community pharmacists and GPs to develop closer professional links and to gain better understanding of their respective roles in the interest of patient care. The topics for the three events were carefully chosen to be as inclusive as possible, but without raising any contentious issues over professional boundaries.<br>The three topics were closely aligned to the PIANA expression of strategic intent and included:The costs of non-compliance in hypertension; The management of minor ailments; Repeat prescribing and medication review. Each of the three courses consisted of a keynote speaker to introduce the topic (40 minutes) and small group work in a problem-based learning format to discuss patient case studies, protocols and systematic approaches to pharmaceutical services in the primary care environment		
103	Eickhoff et al.	2022	Patients' perception on generating medication plans in an interprofessional medication management program: a mixed-methods study.	Journal of Interprofessional Care.	Germany.	Mixed-method study.	Primary research.	N/A	Patients' perception on generating medication plans in an interprofessional medication management program	Inclusion criteria were: ≥18 years, living at home, ≥5 medica- tions, agreed to be cared for by one GP and CP, insured with the health insurance fund AOK PLUS, and written informed consent. Patients with at least one follow-up intervention were eligible. To include a convenience sample of at least 100 patients in the survey, 12 GP/CP teams invited all patients newly signing up for the MMP until the target number was reached. For the interviews, four CPs recruited 10 additional patients.	To assess patients' initial acceptance of the service, how they use the printed MP, and whether they perceived a benefit from it.	Since October 2016, all patients in Germany with at least three prescribed drugs are eligible to receive a paper-based, standardized medication plan (MP) to ensure this overview on their medication. This MP is issued on patients' requests by the prescribing physician, most often the general practitioner (GP), and added to by other physicians and community phar- macists (CPs). To ensure that these MPs are complete, checked for DRPs, and are regularly updated, an interprofessional medication management program (MMP) was implemented involving both the patients' GP and CP.		
104	Piquer-Martinez et al.	2022	Integration of community pharmacy in primary health care: The challenge.	Research in Social and Administrative Pharmacy.	N/A	Narrative Review.	Secondary research.	N/A	Integration of community pharmacy in primary health care.	N/A	A narrative review of the literature identified five articles describing integration involving community pharmacies.	N/A		
105	Pojskic et al.	2009	Ontario family physician readiness to collaborate with community pharmacists on drug therapy management: Lessons for pharmacists.	Research in Social and Administrative Pharmacy.	Canada.	Quantitative study.	Primary research.	N/A	Ontario family physician readiness to collaborate with community pharmacists on drug therapy management.	N/A	To assess Ontario family physicians' attitudes and readiness to collaborate with community pharmacists on drug therapy management.	N/A		
106	Pojskic et al.	2011	Ontario family physician readiness to collaborate with community pharmacists on drug therapy management.	Research in Social and Administrative Pharmacy.	Canada.	Quantitative study.	Primary research.	N/A	Ontario family physician readiness to collaborate with community pharmacists on drug therapy management.	N/A	To ascertain Ontario family physician readiness to collaborate with community pharmacists on drug therapy management.	The conceptual framework for this study of pharmacist-physician collaboration was the trans- theoretical model of behavior change. This model describes behavior change as a series of stages: precontemplation, when individuals are not considering changing their behavior; contem- plation, when they are thinking about it; prepara- tion, when they are taking concrete steps toward it; and action, when they have changed their behavior. The model also proposes that individ- uals continuously weigh the advantages (pros) of changing their behavior against the disadvantages (cons), and that the relative perceived importance of the pros increases and that of the cons de- creases as individuals progress through the stages of change. Another predictor of change included in the model is self-efficacy, or the individual's situation-specific confidence. Finally, the model proposes a number of processes (mental or physi- cal) used to progress through the stages (eg, substituting desirable behaviors for undesirable ones). Collaboration was oper- ationalized as 3 distinct behaviors on a continuum: taking pharmacists' phone calls (low), seeking pharmacists' recommendations regarding patients' drug therapy (mid), and referring patients to phar- macists for medication reviews (high).	Female gender was a significant predictor of physician readiness to seek pharmacists' recom- mendations, with females more likely than males to be in the action stage than the precontempla- tion stage. This may relate to gender differences in leadership style, as previous research has illus- trated that females typically lead in a more democratic or participatory style, whereas males are more likely to lead in a more autocratic or directive style.<br>Number of years in practice was a significant predictor of physician readiness to refer patients to pharmacists for medication reviews, with more experienced physicians more likely to be in the preparation stage than the action stage.	
107	Puspitasari et al.	2015	Challenges in the management of chronic noncommunicable diseases by Indonesian community pharmacists.	Pharmacy Practice.	Indonesia.	Qualitative study.	Primary research.	Chronic noncommunicabl e diseases.	Factors influencing Indonesian primary care pharmacists' practice in chronic noncommunicable disease management.	N/A	Explored factors influencing Indonesian primary care pharmacists' practice in chronic noncommunicable disease management and proposed a model illustrating relationships among factors.	N/A	In Indonesia, the core curriculum for a 4-year undergraduate program predominantly consists of basic and pharmaceutical sciences, with lack of social, behavioural and clinical sciences. The lack of clinically oriented learning and practice represents a key barrier to providing patient-centered practice. Some authors have argued that barriers were derived from a lack of skilled clinical pharmacists to develop an effective pharmacy curriculum and supervise clinical placements where students may practice clinical aspects of pharmacy as well as poor pharmacy practice settings.	
108	Qazi et al.	2021	Perspectives of pharmacists about collaborative asthma care model in primary care.	Research in Social and Administrative Pharmacy.	Australia.	Qualitative study.	Primary research.	Asthma.	Elicit pharmacists' views and recommendations about pragmatic models of collaboration between GPPs and general practitioners.	Asthma patients.	The aim of this study was to elicit pharmacists' views and recommendations about pragmatic models of collaboration between GPPs and general practitioners in providing asthma management services in future service delivery models.	N/A		
109	Rakvaag et al.	2020	Complementing or conflicting? How pharmacists and physicians position the community pharmacist.	Pharmacy Practice.	Norway.	Qualitative study.	Primary research.	N/A	How pharmacists and physicians position the community pharmacist.	N/A	1) To identify how community pharmacists position themselves, and how they are positioned by general practitioners. 2) To assess how well these positions correspond, how the positions align with a proactive position for the pharmacists, and discuss how the positions could potentially impact collaboration.	Positioning theory is focused on how people position themselves and each other in storylines through their speech acts. Positioning can be either reflexive, meaning the positioning of oneself, or interactive, meaning the positioning of each other. The three concepts "speech act", "position" and "storyline" are central in positioning theory. A speech act is the act of making an utterance. A position comprises a cluster of personal attributes, rights, duties and obligations that limits the possible social acts that are available to a person or group as so positioned. A position is negotiable and the result of a dynamic relation between the participants in a social episode. The participants in a social episode co-construct a storyline where each participant claim for themselves or is given by others, a position. A storyline can be defined as the conversational history according to which a social episode evolves and positions arise.<br>Positioning does not only happen at an individual level, but also at group level, where a person's history includes his or her story both as an isolated individual and as a member of various groups. Positioning theory can therefore be used as a tool to better understand social phenomena such as intergroup relations. The stories we tell about ourselves and "the others" may show where there are divergences that may impact collaboration. How we position ourselves and each other can shed light upon the relationship.		
110	Rathbone et al.	2016	Qualitative study to conceptualise a model of interprofessional collaboration between pharmacists and general practitioners to support patients' adherence to medication.	BMJ Open.	Australia.	Qualitative study.	Primary research.	N/A	Interprofessional collaboration within the context of identifying and improving medication non-adherence in primary care.	N/A	This study aimed to propose a model of interprofessional collaboration within the context of identifying and improving medication non-adherence in primary care.			
111	Renfro et al.	2018	Development of a Communication Strategy to Increase Interprofessional Collaboration in the Outpatient Setting.	Pharmacy.	United States.	Mixed-method evaluation.	Primary research.	Diabetes, hypertension.	Development of a Communication Strategy to Increase Interprofessional Collaboration in the Outpatient Setting.	Patients with diabetes (type 1 and 2) and/or patients with hypertension.	To design and implement a communication strategy for patients with diabetes and hypertension between a community pharmacy and physician practice.	An interprofessional team for the practice settings was formed to develop a strategy for collaboration. After agreeing on the common goals and target patient population for the disease states, the team devised a way to communicate via electronic health record (EHR). The communication strategy allowed for more frequent follow-up with the patients which has the potential to result in better clinical outcomes. A communication strategy between a community pharmacy and a physician practice office can be achieved using EHR technology		
112	Rieck & Pettigrew.	2013	How physician and community pharmacist perceptions of the community pharmacist role in Australian primary care influence the quality of collaborative chronic disease management.	Quality in Primary Care.	Australia.	Qualitative study.	Primary research.	Chronic diseases.	How physician and community pharmacist perceptions of the community pharmacist role in Australian primary care influence the quality of collaborative chronic disease management.	N/A	To explore physician and CP perceptions of the CP's role in Australian primary care and how these perceptions may influence the quality of physician/CP CDM programmes.	The definition, clarification and negotiation of roles are aspects of role theory. When roles are created, they give social cues and set a framework whereby the behaviours of individuals performing the roles might be predicted.However, the labelling of roles can also lead to stereotypical assumptions being made about individuals and their roles which may not be accurate.<br>Role theory has been discussed in health care research relating to the roles of physicians and nurses. However, only scant investigation of CPs regarding their role in primary care has been undertaken. This study suggested that the issues relating to role are a very important aspect of physician/CP CDM collaboration		
113	Rieck.	2014	Exploring the nature of power distance on general practitioner and community pharmacist relations in a chronic disease management context.	Journal of Interprofessional Care.	Australia.	Qualitative study.	Primary research.	Chronic diseases.	Power distance on general practitioner and community pharmacist relations in a chronic disease management context.	N/A	This study explored perceptions and attitudes of community pharmacists and GPs regarding the GP/community pharmacist relationship and its impact on GP/community pharmacist collab- orative CDM in primary healthcare. The objective of this study was to ascertain how these aspects influence collaborative CDM between these healthcare professions and if these findings could be illuminated by a theory or concept.	In light of the power disparity apparent in the GP/community pharmacist relationship, the organisational and relationship theory that best described this relationship dynamic was power distance. Power distance is defined as the extent to which less powerful members of an organisation understand and expect the unequal distribution of power (Hofstede, 1994). The difference in status of GPs and community pharmacists has been acknowledged in literature discussing community pharmacist reprofessionalisation (Chen & de Almeida Neto, 2007; Hughes & Mccann, 2003; Rieck & Pettigrew, 2013). The concept of power distance does not appear to have been explicitly discussed in relation to the GP/ community pharmacist relationship and CDM issues. Hofstede (1980) raised the concept of power distance in his work on the dimensions of national and organisational cultures (Hofstede & Hofstede, 2005). Power distance reflects the difference in power between a superior and subordinate (Hofstede, 1980; Hofstede & Hofstede, 2005; Mulder, Veen, Rodenberg, Frenken, & Tielens, 1973; Wood, Zeffnae, Fromholtz, & Fitzgerald, 2006). When power distance is small, the subor- dinate's power is close to that of their superior (Hofstede, 1994). When high power distance exists, superiors are more likely to make decisions alone, without including subordinates in solving problems (Hofstede, 1980; Wood et al., 2006).		
114	Roach.	2018	Supporting GPs through community pharmacies.	National Health Executive.	UK.	N/A	Report.	N/A	Integrated primary care model.	N/A	To evaluate this integrated primary care model, outcome and impact.	Outline of the integrated service delivery model (Jaunty Springs and Baseline Surgery)<br>The community pharmacy accessed the GP practice's SystmOne from the pharmacy consultation room, utilising the existing N3 connection for virtual review booking of patients and updating of the patient record in real time. In-depth medicines data analysis was led by the community pharmacist. Patients were given the choice of healthcare professional in either the practice or undertaking a consultation with the community pharmacist in their consulting room. They had access to the full community pharmacy team, which consisted of two community pharmacists (one of whom was an independent prescriber), two dispensers, and 1.5 WTE accuracy-checking technician and medicines counter assistant. This team, working in tandem with the general practice team, generates a high-quality, seamless integrated care service for the patients. This model also brings the additional benefits of utilising existing patient and professional relationships and breaking down silos within the health system, which combined have led to improved trust and confidence in the clinical input of the community pharmacy team.<br>The community pharmacist undertakes direct consultations with patients in the community pharmacy or in relation to the elderly in their place of residence.		
115	Rubio-Valera et al.	2021	Factors affecting collaboration between general practitioners and community pharmacists: a qualitative study.	BMC Health Services Research.	Spain.	Qualitative study.	Primary research.	N/A	Factors affecting collaboration between general practitioners and community pharmacists	N/A	To identify and analyze factors affecting GP-CP collaboration.	N/A	Commitment to primary care is higher in Catalonia than in Balearic Islands and there is a greater investment of resources in primary care with respect to total health expenditure. Consequently, in comparison with Balearic Islands, there is a higher dens- ity of PCHCs, a lower ratio of patients per GP, a greater number of services integrated within primary care (i.e. dentistry) and better access to diagnostic procedures by GPs in Catalonia. Electronic pre- scribing links the GP's prescriptions with the community pharmacists and acts as a communication channel be- tween the two professionals. The implementation of electronic prescribing in the Balearic Islands began in 2006, long before its introduction in Catalonia.	
116	Woolley & Cantrill.	2000	Professional perspectives on a feasibility stcdy of GP-pharmacist collaboration in the management of angina.	International Journal of Pharmacy Practice.	UK.	Qualitative study.	Primary research.	Angina.	GP and CP perspectives on a feasibility stcdy of GP-pharmacist collaboration in the management of angina.	Patients aged 45-75 years with stable angina (grades I - I11 using the Canadian Cardiovascu- lar Society [CCS] classification criteria)26and re- ceiving four or fewer prescribed medications for their IHD, were eligible for the study. A purpo- sive sample of patients who satisfied the inclu- sion criteria was selected by the participating GPs and pharmacists. A total of 327 patients who satisfied the study inclusion criteria were invited to attend the CPRACs. Two hundred and thirty-six (72 per cent) attended and 208 (88 per cent) remained in the study until completion.	To report the views and experiences of a convenience sample of community pharmacists and general practitioners (GPs)who participated in a five-month feasibility study exploring the ways in which community pharmacists could contribute to the management of patients with stable angina when working in partnership with GPs.	The "stages of change" model, which has been widely integrated into health promotion ac- tivities, was employed to support delivery of the lifestyle interventions. In brief, this model pro- poses that, in relation to readiness to change be- haviour, people pass through seven distinct stages, from lack of awareness through to main- taining changed behaviour. Use of this model provided the pharmacists with a framework for breaking down the complex process of be- havioural change into manageable "pieces". Once each patient had accepted the "stages of change" model, a tailored approach to the mod- ification of their risk factors was established. Progress towards the desired changes then pro- ceeded according to each patient's individual needs and feelings and, because of the number of stages within the framework, monitoring and ap- propriate support of progress was facilitated. Hence, a patient who revealed an awareness of the need, and a firm desire, to quit smoking was given considerable verbal and written material and was encouraged to attend a local smoking cessation group, whereas a patient who had re- cently quit smoking was commended for achiev- ing success and was encouraged to maintain this health promoting behaviour. Individualised care plans were established by the pharmacists in collaboration with the pa- tients at the end of the first reviews. Interven- tions, including investigations (usually full lipid profile), and GP referrals were made as neces- sary. Patients were sometimes referred to their GP for medication review, with recommenda- tions made by the pharmacists relating to one or more therapeutic groups prescribed for the man- agement of angina. In most cases, suggested changes in prescribed medications, within the confines of the study protocol, were agreed by the GPs at the end of the patient reviews, result- ing in the issue of new prescriptions. Patients were also referred to local smoking cessation and slimming groups where appropriate, to reinforce advice		
117	Saha et al.	2020	A Nationwide Survey of Australian General Practitioners on Antimicrobial Stewardship: Awareness, Uptake, Collaboration with Pharmacists and Improvement Strategies.	Antibiotics.	Australia.	Mixed-method study.	Primary research.	Antimicrobial stewardship.	General practitioners' (GPs') awareness of AMS, uptake of AMS strategies, attitudes towards GP–pharmacist collaboration in AMS and future AMS improvement strategies.	N/A	This study aims to assess general practitioners' (GPs') awareness of AMS, uptake of AMS strategies, attitudes towards GP–pharmacist collaboration in AMS and future AMS improvement strategies.	N/A		
118	Saha et al.	2021	Divergent and Convergent Attitudes and Views of General Practitioners and Community Pharmacists to Collaboratively Implement Antimicrobial Stewardship Programs in Australia: A Nationwide Study.	Antibiotics.	Australia.	Mixed-method study.	Primary research.	Antimicrobial stewardship.	Attitudes and Views of General Practitioners and Community Pharmacists to Collaboratively Implement Antimicrobial Stewardship Programs in Australia.	N/A	This study aimed to investigate the convergent and divergent attitudes and views of general practitioners (GPs) and community pharmacists (CPs) about AMS implementation and their perceived challenges of collaboration to design a GP–pharmacist collaborative AMS (GPPAS) model	N/A	In the UK, a national consensus-led competency framework has been used to provide a basic set of AMS competencies among undergraduate healthcare professionals including GPs and CP.	
119	Salim & Elgizolil.	2016	Exploring self perception of community pharmacists of their professional identity, capabilities, and role expansion.	Journal of Research in Pharmacy Practice.	Sudan.	Qualitative study.	Primary research.	N/A	Self-perception of community pharmacists of their professional identity, capabilities, and role expansion.	N/A	To define the identities and roles of community pharmacists from their own perspectives and to examine how they think patients and doctors perceive them and how they assess their capabilities. The study also aimed at exploring pharmacists' opinions regarding role expansion.	N/A		
120	Saragi et al.	2019	Exploring The Potential of Interprofessional Collaboration in Medication Therapy Management in Primary Health Care.	FABAD Journal of Pharmaceutical Sciences.	Indonesia.	Qualitative study.	Primary research.	Type 2 Diabetes.	Interprofessional collaboration between doctor and pharmacsts in MTM at type 2 diabetic patient	Patients with type 2 diabetes.	This study aims to explore the potential of interprofessional collaboration between doctor and pharmacsts in MTM at type 2 diabetic patient	N/A		
121	Saramunee et al.	2014	How to enhance public health service utilization in community pharmacy?: General public and health providers' perspectives.	Research in Social and Administrative Pharmacy.	UK.	Qualitative study.	Primary research.	N/A	General public and health providers' perspectives on pharmacy-based public health services, and identify potential factors affecting service use.	Forty-one members of the public (a 4% re- sponse to the mailing) indicated interest in joining a FG, of whom 13, recruited by mailing, actually took part.	To explore experiences and views of 4 groups of participants, the general public, PHs, general practitioners (GPs), and other stakeholders (STs) on pharmacy-based public health services, and identify potential factors affecting service use.	N/A	In this paper, PH refers to community pharmacists.	

122	Sokhi et al.	2020	Learning from community pharmacists' initial experiences of a workplace-based training program.	Currents in Pharmacy Teaching and Learning.	UK.	Qualitative study.	Primary research.	N/A	Learning from community pharmacists students' initial experiences of a T workplace-based training program	N/A	To identify course components that support development, describe the effect on practice, and explore the perceived advantages and disadvantages of this model of postgraduate education.	The program incorporated personal development planning against a personal de- velopment framework, workplace mentoring, employment of work-based assessment tools, ac- tivities that encouraged increased inter-professional working, reflection, and opportunities for peer support. Community pharmacists undertaking the course (students) maintained a learning portfolio and measured progress and identified development needs using the General Level Framework (GLF).Students were assigned an experienced community or primary care-based pharmacist as a tutor to support them in selecting appropriate workplace learning activities and to conduct workplace and work-based assessments. Support from a local general practitioner (GP) practice was desirable to facilitate learning opportunities such as access to patient clinical records. To facilitate access, the university provided a letter explaining the course requirements that students could use to introduce themselves.		
123	Tarn et al.	2012	Older patient, physician and pharmacist perspectives about community pharmacists' roles.	International Journal of Pharmacy Practice.	United States.	Qualitative study.	Primary research.	N/A	Older patient, physician and pharmacist perspectives about community pharmacists' roles.	Patients aged 63 years and older.	To investigate (1) perspectives about the pharmacist's role in pharmacist–patient interac- tions and (2) barriers to pharmacist–patient and pharmacist–physician interactions that may affect patient care	N/A		
124	Thomas & Cashin-Cox.	2016	Evaluation of Participant Experience of the Lewisham Clinical Commissioning Group 'Walk In My Shoes' Inter-Professional Exchange Project.	Pharmacoepidemiology and Drug Safety.	UK.	Qualitative study.	Primary research.	N/A	GP and CP Experience of the Lewisham Clinical Commissioning Group 'Walk In My Shoes' Inter-Professional Exchange Project	N/A	This evaluation aimed to analyse individual personal re- flection forms (PRFs) and service action planning forms (APF) to identify best practice to support joint working around medicines management.		It's a supplement article from Prescribing and Research in Medicines Management (UK & Ireland) Conference 2016 Health Foundation London January 29th 2016 "Ethics, Economics and the Future of Medicines – A Population Perspective".	
125	Toh et al.	2017	An analysis of inter-professional collaboration in osteoporosis screening at a primary care level using the D'Amour model.	Research in Social and Administrative Pharmacy.	Malaysia.	Qualitative study.	Primary research.	Osteoprosis.	Analysis of inter-professional collaboration in osteoporosis screening at a primary care level in Malaysia.	Purposive sampling was used to select non-osteoporotic post- menopausal women !50 years of age from three main ethnics groups (Malays, Chinese, and Indians) in Malaysia.	This study describes the perspective of patients, nurses, pharmacists, doctors and policy makers to identify the level of collaboration and the areas for improvement to achieve inter-professional collaboration between doctors, nurses, pharmacists and policy makers in a primary care clinic.	D'Amour model: There are ten indicators within the four interlinked domains of shared goals and vision, internalization, formalization and gover- nance. The 10 indicators can be categorized to three levels repre- senting the level of achievement of an indicator. Level three is called "active collaboration" which is the maximum level of achievement. It refers to a setting that has successfully established stable collaboration and is sustainable despite uncertainties in the health care system. Level two is called "developing collaboration". It is collaboration that is not stable and may still be subjected to re- evaluation as a consensus has not been reached. Although progress on collaboration is being made, it would require more time to achieve an active collaboration. The minimum level of achievement is level one, which is called "potential/latent collaboration". This level refers to collaboration that does not yet exist or has been blocked by conflicts that are so serious that the system cannot move forward.	In Malaysia, primary care services comprise of private general practices, government primary care clinics in the community, and government primary care clinics located within teaching hospitals. All three practices manage both acute and chronic conditions. This study was conducted in a government primary care clinic located within a teaching hospital. Malaysia has not achieved dispensing separation. This means that general practitioners can dispense medications from their clinic, but patients would have to pay the full cost of their medications. However, in government clinics where the cost of healthcare and medications are heavily subsi- dized, medications are dispensed by pharmacists.	
126	Twigg et al.	2013	The current and future roles of community pharmacists: Views and experiences of patients with type 2 diabetes.	Research in Social and Administrative Pharmacy.	UK.	Qualitative study.	Primary research.	Type 2 Diabetes.	Experiences and views of patients with type 2 diabetes regarding the current and proposed future role of community pharmacists.	Forty-four patients were consented from whom 25 participants were recruited and attended one of the four focus groups. The remaining participants were not selected either as the demographic in- formation made them less suitable, or they were unable to attend one of the focus groups. The reasons for selecting he four demographic characteristics on which to base sampling are detailed below:<br>Gender<br>There is evidence to suggest that men and women cope differently with type 2 diabetes and that it can impact on their ability to handle the condition.<br>Age<br>As age increases, a patient is more likely to be prescribed more medicines and therefore may have a more in-depth experience of visiting the pharmacy and interacting with pharmacists.<br>Number of medicines prescribed<br>If patients are prescribed more medicines they may be visiting the pharmacy more often and interacting with the pharmacist.	To explore the experiences and views of patients with type 2 diabetes regarding the current and proposed future role of community pharmacists.			
127	Van et al.	2007	General Practitioner Perceptions of Extended Pharmacy Services and Modes of Collaboration with Pharmacists.	Journal of Pharmacy Practice and Research Volume.	Australia.	Qualitative study.	Primary research.	N/A	GP perceptions of extended pharmacy services and modes of collaboration with pharmacists.	N/A	To explore the perceptions of Australian general practitioners (GPs) towards extended pharmacy services, and to investigate the modes and extent of collaboration between community pharmacists and GPs	This study used the CWR model proposed by McDonough and Doucette. It has been suggested that the pharmacist-physician collaborative working relationship progresses through a series of stages from brief interactions on routine matters (Stage 0) to a mutually beneficial relationship in which both parties have clearly defined roles and responsibilities (Stage 4)		
128	Van et al.	2012	Community pharmacist attitudes towards collaboration with general practitioners: development and validation of a measure and a model.	BMC Health Services Research.	Australia.	Quantitative evaluation.	Primary research.	N/A		N/A	This study aims to develop and validate 1) an instrument to measure pharmacist attitudes towards collaboration with GPs and 2) a model that illustrates how pharmacist attitudes (and other variables) influence collaborative behaviour with GPs.	Development of the attitudes towards collaboration instrument for pharmacists<br>Generation of the items of the Attitudes Towards Col- laboration Instrument for Pharmacists (ATCI-P) was based on the hypothesised model which was guided by existing literature on interprofessional collab- oration, and pharmacist and GP interviews. The questionnaire consisted of the refined ATCI-P, a previously validated behavioural measure "Frequency of Interprofessional Collaboration Instrument for Pharma- cists" (FICI-P) and demographic questions. The FICI-P is a ten-item unidimensional measure that cap- tures collaborative behaviour between pharmacists and GPs in primary care.<br>Frequency of Interprofessional Collaboration Instrument for Pharmacists (FICI-P)<br>1. The GP and I openly communicated with each other. 2. I informed the GP of new products/services that are available/that I provide. 3. The GP contacted me for specific drug information.<br>4. The GP contacted me for specific patient information.<br>5. I contacted the GP to clarify scripts.<br>6. I contacted the GP to discuss dosage adjustments.<br>7. I contacted the GP to recommend an alternative medication (e.g. due to an adverse reaction, contraindication etc).<br>8. The GP adjusted patient medication after my recommendation.<br>9. The GP shared patient information with me.<br>10. The GP involved me in decisions regarding	The drive for collaboration in the provision of healthcare has also led to the development of a number of models that conceptualise collaborative behaviour. The "Collaborative working relationship model", proposed by McDonough and Doucette, is a general model for physicians and pharmacists. The model describes collaboration as an evolving process consisting of five stages: stage 0 – professional awareness, stage 1 – professional recogni- tion, stage 2 – exploration and trial, stage 3 – profes- sional relationship expansion and stage 4 – commitment to the collaborative working relationship. The model also posits that the drivers of collaboration include partici- pant, context and exchange characteristics. More recently, Bradley et al. developed a "Conceptual model of GP-pharmacist collaboration" specifically for GPs and community pharmacists, consisting of three stages: stage 1 – isolation, stage 2 – communication and stage 3 – col- laboration, with key components of collaboration identi- fied as locality, service provision, trust, 'knowing' each other, communication, professional roles and respect.	
129	Van et al.	2013	Development and validation of a measure and a model of general practitioner attitudes toward collaboration with pharmacists.	Research in Social and Administrative Pharmacy.	Australia.	Quantitative evaluation.	Primary research.	N/A	General practitioner attitudes toward collaboration with pharmacists.	N/A	To develop and validate, in the context of primary care in Australia 1) an instrument to measure GP attitudes toward collaborating with pharmacists and 2) a model that illustrates how GP attitudes (and other variables) influence GP-pharmacist collaborative behavior.	scale the extent to which they agreed or disagreed with a statement concerning themselves and the pharmacist with whom they had most dealings. The points on the response scale were: 1 1⁄4 'strongly disagree', 2 1⁄4 'disagree', 3 1⁄4 'neither agree nor disagree', 4 1⁄4 'agree' and 5 1⁄4 'strongly agree.'Frequency of Interprofessional Collaboration Instrument for GPs (FICI-GP)<br>1. The pharmacist and I openly communicated with each other.<br>2. The pharmacist informed me of new prod- ucts/services that are available/that they provide.<br>3. I contacted the pharmacist for specific drug information.<br>4. I contacted the pharmacist for specific pa- tient information.<br>5. The pharmacist contacted me to clarify scripts.<br>6. The pharmacist contacted me to discuss dos- age adjustments.<br>7. The pharmacist contacted me to recommend an alternative medication (e.g., due to an ad- verse reaction, contraindication etc).<br>8. I adjusted patient medication after the phar- macist's recommendation.<br>9. I shared patient information with the pharmacist.<br>10. I involved the pharmacist in decisions re- garding medication management.<br>Attitudes Toward Collaboration Instrument for GPs (ATCI-GP)<br>1. The professional communication between myself and the pharmacist is open and honest.<br>2. The pharmacist is open to working together with me on patients' medication management.<br>3. The pharmacist delivers high quality health-care to patients.<br>4. The pharmacist has time to discuss with me matters relating to patients' medication regimens 5. The pharmacist meets		
130	Driel et al.	2017	Learnings and challenges to deploy an interprofessional and independent medical education programme to a new audience.	Journal of European CME	Australia.	Qualitative evaluation.	Primary research.	Medication-overuse headache.	An Australian IPE activity on medication-overuse head- ache (MOH) with general practitioners (GPs) and community pharmacists as learners.	N/A	This study reports on the outcomes of an Australian IPE activity on medication-overuse head- ache (MOH) with general practitioners (GPs) and community pharmacists as learners.	The design of the activity, which followed the predisposing–enabling–reinforcing instructional framework by Green and Kreuter, aimed to: (1) improve knowledge and foster a willingness in GPs and pharmacists to work collaboratively to enhance the prevention, diagnosis and management of MOH; and (2) address their educational gap by demonstrating the utility of a blended learning IPE strategy on MOH. Integrated into the activity was an assessment of its effectiveness and impact to instil change in the participants' knowledge of MOH, attitude and willingness to treat, and clinical practice behaviours of GPs and pharmacists to work together. As such, it consists of: (a) a predisposing activity that provides an opportunity for participants to reflect on their current clinical practice; (b) a structured learning activity of at least 6 hours, covering both a person approach to enhance profes- sional competence (behaviour, attitude, skills and knowledge) and a system approach that focuses on team and procedural processes to safeguard patient safety; and (c) a reinforcing activity that consolidates learning. The MOTIVATE programme's structure and con- tent were as follows:<br>● Predisposing activity/pre-work – a short question- naire (available online or paper-based), which required participants to recall a patient/client who presented with frequent headaches or often sought pain medication for headaches in their practice or pharmacy, and describe how headache histories were obtained, how these patients were diagnosed, treated, managed and whether they were referred for speciality care. This aimed to help the partici- pants "recognise a teachable moment" [16, p. 5], and an interest to pursue the subsequent modules.<br>● Module 1: Fundamental concepts of MOH – a 2- hour online course, with case-stemmed, multiple- choice and short-answer questions, short videos of	Medication-overuse headache (MOH) and other recur- rent headaches are a public health concern because of the disability and ill-health they cause in 1–2% of the popula- tion. Community pharmacists can also play a crucial role in the management of chronic headaches, particularly due to their knowledge of prescription and over-the-counter (OTC) medications and their easy access to patients. However, effective outcomes are only possible with "ade- quate and continuous training on both the management of therapies and the relationship with the physicians and/ or patients"	
131	Weissenborn et al.	2017	Interprofessional communication between community pharmacists and general practitioners: a qualitative study.	International Journal of Clinical Pharmacy.	Germany.	Qualitative study.	Primary research.	N/A	Interprofessional communication between community pharmacists and general practitioners.	N/A	To assess both professions' perceptions of interprofessional communication with regard to content and methods of communication as a basis to subsequently develop best-practice recommendations for information exchange.			
132	Wright & Twigg.	2016	Community pharmacy: an untapped patient data resource.	Integrated Pharmacy Research and Practice.	UK.	Review article.	Review.	N/A	How the information that is currently available in community pharmacies can be used to enhance service delivery and patient care.	N/A	This review describes how the information that is currently available in community pharmacies can be used to enhance service delivery and patient care.			
133	Hermansyah et al.	2020	Primary health care policy and vision for community pharmacy and pharmacists in Indonesia.	Pharmacy Practice.	Indonesia.	Review article.	Review.	N/A	Primary health care policy and vision for community pharmacy and pharmacists in Indonesia.	N/A	To review the primary health care policy and vision for community pharmacy and pharmacists in Indonesia.		Community pharmacists in Indonesia contribute to health care through the delivery of medicines and health-related products and services either independently or in collaboration with other primary care members, particularly General Practitioners (GPs). Dispensing has been the predominant activities in community pharmacy. However, the sale of over the counter medications and practice consultations are also common with pharmacist plays a role in providing advice and assistance for self- medication. These features might imply that community pharmacies are strategically placed and can offer a unique role to relieve strains on the health care system and reduce the over-reliance on the major primary care providers, e.g. GPs and the hospital admissions by operating at their full scope of practice.	
134	Dew et al.	2018	Attitudes and perceptions of health professionals towards management of hypothyroidism in general practice: a qualitative interview study.	BMJ Open.	UK.	Qualitative study.	Primary research.	Hyperthyroidism.	Attitudes and perceptions of health professionals towards management of hypothyroidism in general practice	N/A	To explore the attitudes and perceptions of health professionals towards management of hypothyroidism that contributes to the suboptimal treatment of hypothyroidism in general practice.		There is a significant health professional behavioural component to the management of hypothyroidism. Addressing the differences in patient and professional knowledge and perceptions could reduce the barriers to optimal treatment, while continuity of care and increased involvement of pharmacists and practice nurses would help to promote optimal thyroid replacement.	
135	Deslandes & Frazer.	2011	An exploration of the views of general practitioners on the role of the community pharmacist.	International Journal of Pharmacy Practice.	UK.	Qualitative study.	Primary research.	N/A	Opinions of general practitioners (GPs) regarding the services provided by community pharmacists.	N/A	To explore the opinions of general practitioners (GPs) regarding the services provided by community pharmacists.		This was an abstract from Oral Session 3 – Enhancing the Role of the Community Pharmacist – Chairs: James Desborough and Amanda Wellings, Patient Representativ	
136	Cheong et al.	2013	Multidisciplinary collaboration in primary care: through the eyes of patients.	Australian Journal of Primary Health.	Australia.	Qualitative study.	Primary research.	Asthma.	Asthma patient perception of multidisciplinary care.	A total of 16 asthma patients (10 female) participated in the study, with data saturation achieved following the 12th participant. Patients were aged between 19 and 74 years (mean, 39 years). Ten patients had lived with asthma since childhood while six patients were first diagnosed with asthma as an adult more than 5 years ago. Although a majority of patients reported their asthma to be well controlled, many reported ongoing asthma symptoms and past experiences of asthma 'flare ups' or asthma attacks. Seventy- five percent (12 patients) reported using combination therapy (preventer and reliever medication) on a regular basis. Nineteen percent (three patients) only used reliever medication when required for symptom relief.	To gain an understanding of the experiences, perceptions, attitudes and potential role of people with chronic illness (asthma) on the delivery of MDC in the Australian primary health care setting.			

Paper N	Rationale for paper/review	What problems is the initiative/intervention designed to address?	What solutions/recommendations are proposed?	From which stakeholders' perspective? (Community pharmacy, general practice, patients)	Descriptive information about the General Practice/Community Pharmacy	Definitions of collaboration and integration	Collaboration and Integration information	Patient perspective	Summary of findings	Strengths, limitations, and future research directions
1	AR is both a common and high burden disease, with the majority of AR sufferers purchasing suboptimal/inappropriate AR medication from community pharmacies. Unfortunately, it is still a challenge to translate the AR management guidelines that are available at both a global and national level into practice. This study aimed to explore the experiences and perceptions of community pharmacists with regards to the implementation of AR management guidelines in real-life everyday practice.	The intervention is designed to optimise AR management within the community pharmacy setting.	Optimal AR management requires a multi-pronged approach which goes beyond the pharmacist. It was evident from the research that AR management must be a collaborative effort between pharmacists, GPs, pharmacy assistants, and their patients, and pharmacists must be equipped with comprehensive tools and strategies to communicate with and engage patients about optimizing their AR treatment. Although in some non-Australian environments the GP is central to the management of AR [25], in Australia and countries with similar health care systems where there is an extensive availability of AR treatments in pharmacy and limited access to GP appointments [27], it is essential that pharmacists and GPs work collaboratively to manage AR and support their patients with AR self-management.	Community pharmacy.	N/A	N/A	N/A	N/A	Pharmacists regularly cited the patient as a barrier to their ability to improve AR management. Patient beliefs, expectations, and perceptions with respects to their AR were a common concern expressed by pharmacists. While pharmacists appreciated the burden associated with AR and the importance of controlling the symptoms, they felt that patients did not feel the same way and that AR continues to be viewed as a trivial or nuisance condition by the patient. Patients would frequently rush in and out when visiting the pharmacy to purchase AR medication and appeared to have limited time to speak to the pharmacist. An additional patient factor that pharmacists reported in AR management was the inability of pharmacists to follow up and monitor their patients. While pharmacists told their patients to come back for a follow up consultation, they very rarely saw the same patient again. Pharmacists in larger pharmacies expected that they may not see the same patient again for follow up but were happy to follow up patients on behalf of other pharmacists. Pharmacists from small pharmacies stated that it was helpful to have regular patients, as it made the follow up of their treatment recommendations a lot easier. A significant challenge reported by pharmacists was the absence of collaboration and inconsistency in the messages delivered by doctors working within a shared pharmacy and medical centre premises. Pharmacists found it difficult to challenge the appropriateness of doctors' recommendations when they felt it was inappropriate. For example, they reported that many people with AR came from the doctor with a doctor's note or a prescription for an OAH (antihistamine). Upon review of the patient, pharmacists determined that this was not necessarily an optimal treatment for the patient, however they felt it was not appropriate to challenge the general practitioner's recommendations. The physical layout of the pharmacy also presented a challenge to some pharmacists. Some pharmacies had no comfortable space in between shelves or a private counselling area, and pharmacists felt this limited their ability to deliver effective counselling to their AR patients. Many pharmacists felt that the implementation of AR management guidelines into real-life everyday practice should be collaborative, with the AR-CMaP program broadened to include pharmacy assistants and GPs. The pharmacists stated that if their neighbouring GP colleagues were provided with the same training at the same time, it would ensure a consistent message was delivered to their common patient community and would re-enforce the recommended AR management pathways.	Although this study is limited by its small sample size, it is valuable in its methodology and the unique location chosen for its high AR prevalence. AR pathways are traditionally evaluated by the changes in clinical outcomes that they generate [31]. However, this study employed the principles of evaluation, where the interest was in finding out what worked well, what did not, why, and in what context, in order to further inform the development of the program [31], as well as exploring physical, social, cultural, and resource limitations from the perspective of individuals within the PARIHS implementation framework.
2	Asthma is a chronic airway condition that affects approximately 300 million people worldwide. Community pharmacists can play an important role in improving asthma management, by providing education on appropriate use of asthma medication. There is evidence that community pharmacy based interventions can substantially improve inhalation technique, medication adherence and asthma control. The rationale for the study was to evaluate this new community pharmacy asthma service, and its implementation.	Asthma management.	Future NMS could benefit from formalising the role of the physician in the service.	Community pharmacy.	N/A	N/A	The current evaluation study suggests that physicians and patients were generally not aware of the intervention and that pharmacists lack collaboration with physicians.	N/A	As revealed by the focus groups discussions, some GPs found it inappropriate that pharmacists can initiate an NMS without a physician's approval, and suggested that the NMS should be prescribed and that feedback from the pharmacist should be added to the patient's file (if applicable). As revealed by the interviews, pharmacists considered the remuneration for NMS as an important step in the evolution of pharmaceutical care and as a motivation for pharmacists to perform NMS. In contrast, the focus groups with the GPs indicated that they found it unnecessary or even inappropriate to give a fee to pharmacists to provide this kind of service, in proportion to existing services in general practice. The current evaluation study suggests that physicians and patients were generally not aware of the intervention and that pharmacists lack collaboration with physicians. However, the acceptance of the intervention was also a topic of debate in the focus groups. GPs did not see the added value of the NMS intervention, and some found it inappropriate that pharmacist receive a fee for this kind of services. GPs during the focus groups indicated that they did not perceive the Asthma Control Test (ACT) as an appropriate tool to be used by pharmacists, as this could imply that the latter is performing a clinical act. Some GPs experienced this kind of service as a threat to their expertise. In terms of the immediate organizational context of the NMS intervention, the physical space that is required in the pharmacy for an intervention to takes place remains an important barrier for pharmacists to engage in NMS. An NMS requires a designated space where the pharmacist can guarantee privacy for the patient. Although the presence of a private space in the Belgian pharmacy is mandatory since [illegible]	It must be acknowledged that although this study allowed to partly describe how community pharmacists managed NMS in practice, it does not provide enough information to fully describe the quality of the services that were delivered, or to conclude whether the pharmacist did well in implementing NMS and if the patients benefited from it. Quality improvement can also be done through education and training. Although some training on introducing NMS was provided by the pharmacist's organizations, these occurred only after the initiation of the project in October 2013. In the UK, community pharmacists are obliged to follow a training program and have to achieve national accreditation through a competency-based assessment before they are allowed to provide advanced pharmaceutical services. This was a strong driver for increasing the engagement of pharmacists in NMS in the UK and might be considered in future initiatives.
3	An earlier study in NZ reported concerns from GPs re: pharmacists' clinical roles in performing medication reviews. Currently pharmacists in some parts of NZ are funded to provide medication reviews and a further proposed service is pharmacist prescribing. The present study focused on GPs' perceptions of pharmacists' contributions to medication reviews and prescribing and the potential impact on patients and on GPs' own daily practice.	This paper aims to understand GPs perceptions of pharmacists contributions.	GPs perceived that pharmacist prescribing would include pharmacists making a diagnosis. This is different from the proposed model for pharmacist prescribing that is reliant on a medical practitioner's diagnosis and treatment plan. In general, the current study found GPs to be more accepting of pharmacists providing medication reviews and reluctant to accept pharmacist prescribing unless appropriate controls with close collaboration and co-location of services occurred.	Community pharmacy, general pratice.	Three groups of [illegible] in two localities in [illegible]	[illegible]	[illegible]	[illegible]	[illegible]	[illegible]
4	Australian community pharmacies offer a range of professional pharmacy services (PPS) which include HMR and the Diabetes Medication Assistance Service (DMAS). The extent of interaction and collaboration between general practitioners (GPs) and pharmacists in the context of these services is unknown. Therefore, the aim of this study was to investigate (1) the nature and extent of interactions between GPs and community pharmacists and; (2) the factors that influence these interactions in the context of PPS.	PPS aims to provide a platform for collaboration to enable GPs and pharmacists to work together with the goal of improving [illegible]	To improve collaboration between GPs and pharmacists in the primary care setting it would be necessary to develop strategies targeted not only at the [illegible]	General pratice.	15 GP and 15 pha[illegible] conducted giv[illegible]	[illegible]	[illegible]	[illegible]	[illegible]	[illegible]
5	[illegible]	[illegible]	[illegible]	[illegible]	[illegible]	[illegible]	[illegible]	[illegible]	[illegible]	[illegible]
6	[illegible]	[illegible]	[illegible]	[illegible]	[illegible]	[illegible]	[illegible]	[illegible]	[illegible]	[illegible]
7	[illegible]	[illegible]	[illegible]	[illegible]	[illegible]	[illegible]	[illegible]	[illegible]	[illegible]	[illegible]
8	[illegible]	[illegible]	[illegible]	[illegible]	[illegible]	[illegible]	[illegible]	[illegible]	[illegible]	[illegible]
9	[illegible]	[illegible]	[illegible]	[illegible]	[illegible]	[illegible]	[illegible]	[illegible]	[illegible]	[illegible]
10	[illegible]	[illegible]	[illegible]	[illegible]	[illegible]	[illegible]	[illegible]	[illegible]	[illegible]	[illegible]
11	[illegible]	[illegible]	[illegible]	[illegible]	[illegible]	[illegible]	[illegible]	[illegible]	[illegible]	[illegible]
12	[illegible]	[illegible]	[illegible]	[illegible]	[illegible]	[illegible]	[illegible]	[illegible]	[illegible]	[illegible]
13	[illegible]	[illegible]	[illegible]	[illegible]	[illegible]	[illegible]	[illegible]	[illegible]	[illegible]	[illegible]
14	[illegible]	[illegible]	[illegible]	[illegible]	[illegible]	[illegible]	[illegible]	[illegible]	[illegible]	[illegible]
15	[illegible]	[illegible]	[illegible]	[illegible]	[illegible]	[illegible]	[illegible]	[illegible]	[illegible]	[illegible]
16	[illegible]	[illegible]	[illegible]	[illegible]	[illegible]	[illegible]	[illegible]	[illegible]	[illegible]	[illegible]
17	[illegible]	[illegible]	[illegible]	[illegible]	[illegible]	[illegible]	[illegible]	[illegible]	[illegible]	[illegible]
18	[illegible]	[illegible]	[illegible]	[illegible]	[illegible]	[illegible]	[illegible]	[illegible]	[illegible]	[illegible]
19	[illegible]	[illegible]	[illegible]	[illegible]	[illegible]	[illegible]	[illegible]	[illegible]	[illegible]	[illegible]
20	[illegible]	[illegible]	[illegible]	[illegible]	[illegible]	[illegible]	[illegible]	[illegible]	[illegible]	[illegible]
21	[illegible]	[illegible]	[illegible]	[illegible]	[illegible]	[illegible]	[illegible]	[illegible]	[illegible]	[illegible]
22	[illegible]	[illegible]	[illegible]	[illegible]	[illegible]	[illegible]	[illegible]	[illegible]	[illegible]	[illegible]
23	[illegible]	[illegible]	[illegible]	[illegible]	[illegible]	[illegible]	[illegible]	[illegible]	[illegible]	[illegible]
24	[illegible]	[illegible]	[illegible]	[illegible]	[illegible]	[illegible]	[illegible]	[illegible]	[illegible]	[illegible]

25	Community pharmacists have demonstrated that, in collaboration with family physicians, they can enhance medication adherence and comprehensive medication assessment. Numerous studies have demonstrated pharmacist–physician collaboration in disease management, such as hyper- tension, anti-coagulation and diabetes, in a primary care setting results in improved patient outcomes. There has been no documented collaboration between a pharmacist and family physicians in providing direct patient acute care in a primary care setting. This study describes a de novo collaborative practice between a pharmacist and a family physician in a family medicine clinic.	The primary objective of the study is to describe the collaboration model between a pharmacist and family physician in a family medicine clinic. The secondary objectives are to describe the workload for the pharmacist.	It is possible to establish a family physician–pharmacist collaboration that involves both providing direct patient care independently and in shared consultation.	Community pharmacy, general pratice.	[illegible]	[illegible]	[illegible]	[illegible]	[illegible]	[illegible]
26	Collaboration between primary care providers is a well-established necessity for the community organization: poly-medicated patients' drug regimen can be optimized by he physician-community pharmacist collaboation (PCPC).The factors that pro- mote and support PCPC remain elusive.	The objective of this review is to identify the theoretical models that have been developed to understand the PCPC, the associated instruments and to deter- mine the essential predictors of PCPC.	The specific models on PCPC reveal collabo- ration as a complex, voluntary and dynamic process. Organizational and interactional deter- minants such as role definition, willingness to collaborate and open and bi-directional commu- nication are critical to the collaboration process. The main concepts discussed in the specific models about PCPC are trust and interdependency. What is needed is a better understanding of the influence of these factors on each other.	Community pharmacy, general pratice.	[illegible]	[illegible]	[illegible]	[illegible]	[illegible]	[illegible]
27	In October 2015 the All-Party Pharmacy Group (APPG) launched an inquiry into primary and community care. The inquiry sought to understand the greater role that pharmacy could, and should, be playing in the context of the Five Year Forward View and its focus on prevention. The Group is particularly interested in population health, given the duty in the Health and Social Care Act 2012 for the Secretary of State to protect or improve public health.	To understand the greater role that pharmacy could, and should, be playing in the context of the Five Year Forward View and its focus on prevention. To explore how can service improvements be achieved and where does pharmacy fit.	Co-commision between pharmacists and GP, record sharing between pharmacists and GP, and care homes or A&E staff.	Community pharmacy, general pratice, patients.	[illegible]	[illegible]	[illegible]	[illegible]	[illegible]	[illegible]
[illegible]	[illegible]	[illegible]	[illegible]	[illegible]	[illegible]	[illegible]	[illegible]	[illegible]	[illegible]	[illegible]

52	GPs' views about community pharmacists doubtless cover the spectrum from respect to resentment and it seems it is the same for pharmacists: the representatives of both professions and their employer believe each needs to under- stand the other a bit better.	The autho describes a new guide for GPs that aims to foster a better understanding of the role of community pharmacists.	10 facts about pharmacists are provided in the article. These days there is more to being a pharmacist than dispensing prescriptions, important as that is, but GPs will only work well with their local pharmacists if they have confidence in what they do. This document should help to encourage respect between the two professions to bring that reality nearer.	Community pharmacy.	N/A	N/A	N/A	N/A	10 facts about pharmacists are as follows: 1. Pharmacists learn a lot through 4-year MPharm course and continued training; 2. There are three high-profile representative bodies for pharmacy: The General Pharmaceutical Council (GPhC, www.pharmacyregulation.org) promotes good governance and enforces professional standards. The Royal Pharmaceutical Society (www.rpharms. com) used to have a regulatory function but now offers professional leadership. The PSNC (www.psnc.org.uk) negotiates terms and conditions for NHS contractors.; 3. Pharmacists can provide three types of services to the NHS: Pharmacists who undergo training and are accredited can provide advanced services, of which there are four (medi- cines use review/prescription interven- tion, stoma appliance customisation, appliance use review and the New Medicines Service). Locally commissioned services (for- merly known as advanced or enhanced services) include anticoagulation moni- toring and palliative care. They are commissioned by NHS England (before that, PCTs) to meet demand identified in the local Pharmaceutical Needs Assessment. Local authorities and their public health services, and also clinical commissioning groups, can also commission services such as smoking cessation, supervised administration and emergency contraception.; 4. The average pharmacy earns 90–95 per cent of its income from NHS services.; 5. Funding for pharmacists is negotiated nationally between the DH, NHS England and the PSNC; 6. Reimbursement for medicines is complicated;7. Cost-saving initiatives can reduce pharmacy income; 8. A pharmacy must operate under the supervision of a single responsible pharmacist; 9. Community pharmacy income will depend more on clinical services than dispensing; 10. Patient records are covered by the same rules in pharmacies and GP practices.	
53	Multidisciplinary care (MDC) has been proposed as a potential strategy to address the rising challenges of modern health issues. However, it remains unclear as to how patients' health connections may impact on multidisciplinary processes and outcomes.	This research aims to gain a deeper understanding of patients' potential role in MDC: i) describe patients' health networks, ii) compare different care groups, iii) gain an understanding of the nature and extent of their interactions, and iv) identify the role of pharmacists within patient networks. Further investigation is required to explore factors impacting patients' network choices and their influence on MDC, particularly how we may best implement pharmacy services into patient-responsive MDC models.	New ways to expand MDC are needed to promote pharmacists as valuable members of patient's health care team.	Patients.	N/A	N/A	Multiple barriers have been reported to influence multidisciplinary collaboration. These involve determinants and processes at the interactional level (i.e., HCPs' abil- ity and willingness to collaborate), the institutional level (i.e., availability of team policies and re- sources), as well as the socio-cultural and political level (i.e., professional educational system, government policies and financial incentives). Crucial interplay and interdependence of collaborative factors highlight the complexity of MDC processes at multiple levels. In response to facilitate collaboration between HCPs, a range of interventions have been developed to implement and integrate MDC into practice. Examples include collaborative workshops for HCPs, provision of communication tools in clinics, referral processes, as well as remuneration and incentive plans. Among all HCPs reported, GPs were the dominant HCP for community group participants while respiratory specialists were the dominant HCP for clinic group participants. Both groups were found to distant themselves from community pharmacists, reporting a lack of contribution, just medication supply.	In many cases, participants were not informed or involved in any HCP collaboration or communication. In this study, patients were found to play an important role in facilitat- ing collaboration among their HCPs. Although participants' asthma networks consisted of a range of HCPs, these did not reflect or encourage MDC.<br>Community pharmacists<br>At least half of the participants from each group reported visiting a regular pharmacy; however, minimal patient–pharmacist relationships were reported. Participants' interactions with their pharmacists simply involved the purchase of asthma medica- tions and a majority felt that pharmacists made little contribution to their asthma. Those with established relationships spoke highly of their pharmacists who were proactive in identifying drug interactions, monitored participants' medica- tion use, assisted with prescription issues and provided useful patient counseling. In contrast, others felt their asthma needs were well met by other HCPs and did not require any further pharmacist support. These participants were unin- terested in the standard questions and common advice provided by pharmacists in relation to asthma. Others reported limited opportunities to interact with the pharmacist in a busy pharmacy and a large proportion was unfamiliar with the potential roles of pharmacists.<br>General practitioners<br>Participants from the community group iden- tified GPs as their first point of HCP contact, while the clinic group participants identified GPs as their back up when the specialist was unavailable. Many preferred to see a regular GP, but at times, due to location or availability, participants saw different GPs for convenience. The frequency of GP interactions depended on participants' asthma needs and health status. The majority of participants interacted infrequently with GPs and reported turning to GPs when asthma symptoms exacerbated or simply for prescription renewals. A complex patient-GP relationship was commonly identified among the community group. While many expressed frustra- tions with the lack of asthma clinical knowledge, consultation time and interest among their regular GPs, participants took no action on obtaining the asthma care they expected and returned to their regular GP. In contrast, participants from the clinic group distanced themselves from GPs, they felt they required minimal GP input and solely relied on specialist care.	Limited opportunities were presented for HCPs to collaborate, particularly pharmacists. As patients' choices of HCPs may strongly influence collaborative processes and outcomes, this research highlights the need to consider patient perspectives in the development of MDC models in primary care. This study highlights the fact that patients' health care networks composed of a variety of HCPs, lay individuals and resources. The typical asthma network relied upon two dominant health connections. This involved one specific HCP and one lay individual, such as a family member or friend. When it came to seeking professional advice for their asthma, GPs were the dominant HCP for community group participants while respiratory specialists were the dominant HCP for clinic group participants. A lack of collaborative opportunities to enhance patient asthma management was presented to HCPs, particularly pharmacists, who were continuously disregarded by participants. Although pharma- cists have the knowledge and skills to contribute to the quality use of medicines, working together with doctors to help patients better manage their asthma, such pharmacists' attributes were not recognized or utilized by participants. This study highlights the essential role of patients as active participants in MDC delivery, particularly bar- riers that patients may present to pharmacists in MDC teams.	This is the first study to have used social network principles to explore MDC in the context of patient health connections. The use of mixed-method SNA approach added value in several areas. By integrating quantitative and qualitative methods in SNA, not only did constructed networks provide an awareness of the individuals and resources involved but also insights to the content and quality of ties for those involved, and their potential impact on MDC. As patients' choices of health contact and resources may strongly influence collaborative processes and outcomes, this study has clearly showed the potential impact of patients on MDC. This finding provides a valuable starting point to further understand how patients develop their health networks and how relationships may drive collaboration among HCPs. The knowledge gained in this study provides insight to further enable MDC within patients' health networks. This research highlights the need to consider patient perspectives in the development of MDC models.
54	In recent years, community pharmacies have reported significant challenges recruiting and retaining colleagues and a high number of longstanding vacancies. Ensuring that community pharmacy has a workforce that is agile and capable of meeting the challenges posed by the healthcare systems across the UK must be a key ambition as the future roles of the pharmacist and pharmacy technician are shaped.	To enable this, the community pharmacy workforce needs to evolve with the needs of patients and the NHS. This paper recognises the ongoing ambition to better utilise the clinical skills of pharmacy colleagues and work to enable the future pharmacists to prescribe. It makes recommendations to enable the community pharmacy workforce to deliver the ambitions set out.	1. Professional bodies, higher education institutes, policy makers, and statutory education bodies should join employers to undertake a programme of collaborative work to ensure community pharmacy is seen as an attractive career choice for future pharmacists.<br>2. Pharmacy technicians should be added to Patient Group Directions as health professionals who can vaccinate patients.<br>3. Pharmacy teams should be supported to take on additional tasks, which allow pharmacists to deliver further clinical services.<br>4. Primary Care Networks should be granted flexibility to utilise funding to commission local community pharmacies to deliver services.<br>5. Community pharmacy teams need a programme of continued education. NHS and HEE should develop a programme of continued professional development and life-long structured learning for pharmacy teams, governed by the regulator, which maximises opportunities for participation.<br>6. TheGroupsupportseffortstobroadentheskillsbaseofpharmacistsvianewIE+T standards. There are a number of areas where further clarity is needed, including in relation to funding, timing and implementation. Statutory Education Bodies must ensure adequate numbers of and support for Designated Prescribing Practitioners.<br>7. Significant efforts are necessary to upskill the existing workforce to prescribers. Programmes for current pharmacists must take into account existing skills and knowledge and be arranged in a way that facilitates and supports engagement. Programmes should include modular and online options.<br>8. Current pharmacy students must be supported as the new learning standards are phased in. To create parity as current students move forward the requirement to complete two years of registration prior to undertaking a prescribing programme will need to be removed.<br>9. The NHS and other stakeholders should work with employers to develop [illegible]	Community pharmacy.	N/A	N/A	Example: Opportunities for closer collaboration between community pharmacy and primary care<br>Plans to utilise the clinical skills and knowledge of pharmacists by employing them in Primary Care Networks (PCNs) were outlined in the NHS Long Term Plan. Additional funding outlined in 2020 updates to the GP contract, enabling PCNs to directly employ six clinical pharmacists and two pharmacy technicians by 2023/24 heightened concerns that PCN pharmacists and pharmacy technicians would be recruited directly from community pharmacy into PCNs, exacerbating existing workforce shortages, in the community sector.<br>The introduction of the PCN pharmacist role is inconsistent. In some areas, for example, PCNs are struggling to recruit staff.<br>As community pharmacy looks to support the NHS in its post pandemic restoration and recovery there are clear opportunities for community pharmacy and primary care to work more closely. We are clear that community pharmacists are, for example, ideally placed to conduct Structured Medication Reviews (SMRs).<br>With appropriate funding and support (including records access) community pharmacists can complete services, including, SMRs both within the community pharmacy and off-site. This may require changes to the current funding arrangements, which stipulate the direct employment of pharmacists within PCNs.	N/A	Enhancing the skills of pharmacists through education and training, including through prescribing qualifications, will enable a change in the direction of community pharmacy allowing for the delivery of additional clinical services. The capacity of the workforce to manage patient facing care can be improved by:<br>• enabling greater use of the skill mix in pharmacies. This would allow the pharmacist to provide patient facing care while the safe and effective supply of medicines is maintained.<br>• reconsidering restrictive commissioning arrangements to enable greater integration of pharmaceutical services within primary care.<br>Whilst community pharmacy teams already play a crucial role in the delivery of health care interventions, they could be better utilised to meet the needs of local communities and tackle health inequalities.<br>Pharmacists are experts in medicines and trusted members of their communities. There is an opportunity to further position community pharmacy as an integral part of the wider health care system playing a key role in the delivery of targeted health care interventions and clinical services at a local level. This could include pharmacists spending considerably more of their time providing direct patient facing care and the delivery of a wider array of urgent and non-urgent care services in local communities. This potential could be further enhanced through the addition of increased numbers of prescribers in the community.<br>Whilst there are opportunities to both support the NHS in its post pandemic restoration and recovery and to facilitate access to care for local communities, a number of changes are necessary. These include sufficient training for current and future pharmacists and pharmacy colleagues; the commissioning of services which enable team members to utilise skills in the community; and a framework which supports the delegation of responsibilities within the pharmacy. Alongside this, the NHS should undertake work to frame community pharmacy as a central part of the health care system, within public perceptions.	
55	The Home Medicines Review (HMR) involves a home visit from an accredited HMR pharmacist to review a patient's medicines, and a report to the patient's general practitioner (GP) with recommendations for improving medicine management. Notwithstanding evidence supporting the benefits of medicines review, broad uptake by GPs in Australia remains low.	The aim of this study was to measure GPs' attitudes to the HMR and other factors which may influence the frequency with which they engage in specific HMR-related behaviours.	To foster more widespread uptake of HMRs, there is a clear need to provide opportunities for building communication and trust between GPs and pharmacists through interprofessional education and joint meetings at the local level. Findings also support the need to establish systems of working together. These might involve developing local protocols for collaboration, working with preferred accredited pharmacists enabling the establishment of a team care approach, or employing accredited pharmacists within GP practices.	General pratice.	A stratified sample of 12 divisions of general practice across Australia was prepared and a random sample of 1145 GPs was selected.	N/A	Of the GPs in our sample who returned questionnaires, just under half reported having participated in HMRs. It is noteworthy that, amongst this sub-sample, although attitudes to the HMR were generally positive and were predictors of HMR behaviours, the, HMR-related behaviours tended to occur with low frequency. It is especially noteworthy that the lowest frequency of specific HMR behaviours related to the mutual discussion by the pharmacist and GP and the pharmacists' HMR report despite being stipulated in the HMR protocol. This underscores the difficulties in taking part in case conferencing and other interpersonal communication imposed by the silo structure of primary care wherein pharmacies and GP practices are mostly separated from each other by some distance. The lack of follow-up by the GP with the pharmacist post HMR may negatively affect the downstream impact of the HMR and their professional relationship. There continue to be many eligible people who are missing out on the service and many GPs who have yet to refer eligible patients to the program. Female GPs were found to engage in HMR more frequently than male GPs, however gender had no direct association with attitudes. As expected, having a system for working together and prior positive experiences with collaboration with pharmacists was also associated with more positive attitudes to HMR and its benefits. Having a system of working together may reflect a commitment to mutual collaboration. Prior positive experiences with pharma- cists also play a pivotal role in shaping attitudes and increase the likelihood of developing a collaborative working relationship. They help build a trusting relationship between physician and pharmacist, which, as previously been identified, is critical to facilitating collaboration in pharmacist medication therapy management and medication use review. Although not necessarily designed to foster inter-professional collaboration, GPs who had participated in these had more positive attitudes towards HMR. Involvement in joint education activities has also been shown to have a positive influence on GP attitudes by offering the opportunity to explore mutual roles and raise GP awareness of the value of a pharmacist's expertise in the medication review process.	N/A	Estimates suggest that HMR is reaching only a small proportion of the at-risk population. Attitudes to HMR, gender, previous positive experiences with pharmacists, a system for working together, and participation in joint education activities predicted frequency of HMR-related behaviours. Although GPs' attitudes to HMR were generally positive, HMR-related behaviours tended to occur with low frequency. To foster more widespread GP uptake of HMRs, there is a clear need to provide opportunities for building communication and trust between GPs and pharmacists and address the systematic barriers which impeded interprofessional collaboration.	Several limitations to the study should be noted: firstly, the response rate of 33% was only modest, and may not be representative of the Australian GP population. However, the size of the sample was adequate for EFA, Rasch analysis and SEM. Secondly, the items that make up the HMRI are reflective of current HMR protocol and practice and may require amend- ment if changes occur in the future. This, however, is not a shortcoming of the theoretical model proposed, but rather a qualification that the specific items employed in the model must align with current practice.
56	To assess the performance indicators for pharmaceutical services (PS) in primary health care (PHC), the level of satisfaction with pharmacy services among users and managers / pharmacists' impressions in relation to the findings were evaluated.Although studies describing PS in Brazilian public health system already exist, it is not from author's knowledge any studies evaluating PS under the optics of management indicators and user satisfaction, linking these results with the perceptions of users, managers and pharmacists in relation to the structure and services provided.	The objective of this study was to evaluate the performance of PS actions in PHC, the satisfaction and perception of users regarding the services offered by pharmacies in health units (HU), and the impressions of managers and pharmacists on the verified data.	PS in PHC has structural and organizational weaknesses that require changes. The results indicate the necessity of improvements in relation to structural, technological, human, and organizational resources, especially in the service offered by HU pharmacies.	Community pharmacy, patients.	Only 44.4% of pharmacies had a full-time pharmacist. From the establishments visited, 5.3% did not have an air-conditioned environment, and only 33.3% of the items essential to the Good Practices of Storage of Medicines and Supplies criteria were fulfilled. Although 77.9% of the prescribed medicines were dispensed, it did not reach the 80% standard.	N/A	One of the problems found in the municipality, is prescribers' lack of familiarity with standardized medicine. In this scenario, it is assumed that one of the ways to guarantee prescriber's knowledge on medicine standardization is through his or her interaction with pharmacists.	In general, users are dissatisfied with pharmacies' services, especially with pharmaceutical care. Privacy during conversations between users and professionals is among the variables that presented the lowest user satisfaction ratings. Many pharmacies are not structured with a private room to converse with users and provide the necessary guidelines. For users, there is a direct relationship between ensuring access to medicines and care quality. Users, when referring to pharmacists, do not address issues related to guidelines concerning medicine use, but are restricted to the willingness and goodwill of these professionals. Most users did not receive adequate guidance from pharmacists on how to properly administer medicines. The users evaluated in the present study demonstrated that professional friendliness is enough to guarantee satisfaction.	The results of this study indicate that there are gaps in the infrastructure, personnel capacity, and quality of the health care provided in the PS assessed. In this sense, data revealed the fragility in the pharmaceutical-patient relationship. It was possible to observe that variables related to pharmaceutical care presented low scores in relation to other domains, thus evidencing the fragility of the pharmaceutical- patient relationship in users' perception. The scenario identified in the studied municipality –demonstrated by PS results and the perception of users, managers and pharmacists– converges with data from previous studies, which showed that the absence of full- time pharmacists in pharmacies hinders communication with users and impedes their adherence to treatment, revealing how essential their constant presence is. Routine development in pharmacies with only one part-time pharmacist contributes to impoverish the quality of the service provided. Users, managers and pharmacists expressed the need for improvements in the structure and organization of pharmacies due to the lack of sufficient space for storing medicine, ideal infrastructure for promoting PC and user accommodation. The best results related to medicine stock control reinforce the belief that computerized systems would minimize errors that may occur in manual processes, both for management and assistance provided to health service users. Computerization could streamline work processes to ensure optimal management of medicine [illegible]	The limitation of this study is the evaluation of the perception of managers. The profile of managers that agreed to participate in the study was mainly constituted by pharmaceutical services professionals, limiting the contribution of external managers' perception of the area.
57	Effective communication between prescribers of opioids and community pharmacists can con- tribute to maximizing appropriate pain management and reducing opioid misuse and diversion. While much of the education and training available on reducing opioid misuse and diversion stresses the importance of in- terprofessional communication between prescribers and pharmacists, few studies have been explored those communication patterns directly.	The objectives of this manuscript are to present and explore key emergent themes from a qualitative study around the nature, frequency, and content of communication between primary care physicians (PCPs) and pharmacists focusing on opioids.	Some pharmacist participants offered recommendations for improved communication with PCPs, such as "direct lines" being kept open for opioid consults and better non-verbal information sharing, like providing a diagnosis and other information on prescriptions. Future interventions should consider these recommendations. There are several implications from these findings. First and fore- most is that interventions for improved communication between PCPs and community pharmacists around opioids (or generically) are needed. Intervention research should seek to 1) increase motivation and expectations for collaboration among both community pharmacists and prescribers; 2) refine and clarify professional roles and standards for "mutually acceptable" topics of communication/collaboration; 3) de- velop and test models of respectful and efficient interprofessional communication; [illegible]	Community pharmacy, general pratice.	Interviews were conducted with 48 PCPs and 60 community pharmacists across four states in the U.S.: Washington, Idaho, Kentucky and Arkansas.	N/A	[illegible]	[illegible]	[illegible]	[illegible]
[illegible]	[illegible]	[illegible]	[illegible]	[illegible]	[illegible]	[illegible]	[illegible]	[illegible]	[illegible]	[illegible]

75	Many pharmacists are actively enhancing their role in the delivery of health care by offering a variety of pharmacist-led clinical services. The delivery of these services within community pharmacies can contribute to overcoming the cost and accessibility challenges currently facing U.S. health care, especially when pharmacist–physician collaborative efforts are utilized.	The objectives of this study were (1) to identify physi- cians' attitudes towards pharmacists' delivery of clinical services, (2) understand their perceived barriers to collab- oration with community pharmacists and (3) identify their recommendations regarding how to overcome such barriers. The results from this study can be used to develop interventions to promote physician–pharmacist collaboration.	Pharmacists must proactively increase overall awareness of their clinical skills, the clini- cal services pharmacists provide, outcomes from those clinical services, advantages of collaborative practice and strategies to collaborate for better outcomes for the patient. The pharmacist associations, both at national and local levels, should utilize methods to communicate the role of pharmacists in team-based care to other healthcare providers including physicians. There should also be dia- logue to alleviate physicians' concerns about loss of con- trol of patients and reimbursement challenges. The discussions should focus on the positive outcomes from collaborations, such as improved clinical outcomes, reduced physician workload, and better access and lower costs to the patients.	General pratice.	[illegible]	[illegible]	[illegible]	[illegible]	[illegible]	[illegible]
76	Physicians in Arab countries appear to be unaware of pharmacists' potential in providing primary care services to the community. Understanding physicians' attitudes towards community pharmacists providing primary care is pivotal to strategically plan or initiate interventions to improve the delivery of com- munity pharmacy services and to implement effective train- ing programmes for pharmacists.	The aim of this study was to elucidate physicians' attitudes towards pharmacists' roles in providing primary care services through community pharmacies in the UAE.	In order to enhance physicians' acceptance of pharmacist-led services, awareness of and familiarity with pharmacist services through face-to-face contact is required. The need for opportunities for pharmacists to enhance their clinical skills and possibly seek specialisation, as suggested by participants was emphasised. Guidelines should be established to ensure that these concerns are adequately addressed through a combination of legislative requirements and professional standards to ensure high-quality, ethical practice by community pharmacists in addition to changes in pharmacy layout that allow private consultation. Enforcement of regulation regarding prohibition of the sale of prescription medications without a prescription remains the most power- ful approach to help control medication access, which could favourably affect the views of physicians about the provision of primary care services in community pharmacies. There is a need for establishment of a professional pharmacy organisation in the UAE to facilitate communication with physicians and to advocate for and support initiatives taken by pharmacists to provide extended care for their patients. Such an organisation would be impor- tant in defining competency standards for the profession, setting standards of practice and delivering continuing professional education. This key issue needs to be explored by stakeholders in academic, business and regulatory sectors in the UAE.	General pratice.	[illegible]	[illegible]	[illegible]	[illegible]	[illegible]	[illegible]
77	Interprofessional collaborative care has been shown to improve patient outcomes. Physicians' views on collaboration with pharmacists give an insight into what contributes to a well-functioning team. Little is known about these views from low and middle-income countries and nothing from the United Arab Emirates (UAE).	The purpose of this study is to investigate physicians' opinions on collaborative relation- ships with community pharmacists in the UAE.	The findings suggest a need to educate physicians about what pharmacists could offer and to increase their awareness of the nature and benefits of colla- boration, possibly through face-to-face meetings. Another potential strategy that has been shown to increase awareness is interprofessional education, whether at the undergraduate, postgraduate or continuing professional education levels. This approach enables professionals to learn [illegible]	General pratice.	[illegible]	[illegible]	[illegible]	[illegible]	[illegible]	[illegible]
78	[illegible]	[illegible]	[illegible]	[illegible]	[illegible]	[illegible]	[illegible]	[illegible]	[illegible]	[illegible]
79	[illegible]	[illegible]	[illegible]	[illegible]	[illegible]	[illegible]	[illegible]	[illegible]	[illegible]	[illegible]
80	[illegible]	[illegible]	[illegible]	[illegible]	[illegible]	[illegible]	[illegible]	[illegible]	[illegible]	[illegible]
81	[illegible]	[illegible]	[illegible]	[illegible]	[illegible]	[illegible]	[illegible]	[illegible]	[illegible]	[illegible]
82	[illegible]	[illegible]	[illegible]	[illegible]	[illegible]	[illegible]	[illegible]	[illegible]	[illegible]	[illegible]
83	[illegible]	[illegible]	[illegible]	[illegible]	[illegible]	[illegible]	[illegible]	[illegible]	[illegible]	[illegible]
84	[illegible]	[illegible]	[illegible]	[illegible]	[illegible]	[illegible]	[illegible]	[illegible]	[illegible]	[illegible]
85	[illegible]	[illegible]	[illegible]	[illegible]	[illegible]	[illegible]	[illegible]	[illegible]	[illegible]	[illegible]
86	[illegible]	[illegible]	[illegible]	[illegible]	[illegible]	[illegible]	[illegible]	[illegible]	[illegible]	[illegible]
87	[illegible]	[illegible]	[illegible]	[illegible]	[illegible]	[illegible]	[illegible]	[illegible]	[illegible]	[illegible]
88	[illegible]	[illegible]	[illegible]	[illegible]	[illegible]	[illegible]	[illegible]	[illegible]	[illegible]	[illegible]
89	[illegible]	[illegible]	[illegible]	[illegible]	[illegible]	[illegible]	[illegible]	[illegible]	[illegible]	[illegible]
90	[illegible]	[illegible]	[illegible]	[illegible]	[illegible]	[illegible]	[illegible]	[illegible]	[illegible]	[illegible]
91	[illegible]	[illegible]	[illegible]	[illegible]	[illegible]	[illegible]	[illegible]	[illegible]	[illegible]	[illegible]
92	[illegible]	[illegible]	[illegible]	[illegible]	[illegible]	[illegible]	[illegible]	[illegible]	[illegible]	[illegible]
93	[illegible]	[illegible]	[illegible]	[illegible]	[illegible]	[illegible]	[illegible]	[illegible]	[illegible]	[illegible]
94	[illegible]	[illegible]	[illegible]	[illegible]	[illegible]	[illegible]	[illegible]	[illegible]	[illegible]	[illegible]
95	[illegible]	[illegible]	[illegible]	[illegible]	[illegible]	[illegible]	[illegible]	[illegible]	[illegible]	[illegible]
96	[illegible]	[illegible]	[illegible]	[illegible]	[illegible]	[illegible]	[illegible]	[illegible]	[illegible]	[illegible]
97	[illegible]	[illegible]	[illegible]	[illegible]	[illegible]	[illegible]	[illegible]	[illegible]	[illegible]	[illegible]

98	Primary care needs to be patient-centered, integrated, and interprofessional to help patients with complex needs manage the burden of medication-related problems. Considering the growing problem of polypharmacy, increasing attention has been paid to how and when medication-related decisions should be coordinated across multidisciplinary care teams. Improved knowledge on how integrated electronic health records (EHRs) can support interprofessional shared decision-making for medication therapy management is necessary to continue improving patient care.	The objective of our study was to examine how physicians and pharmacists understand and communicate patient-focused medication information with each other and how this knowledge can influence the design of EHRs.	Workable solutions to how information is shared are both social and technical. Most electronic health information systems are capable of semantic interoperability, where a receiving information system is able to clearly interpret information in exactly the same way as the sending information system.	Community pharmacy, general pratice.	[illegible]
99	There is a growing global interest in formulating such policies and strategic plans that help devise collaborative working models for community pharmacists (CPs) and general practitioners (GPs) in primary care settings. Despite the existence of many successful collaborative healthcare models in developed countries, collaboration is still not a common practice in primary care in many upper-middle-income countries, including Malaysia. The preceding two decades have witnessed an inadequate response of the Malaysian primary care system (especially in the private sector) to the delivery of well-coordinated interventions to improve management of medicines in chronic diseases largely due to poor interprofessional collaboration.	To conceptualize a stakeholder-driven framework to improve collaboration between CPs and GPs in Malaysian primary care to effectively manage medicines in chronic diseases.	CMTM type of collaborative services require greater clinical knowledge and skills for specific chronic diseases. Professional organizations representing pharmacists, such as the Malaysian Pharmaceutical Society and the Community Pharmacy Guild Malaysia, can play a leading role in training CPs for a more clinical role in chronic disease management. Future medical and pharmacy education should focus on and practise interprofessional collaboration right from the beginning to eradicate the silo-mindset that is at the root of this issue.	Community pharmacy, general pratice.	[illegible]
100	As the population has aged, it has become more dependent on health services. The GP workforce is under pressure, while there is a huge potential of community pharmacies - Many of these pharmacies are open extended hours and many are open over 85 hours a week. In almost all cases, access to a pharmacy will be far easier than a GP.	To Review Jaunty Springs Integrated Care Model.	N/A	Community pharmacy, general pratice, patients.	[illegible]
101	The NHS has been working towards the implementation of integrated care systems (ICSs) for several years and recently there has been a move to solidify this way of working through statutory changes.	This report explores the opportunities for greater collaborative working between community pharmacy, primary care networks and federations within integrated care systems, and how barriers can be overcome.	[illegible]	Community pharmacy, general pratice, patients.	N/A
102	[illegible]	[illegible]	[illegible]	Community pharmacy, general pratice.	N/A
103	[illegible]	[illegible]	[illegible]	Patients.	N/A
104	[illegible]	A narrative review of the literature identified five articles describing integration involving community pharmacies.	[illegible]	N/A	N/A
105	[illegible]	To assess Ontario family physicians' attitudes and readiness to collaborate with community pharmacists on drug therapy management.	[illegible]	General pratice.	N/A
106	[illegible]	To assess Ontario family physicians' attitudes and readiness to collaborate with community pharmacists on drug therapy management.	[illegible]	General pratice.	N/A
107	[illegible]	[illegible]	[illegible]	Community pharmacy.	N/A
108	[illegible]	[illegible]	[illegible]	Community pharmacy.	[illegible]
109	[illegible]	[illegible]	[illegible]	Community pharmacy, general pratice.	[illegible]
110	[illegible]	This study aimed to propose a model of interprofessional collaboration within the context of identifying and improving medication non-adherence in primary care.	Shared perspectives and trust constructed through regular, face-to-face interaction, embedded by co-location, is essential for successful interprofessional collaboration to improve adherence.	Community pharmacy, general pratice.	[illegible]
111	[illegible]	[illegible]	[illegible]	Community pharmacy, general pratice.	[illegible]
112	[illegible]	[illegible]	[illegible]	Community pharmacy, general pratice.	[illegible]
113	[illegible]	[illegible]	[illegible]	Community pharmacy, general pratice.	[illegible]
114	There is an innovative GP practice (Jaunty Springs) and community pharmacy in Sheffield (Basegreen Pharmacy).	To evaluate this integrated primary care model, outcome and impact.	[illegible]	Community pharmacy, general pratice, patients.	N/A
115	[illegible]	To identify and analyze factors affecting GP-CP collaboration.	[illegible]	Community pharmacy, general pratice.	[illegible]
[illegible]	[illegible]	[illegible]	[illegible]	[illegible]	[illegible]

126	The current UK government agenda is for community pharmacists to assume greater responsibility for medication management in patients with chronic conditions. This agenda may require a significant change in the way patients currently interact with their community pharmacist.	To explore the experiences and views of patients with type 2 diabetes regarding the current and proposed future role of community pharmacists.	Participants identified that, for their view to change, pharmacists needed to become more integrated into the primary care team with better communication between the different pro- fessionals. Such communication would include being referred by the doctor to the pharmacist, so validating the pharmacist's input whilst not deviating from what they saw as the natural line of treatment as well as developing procedures so that the pharmacist can communicate information back to the doctor if necessary without the patient having to visit the medical practice. profession may be need to educate patients about what the commu- nity pharmacy consultation room is like and how it can be used and remove their own perception of how patients view them. Changes to the national pharmacy contract may be appropriate to ensure pharmacists have the time and space to not only engage effectively with patients but also members of the wider primary healthcare team.	Patients.	N/A	N/A	Par- ticipants also referred to what they saw as the iso- lated position of the pharmacist within primary care, as they did not see them as fully integrated with the rest of the primary care team. In charac- terizing what the pharmacist could provide they highlighted it as lacking in joined up care and as duplicating services they received from the medical practice.	Our participants seeking information and advice would normally first approach their doctor or nurse as these professionals could make any necessary changes to medication or treatment plans. If, for some reason this was not possible either due to a problematic relationship between them or being unable to obtain an ap- pointment then the pharmacist became the next port of call. The participants in this study viewed the doctor as the 'controller' of their medication in a strictly observed health hierarchy so that they did not want to go against any treatment recommenda- tions made by their doctor by obtaining advice from the pharmacist. They saw approaching the pharmacist as violating the natural line of treat- ment. However, their discussions also made clear that participants were not simply 'anti-pharma- cist'; it was more that they did not have a clear understanding of where pharmacist expertise could be useful to them in managing their condition. Study participants highlighted that pharmacies are busy places, often full of patients collecting prescriptions or asking the pharmacist questions and this can have an impact on whether and when they feel it is appropriate to ask for advice. Pharmacists' public role and professional knowledge were not clearly understood by patients to identify which of their questions pharmacists can appropriately answer. Participants identified the existence of a relationship between the pharmacist and patient as another criterion for their being willing to engage with the pharmacist; or with any services in the community pharmacy. In terms of the pharmacy environment, most pharmacies cannot offer the privacy that this group of patients was seen to prefer to enable them to adequately discuss their medical condi- tion. Even the consultation room, with which most pharmacies are now equipped, did not escape criticism. The 'little room' as it was de- scribed is often used as stock storage space and was often small and cramped. Some patients re- ported wondering why they had been taken into the room, fearing they had done something wrong or identifying it as somewhere stigmatized groups such as drug addicts might be treated. This gives the impression that community pharmacy services and the consultation room are still not used rou- tinely by patients and as a result they are still wary of them and what to expect once in there.	The findings from this series of focus groups involving patients with type 2 diabetes indicates that they do perceive pharmacists as an essential part of the wider healthcare team, particularly with reference to allaying patients' anxiety regarding over-the-counter medicines and matters associated with the ordering of prescriptions. These are roles that have long been associated with community pharmacists and it could be expected that patients would be more familiar with these while recent changes in service provision might have impinged less on their own healthcare experiences. This group of patients clearly articulated their need to control their diabetes themselves and the importance they attached to self-monitoring was evident. This was reflected by all participants and not just those that had experienced complications or symptoms as identified elsewhere in the litera- ture. This is contradictory to the commonly held professional thought that patients know little about their medicines and their conditions and implies that many patients are fully aware of their circumstances. However, when seeking advice about their condition, they did not necessarily see the pharmacist as their first healthcare profes- sional access point.	A key limitation with this study was that it could not include the views and experiences of patients with type 2 diabetes who do not interact with the pharmacy or the pharmacist. Gaining these views would have helped provide insights into what factors would make them engage with the pharmacist about their condition and why they do not currently engage with even basicpharmacy services. Accessing and studying the views of this group would call for a different study using different methods.
127	In recent decades there has been a trend towards community pharmacists offering extended pharmacy services over and above the traditional dispensing role. Examples of extended pharmacy services aimed at improving patient care include provision of consumer medicines information (CMI), screening and monitoring of disease parameters and home medicines review (HMR). Australian pharmacists offer a range of extended pharmacy services in which collaboration with GPs is an important component. However, research into the extent of collaboration in different types of extended pharmacy services has not yet been carried out.	To explore the perceptions of Australian general practitioners (GPs) towards extended pharmacy services, and to investigate the modes and extent of collaboration between community pharmacists and GPs.	Suggestions for promoting collaboration included combined meetings and training, rapport building, information sharing and co-location of the pharmacist and GP. There is scope for strengthening the collaborative process by encouraging pharmacists and GPs to complete the prescribed cycle of HMR.	General pratice.	Twenty-three of the 68 (34%) GPs approached, agreed to participate in the interviews. Lack of time was routinely offered as the basis of non-participation. Of the 23 respondents, 4 were female and 19 were male. Nine respondents practised in their own private clinics, 12 worked in medical centres and 2 in specialised settings.	Inter-professional collaboration is defined as a joint communication and decision-making process using the unique qualities and abilities of all health professionals involved.14 It has been suggested that the pharmacist-physician collaborative working relationship progresses through a series of stages from brief interactions on routine matters (Stage 0) to a mutually beneficial relationship in which both parties have clearly defined roles and responsibilities (Stage 4). Modes of collaboration between pharmacists and GPs are diverse and include written communication (letter, note attached to a drug chart, facsimile) as well as verbal communication (face-to-face, telephone)	When respondents were asked what benefits existed for the various parties involved in extended pharmacy services, the patient was universally believed to benefit most. Respondents identified drug optimisation, increased patient comfort and timely access as the main advantages for patients. Extended pharmacy services were also believed to benefit GPs by decreasing GP workload and increasing GP drug knowledge. The most commonly reported barrier to GP acceptance of extended pharmacy services was their concern for activities that might undermine the doctor/patient relationship. Several respondents also highlighted that older GPs, whose training did not emphasise a multidisciplinary approach to patient care, were wary of the extension of pharmacy services. Other barriers identified related to the pharmacist and included pharmacy time constraints, accuracy of tests and pharmacists'competency. Contact between respondents and pharmacists occurred most often via telephone. Those who were located in close proximity to a pharmacy reported face-to-face communication. Contact frequency ranged from a couple of times per year to twice per day and generally respondents reported contacting the pharmacist as often as the pharmacist contacting them.<br>Many of the respondents agreed that there was a need for more communication between GPs and pharmacists. Generally, GPs who reported a high frequency of contact with pharmacists also tended to find input from the pharmacist more beneficial to their practice. Information sharing plays a major role in HMRs. Pathology results were provided in the majority of cases; only a few respondents reported not providing this information.Although the extent of GP-pharmacist collaboration varied, support was frequently expressed for HMRs as GPs saw it to be a useful tool in helping them manage patients.	N/A	The results of this study indicated that GP perceptions of extended pharmacy services varied according to the type of service offered by pharmacists. While GP perceptions were mostly positive for HMR, information provision and monitoring, most GPs did not favour pharmacists' provision of screening and immunisation. The GP-pharmacist relationships observed in this study existed on a spectrum ranging from Stage 0 to Stage 3 of the Collaborative Working Relationships (CWR) Model as proposed by McDonough and Doucette. In relation to screening and immunisation, the GP-pharmacists relationship displayed characteristics of a Stage 0 relationship. For some GPs and pharmacists involved in information provision and monitoring, the relationship was characteristic of Stage 1. However, HMRs were seen to be the most effective platform for expanding CWRs. The structure of HMRs produced an overall improvement in CWRs with GP-pharmacist relationships frequently falling into the Stage 3 classification as GPs reported satisfaction arising from the benefits they saw for both themselves and their patients.<br>None of the GP-pharmacist relationships observed in this study consisted of GP-pharmacist collaboration characteristic of a Stage 4 relationship, i.e. a relationship that is mutually beneficial and where each party has a clearly defined role in the care of the patient. This may be due to pharmacists fearing damaging their relations with GPs by taking on extended roles in the absence of firm agreements for a 'shared care' process.This continues to be a significant barrier to the development of extended roles for Australian pharmacists.<br>In the case of HMR services, there is a protocol with clearly defined responsibilities for the GP and the pharmacist. However, GPs acknowledged that they did not consistently send the medication management plan to the pharmacists nor did they consistently provide pharmacists with feedback. GPs only discussed the HMR report with pharmacists if they believed it to be required. This is consistent with a recent survey which indicated that feedback from the GP after provision of the HMR report was the exception rather than the rule. Overall, HMR appears to be an effective platform for inter- professional collaboration. It was widely supported by the respondents who suggested that its success may be due to four drivers: 1) perceived benefits for both patients and GPs; 2) available remuneration; 3) appointment of HMR facilitators in Divisions of General Practice; and 4) a set protocol for HMR implementation. Other drivers of CWRs identified were: 5) repeated exposure to pharmacists (leading to increased acceptance); and 6) experiencing consistently positive encounters with pharmacists.	A limitation of the study is the small number of participants. Further investigations using a larger sample of GPs are needed to determine if the findings represent the broader profession of Australian GPs.
128	Community Pharmacists and General Practitioners (GPs) are increasingly being encouraged to adopt more collaborative approaches to health care delivery as collaboration in primary care has been shown to be effective in improving patient outcomes. However, little is known about pharmacist attitudes towards collaborating with their GP counterparts and variables that influence this interprofessional collaboration.	This study aims to develop and validate 1) an instrument to measure pharmacist attitudes towards collaboration with GPs and 2) a model that illustrates how pharmacist attitudes (and other variables) influence collaborative behaviour with GPs.	Exposure to col- laboration during the final year of pharmacist training equips pharmacists with the skills and confidence for fu- ture collaboration. Policy makers may wish to consider strategies for fostering good communication, trust and respect be- tween GPs and pharmacists. One strategy may be to re- structure primary health care services so that GPs and pharmacists are collocated. This would make them more accessible to one another and thereby increase oppor- tunities for interprofessional collaboration. For pharma- cist collaboration with GPs to be successful there must also be a willingness from both parties to work together. This may be nurtured by creating educational opportun- ities that allow pharmacists more interaction with their medical colleagues in their formative years to build con- fidence and encourage teamwork. It would also allow GPs to recognise and appreciate that pharmacists have an important contribution to make to medication safety and effectiveness.	Community pharmacy.	Four hundred and ninety-two surveys were completed and returned for a response rate of 40%. The gender and age distribution is similar to that reported in the 2006 Australian Census [38] which found 47% of retail phar- macists to be male and 39% to be aged 45 years or older. The distribution of pharmacists by state/territory is also similar to that reported in the Australian Government Department of Health and Ageing Annual Report 2005– 2006	N/A	'Interactional determinants' and an 'environ- mental determinant' were shown to influence collaboration directly, while 'practitioner determinants' was found to in- directly influence collaboration. 'Interactional determinants' was found to be the strongest predictor of pharmacist col- laboration with GPs, and was in turn strongly influenced by 'practitioner determinants'. PCA of the ATCI-P indicated that items making up the variable 'trust' belong to 'practitioner determinants' rather than 'interactional determinants' as initially hypothesised. This may be because trust is linked to the individual rather than their interaction; that is, these items entail the pharmacist's assessment of the GP, rather than an assessment of their interactions. It may be argued that a positive assessment of a practi- tioner is in fact a prerequisite for strong interactions. In contrast 'recognition of roles' was found to belong to 'interactional determinants' rather than 'practitioner determinants'. This may be because perceptions on role affect how practitioners interact with one another.<br>Regarding the 'environmental determinants', only 'physician contact during training' was found to be a predictor of collaboration. The item asks pharmacists whether during their pre-registration training they rarely, occasionally or frequently had contact with GPs (if training was carried out in community pharmacy) or medical officers (if training was carried out in hospital pharmacy). Those pharmacists who had frequent contact with GPs and/or medical officers during their pre-registration training were more likely to have higher levels of collaboration with their GP counterpart in their current practice. When 'proximity to GP's office' was modelled as a pre- dictor of collaboration using Sample 1 data, those phar- macists working in closer proximity to their GP counterparts were found to have higher levels of collab- oration than isolated practitioners.	N/A	The results of the study provide evidence for the validity of the ATCI-P in measuring attitudes towards collaboration and illustrate the relationship between the frequency of interprofessional collaboration and 'interactional,' 'practi- tioner' and 'environmental determinants'. The results of the study provide evidence for the validity of the ATCI-P in measuring attitudes towards collabor- ation and support a model of collaboration in which collaborative behaviour is influenced directly by 'inter- actional' and 'environmental determinants', and indirectly by 'practitioner determinants'. The proposed model pro- vides valuable insight and a good foundation for policy makers, researchers and practitioners to develop strat- egies for pharmacists to improve collaboration with GPs in the interest of quality use of medicines and improved health outcomes. The FICI-P and ATCI-P may also be useful tools for determining the extent of collaboration in current practice and will allow for empirical assess- ment of interventions targeted at enhancing pharmacist collaboration with GPs.	Several limitations to the study should be noted: firstly, the response rate of 40% was only modest however the size of the sample was adequate for the analysis. Secondly, the items that make up the instruments are reflective of current primary care practice and may require refinement if changes to practice occur in the future. This, however, is not a shortcoming of the theoretical model proposed, but rather a qualification that the specific items employed in the model must align with current practice. It should also be noted that the ATCI-P and model have been developed for community pharmacists practicing in Aus- tralia.
129	Collaboration between general practitioners (GPs) and community pharmacists has been shown to be effective in improving patient outcomes. However, little is known about GP attitudes toward collaborating with their pharmacist counterparts and variables that influence this interprofessional collaboration.	To develop and validate, in the context of primary care in Australia 1) an instrument to measure GP attitudes toward collaborating with pharmacists and 2) a model that illustrates how GP attitudes (and other variables) influence GP-pharmacist collaborative behavior. This study has several important implications for practice and highlights possible strategies for improving GP-pharmacist interprofessional col- laboration. Whether the GP saw a role for the pharmacist in medication safety and effectiveness was the variable found to be the strongest predictor in our model. Collaboration will improve as GPs become more aware and accepting of the pharma- cist's role in medication management. One such strategy to foster this may be to create opportuni- ties for GPs to interact more with pharmacists on a professional level to gain a better understanding of the pharmacist's potential contribution to pa- tient care, for example via co-location or interpro- fessional education. Creating more opportunities for GPs to interact with pharmacists in their formative years may be an effective way to pro- mote acceptance and appreciation for the pharma- cist's role. Professional pharmacy services such as HMR are also helpful as they provide a formal platform for GP-pharmacist interaction and clearly highlight the contribution and responsibilities of each partner.	Establishment of pharmacists' poten- tial contribution early on in a physician's career is important as once perceptions of professional identities are formed they may be more difficult to change.	General pratice.	Three hundred and seventy-six surveys were completed and returned for a response rate of 33%. The gender and age distribution is similar to the Australian Government, Australian Insti- tute of Health and Welfare 2009–10 general prac- tice report31 that found 56% of GPs to be male and 35% to be aged 55 years or older.	N/A	egarding the 'environmental determinants', only 'contact with pharmacists during formative years', 'pharmacist's location' and 'participation in HMR' were found to be predictors of collab- oration. The item that made up contact with pharmacists during formative years asked respon- dents whether during their residency, they rarely, occasionally or frequently had contact with phar- macists regarding drug therapy. Those GPs who had frequent contact with pharmacists during their residency were more likely to have higher levels of collaboration with their pharmacist counterpart in their current practice. This may be due to two reasons. Firstly it may indicate that physicians who are more likely to have or seek contact with pharmacists during their residency are also more likely to collaborate with pharma- cists later on. Secondly, it may be because an effective time to expose physicians to pharmacists and raise awareness of the potential contribu- tions a pharmacist can make to medication management is during a physician's formative years. GPs working in closer proximity to their pharmacist counterparts were also found to have higher levels of collaboration than isolated practi- tioners.	N/A	The results of the study provide evidence for the validity of a 13-item ATCI-GP (items 8 and 15 removed) in measuring attitudes toward collabo- ration and illustrate the relationship of frequency of interprofessional collaboration with attitudes and other external variables. When modeled, 'interactional and practitioner determi- nants' and three separate 'environmental de- terminants' were shown to influence collaboration directly, while 'role for pharmacist in medication management' was found to indirectly influence col- laboration. 'Interactional and practitioner determi- nants', was found to be the strongest predictor of GP-pharmacist collaboration, and was in turn strongly influenced by 'role for pharmacist in med- ication management'. PCA of the ATCI-GP indicated that most of the items making up 'interactional' and 'practitioner' determinants loaded together on the same factor, suggesting that components of interper- sonal relationships such as communication and trust are strongly related to the GP's perception/ex- pectations of their pharmacist counterpart. The ex- ceptions were the two items that made up 'role for pharmacist in medication management'. The FICI-GP and ATCI-GP may also be useful tools for determining the extent of collaboration in current practice and will allow for empirical assessment of interventions targeted at enhancing GP-pharmacist collaboration.	Several limitations to the study should be noted: firstly, the response rate of 33% was only modest however the size of the sample was adequate for SEM. Secondly, the items that make up the in- struments are reflective of current practice and may require refinement if changes to practice occur in the future. This, however, is not a shortcoming of the theoretical model proposed, but rather a qual- ification that the specific items employed in the model must align with current practice. Finally, as GPs have different interactions with different pharmacists it was necessary to ask the respondents to think of only one pharmacist when completing the questionnaire. Respondents were asked to 'think of the pharmacist with whom you have most dealings'. Therefore, the results may be biased toward reflecting the relationship of more actively collaborating GP-pharmacist pairs than that of the average population.
130	The importance of interprofessional education (IPE) in continuing medical education and professional development has long been recognised by health organisations and academic societies, benefiting not only patient outcomes and interprofessional relationships but also overall health systems and work- force shortage.	This study designed and deployed the programme, Medication Overuse headache: educaTion in diagnosis, preVention, mAnagement and paTient Education (MOTIVATE), reports on the outcomes of an Australian IPE activity on medication-overuse head- ache (MOH) with general practitioners (GPs) and community pharmacists as learners.	Even though the authors of this paper have many years of combined experience in education, training and CPD development, the MOTIVATE programme was a new learning experience. Independent education providers need to be aware of the potential pitfalls when competing with pharmaceutical-company-sponsored and delivered programmes. However, it is likely that the future of accre- dited CPD education in Australia will follow the example of other countries, where the concept of education plan- ning and content will be conducted entirely independently of pharmaceutical industry involvement. The value of IPE is recognised not just by large organisations such as the WHO and governments, but by the learners themselves. Therefore, postgraduate continuing professional education should consider including all relevant professional disci- plines in education activities.	Community pharmacy, general pratice.	Three hundred participants completed both the pre- disposing activity and Module 1 (179 GPs, 121 phar- macists); of these, 147 proceeded to complete Module 2 (67 GPs, 80 pharmacists), 137 continued to Module 3 (102 GPs and 35 pharmacists), and 132 eventually completed the entire programme includ- ing the reinforcing activity (98 GPs and 34 pharma- cists).	The Society for Academic Continuing Medical Education describes interprofessional education (IPE) as "an intervention where the members of more than one health or social care (or both) profession learn interactively together, for the explicit purpose of improving interprofes- sional collaboration or the health/well-being (or both) of patients/clients".	The IPE approach suited the topic and the activity well, as it highlighted the roles and responsibilities of and between GPs and pharmacists. Working together for the common goal of patient health and recognising their respective skill sets leads to respect and interpro- fessional trust. Change in clinical practice behaviours as a result of IPE occurs when: (1) the education emphasises the participants' common goals or experi- ence; (2) encourages respect and trust; and (3) provides an opportunity to share differing opinions and experi- ences in a safe environment. This allows a coherent, collective decision rather than just a consensus to be reached. These conditions were observed during the face-to-face meetings and self-reported by the par- ticipants, but we were unable to link this to actual change in clinical behaviours, for example by way of observations in patient care setting or charts.	N/A	The MOTIVATE programme was designed to enhance insights and knowledge, and bring about change in clinical practice behaviours among GPs and pharmacists to work together in order to enhance prevention, diagnosis and management of MOH. Moreover, the programme was designed and deployed independently according to the parameters of an IME, and followed an IPE CPD model wherein GPs and pharmacists were trained together using the predisposing–enabling–reinforcing instructional framework.<br>The participants were found to have increased knowledge and improved confidence in diagnosing headaches in general, identifying patients at risk of MOH, including its likely causes based on the ICHD- III criteria and in identifying patients susceptible to MOH. They also gained confidence in their ability to educate or inform patients about MOH and setting up appropriate referral systems, if needed.	To our knowledge, our study is the first to report on an IPE programme with GPs and pharmacists in Australia. However, there are some limitations to its generalisability, as the response rate decreased at each point of measurement due to attrition of the enrolled participants. But this attrition reflects a real-life sce- nario and provides pragmatic evidence. The qualitative data reveal interesting insights to understand the com- plex reality of pushing education out to health profes- sionals in a context where a plethora of (often industry- sponsored) education is freely available.
131	While collaboration between com- munity pharmacists (CPs) and general practitioners (GPs) is essential to provide comprehensive patient care, their com- munication often is scarce and hampered by multiple barriers.	To assess both professions' perceptions of interprofessional communication with regard to content and methods of communication as a basis to subsequently develop best-practice recommendations for information exchange.	Prior to initializing research projects involving collab- orative medication management, GPs and CPs should get to know each other in order to establish mutual understanding and make common agreements regarding content and methods of communication. First of all, GPs and CPs should be supported to get to know to each other. This may be achieved by either interprofessional quality circles (an interdisciplinary group that meets to discuss work related problems or to train each other) or personal face-to-face meetings. As early as at university collaborative teaching sessions could be initi- ated. Secondly, the concept definitely has to point out how and why GPs and CPs can benefit from one another and from good collaborations. Communicating and cooperat- ing, for example, implies the opportunity to discuss and standardize important procedures, and thus work more efficiently (e.g. "CPs should call during GP's lunch hours instead of calling ten times during opening hours" or "CPs offer training how to write prescriptions correctly"). Third, the concept should acquaint GPs and CPs to collaboratively identify which method of communication suits their situation, their individual needs, and shared tasks best. Forth, the concept should encourage both professions to identify the topics and information they want to exchange.	Community pharmacy, general pratice.	Four FG (FG 1–4) taking 60–90 min were conducted from July to September 2015. The FGs consisted of four (GP:CP ratio 1:1) to five (GP:CP ratio 1:1.5) participants per group. Eight GPs and ten CPs participated in total.	N/A	Although GPs and CPs often get in touch with each other, they do not know much about each other's competencies, working conditions and statutory duties leading to little appreciation and understanding for each other's concerns. Knowing each other is a premise for sympathy and trust and one GP stated that he received a better under- standing of CPs' obligations after having talked to him. Therefore, the concept should encourage GPs and CPs to deal with each other and to make a first move towards the respective profession. Moreover, information about both professions' working conditions and statutory duties should be provided to establish better mutual understanding. The importance of GPs and CPs knowing each other's competences and tasks has been highlighted, and that their collaboration can jointly contribute to patient care. There was no general agreement between the pro- fessions regarding their preferred method of communica- tion. Both professions generally identified urgent issues they would preferentially communicate by phone. Face-to-face contact and written communication were often indicated to be used as an alternative to phone calls whereas CPs were more willing to meet than GPs. In our study, the requested types of information varied between the professions. CPs requested more items than GPs, particularly with regard to organizational issues. This might be spurred by the fact that CPs are held responsible for formal correctness of prescriptions with the threat of lacking reimbursement by health insurance if not so. In contrast, GPs were mostly interested in patient- related issues, often highlighting how these issues would support them in providing patient care. Therefore, CPs and GPs have to discuss what kind of individual arrangements should be made and why a specific type of information might be particularly relevant for one of them.	N/A	Communication between GPs and CPs is perceived dif- ferently in their daily work routine. However, both pro- fessions are generally willing to collaborate even more closely. Sympathy, trust, and motivation thereby act as important indicators. GPs and CPs prioritize similar types of information, however, they sometimes fundamentally disagree on the relevance, particularly with regard to organizational issues. While phone communication was indicated overall as the most common method of commu- nication used, participants still preferred different ways to transfer specific information. Therefore, a standardized concept should be provided that simplifies and supports GPs and CPs communication but still leaves room for individualization. The usefulness of such information is enhanced further when records between pharmacies are combined, and then, again when these are linked to medical practices. However, many new services can be provided using the information available in individual pharmacies that have a regular patient base. However, the quality of information held within patient medication records will affect the efficiency of new service provision as the more accurate and complete it is the more focused approaches to patients can be and less unnecessary duplication of effort should occur.	This study has several limitations. The results may not be representative because of the small number of partici- pants. Nevertheless, it was recruited until saturation of themes, meaning that the statements were repeated and confirmed across different FGs and IDI implying that these opinions were rather common understanding than individ- ual views. Moreover, the results might be biased by social desirability because the participants may answer in a manner that is viewed favourably by the other participants.
132	As community pharmacy services become more patient centered, they will be increasingly reliant on access to good quality patient information.	This review describes how the information that is currently available in community pharmacies can be used to enhance service delivery and patient care.	The purchase of medicines over the counter is currently an area where there is a lot of potentially useful information that could be collated either to identify patients who may benefit from screening or public health interventions or to identify abuse, which requires greater support. Empowering the patients to decide when such purchases can be recorded should increase the wealth of information available regarding patient self-medication and enable those patients choosing not to record information to be identified. Integration of patient records between different practitio- ners will enable a more accurate picture of medicines utili- zation and cost to be obtained, thus providing opportunities for tailoring services both geographically and individually. While linking of records increases the quality of information available, its eventual effectiveness will depend on whether individuals have time and sufficient incentives to access and use the collated data.	Community pharmacy, general pratice, patients.	N/A	N/A	With integration of community pharmacy and medical practice records on the horizon, the opportunities this will provide are also considered. The community pharmacy held patient medication record, which is the central information repository and has been used to identify non-adherence, prompts the pharmacist to clinically review prescriptions, identify patients for additional services, and identify those patients at greater risk of adverse drug events. While active recording of patient consultations for treatment over the counter may improve the quality of consultations and information held, the lost benefits of anonymity afforded by community pharmacies need to be considered. Recording of pharmacy staff activities enables the workload to be monitored, remuneration to be justified, critical incidents to be learned from, but is not routine practice. Centralization of records between community pharmacies enables practices to be compared and consistent problems to be identified. By integrating pharmacy and medical practice records, patient behavior with respect to medicines can be more closely monitored and should prevent duplication of effort. When using patient information stored in a community pharmacy, it is, however, important to consider the reason why the information was recorded in the first instance and whether it is appropriate to use it for a different purpose without additional patient consent. Currently, community pharmacies have access to large amounts of information, which, if stored and used appropriately, can significantly enhance the quality of provided services and patient care. Integrating the records increases opportunities to enhance patient care yet further. While community pharmacies have significant amounts of information available to them, this is frequently untapped.	The patient is a source of information that routinely enters a community pharmacy, and this has been tapped by the use of short questionnaires to enable risk assessment to be performed and inform interventions to enable them to be tailored to the individual patient.	Information stored within community pharmacy patient medication records routinely enhances patient care through the ability to identify interactions between new medicines and existing therapy and by supporting the provision of medicines to patients who have failed to obtain their medicines in a timely manner. However, it provides a multitude of oppor- tunities to further enhance patient care, many of which are currently untapped either due to a lack of resources to fund services based upon them or due to a lack of awareness of how the information could be more usefully used. When considering using patient information for purposes for which it was not intended, ie, using prescription supply information to identify patients possibly in need of a public health intervention, we need, however, to think carefully about the appropriateness of this, not only within the confines of local legislative frameworks for data management, but also from the perspective of the patient who may not be aware that their data are being used in this manner. Information held within community pharmacies is untapped as it currently stands; however, tapping it may, in certain circumstances, require a review of how the data were collected in the first place and what the patient was told the information would be used for.	
133	The practice of community pharmacy in low and middle-income countries, including in Indonesia, is often described as in the state of infancy with several intractable barriers that have been substantially and continuously hampering the practice. Such description might be valid in highlighting how pharmacy is practiced and the conditions within and beyond community pharmacy organizations. Therefore, it is not surprising that the concept of integrating community pharmacy into the primary care system may not be considered in the contemporary discourse despite the fact that community pharmacy has been operating within communities for years. However, in the case of Indonesia, we argue that changes in the health care system within the past decade particularly with the introduction of the universal health coverage (UHC) in 2014, may have significantly amplified the role of pharmacists.	To review the primary health care policy and vision for community pharmacy and pharmacists in Indonesia.	The challenge as aforementioned is to prepare graduates that can collaborate and communicate with other professionals and the patients. It is indeed an explicit call from the Association of Indonesian Pharmacy Higher Education (APTFI) for the integration of curriculum content and practice experience i.e. period of workplace learning in form of interprofessional education.	Community pharmacy.	N/A	N/A	The involvement of pharmacists in puskesmas allows for collaboration with other primary care providers within puskesmas. For example, pharmacists can assess medication appropriateness, ensure the safety, efficacy and adherence to medication, as well as provide recommendations to the other providers. In addition, pharmacists' role in puskesmas can also include the delivery of home care pharmacy programs and public health campaigns to the general public. The degree of pharmacist's involvement and collaboration, however, may vary across puskesmas, depending on their workload, given that most puskesmas have only one pharmacist in charge of providing a service to roughly 300 patients per day. The problem arises when there is a need for interprofessional collaboration which requires communication and teamwork skills. In addition to the lack of interprofessional skills, individual pharmacists have often had a lack of knowledge in particular topics and lack of individual confidence, which affects the variety of service provision to the patients. A shortcoming in the graduate's competence themselves could also be one of the causes of the lack of pharmacists' competency.Other obstacles from the individual pharmacists relate to the poor motivation from the pharmacist to be more involved in patient care, to adopt new roles and to participate in Continuing Professional Development (CPD) program.	N/A	There is good evidence which highlights the contribution of pharmacist as a substantial health care element in primary care practice. The initiative for employing pharmacist, identified in this article as primary care pharmacist, in the setting of community health center [puskesmas] and the introduction of affiliated or contracted community pharmacy under the UHC have enabled pharmacist to work together with other primary care providers. Moreover, government agenda under the "Smart Use of Medicines" program [Gema Cermat] recognizes pharmacists as the agent of change for improving the rational use of medicines in the community. Community pharmacy is developing, albeit slowly, and is able to grasp a novel position to deliver pharmacy-related primary care services to the general public through new services, for example drug monitoring and home care. Nevertheless, integrating community pharmacy into primary care is relatively a new notion in the Indonesian setting, and is a challenging process given the presence of barriers in the macro, meso- and micro-level of practice. This article promulgates the notion that the integration of community pharmacy into primary care in Indonesia is an incremental process and often requires policy initiatives to drive such changes. Community pharmacy in Indonesia can be a great example to untap the tremendous opportunity to deliver greater health outcomes. The conditions under which pharmacists are able to play a role in primary care services either via puskesmas or the network of primary care providers may suggest that pharmacist is an essential part of allied health services and integrated care. Arguably, the "Gema Cermat" program has improved pharmacist engagement with the communities which is vital for any practitioners working in the primary care sector.Major reforms are needed to reduce the barriers, improve the quality of pharmacy services and enable pharmacists to establish their ground within the primary care system strategically.	
134	Suboptimal treatment of hypothyroidism in general practice is common, and behavioural approaches to treatment by health professionals involved in the management of patients with hypothyroidism warrant exploring.	To explore the attitudes and perceptions of health professionals towards management of hypothyroidism that contributes to the suboptimal treatment of hypothyroidism in general practice.	To help increase perceived health professional control in the future, quantifying the extent that clinical, behavioural and pharmacogenomic associations may affect levothy- roxine therapy and providing this information to health professionals involved in management of hypothyroidism may improve management and therefore treatment for this group of patients. However, it should be considered that health professionals in general practice have limited time and specialism. Moreover, increasing the involve- ment of pharmacists or practice nurses in the manage- ment of hypothyroidism may improve their perceived control and help relieve the pressure on GPs, and may also increase continuity of care, for example, through pharmacist-led medication reviews or specific chronic disease management clinics for this cohort of patients.<br>To reinforce perceived health professional responsi- bility, consistent provision of patient information leaflets and discussions about hypothyroidism would help increase patient understanding and this type of information can be exchanged in a short period of time, and may reduce the time pressures on GPs if patients are more informed. Finally, clarity on the timing of initiation of treatment in asymptomatic patients with slightly elevated TSH, and an age specific and symptom focus where appropriate may help to improve the management of patients with hypo- thyroidism within general practice.	Community pharmacy, general pratice.	Sixteen one-off interviews were conducted with nine GPs, four pharmacists (P), two practice nurses (N) and one NP.	N/A	Continuity of care was perceived by GPs to be important for the management of patients with hypothyroidism. Pharmacists felt the advice they could give patients was limited by their lack of access to patient medical records, and that they could only advise depending on a patient's account of their TSH results and medications. Pharma- cists felt that access to TSH results would save time and inaccuracies in advice they give. The information exchanged between health professional and patient was limited by time restrictions, with most GPs and nurses assuming that patients would research their condition online. Written information leaflets were rarely provided to patients.	N/A	Regarding perceived professional control, health profes- sionals felt that hypothyroidism was an easy condition to manage, and felt they relied on blood tests over non-spe- cific clinical symptoms to confirm a diagnosis. However, health professionals generally perceived they had a lack of in-depth knowledge of medication interaction. On the other hand, pharmacists felt they could provide limited advice due to their lack of access to patient data and lack of time. Enablers perceived by health professionals to improve the management of hypothyroidism included continuity of care, reminders for blood tests, system alerts for medication interactions and prescription renewal, and easy access to blood tests and levothyroxine prescrip- tions for patients.	Due to time restrictions of this research project, this study did not have the opportunity to be pilot tested, which may have affected how the interviews progressed. Moreover, no feedback on the transcripts was provided by partici- pants; feedback may have contributed to enriching the data further. Since our sample only included four pharmacists and three nurses, the evidence presented from these groups may lack validity and generalisability outside of this small sample size.
135	Community pharmacists provide an extensive range of ser- vices within the three tiers of the pharmacy contract (essen- tial, advanced and enhanced), for example, Medicines Use Reviews (MURs). Further developments within the profes- sion include the implementation of extended roles such as supplementary prescribing. It is imperative that health care professionals are aware of these services and work in partner- ship to maximise patient benefit.	To explore the opinions of general practitioners (GPs) regarding the services provided by community pharmacists.	Efforts needed to set clear boundaries of responsibilities within the health care team and increased awareness of the pharmacist's role.	General pratice.	N/A	N/A	The pharmacist's role was perceived to be important to patient care and to the GP's role. Benefits included ensuring patient safety whilst dispensing medication, prescribing advice and referral to the GP.Conflicting opinions were expressed regarding the provi- sion of MURs. Some believed they benefited patient care, others saw it as a reason for increased GP workload and paper- work, with little patient benefit. The ability of some pharma- cists to undertake this form of review and their motivation was also questioned, for example, to reach company targets.<br>There was little awareness of pharmacist prescribing; par- ticipants were more familiar with nurse prescribing and con- fusion existed with regards to differentiating the roles a nurse and pharmacist would have in general practice. GPs had res- ervations surrounding the loss of their professional autonomy if pharmacists were to significantly extend their role. However, participants expressed general support for the extension of the pharmacists' role, as long as each profes- sion's responsibilities were clearly defined.	N/A	This is a small scale exploratory study which examined the views of GPs on the community pharmacist's role. Previous research has highlighted that doctors may fear for their autonomy if the pharmacy profession continues to expand. Support was expressed, however, with the caveat for clear boundaries of responsibilities within the health care team and increased awareness of the pharmacist's role. Limitations include the small sample size therefore the views expressed may not be true of all GPs. The manner of sampling may have also introduced some bias. It would have been appropriate to explore the GPs' experiences of working with their local com- munity pharmacists as this may have impacted on their views. Further work should explore the views of an increased number of GPs from around the UK.	
136	Managing chronic illness is highly complex and the pathways to access health care for the patient are unpredictable and often unknown. While multidisciplinary care (MDC) arrangements are promoted in the Australian primary health care system, there is a paucity of research on multidisciplinary collaboration from patients' perspectives.	To gain an understanding of the experiences, perceptions, attitudes and potential role of people with chronic illness (asthma) on the delivery of MDC in the Australian primary health care setting.	It might be argued that these challenges could be overcome if patients experienced collaborative health care delivery.	Patients.	N/A	Multidisciplinary care is the process of health professionals from different disciplines, for example, doctors, pharmacists, nurses and physiotherapists, working as a combined team to provide patients with comprehensive health services; so that they are continuously interacting with one another, sharing their expert knowledge, discussing and planning the patient's care together as a team.	N/A	The results of this study indicate that patients are significant 'players' in MDC and their perceptions of their chronic condition, perceived roles of HCP and expectations of health care delivery influence their views. These patients saw no need for or had no interest in receiving MDC for themselves. However, they believed that some patients may benefit from it. We propose that patients may play a significant role in the establishment of health care teams by influencing collaborative opportunities between HCP. In the present study patients sought limited interactions with any HCP in primary care, perceiving themselves as 'experts' and often engaging with HCP through intermittent and superficial encounters. That is, patients did not see a need for additional HCP interaction and presented minimal opportunities for HCP to engage with one another, particularly in the circumstances surrounding the long-term management of asthma. Instead they adopted a more self-sufficient and independent role. They did, however, turn to alternative sources (family, friends, the internet) for advice and support, developing their own asthma networks composed of individuals in addition to their HCP. This approach presents an additional challenge to implementing MDC from the HCP perspective, as engagement with the patient is difficult and consequent engagement with each other even more so.<br>Regardless of their lack of experience with collaborative health care teams, patients appeared to have developed clear views of MDC and their need for it. In this study the majority of patients were supportive of HCP working together, believing it could improve health outcomes for specific groups of patients; however, they consistently felt that it was not for them. Essentially, they did not see a personal need for MDC as they: (i) perceived their condition to be mild or well controlled; (ii) perceived that they could self-manage effectively; and (iii) accepted the nature of the care delivered by HCP.<br>When it came to their experience with MDC, patients had none and despite being aware of the concept, they did not want to find out about it. Patients were comfortable with a traditional model of care, seeking medical advice from GPs and additional advice from family and friends. This finding is perhaps the most significant of all with regard to the concept of MDC as it suggests that patients develop their own health networks or 'teams', including individuals outside the traditional medical network.<br>When it came to the expectations of different HCP, patients expressed limited understanding of their roles, especially with regard to the pharmacist.	This research is the first in the field of MDC to focus on the perceptions of patients. Our research shows that patients with asthma are personally not interested in MDC, even though they may recognise its potential benefits. Their reasoning was based on their perceptions of the severity of their illness and ability to self- manage. Patients in this study had limited experience with MDC and clearly expressed a limited understanding of the role of the different HCP groups. We can postulate that the attitudes of patients, as they were expressed in this study, make the delivery of multidisciplinary services challenging. If we try to use the knowledge gained in this study to further enable MDC, we can also view this as an opportunity to improve disease management in people with asthma, especially for those in whom asthma was clearly not as well controlled as they perceived. Being the first to focus on patients with asthma in the field of MDC, this study provides a valuable starting point in facilitating the development of patient-responsive MDC models. It highlights the need to consider patient perspectives in the development of MDC models. Future studies are required to explore the perspectives of patients with other chronic illnesses and to understand the full extent of the patient-health network and their influences. Only then can we develop models of MDC that are acceptable and sustainable.	This study took place in one particular Australian district and focussed on one chronic illness (i.e. asthma); consequently, as with most qualitative research, the findings may not be generalisable to the larger population. At this stage, we are not able to predict whether the results would have been different had we chosen to focus on another chronic illness. This needs to be explored in future research.
